# A systematic and comprehensive analysis of colorectal squamous cell carcinoma: Implication for diagnosis and treatment

**DOI:** 10.1002/cam4.4616

**Published:** 2022-02-23

**Authors:** Yang Yang, Jiarui Yu, Jitao Hu, Chaoxi Zhou, Jian Niu, Hongqing Ma, Jiaxu Han, Shaoqing Fan, Youqiang Liu, Yalei Zhao, Lianmei Zhao, Guiying Wang

**Affiliations:** ^1^ Department of Gastrointestinal Surgery The Third Hospital of Hebei Medical University Shijiazhuang China; ^2^ Department of Radiation Oncology North China University of Science and Technology Affiliated People's Hospital Tangshan Hebei China; ^3^ Department of General Surgery The Fourth Hospital of Hebei Medical University Shijiazhuang China; ^4^ Research Centers The Fourth Hospital of Hebei Medical University Shijiazhuang China

**Keywords:** colorectal squamous cell carcinoma, drug prediction, molecular mechanism, nomogram, treatment strategy

## Abstract

**Background:**

This study was aimed at establishing a nomogram for survival prediction of Colorectal squamous cell carcinoma (CSCC), understanding the molecular pathogenesis, exploring a better treatment, and predicting the potential therapeutic agents.

**Methods:**

Surveillance, Epidemiology, and End Results (SEER) database was used to obtained CSCC patients and the nomogram was performed. Propensity score matching (PSM), Kaplan–Meier analysis, subgroup analysis, and interaction test were used to explore the better treatment strategy for CSCC. Bioinformatics were used to explore the molecular mechanism and potential therapeutic drugs of CSCC.

**Results:**

A total of 3949 CSCC patients were studied. The nomogram was constructed and evaluated to have a good performance. We found that the radiotherapy had a better effect than surgery, and the difference between radiotherapy and combined therapy was not significant. 821 differentially expressed genes in CSCC were obtained from GSE6988 dataset. DNA damage repair, mismatch repair, and cell cycle pathways might contribute to CSCC occurrence as indicated by KEGGpathway and GSEA analysis. Transcription factors analysis revealed that TP63 and STAT1 may have an important role in occurrence and development of CSCC. 1607 potential drugs against CSCC were found using the CMAP database, and molecular docking was carried out to show the binding energy between TP63 and drugs.

**Conclusions:**

A good prognosis nomogram was constructed for CSCC. We also have a better understanding of the underlying molecular mechanisms of occurrence and development of CSCC and predicted potential therapeutic drugs, providing a theoretical basis for the treatment of CSCC.

## Graphical abstract

(1) The nomogram for survival prediction of colorectal squamous cell carcinoma (CSCC) was constructed; (2) Our study provided a theoretical ground for the individualized treatment of patients with CSCC. (3) We revealed potential biomarkers and therapeutic targets, explored the molecular mechanism; (4) We predicted the potential therapeutic drugs of CSCC followed by molecular docking.

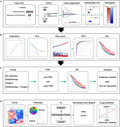

## 1 INTRODUCTION

The majority of colorectal cancer are adenocarcinoma, and squamous cell carcinoma is occasionally diagnosed, accounting for <1% of all colorectal cancer.^1^ Although colorectal squamous cell carcinoma (CSCC) is rare, the degree of malignancy is high, with the characteristics of poor differentiation and malignant metastasis.^1^, ^2^ As the low morbidity, the research for molecular mechanism of the occurrence and development of CSCC is relatively few. In addition, a few clinical studies on CSCC and few basic researches on the biomarkers and therapeutic targets of CSCC result in no significant progress in the treatment.

The tumorigenesis of CSCC is a complex process with multiple factors involved such as stem cell differentiation, colonic adenomas with squamous differentiation, and squamous differentiation induced by external stimulation.^3^, ^4^, ^5^ Other reported contributing factors associated with CSCC include chronic inflammation, smoking, HIV.^6^, ^7^, ^8^, ^9^ Moreover, HPV contamination also played an important role in the occurrence and development of many squamous cell carcinomas, including head and neck squamous cell carcinoma,^10^ anal squamous cell carcinoma,^11^ and rectal squamous cell carcinoma.^12^

Colorectal cancer treatment includes surgery, chemotherapy, and immunotherapy. Currently, the treatment of colorectal adenocarcinoma is considered highly authoritative (nccn.org), while there is no unified method for the treatment of CSCC, and the current researches on CSCC are mostly limited to rectal SCC, for which, surgical treatment,^13^ non‐surgical treatment such as radiotherapy,^14^, ^15^ or a combination of multiple treatment methods^16^ were recommended by different agencies, while, there was no unified standardized treatment for CSCC, and no large‐scale study was conducted to compare the efficacy of three treatment methods: radiotherapy, surgery, and surgery combined with radiotherapy. In short, no systematic and comprehensive study covers trend changes in incidences, survival prognosis analysis, exploration of treatment methods such as therapeutic drug discovery, and the occurrence and development of molecular mechanisms involved in the disease prognosis. We conducted relevant research that aimed at developing a survival prediction model and discovering better treatment choices for patients suffering from CSCC.

This study was targeted at a better understanding of the molecular mechanism, predicting the potential therapeutic drugs, and filling the knowledge gap of CSCC, to achieve a theoretical guarantee for better treatment of CSCC in the future.

## 2 MATERIALS AND METHODS

### 2.1 Patients

The SEER database was used for this retrospective study from 1975 to 2017. The specifications of search criteria include that the tumor must be in the colorectal area, it should be pathologically malignant and diagnosed as SCC (Histologic recode, 8050–8089), with complete survival data. Data on a total of 3949 patients of CSCC were obtained with clinical characteristics such as race, gender, age, tumor location and size, tumor grade, CEA, perineural invasion, surgery, radiotherapy, chemotherapy, and T, N, M stage. Before multivariate cox regression analysis of overall survival (OS) in training cohort, patients were grouped by radiation sequence with surgery, reason no cancer‐directed surgery, and radiation recode. Study groups were split into no surgery and radiotherapy group, radiotherapy group, surgery group, radiotherapy combined surgery group, and a group with unknown treatment method.

### 2.2 PSM

In our study, these clinical characteristics (age, gender, site, grade, T, N, M, and size) were used for propensity score matching (PSM) by R software, and patients with unknown clinical characteristics were removed. In addition, we divided size into three groups: ≤2, >2 to ≤5, and >5 cm; the ratio = 3 and caliper = 0.02.

### 2.3 Data processing

The GSE6988 data set was used to obtain microarrays of CSCC and normal colorectal tissues. Limma packet was used to obtain the differentially expressed genes (DEGs), String database was used to construct the PPI network, KEGG pathway enrichment analysis of DEGs was performed by David database, and GSEA analysis was conducted. Finally, potential therapeutic agents for CSCC were predicted by the CMAP website (https://portals.broadinstitute.org/cmap/).

### 2.4 Drug prediction

We identified 150 high‐expression genes and 150 low‐expression genes in CSCC (log Fold Change [logFC] > 1 or < −1) according to the *p* value. These 300 genes were imported into the CMAP website (https://portals.broadinstitute.org/cmap/) to obtain the potential therapeutic drugs for CSCC. Drugs with antagonistic effects were believed as the candidate anti‐tumor medicine.

### 2.5 Molecular docking

The 3D structures (sdf format) of potential therapeutic agents for CSCC were obtained from The PubChem Project (https://pubchem.ncbi.nlm.nih.gov/). And then the 3D structures were translated into“.pdb” format from the “.sdf” format by Open Bable software. The protein structure of TP63 was downloaded from the RSCB PDB database (https://www.rcsb.org/). AutoDock 4.2.6 software was used for molecular docking after removing water molecules, heteromolecules, and other operations. The lowest binding energy score was selected among 10 molecular docking. The visualization of molecular docking was performed by PyMol software.

### 2.6 Statistical analysis

The measurement data were expressed as mean ± standard deviation (SD), the counting data were expressed as frequency and proportion, and the chi‐square test or Fisher Precision Test were used for comparison. For OS, multivariate Cox survival analysis was performed by the survival package in the R software which was also used to build a nomogram. The nomogram was evaluated using concordance index (C‐index), calibration plot, receiver operating characteristic (ROC), and decision curve analysis (DCA) decision curve. For patient survival analysis, the Kaplan–Meier (KM) curve analysis was used. Univariate Cox survival analysis and interaction test were used for the analysis of subgroup, and PSM used pre‐ and post‐subgroup analysis for baseline adjustment. *p* < 0.05 was statistically significant.

## 3 RESULTS

### 3.1 3949 patients with CSCC from SEER database were used in this study

**FIGURE 1:**
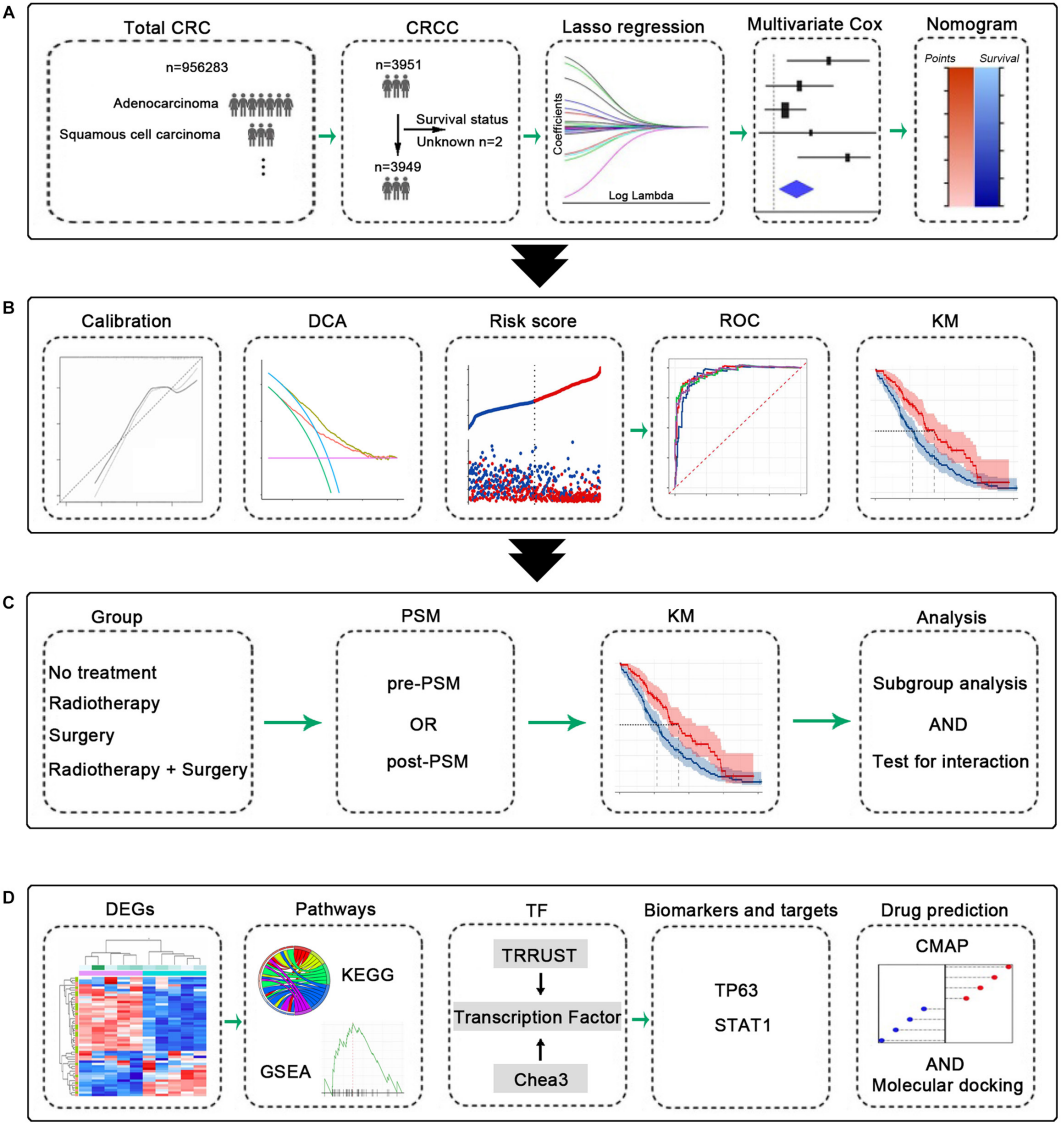
Experimental design model diagram. (A) The establishment of nomogram for predicting the survival of CSCC. (B) Evaluation of the nomogram. (C) The PSM and subgroup analysis. (D) Bioinformatics analysis, transcription factor analysis, and prediction of potential therapeutic drugs of CSCC. CSCC, colorectal squamous cell carcinoma; PSM, propensity score matching

Our experimental design was shown in Figure 1. In our study, information from 956,283 patients diagnosed with colorectal cancer was obtained between 1975 and 2017 using the SEER database, out of which 3951 patients were of CSCC, accounting for approximately 0.41% of the total colorectal malignancies, which was consistent with the data reported previously.^1^ Overall, there was a decline in the incidences of CSCC annually, with an APC of −1.4% (95% CI: −1.5 to −1.2, *p* < 0.05) (Figure S1A). This trend was more pronounced in male patients (Figure S1B).

### 3.2 Lasso regression and multivariate Cox regression analysis of OS in the training cohort

**FIGURE 2:**
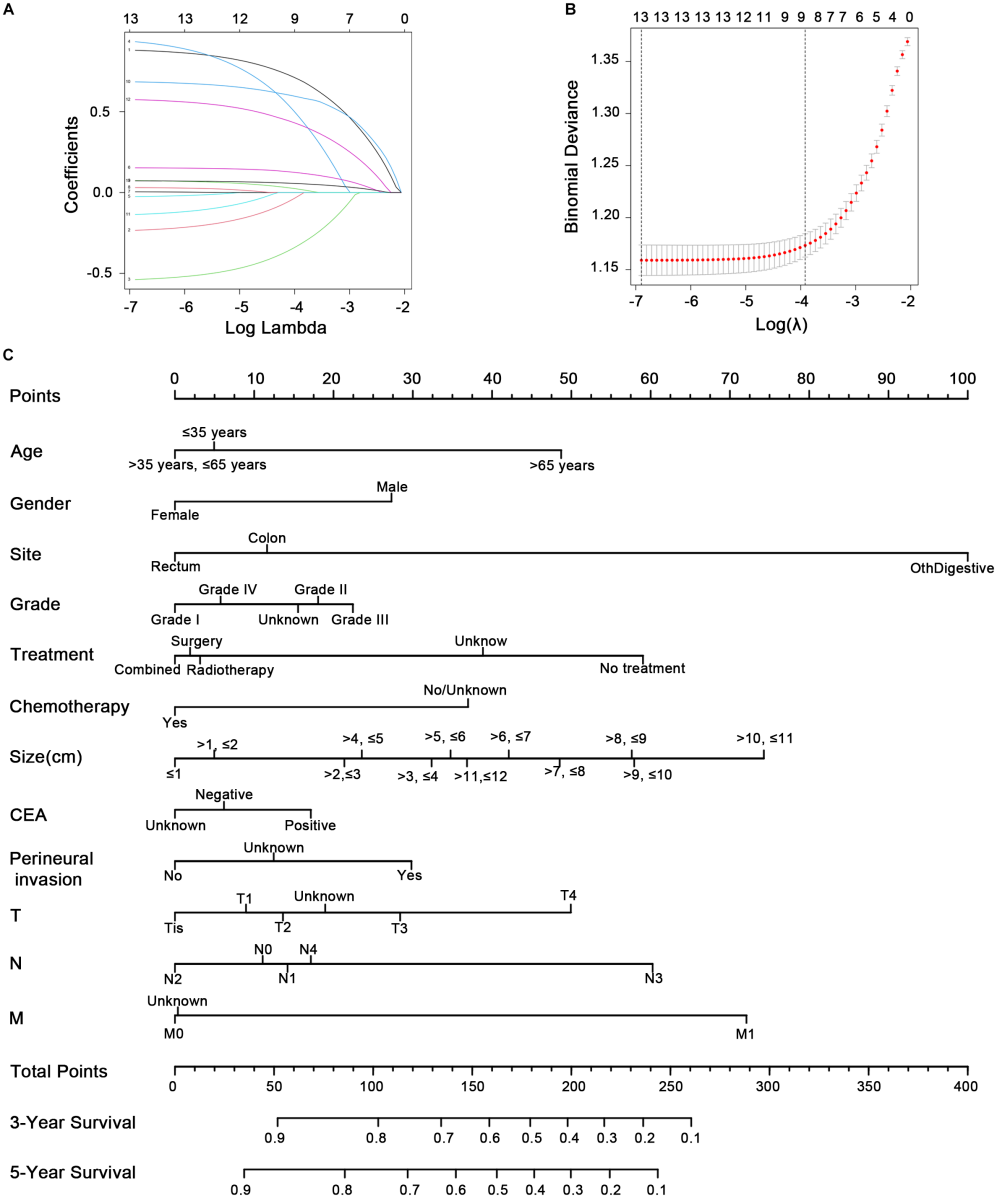
Lasso regression and nomogram for predicting the survival of CSCC. (A) The clinical features of CSCC in the Lasso model. (B) Tuning parameter (λ) selection in the LASSO model used cross‐validation via the maximum criteria. (C) Thenomogram for predicting the survival of CSCC. CSCC, colorectal squamous cell carcinoma

Given the rarity of CSCC, we aimed at the development of a nomogram model for survival prediction of CSCC. Lasso regression was performed at first (Figure 2A,B) on the clinical parameters of 3949 CSCC patients (2 patients were excluded due to no survival information), and 14 variables were obtained. Subsequently, these patients were divided into the training cohort and the validation cohort according to the 9:1 ratio by random sampling. Multivariate COX analysis showed that all factors were associated with patient survival excluding race and carcinoembryonic antigen (CEA) level (Table S1). The prognostic factors associated with OS were utilized to construct a nomogram. The CEA was included in the nomogram due to its significance. The 3 and 5 years survival rates for CSCC were shown in Figure 2C.

### 3.3 Construction and evaluation of nomogram in patients with CSCC

The C‐index (0.752), calibration plot, and DCA curve indicated the good performance of the model we established. Specifically, the probability of 3/5‐year survival from actual observation agreed with the results from nomogram prediction (Figure S2A). The DCA curves indicated that our model for 3‐/5‐year survival prediction could achieve more net benefit when the threshold probability ranges from 0.13 to 0.82/from 0.15 to 0.86 (Figure S2B). In addition, we used the survival package to get patients' risk scores, which divided patients into two groups of low‐risk and high‐risk based on the median and the risk variable plots as demonstrated by the graph (Figure S2C). The ROC curve also showed that patients of high risk had a poor prognosis (Figure S2D). Moreover, the validation cohort achieved similar results, showing good consistency (Figure S3A–C). These results fully supported the accuracy of our nomogram.

### 3.4 Survival analysis in the training cohort

**FIGURE 3:**
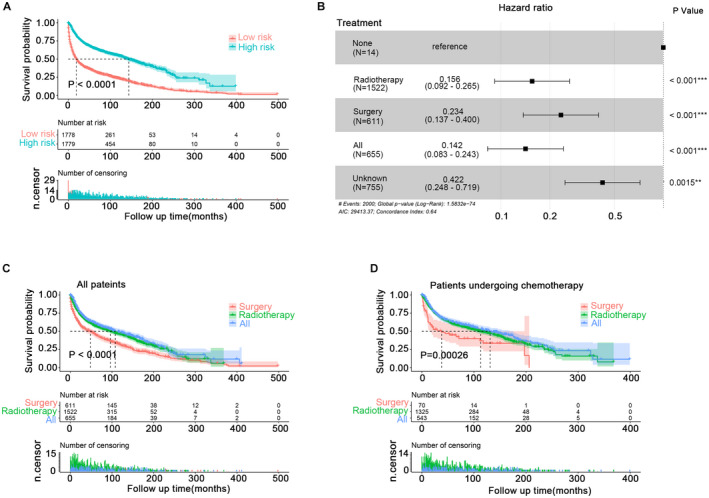
KM analysis of CSCC patients. (A) TheKM analysis of high‐risk and low‐risk populations from (C). (B) Univariate cox regression analysis of treatment. (C) The KM survival analysis of patients in groups radiotherapy, surgery, and radiotherapy combined with surgery. (D) The KM survival analysis of patients (undergoing chemotherapy) in groups radiotherapy, surgery, and radiotherapy combined with surgery. CSCC, colorectal squamous cell carcinoma; KM, Kaplan–Meier

After diving patients into high‐risk and low‐risk groups, KM analysis was done to show that high‐risk was unfavorable to the prognosis (Figure 3A). Followed, better treatments were explored. Considering that the chemotherapy status in the data was yes or unknown, further studies were performed ignoring the chemotherapy status. Univariate cox regression analysis showed that radiotherapy, surgery, and radiotherapy combined with surgery could improve the survival of patients (Figure 3B). And surgery showed a poorer prognosis as compared to the other two regimens (Figure 3C). Moreover, a similar result was achieved in the patients undergoing chemotherapy (whose chemotherapy status was yes) (Figure 3D).

PSM was done to distinguish the survival difference between radiotherapy and radiotherapy combination with surgery (the clinical characteristics of the patients are shown in Tables S2 and S3). The KM curve showed that pre‐PSM and post‐PSM, independent of the chemotherapy status, there was no significant difference between radiotherapy and radiotherapy combined with surgery (Figure S4A–D). PSM was performed again for comparison of survival between radiotherapy and surgery (the clinical characteristics of the patients are shown in Tables S4 and S5). The KM curve showed that pre‐PSM and post‐PSM, with or without chemotherapy status determination, survival of radiotherapy alone was better than surgery (Figure S5A–D).

### 3.5 Subgroup analysis

**FIGURE 4:**
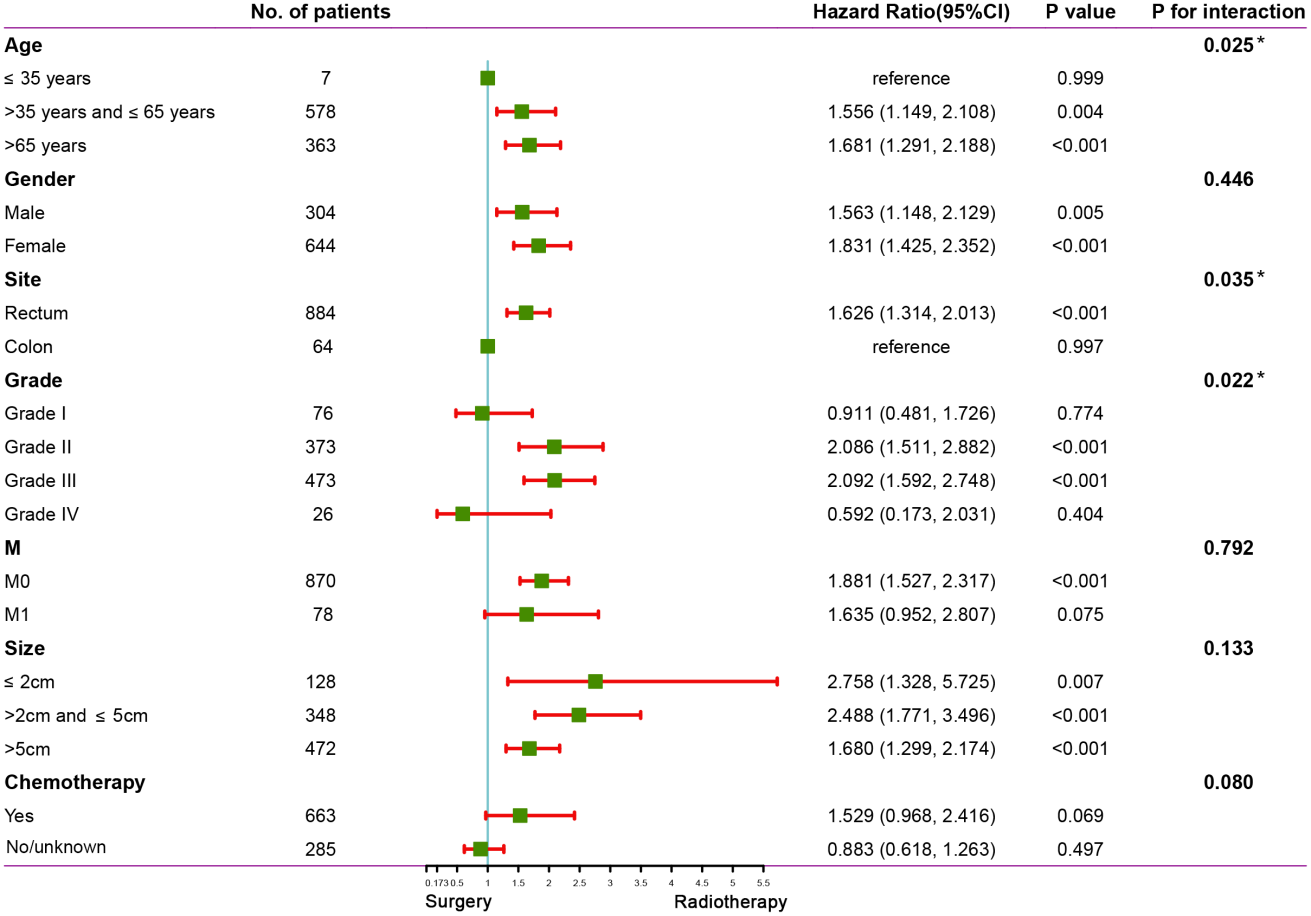
Subgroup analysis and interaction test between surgery and radiotherapy

To further explore a more effective treatment for CSCC patients, we performed a subgroup analysis for patients treated only with radiotherapy and those treated only with surgery (the patients were those in Table S4, pre‐PSM). The results showed that the interaction test (gender, distant metastasis, and size) was insignificant. For patients over 35 years old, with rectal lesions, grade II or III, receiving chemotherapy, regardless of gender, whether distant metastasis or not, regardless of lesion size, radiotherapy was superior to surgery (Figure 4). Tumor metastasis is an important problem that could not be ignored. The 1‐year survival rate and median survival time of advanced patients were shown in Table S6. In addition, there was no significant difference in survival among various metastases (Table S7).

### 3.6 Exploring the underlying molecular mechanism of CSCC

So far, the molecular mechanism of SCC was unknown. We tried to explore the occurrence and study the underlying molecular mechanism of CSCC. The GSE6988 data set provided us with a total of 821 DEGs between CSCC and normal mucosa (logFC >1 or < −1, FDR < 0.05) (Figure S6A). Most of the top 10 highly expressed genes (TP73L, CSTA, KRT5， DSG3， TRIM29， FOSL1， NEDD1， CALML3， CSRP2， and PMAIP1) sorted by FDR were also highly expressed in colorectal adenocarcinoma tissues, such as TP73L (also known as TP63)^17^, ^18^ and TRIM29.^19^ Our study indicated the overexpression of 528 genes out of the total and 293 genes were under‐expressed in CSCC as compared to the gene expression levels in the intestinal mucosa (Figure S6B). The PPI network for 821 DEGs was constructed through the STRING database (https://www.string‐db.org/) (Figure S6C) and 15 hub genes were obtained (Figure S6D). However, survival analysis of these genes could not be performed due to the lack of cases. Then KEGG pathway analysis was performed using the DAVID database (https://david.ncifcrf.gov/) for the 821 genes and the results suggested that CSCC may be associated with DNA damage repair, mismatch repair, and cell cycle pathways (Figure S6E). In addition, similar results were obtained by GSEA analysis (Figure S6F).

**FIGURE 5:**
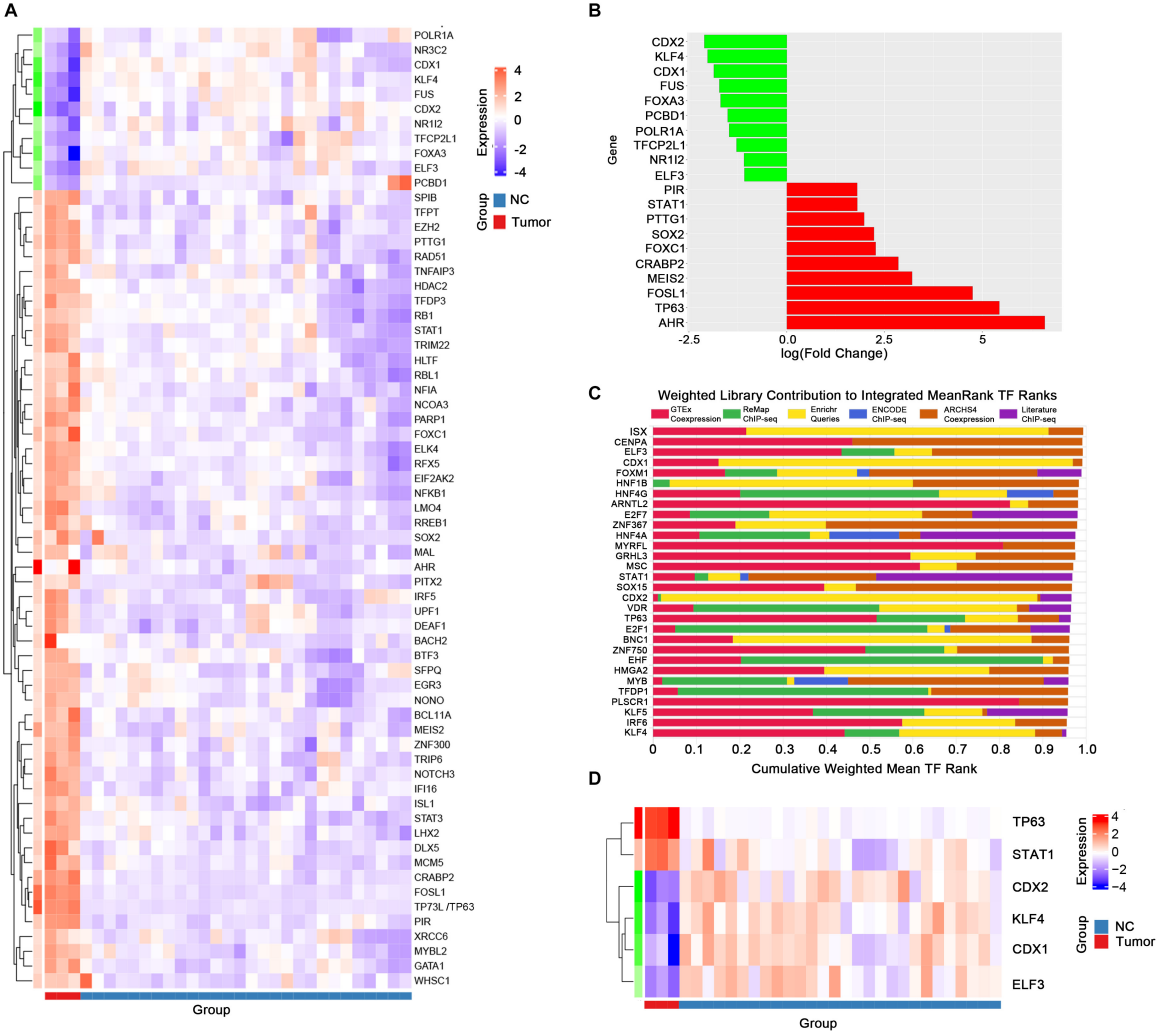
Transcription factor analysis of genes related to CSCC. (A) Thermograms of differentially expressed transcription factors. (B) The top 10 transcription factors up and down with the LogFC. (C) The top 30 transcription factors by mean ranking in the Chea3 database. (D) Six of the 30 transcription factors from Figure 5C were overexpressed or underexpressed in CSCC. CSCC, colorectal squamous cell carcinoma

The upstream transcriptional regulators of these DEGs were also explored. First, 821 DEGs were mapped with transcription factors in the TRRUST database and the results showed overexpression of 54 transcription factors and underexpression of 11 transcription factors in CSCC compared with normal mucosa (Figure 5A). Figure 5B showed the top 10 up and downregulated transcriptional factors. For searching more potential key transcription factors in CSCC, the Chea3 database was used for transcription factor enrichment analysis for 821 DEGs. The top 30 transcription factors were listed in Figure 5C, among these two transcription factors (TP73L and STAT1) were overexpressed and four transcription factors (CDX2, KLF4, CDX1, and ELF3) were underexpressed in CSCC (Figure 5D) (LogFC > 1 or < −1). Further results showed that all six transcription factors obtained from the Chea3 database were fully included in the top 10 up and down‐regulated transcription factors in the TRRUST database, which revealed that the six genes may have a significant role in SCC.

In addition, we found that three (CDX1, CDX2, and KLF4) of the four transcription genes (CDX2, KLF4, CDX1, and ELF3) with low expression in CSCC also showed low expression in colorectal adenocarcinoma,^20^, ^21^, ^22^, ^23^ while ELF3 achieved the opposite result in colorectal adenocarcinoma.^24^ For the two transcription genes highly expressed in CSCC, TP63 could be used as a poor prognostic marker of colorectal adenocarcinoma, however, the oncogene function of STAT1 in colorectal adenocarcinoma was controversial.^25^, ^26^

### 3.7 Drug prediction for the treatment of CSCC and the molecular docking between TP63 protein and potential therapeutic drugs

**FIGURE 6:**
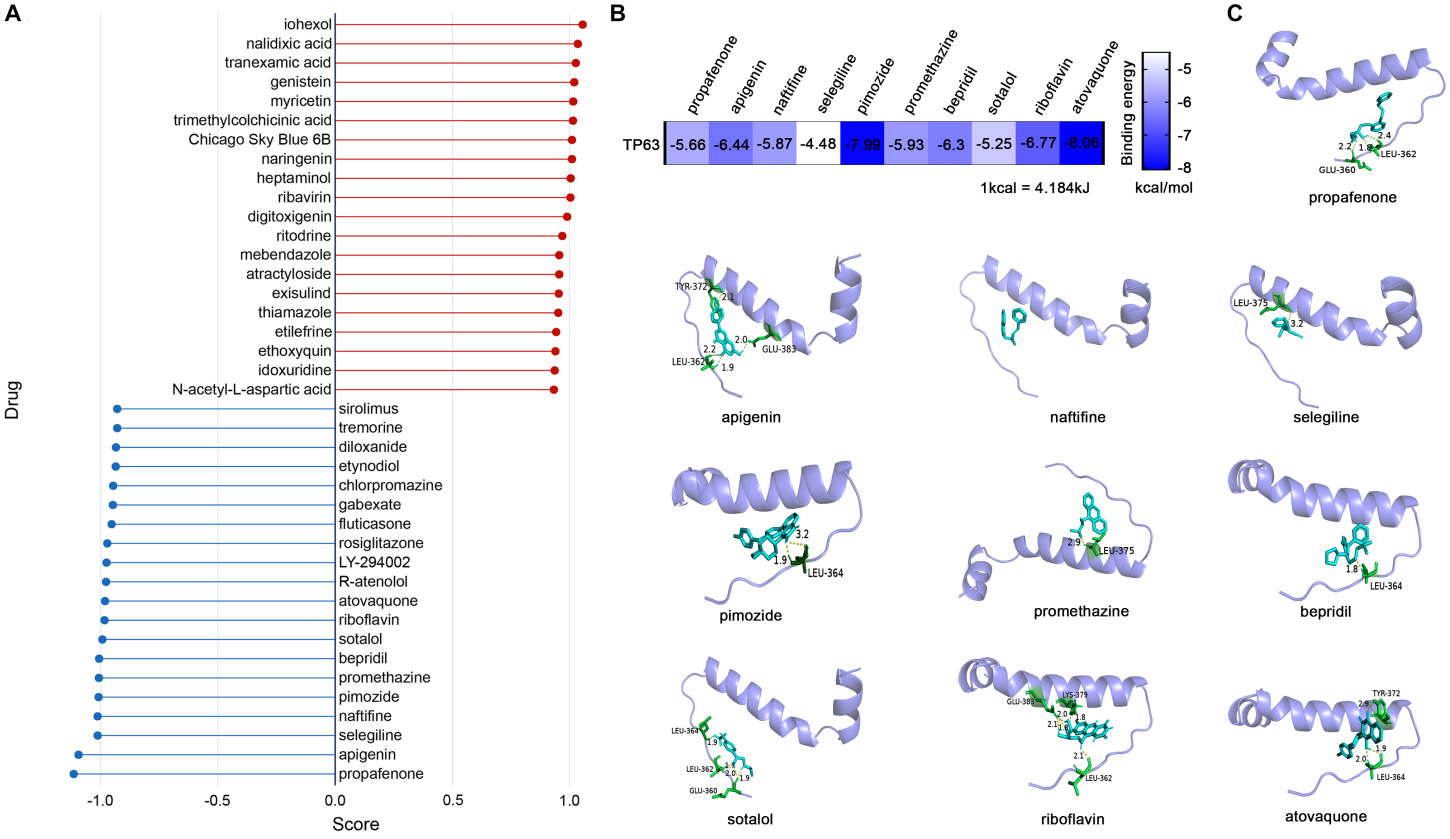
Predicting the therapeutic agents of CSCC through the CMAP database and molecular docking. (A) The potential therapeutic agents of CSCC. (B) The binding energy estimation between TP63 protein and top 10 negatively related drugs. (C) The molecular models of the binding between TP63 protein and top 10 negatively related drugs. CSCC, colorectal squamous cell carcinoma

It is still devoid of an effective drug for CSCC at present, so related drug research is therefore of great significance. CMAP database was utilized to predict potential therapeutic agents for CSCC. We selected 150 high‐expression genes and 150 low‐expression genes (logFC >1 or < −1). The database suggested 6100 drugs (Table S8). As shown in Figure 6A, 20 drugs were listed with positive correlation and 20 drugs with negative correlation ranking by score. The 20 drugs negatively correlating to gene arrays were opposed to SCC, indicating that they have the potential to act as antagonists in CSCC. These drugs are mostly have been used in clinical drug treatment for many years. For example, propafenone is an oral antiarrhythmic agent; selegiline is used to treat depression or Parkinson's disease; pimozide is a conventional antipsychotic (https://pubchem.ncbi.nlm.nih.gov/). Following this, binding energy estimation between TP63 protein and top 10 negatively related drugs were carried out (Figure 6B). The molecular models of the binding between TP63 protein and top 10 negatively related drugs were shown in Figure 6C, revealing a certain affinity.

## 4 DISCUSSION

The current research was the first comprehensive and systematic study of survival prediction, molecular mechanism, and therapeutic drugs for CSCC. We revealed the decreasing trend of CSCC incidences and established a survival prediction model. Besides, it was the first attempt to understand the potential molecular mechanism of CSCC and to develop more effective treatment methods and explore potential drugs for patients, which played an important role in guiding the treatment of CSCC patients.

Very little clinical information was available on the prognosis of CSCC. A promising survival prediction model of rectal SCC has been reported previously, however, it was limited to the rectum and its analysis also excluded some clinical features such as perineural invasion and CEA.^27^ Therefore, in this study, a nomogram was established that included clinical features such as tumor grade and location, perineural invasion, and CEA to predict prognosis in CSCC patients. The effective performance of the nomogram was supported by analyzing the C‐index, Calibration plot, ROC curve, and DUA decision curve.

As far as the treatment of rectal CSCC is concerned, some studies suggested that surgery can improve patient survival,^1^, ^13^ while others suggested that chemotherapy and radiotherapy are better treatments of choice.^28^, ^29^ However, the treatment of CSCCsupported by large samples has not been studied. Here we found that regimens including surgery, radiotherapy, and surgery combined with radiotherapy can improve patient survival but the surgical group showed the least improvement among the other two groups. The reason may be that radiotherapy was very favorable in improving prognosis. Of course, as shown by subgroup analysis, radiotherapy was superior to surgery for some specific patients (not all patients). For example, the study had shown that precision radiotherapy was better than surgery for early lung cancer.^30^ Our study provided a theoretical ground for the individualized treatment of patients with CSCC, which was beneficial for the accurate treatment of CSCC.

We analyzed the DEGs of CSCC for the first time, revealing potential biomarkers and therapeutic targets. KEGG and GSEA analysis suggested that CSCC was related to mismatch repair, DNA damage repair, and cell cycle, which provided a further understanding of the occurrence and development mechanism of CSCC and a direction for future research. Additionally, we firstly found that the transcription factor enrichment of DEGs in CSCC suggested that TP73L (also known as TP63) and STAT1 may have a significant role in CSCC. TP63 played an important role and was amplified in numerous SCCs.^31^, ^32^, ^33^ In addition, TP63 could be used to differentiate tissues affected by CSCC from other tissue subtypes.^34^, ^35^ Overexpression of STAT1 was observed in many SCCs and can promote the malignant ability of SCC.^36^, ^37^, ^38^ Our findings suggested that TP63 and STAT1 may play a critical role in CSCC, and more studies on their mechanisms are necessary for the future.

To discover better therapeutic drugs for CSCC, we predicted potential therapeutic drugs, such as propafenone, apigenin, rosiglitazone, and so on. Many drugs had been reported to have antitumor effects.^39^, ^40^, ^41^ What is more, these old drugs had been used for many years, showing the safety of the drugs. However, the anti‐cancer effects of these drugs in CSCC need to be verified in the future.

The limitations of this study include incomplete treatment protocols from the SEER database and some clinical features, such as lymphatic vessel invasion, were lacking. In addition, the rare cases of CSCC made it impossible to verify DEGs, analyze the survival of hub genes and test potential therapeutic drugs. However, large samples could not be obtained through a prospective study of CSCC, therefore, analysis through the SEER database seems to be the best way at present.

In summary, our study is the most systematic and comprehensive study on CSCC. We not only analyzed the clinicopathologic features of CSCC but also analyzed the treatment methods of this disease. Finally, we explored the molecular mechanism and predicted the potential therapeutic drugs for CSCC, which brought a thorough understanding of the disease and provided a theoretical basis for its future treatment.

## CONFLICT OF INTERESTS

The authors declare no competing interests.

## AUTHOR CONTRIBUTIONS

Guiying Wang and Lianmei Zhao conceived and designed the project. Yang Yang and Jiarui Yu constructed and verified the survival prediction model, made figures, and tables, and wrote the manuscript. Jitao Hu and Chaoxi Zhou carried out the PSM and KM analysis. Jian Niu and Hongqing Ma performed the bioinformatics analysis. Jiaxu Han predicted the potential therapeutic agents for CSCC by CMAP database. Shaoqing Fan downloaded and organized data from SEER database and GEO database. Youqiang Liu performed molecular docking. Yalei Zhao reviewed the literature.

## ETHICS STATEMENT

Not applicable.

## Supporting information


Figure S1

Figure S2

Figure S3

Figure S4

Figure S5

Figure S6

Tables S1–S8

## Data Availability

The research data could be obtained from SEER database and GEO database, or obtained from the first author.
