# Supplementary figures and images for: A systematic and comprehensive analysis of colorectal squamous cell carcinoma: Implication for diagnosis and treatment
Source: Cancer Med. 2022 Feb 23;11(12):2492–502. doi: 10.1002/cam4.4616 (PMC9189455; doi:10.1002/cam4.4616)

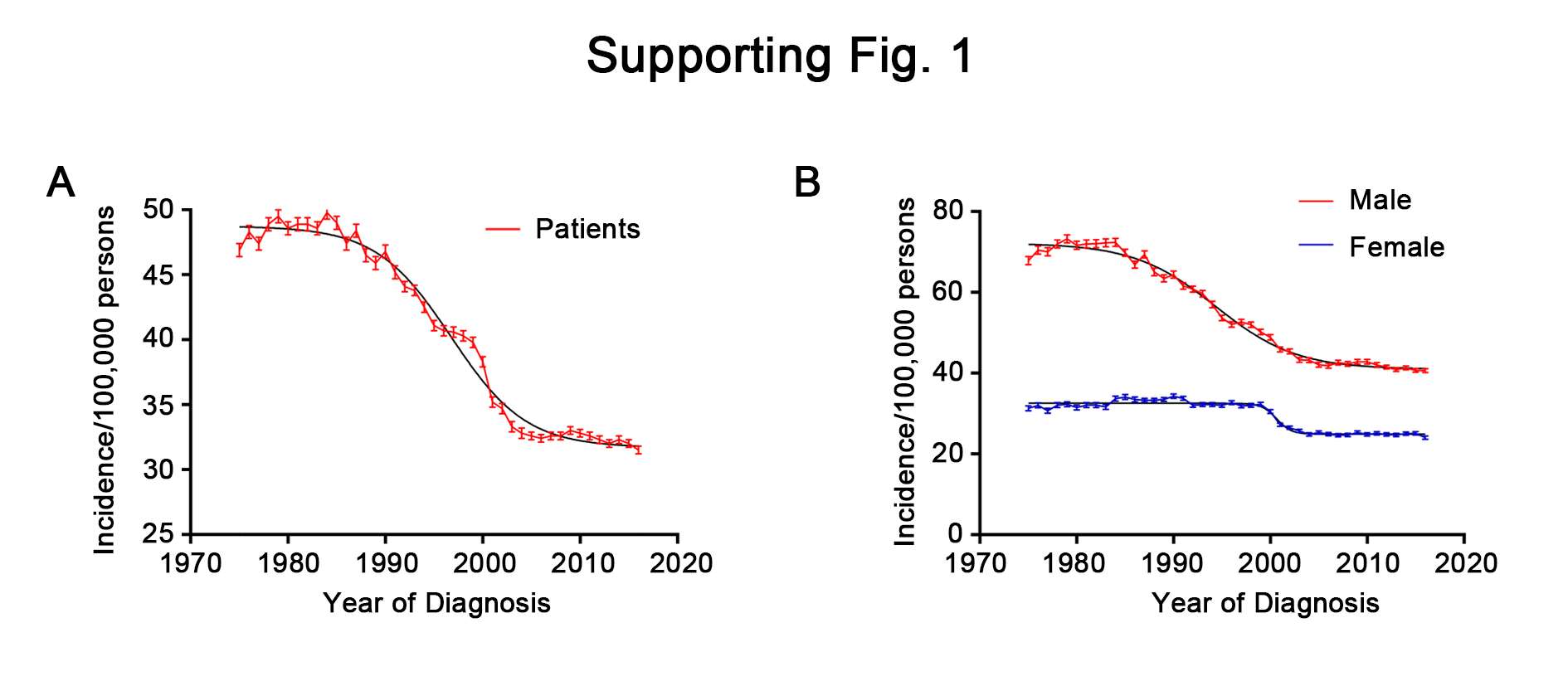

Supplement: Supplementary file 1 — Figure S1 [file CAM4-11-2492-s002.tif]

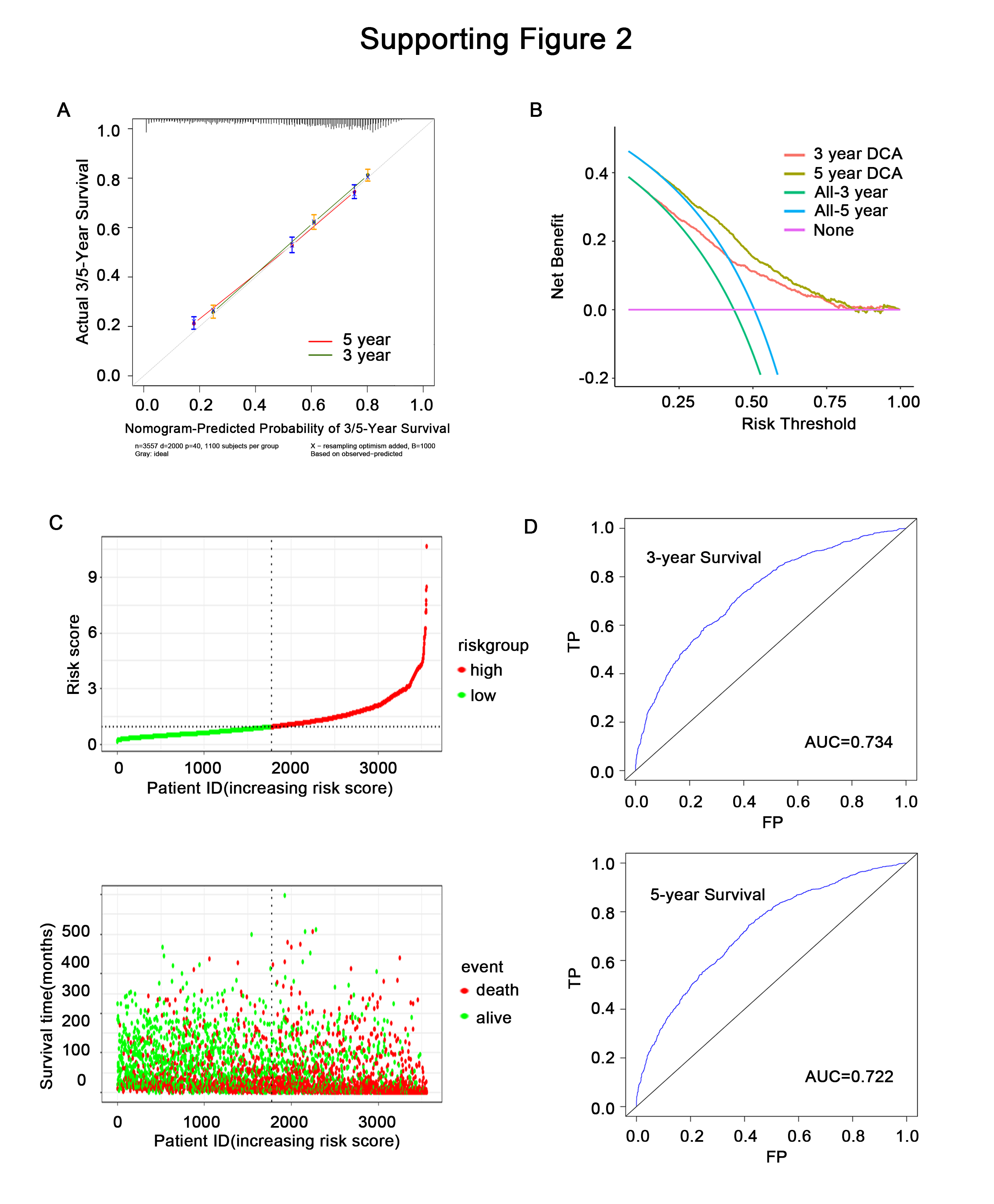

Supplement: Supplementary file 2 — Figure S2 [file CAM4-11-2492-s003.tif]

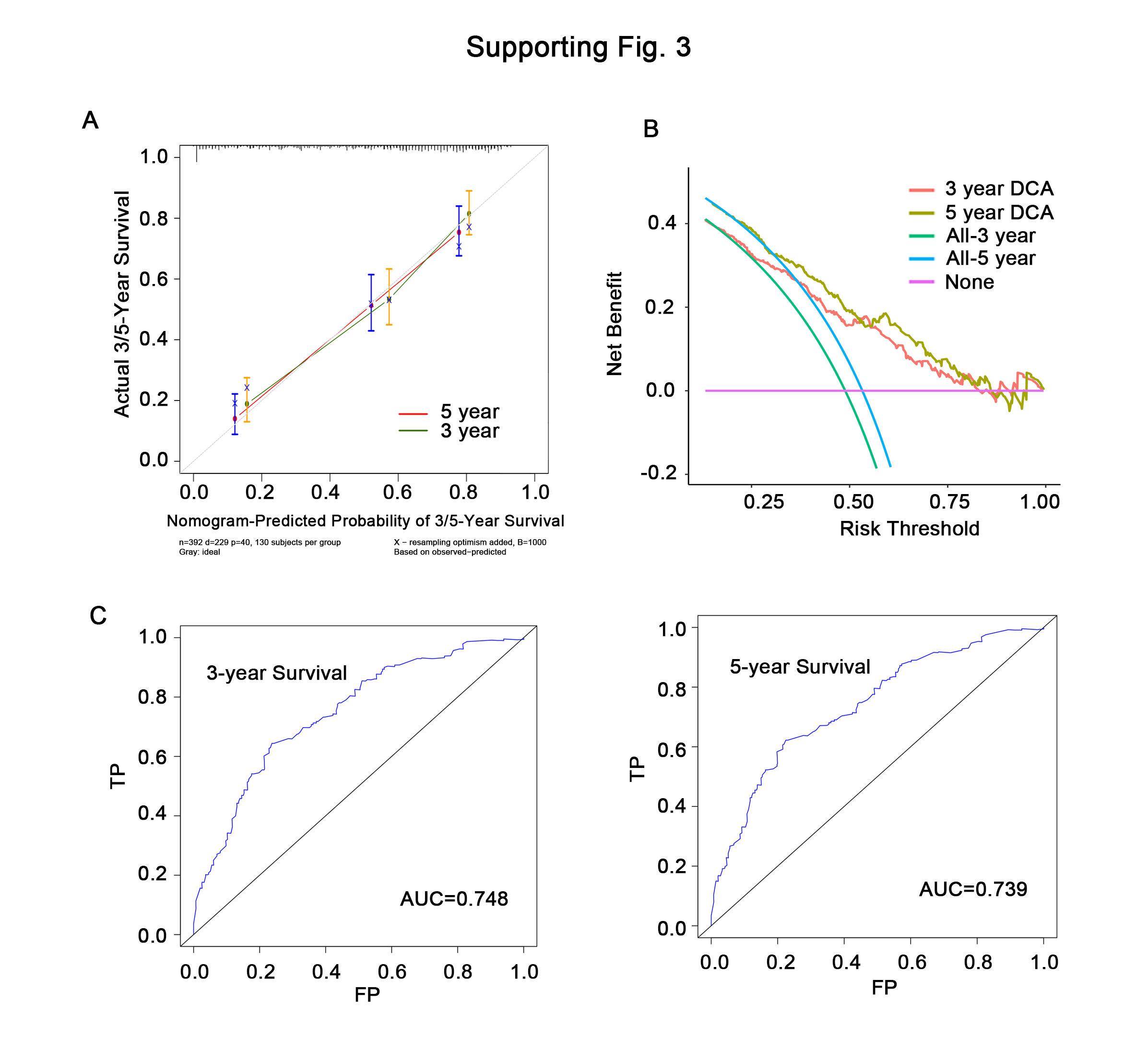

Supplement: Supplementary file 3 — Figure S3 [file CAM4-11-2492-s006.tif]

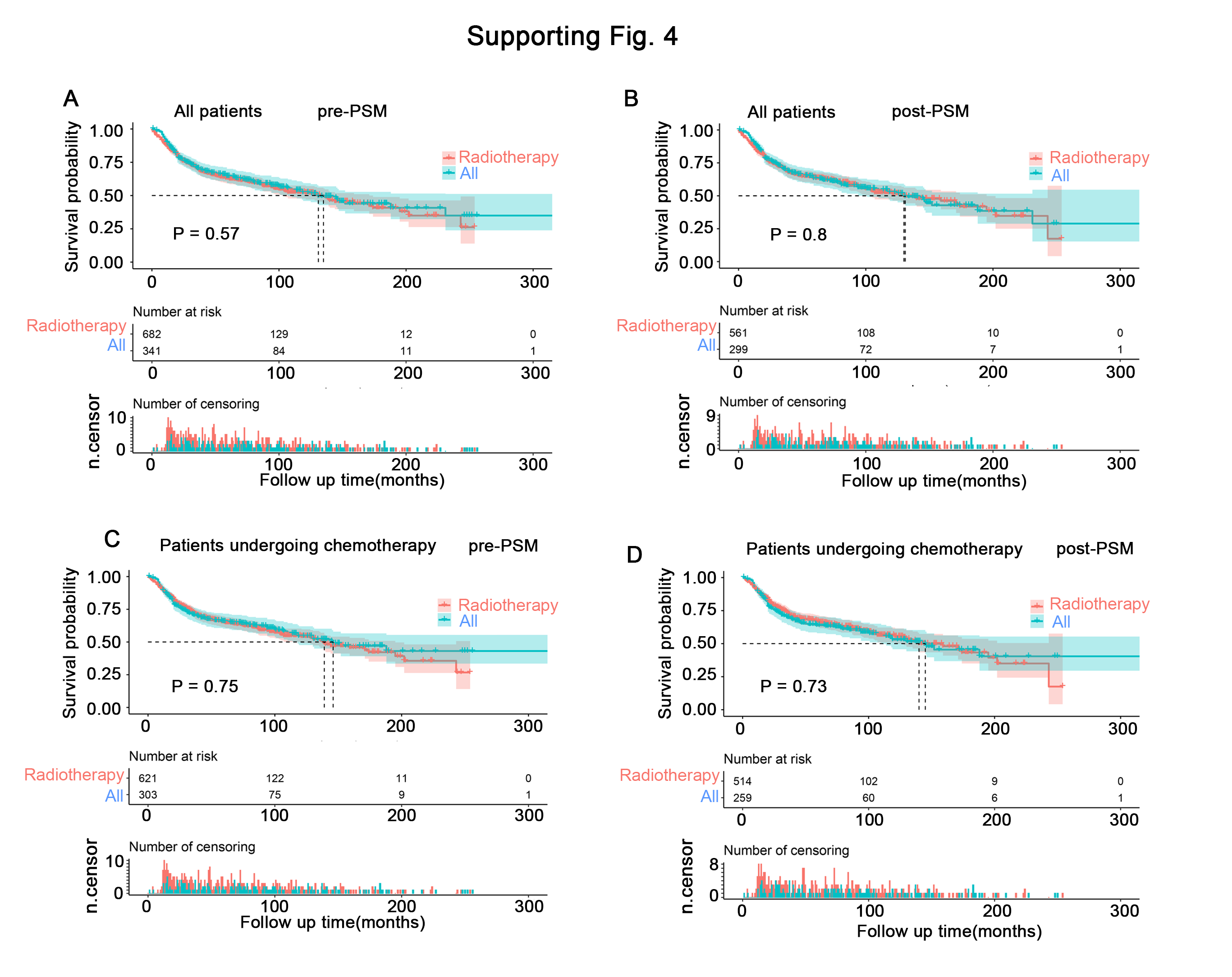

Supplement: Supplementary file 4 — Figure S4 [file CAM4-11-2492-s007.tif]

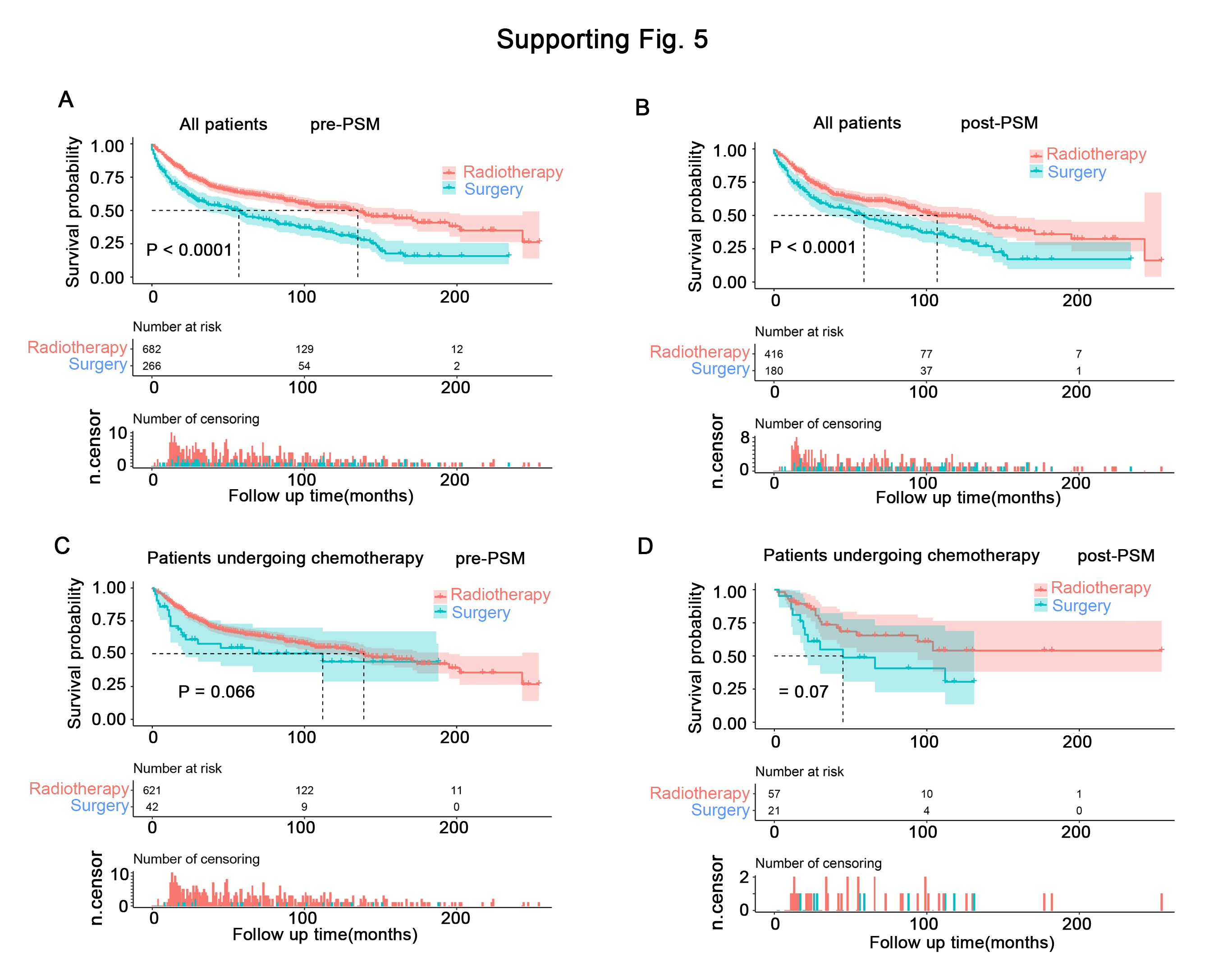

Supplement: Supplementary file 5 — Figure S5 [file CAM4-11-2492-s005.tif]

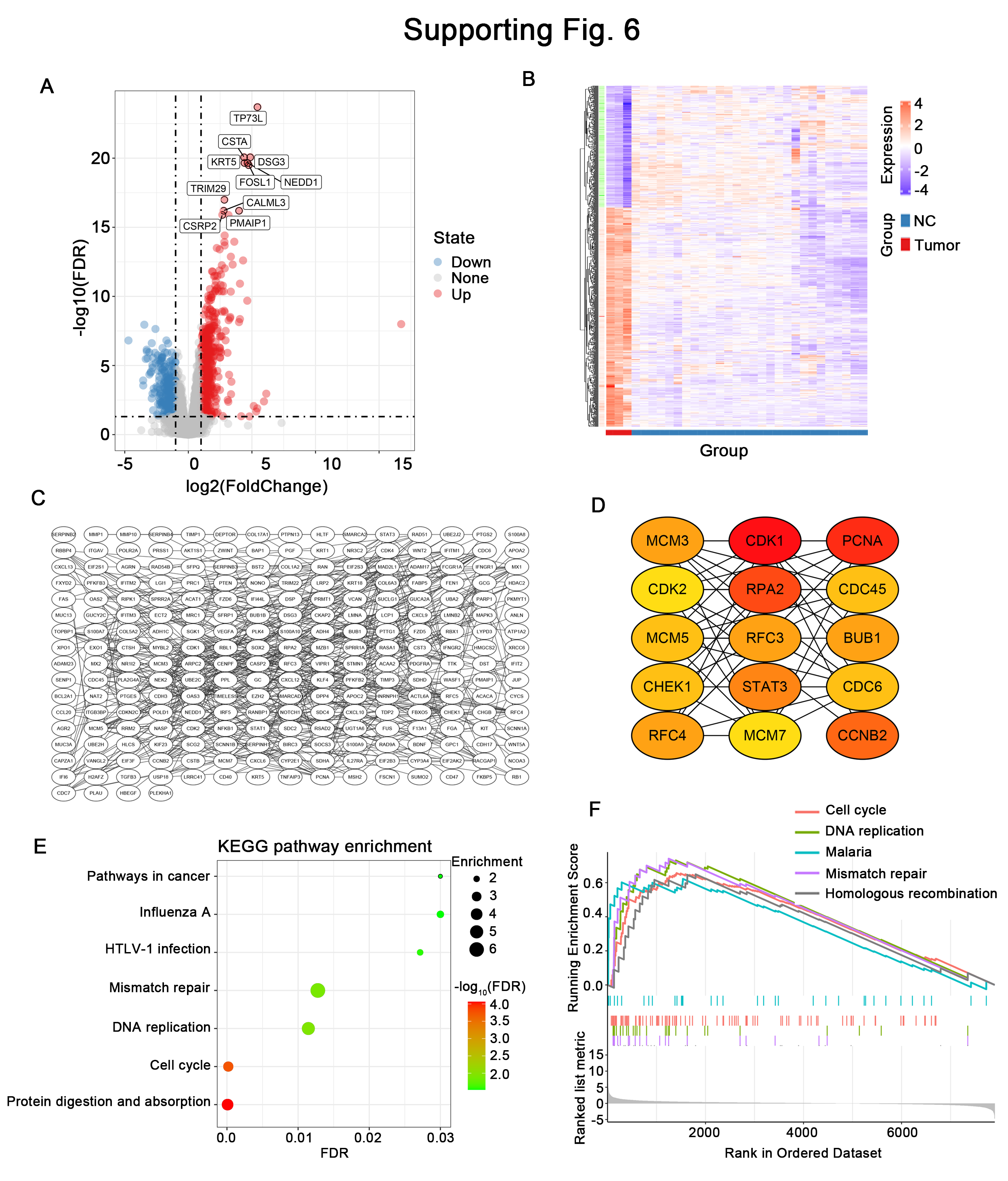

Supplement: Supplementary file 6 — Figure S6 [file CAM4-11-2492-s004.tif]
