# Supplementary material for: A systematic and comprehensive analysis of colorectal squamous cell carcinoma: Implication for diagnosis and treatment
Source: Cancer Med. 2022 Feb 23;11(12):2492–502. doi: 10.1002/cam4.4616 (PMC9189455; doi:10.1002/cam4.4616)
Supplement: Supplementary file 7 — Tables S1–S8 [file CAM4-11-2492-s001.docx]

**Supporting Tables**

**A systematic and comprehensive analysis of Colorectal squamous cell carcinoma: implication for Diagnosis and treatment**

Yang Yang, M.M.,^1,†^, Jiarui Yu, M.M.,^4,†^, Jitao Hu, M.M.,^2^, Chaoxi Zhou, M.D.,^2^, Jian Niu, M.M.,^2^, Hongqing Ma, M.M.,^2^, Jiaxu Han, M.M.,^2^, Shaoqing Fan, M.M.,^2^, Youqiang Liu, M.M.,^2^, Yalei Zhao, M.D.,^2^, Lianmei Zhao, Ph.D.,^3,*^, & Guiying Wang, M.D.,^1,2,*^

Affiliations:

^1^ Department of Gastrointestinal Surgery, The Third Hospital of Hebei Medical University, Shijiazhuang, 050011, China

^2^ Department of General Surgery, The Fourth Hospital of Hebei Medical University, Shijiazhuang, 050011, China

^3^ Research centers, The Fourth Hospital of Hebei Medical University, Shijiazhuang, 050011, China

^4^ Department of Radiation Oncology, North China University of Science and Technology Affiliated People's Hospital, Tangshan, Hebei 063000, China

† These authors contributed equally to this work.

Correspondence to: *To whom correspondence should be addressed. Guiying Wang, The Third Hospital of Hebei Medical University/ The Fourth Hospital of Hebei Medical University, Shijiazhuang, China. Tel: 86-0311-8609-5347, Email: [wangguiyingtgzy@163.com](mailto:wangguiyingtgzy@163.com); Lianmei Zhao, [zhaolianmei@hbydsy.com](mailto:zhaolianmei@hbydsy.com).

**Supporting Table 1.** Multivariate Cox survival analysis of CSCC

| Variable | | HR(95%CI) | P |
| --- | --- | --- | --- |
| Race | White (N=3031) | reference |  |
|  | Black (N=412) | 1.104 (0.965 - 1.264) | 0.148 |
|  | Other (N=81) | 0.812 (0.599 - 1.099) | 0.178 |
|  | Unknown (N=33) | 0.115 (0.029 - 0.461) | 0.002** |
| Age | ≤35 years (N=52) | reference |  |
|  | >35 years and ≤65 years (N=2005) | 0.876 (0.602 - 1.274) | 0.488 |
|  | >65 years (N=1500) | 1.797 (1.234 - 2.616) | 0.002** |
| Gender | Male (N=1198) | reference |  |
|  | Female (N=2359) | 0.673 (0.614 - 0.738) | <0.001*** |
| Site | Rectum (N=3331) | reference |  |
|  | Colon (N=224) | 1.186 (1.0003 - 1.407) | 0.0495* |
|  | OthDigestive (N=2) | 4.262 (1.039 - 17.481) | 0.044* |
| Grade | Grade I (N=226) | reference |  |
|  | Grade II (N=1089) | 1.320 (1.086 - 1.604) | 0.005** |
|  | Grade III (N=1259) | 1.417 (1.166 - 1.722) | <0.001*** |
|  | Grade IV (N=66) | 1.101 (0.744 - 1.630) | 0.6298 |
|  | Unknown (N=917) | 1.282 (1.053 - 1.561) | 0.013* |
| Treatment | none (N=14) | reference |  |
|  | Radiotherapy (N=1522) | 0.466 (0.269 - 0.808) | 0.007** |
|  | Surgery (N=611) | 0.458 (0.265 - 0.794) | 0.005** |
|  | Radiotherapy+Surgery(N=655) | 0.440 (0.252 - 0.767) | 0.004** |
|  | Unknown (N=755) | 0.797 (0.464 - 1.369) | 0.411 |
| Chemotherapy | Yes (N=2077) | reference |  |
|  | No/Unknown (N=1480) | 1.736 (1.535 - 1.963) | <0.001*** |
| Size | ≤ 1cm (N=119) | reference |  |
|  | >1cm and ≤ 2cm (N=274) | 1.098 (0.743 - 1.623) | 0.637 |
|  | >2cm and ≤ 3cm (N=339) | 1.385 (0.953 - 2.012) | 0.087 |
|  | >3cm and ≤ 4cm (N=314) | 1.626 (1.117 - 2.367) | 0.011* |
|  | >4cm and ≤ 5cm (N=273) | 1.455 (0.994 - 2.130) | 0.054 |
|  | >5cm and ≤ 6cm (N=191) | 1.702 (1.148 - 2.523) | 0.008** |
|  | >6cm and ≤ 7cm (N=111) | 1.877 (1.23 - 2.865) | 0.004** |
|  | >7cm and ≤ 8cm (N=116) | 2.110 (1.395 - 3.192) | <0.001*** |
|  | >8cm and ≤ 9cm (N=49) | 2.388 (1.469 - 3.880) | <0.001*** |
|  | >9cm and ≤ 10cm (N=108) | 2.389 (1.598 - 3.573) | <0.001*** |
|  | >10cm and ≤ 11cm (N=47) | 3.066 (1.907 - 4.931) | <0.001*** |
|  | >11 cm and ≤ 12cm (N=1616) | 1.747 (1.239 - 2.463) | 0.002** |
| CEA | Positive (N=260) | reference |  |
|  | Negative (N=445) | 0.863 (0.696 - 1.070) | 0.178 |
|  | Unknown (N=2852) | 0.785 (0.659 - 0.935) | 0.007** |
| Perineural invasion | No (N=567) | reference |  |
|  | Yes (N=37) | 1.595 (0.997 - 2.551) | 0.052 |
|  | Unknown (N=2953) | 1.192 (1.017 - 1.398) | 0.03* |
| T | Tis (N=128) | reference |  |
|  | T1 (N=725) | 1.156 (0.879 - 1.520) | 0.3 |
|  | T2 (N=300) | 1.240 (0.905 - 1.699) | 0.181 |
|  | T3 (N=649) | 1.534 (1.158 - 2.033) | 0.003** |
|  | T4 (N=393) | 2.108 (1.579 - 2.816) | <0.001*** |
|  | Unknown (N=1362) | 1.347 (1.027 - 1.767) | 0.032* |
| N | N0 (N=1704) | reference |  |
|  | N1 (N=435) | 1.054 (0.899 - 1.234) | 0.517 |
|  | N2 (N=99) | 0.847 (0.630 - 1.139) | 0.273 |
|  | N3 (N=14) | 2.048 (1.092 - 3.841) | 0.026* |
|  | N4 (N=1305) | 1.098 (0.966 - 1.247) | 0.153 |
| M | M0 (N=2377) | reference |  |
|  | M1(N=474) | 2.848 (2.493 - 3.255) | <0.001*** |
|  | Unknown (N=706) | 1.001 (0.862 - 1.164) | 0.985 |
| Events: 2000; Global p-value (Log-Rank): 6.2076e-253 | | |  |
| AIC: 28500.94; Concordance Index: 0.75 | |  |  |

*P < 0.05, **P < 0.01, ***P < 0.001

**Supporting Table 2.** Clinical characteristics of patients receiving radiotherapy and radiotherapy combined with surgery pre-PSM and post-PSM

|  | pre-PSM | | | post-PSM | | |
| --- | --- | --- | --- | --- | --- | --- |
|  | Radiotherapy | Radiotherapy+Surgery | P | Radiotherapy | Radiotherapy+Surgery | P |
|  | N=682 | N=341 |  | N=561 | N=299 |  |
| Gender: |  |  | 0.716 |  |  | 0.866 |
| Male | 197(28.9%) | 103(30.2%) |  | 155(27.6%) | 85(28.4%) |  |
| Female | 485(71.1%) | 238(69.8%) |  | 406(72.4%) | 214(71.6%) |  |
| Site: |  |  | 0.003 |  |  | 1 |
| Rectum | 680(99.7%) | 333(97.7%) |  | 560(99.8%) | 298(99.7%) |  |
| Colon | 2(0.29%) | 8(2.35%) |  | 1(0.2%) | 1(0.3%) |  |
| Grade: |  |  | 0.012 |  |  | 0.475 |
| Grade I | 35(5.13%) | 33(9.68%) |  | 30(5.3%) | 19(6.4%) |  |
| Grade II | 273(40.0%) | 144(42.2%) |  | 218(38.9%) | 127(42.5%) |  |
| Grade III | 359(52.6%) | 153(44.9%) |  | 302(53.8%) | 145(48.5%) |  |
| Grade IV | 15(2.20%) | 11(3.23%) |  | 11(2.0%) | 8(2.7%) |  |
| T: |  |  | 0.021 |  |  | 0.62 |
| Tis | 26(3.81%) | 16(4.69%) |  | 20(3.6%) | 13(4.3%) |  |
| T1 | 213(31.2%) | 113(33.1%) |  | 185(33.0%) | 97(32.4%) |  |
| T2 | 132(19.4%) | 43(12.6%) |  | 80(14.3%) | 41(13.7%) |  |
| T3 | 221(32.4%) | 105(30.8%) |  | 195(34.8%) | 94(31.4%) |  |
| T4 | 90(13.2%) | 64(18.8%) |  | 81(14.4%) | 54(18.1%) |  |
| N: |  |  | 0.282 |  |  | 0.703 |
| N0 | 490(71.8%) | 260(76.2%) |  | 421(75.0%) | 227(75.9%) |  |
| N1 | 160(23.5%) | 63(18.5%) |  | 116(20.7%) | 55(18.4%) |  |
| N2 | 29(4.25%) | 17(4.99%) |  | 23(4.1%) | 16(5.4%) |  |
| N3 | 3(0.44%) | 1(0.29%) |  | 1(0.2%) | 1(0.3%) |  |
| M: |  |  | 0.098 |  |  | 0.566 |
| M0 | 625(91.6%) | 323(94.7%) |  | 524(93.4%) | 283(94.6%) |  |
| M1 | 57(8.36%) | 18(5.28%) |  | 37(6.6%) | 16(5.4%) |  |
| Size(cm): |  |  | <0.001 |  |  | 0.42 |
| ≤2 | 76(11.1%) | 64(18.8%) |  | 71(12.7%) | 45(15.1%) |  |
| >2and ≤ 5 | 256(37.5%) | 94(27.6%) |  | 182(32.4%) | 86(28.8%) |  |
| >5 | 350(51.3%) | 183(53.7%) |  | 308(54.9%) | 168(56.2%) |  |
| Age(years): |  |  | 0.005 |  |  | 0.649 |
| ≤35 | 4(0.59%) | 8(2.35%) |  | 2(0.4%) | 1(0.3%) |  |
| >35 and ≤65 | 455(66.7%) | 245(71.8%) |  | 394(70.2%) | 219(73.2%) |  |
| >65 | 223(32.7%) | 88(25.8%) |  | 165(29.4%) | 79(26.4%) |  |

**Supporting Table 3.** Clinical characteristics of patients(undergoing chemotherapy) receiving radiotherapy and radiotherapy combined with surgery pre-PSM and post-PSM

|  | pre-PSM | | | post-PSM | | |
| --- | --- | --- | --- | --- | --- | --- |
|  | Radiotherapy | Radiotherapy+Surgery | P | Radiotherapy | Radiotherapy+Surgery | P |
|  | N=621 | N=303 |  | N=514 | N=259 |  |
| Gender: |  |  | 0.508 |  |  | 0.675 |
| Male | 174(28.0%) | 92(30.4%) |  | 140(27.2%) | 75(29%) |  |
| Female | 447(72.0%) | 211(69.6%) |  | 374(72.8%) | 184(71.0%) |  |
| Site: |  |  | 0.018 |  |  | NA |
| Rectum | 619(99.7%) | 297(98.0%) |  | 514(100.0%) | 259(100.0%) |  |
| Colon | 2(0.32%) | 6(1.98%) |  | 0(0%) | 0(0%) |  |
| Grade: |  |  | 0.013 |  |  | 0.459 |
| Grade I | 31(4.99%) | 29(9.57%) |  | 24(4.7%) | 9(3.5%) |  |
| Grade II | 249(40.1%) | 129(42.6%) |  | 206(40.1%) | 117(45.2%) |  |
| Grade III | 328(52.8%) | 135(44.6%) |  | 275(53.5%) | 127(49.0%) |  |
| Grade IV | 13(2.09%) | 10(3.30%) |  | 9(1.8%) | 6(2.3%) |  |
| T: |  |  | 0.015 |  |  | 0.518 |
| Tis | 22(3.54%) | 15(4.95%) |  | 17(3.3%) | 12(4.6%) |  |
| T1 | 189(30.4%) | 98(32.3%) |  | 164(31.9%) | 86(33.2%) |  |
| T2 | 127(20.5%) | 39(12.9%) |  | 81(15.8%) | 36(13.9%) |  |
| T3 | 200(32.2%) | 93(30.7%) |  | 181(35.2%) | 81(31.3%) |  |
| T4 | 83(13.4%) | 58(19.1%) |  | 71(13.8%) | 44(17.0%) |  |
| N: |  |  | 0.318 |  |  | 0.432 |
| N0 | 440(70.9%) | 227(74.9%) |  | 377(73.3%) | 192(74.1%) |  |
| N1 | 152(24.5%) | 59(19.5%) |  | 118(23.0%) | 53(20.5%) |  |
| N2 | 26(4.19%) | 16(5.28%) |  | 19(3.7%) | 14(5.4%) |  |
| N3 | 3(0.48%) | 1(0.33%) |  |  |  |  |
| M: |  |  | 0.348 |  |  | 0.617 |
| M0 | 572(92.1%) | 285(94.1%) |  | 480(93.4%) | 245(94.6%) |  |
| M1 | 49(7.89%) | 18(5.94%) |  | 34(6.6%) | 14(5.4%) |  |
| Size(cm): |  |  | <0.001 |  |  | 0.093 |
| ≤2 | 72(11.6%) | 57(18.8%) |  | 60(11.7%) | 42(16.2%) |  |
| >2and ≤ 5 | 240(38.6% | )81(26.7%) |  | 174(33.9%) | 72(27.8%) |  |
| >5 | 309(49.8%) | 165(54.5%) |  | 280(54.5%) | 145(56.0%) |  |
| Age(years): |  |  | 0.001 |  |  | 0.755 |
| ≤35 | 4(0.64%) | 8(2.64%) |  | 2(0.4%) | 2(0.8%) |  |
| >35 and ≤65 | 423(68.1%) | 227(74.9%) |  | 382(74.3%) | 194(74.9%) |  |
| >65 | 194(31.2%) | 68(22.4%) |  | 130(25.3%) | 63(24.3%) |  |

**Supporting Table 4.** Clinical characteristics of patients receiving radiotherapy and surgery pre-PSM and post-PSM

|  | pre-PSM | | | post-PSM | | |
| --- | --- | --- | --- | --- | --- | --- |
|  | Radiotherapy | Surgery | P | Radiotherapy | Surgery | P |
|  | N=682 | N=266 |  | 416 | 180 |  |
| Gender: |  |  | 0.001 |  |  | 0.594 |
| Male | 197(28.9%) | 107(40.2%) |  | 146(35.1%) | 68(37.8%) |  |
| Female | 485(71.1%) | 159(59.8%) |  | 270 (64.9%) | 112 (62.2%) |  |
| Site: |  |  | <0.001 |  |  | 0.75 |
| Rectum | 680(99.7%) | 204(76.7%) |  | 414(99.5%) | 178(98.9%) |  |
| Colon | 2(0.29%) | 62(23.3%) |  | 2 ( 0.5%) | 2 ( 1.1%) |  |
| Grade: |  |  | <0.001 |  |  | 0.286 |
| GradeI | 35(5.13%) | 41(15.4%) |  | 31 ( 7.5%) | 22 (12.2%) |  |
| GradeII | 273(40.0%) | 100(37.6%) |  | 176 (42.3%) | 76 (42.2%) |  |
| GradeIII | 359(52.6%) | 114(42.9%) |  | 201 (48.3%) | 79 (43.9%) |  |
| GradeIV | 15(2.20%) | 11(4.14%) |  | 8 ( 1.9%) | 3 ( 1.7%) |  |
| T: |  |  | <0.001 |  |  | 0.938 |
| Tis | 26(3.81%) | 23(8.65%) |  | 21 ( 5.0%) | 10 ( 5.6%) |  |
| T1 | 213(31.2%) | 78(29.3%) |  | 134 (32.2%) | 64 (35.6%) |  |
| T2 | 132(19.4%) | 22(8.27%) |  | 48 (11.5%) | 19 (10.6%) |  |
| T3 | 221(32.4%) | 97(36.5%) |  | 150 (36.1%) | 61 (33.9%) |  |
| T4 | 90(13.2%) | 46(17.3%) |  | 63 (15.1%) | 26 (14.4%) |  |
| N: |  |  | 0.01 |  |  | 0.611 |
| N0 | 490(71.8%) | 208(78.2%) |  | 324 (77.9%) | 146 (81.1%) |  |
| N1 | 160(23.5%) | 39(14.7%) |  | 72 (17.3%) | 24 (13.3%) |  |
| N2 | 29(4.25%) | 17(6.39%) |  | 19 ( 4.6%) | 9 ( 5.0%) |  |
| N3 | 3(0.44%) | 2(0.75%) |  | 1 ( 0.2%) | 1 ( 0.6%) |  |
| M: |  |  | 0.919 |  |  | 0.856 |
| M0 | 625(91.6%) | 245(92.1%) |  | 392(94.2%) | 171(95.0%) |  |
| M1 | 57(8.36%) | 21(7.89%) |  | 24 ( 5.8%) | 9 ( 5.0%) |  |
| Size(cm): |  |  | 0.003 |  |  | 0.392 |
| ≤2 | 76(11.1%) | 52(19.5%) |  | 58 (13.9%) | 33 (18.3%) |  |
| >2and ≤ 5 | 256(37.5%) | 92(34.6%) |  | 148 (35.6%) | 61 (33.9%) |  |
| >5 | 350(51.3%) | 122(45.9%) |  | 210 (50.5%) | 86 (47.8%) |  |
| Age(years): |  |  | <0.001 |  |  | 0.554 |
| ≤35 | 4(0.59%) | 3(1.13%) |  | 2 (0.5%) | 1 (0.6%) |  |
| >35 and ≤65 | 455(66.7%) | 123(46.2%) |  | 235 (56.5%) | 93 (51.7%) |  |
| >65 | 223(32.7%) | 140(52.6%) |  | 179 (43.0%) | 86 (47.8%) |  |

**Supporting Table 5.** Clinical characteristics of patients(undergoing chemotherapy) receiving radiotherapy and surgery pre-PSM and post-PSM

|  | pre-PSM | | | post-PSM | | |
| --- | --- | --- | --- | --- | --- | --- |
|  | Radiotherapy | Surgery | P | Radiotherapy | Surgery | P |
|  | N=621 | N=42 |  | N=57 | N=21 |  |
| Gender: |  |  | 0.222 |  |  | 0.81 |
| Male | 174 (28.0%) | 16 (38.1%) |  | 17(29.8%) | 5(23.8%) |  |
| Female | 447 (72.0%) | 26 (61.9%) |  | 40(70.2%) | 16(76.2%) |  |
| Site: |  |  | <0.001 |  |  | NA |
| Rectum | 619 (99.7%) | 25 (59.5%) |  | 57(100.0%) | 21(100.0%) |  |
| Colon | 2 (0.32%) | 17 (40.5%) |  | 0(0%) | 0(0%) |  |
| Grade: |  |  | 0.009 |  |  | 0.442 |
| GradeI | 31 (4.99%) | 3 (7.14%) |  | 1(1.8%) | 2(9.5%) |  |
| GradeII | 249 (40.1%) | 8 (19.0%) |  | 17(29.8%) | 5(23.8%) |  |
| GradeIII | 328 (52.8%) | 28 (66.7%) |  | 37(64.9%) | 13(61.9%) |  |
| GradeIV | 13 (2.09%) | 3 (7.14%) |  | 2(3.5%) | 1(4.8%) |  |
| T: |  |  | <0.001 |  |  | 0.935 |
| Tis | 22 (3.54%) | 4 (9.52%) |  | 6(10.5%) | 1(4.8%) |  |
| T1 | 189 (30.4%) | 4 (9.52%) |  | 12(21.1%) | 4(19.0%) |  |
| T2 | 127 (20.5%) | 2 (4.76%) |  | 5(8.8%) | 2(9.5%) |  |
| T3 | 200 (32.2%) | 20 (47.6%) |  | 21(36.8%) | 8(38.1%) |  |
| T4 | 83 (13.4%) | 12 (28.6%) |  | 13(22.8%) | 6(28.6%) |  |
| N: |  |  | <0.001 |  |  | 0.765 |
| N0 | 440 (70.9%) | 18 (42.9%) |  | 29(50.9%) | 12(57.2%) |  |
| N1 | 152 (24.5%) | 15 (35.7%) |  | 24(42.1%) | 7(33.3%) |  |
| N2 | 26 (4.19%) | 8 (19.0%) |  | 4(7.0%) | 2(9.5%) |  |
| N3 | 3 (0.48%) | 1 (2.38%) |  | 0(0%) | 0(0%) |  |
| M: |  |  | 0.002 |  |  | 0.537 |
| M0 | 572 (92.1%) | 32 (76.2%) |  | 51(89.5%) | 17(81.0%) |  |
| M1 | 49 (7.89%) | 10 (23.8%) |  | 6(10.5%) | 4(19.0%) |  |
| Size(cm): |  |  | 0.808 |  |  | 0.798 |
| ≤2 | 72 (11.6%) | 6 (14.3%) |  | 7(12.3%) | 3(14.3%) |  |
| >2and ≤ 5 | 240 (38.6%) | 15 (35.7%) |  | 18(31.6%) | 8(38.1%) |  |
| >5 | 309 (49.8%) | 21 (50.0%) |  | 32(56.1%) | 10(47.6%) |  |
| Age(years): |  |  | 0.698 |  |  | 0.863 |
| ≤35 | 4 (0.64%) | 0 (0.00%) |  | 0(0%) | 0(0%) |  |
| >35 and ≤65 | 423 (68.1%) | 27 (64.3%) |  | 41(71.9%) | 14(66.7%) |  |
| >65 | 194 (31.2%) | 15 (35.7%) |  | 16(28.1%) | 7(33.3%) |  |

**Supporting Table 6.** The 1-year survival rate and median survival time of advanced patients. The Na values represented inestimable

| Metastatic site | Number of patients | 1-year survival rate | Median survival time(months) |
| --- | --- | --- | --- |
| liver | 94 | 33.4% | 9 |
| lung | 20 | 38.5% | 7 |
| bone | 10 | 35% | 8 |
| Liver + lung | 28 | 26.8% | 7 |
| Liver + bone | 8 | 12.5% | 5 |
| Lung + bone | 3 | 33.3% | 4 |
| Liver + lung + bone | 7 | 28.6% | 4 |
| distant lymph nodes | 6 | 60% | Na |
| distant lymph nodes +  liver | 5 | 33.3% | 6 |
| distant lymph nodes +  Liver + bone | 1 | 0% | Na |
| distant lymph nodes +  liver + lung + bone | 2 | 0% | Na |

**Supporting Table 7.** The survival analysis of metastasis CSCC

| Metastatic site | Number of patients | HR(95CI%) | P |
| --- | --- | --- | --- |
| Liver | 94 | reference |  |
| lung | 20 | 0.903(0.524-1.56) | 0.713 |
| bone | 10 | 1.117(0.537-2.32) | 0.768 |
| Liver + lung | 28 | 1.278(0.814-2.00) | 0.286 |
| Liver + bone | 8 | 1.374(0.630-3.00) | 0.424 |
| Liver + lung + bone | 7 | 1.324(0.606-2.89) | 0.482 |
| distant lymph nodes | 6 | 0.624(0.153-2.56) | 0.513 |
| distant lymph nodes +  liver | 6 | 0.967(0.235-3.98) | 0.963 |

**Supporting Table 8.** The predict potential therapeutic agents for CSCC by CMAP database

| rank | batch | cmap name | dose | cell | score | up | down | instance_id |
| --- | --- | --- | --- | --- | --- | --- | --- | --- |
| 1 | 634 | iohexol | 5 µM | HL60 | 1 | 0.152 | -0.12 | 2461 |
| 2 | 618 | nalidixic acid | 15 µM | HL60 | 0.981 | 0.116 | -0.15 | 2336 |
| 3 | 614 | tranexamic acid | 25 µM | HL60 | 0.973 | 0.1 | -0.164 | 1401 |
| 4 | 602 | genistein | 10 µM | HL60 | 0.967 | 0.081 | -0.182 | 1176 |
| 5 | 631 | myricetin | 13 µM | HL60 | 0.963 | 0.096 | -0.166 | 1334 |
| 6 | 644 | trimethylcolchicinic acid | 12 µM | HL60 | 0.962 | 0.111 | -0.15 | 2146 |
| 7 | 631 | naringenin | 15 µM | HL60 | 0.958 | 0.104 | -0.156 | 1342 |
| 8 | 631 | Chicago Sky Blue 6B | 4 µM | HL60 | 0.958 | 0.069 | -0.191 | 1330 |
| 9 | 629 | heptaminol | 22 µM | HL60 | 0.953 | 0.088 | -0.171 | 1866 |
| 10 | 756 | ribavirin | 16 µM | MCF7 | 0.952 | 0.175 | -0.084 | 6521 |
| 11 | 631 | digitoxigenin | 11 µM | HL60 | 0.939 | 0.08 | -0.176 | 1339 |
| 12 | 630 | ritodrine | 12 µM | HL60 | 0.92 | 0.088 | -0.162 | 1280 |
| 13 | 649 | atractyloside | 5 µM | HL60 | 0.908 | 0.056 | -0.191 | 2573 |
| 14 | 618 | mebendazole | 14 µM | HL60 | 0.908 | 0.149 | -0.098 | 2338 |
| 15 | 24 | exisulind | 50 µM | MCF7 | 0.906 | 0.088 | -0.158 | 314 |
| 16 | 649 | thiamazole | 35 µM | HL60 | 0.904 | 0.08 | -0.166 | 2570 |
| 17 | 704 | etilefrine | 18 µM | PC3 | 0.896 | 0.115 | -0.129 | 4590 |
| 18 | 682 | ethoxyquin | 18 µM | PC3 | 0.893 | 0.105 | -0.137 | 3764 |
| 19 | 610 | idoxuridine | 11 µM | PC3 | 0.89 | 0.1 | -0.142 | 1899 |
| 20 | 631 | N-acetyl-L-aspartic acid | 23 µM | HL60 | 0.887 | 0.105 | -0.136 | 1329 |
| 21 | 644 | cinchonine | 14 µM | HL60 | 0.882 | 0.127 | -0.113 | 2133 |
| 22 | 619 | lincomycin | 9 µM | HL60 | 0.875 | 0.062 | -0.176 | 2380 |
| 23 | 644 | boldine | 12 µM | HL60 | 0.874 | 0.065 | -0.173 | 2148 |
| 24 | 613 | haloperidol | 11 µM | HL60 | 0.873 | 0.111 | -0.127 | 2039 |
| 25 | 650 | estradiol | 100 nM | HL60 | 0.872 | 0.167 | -0.07 | 2668 |
| 26 | 631 | carteolol | 12 µM | HL60 | 0.872 | 0.092 | -0.145 | 1340 |
| 27 | 746 | methanthelinium bromide | 10 µM | MCF7 | 0.87 | 0.1 | -0.137 | 6254 |
| 28 | 649 | bendroflumethiazide | 9 µM | HL60 | 0.865 | 0.082 | -0.153 | 2555 |
| 29 | 757 | estradiol | 100 nM | MCF7 | 0.862 | 0.073 | -0.161 | 5568 |
| 30 | 749 | oxedrine | 24 µM | HL60 | 0.859 | 0.1 | -0.134 | 6156 |
| 31 | 616 | disulfiram | 13 µM | PC3 | 0.858 | 0.102 | -0.131 | 2053 |
| 32 | 69 | monorden | 100 nM | SKMEL5 | 0.857 | 0.077 | -0.156 | 493 |
| 33 | 631 | levothyroxine sodium | 5 µM | HL60 | 0.856 | 0.077 | -0.155 | 1312 |
| 34 | 728 | furosemide | 12 µM | PC3 | 0.855 | 0.098 | -0.135 | 4503 |
| 35 | 618 | ofloxacin | 11 µM | HL60 | 0.855 | 0.12 | -0.113 | 2340 |
| 36 | 644 | rescinnamine | 6 µM | HL60 | 0.854 | 0.079 | -0.153 | 2130 |
| 37 | 651 | corbadrine | 22 µM | HL60 | 0.849 | 0.067 | -0.164 | 2710 |
| 38 | 644 | vitexin | 9 µM | HL60 | 0.847 | 0.077 | -0.154 | 2155 |
| 39 | 687 | levcycloserine | 39 µM | MCF7 | 0.843 | 0.105 | -0.124 | 3870 |
| 40 | 650 | LY-294002 | 100 nM | HL60 | 0.842 | 0.081 | -0.148 | 2676 |
| 41 | 612 | pheniramine | 11 µM | HL60 | 0.842 | 0.108 | -0.121 | 1992 |
| 42 | 644 | canadine | 12 µM | HL60 | 0.841 | 0.062 | -0.167 | 2163 |
| 43 | 747 | suxibuzone | 9 µM | MCF7 | 0.836 | 0.13 | -0.097 | 7163 |
| 44 | 612 | cimetidine | 16 µM | HL60 | 0.833 | 0.102 | -0.124 | 1964 |
| 45 | 702 | piperacillin | 7 µM | PC3 | 0.833 | 0.099 | -0.127 | 4320 |
| 46 | 618 | orphenadrine | 13 µM | HL60 | 0.832 | 0.105 | -0.121 | 2356 |
| 47 | 618 | riluzole | 15 µM | HL60 | 0.832 | 0.101 | -0.125 | 2334 |
| 48 | 630 | clomifene | 7 µM | HL60 | 0.825 | 0.074 | -0.151 | 1269 |
| 49 | 715 | adenosine phosphate | 11 µM | PC3 | 0.825 | 0.126 | -0.098 | 6760 |
| 50 | 693 | orciprenaline | 8 µM | PC3 | 0.822 | 0.138 | -0.086 | 4248 |
| 51 | 612 | prednisone | 11 µM | HL60 | 0.818 | 0.099 | -0.124 | 1978 |
| 52 | 650 | estradiol | 10 nM | HL60 | 0.817 | 0.075 | -0.147 | 2701 |
| 53 | 613 | guanabenz | 14 µM | HL60 | 0.817 | 0.102 | -0.12 | 2045 |
| 54 | 728 | cefalotin | 10 µM | PC3 | 0.816 | 0.069 | -0.153 | 4482 |
| 55 | 649 | trichostatin A | 100 nM | HL60 | 0.815 | 0.081 | -0.14 | 2566 |
| 56 | 644 | Prestwick-682 | 6 µM | HL60 | 0.81 | 0.055 | -0.165 | 2164 |
| 57 | 730 | benfluorex | 10 µM | MCF7 | 0.807 | 0.097 | -0.123 | 5327 |
| 58 | 682 | mexiletine | 19 µM | PC3 | 0.807 | 0.112 | -0.108 | 3781 |
| 59 | 614 | nocodazole | 13 µM | HL60 | 0.806 | 0.082 | -0.137 | 1393 |
| 60 | 75 | monorden | 100 nM | HL60 | 0.804 | 0.075 | -0.144 | 544 |
| 61 | 648 | cefmetazole | 8 µM | HL60 | 0.804 | 0.049 | -0.17 | 2524 |
| 62 | 614 | disulfiram | 13 µM | HL60 | 0.804 | 0.14 | -0.079 | 1369 |
| 63 | 678 | trifluridine | 14 µM | MCF7 | 0.803 | 0.152 | -0.066 | 3559 |
| 64 | 719 | bethanechol | 20 µM | PC3 | 0.803 | 0.108 | -0.11 | 5114 |
| 65 | 719 | pyrantel | 11 µM | PC3 | 0.802 | 0.11 | -0.108 | 5088 |
| 66 | 622 | furosemide | 12 µM | HL60 | 0.802 | 0.083 | -0.135 | 1580 |
| 67 | 644 | sulfabenzamide | 14 µM | HL60 | 0.801 | 0.094 | -0.124 | 2159 |
| 68 | 629 | moxisylyte | 13 µM | HL60 | 0.8 | 0.097 | -0.121 | 1846 |
| 69 | 622 | amodiaquine | 9 µM | HL60 | 0.797 | 0.103 | -0.114 | 1570 |
| 70 | 645 | betonicine | 25 µM | HL60 | 0.797 | 0.076 | -0.14 | 2207 |
| 71 | 719 | sulindac | 11 µM | PC3 | 0.789 | 0.098 | -0.117 | 5103 |
| 72 | 719 | carbamazepine | 17 µM | PC3 | 0.787 | 0.143 | -0.071 | 5093 |
| 73 | 662 | berberine | 11 µM | MCF7 | 0.787 | 0.086 | -0.128 | 2770 |
| 74 | 618 | phenelzine | 17 µM | HL60 | 0.786 | 0.124 | -0.09 | 2357 |
| 75 | 618 | pentolonium | 7 µM | HL60 | 0.786 | 0.101 | -0.112 | 2343 |
| 76 | 614 | acetylsalicylsalicylic acid | 13 µM | HL60 | 0.785 | 0.112 | -0.101 | 1377 |
| 77 | 718 | metformin | 24 µM | PC3 | 0.783 | 0.086 | -0.127 | 5068 |
| 78 | 752 | sulfamethizole | 15 µM | MCF7 | 0.781 | 0.09 | -0.122 | 6099 |
| 79 | 610 | pyrimethamine | 16 µM | PC3 | 0.781 | 0.142 | -0.07 | 1894 |
| 80 | 614 | nomifensine | 11 µM | HL60 | 0.778 | 0.103 | -0.109 | 1378 |
| 81 | 614 | chlorphenamine | 10 µM | HL60 | 0.774 | 0.084 | -0.127 | 1371 |
| 82 | 677 | pivampicillin | 9 µM | MCF7 | 0.773 | 0.126 | -0.085 | 3506 |
| 83 | 610 | hydrochlorothiazide | 13 µM | PC3 | 0.771 | 0.166 | -0.044 | 1906 |
| 84 | 612 | metronidazole | 23 µM | HL60 | 0.77 | 0.111 | -0.098 | 2003 |
| 85 | 660 | oxybenzone | 18 µM | HL60 | 0.77 | 0.064 | -0.146 | 3092 |
| 86 | 622 | mepacrine | 8 µM | HL60 | 0.77 | 0.104 | -0.106 | 1563 |
| 87 | 704 | vancomycin | 3 µM | PC3 | 0.769 | 0.075 | -0.134 | 4598 |
| 88 | 766 | isoxicam | 12 µM | MCF7 | 0.768 | 0.119 | -0.09 | 7028 |
| 89 | 1059 | PF-00562151-00 | 10 µM | MCF7 | 0.766 | 0.134 | -0.074 | 6912 |
| 90 | 665 | netilmicin | 3 µM | HL60 | 0.766 | 0.103 | -0.105 | 2963 |
| 91 | 731 | cefepime | 7 µM | PC3 | 0.763 | 0.055 | -0.152 | 5761 |
| 92 | 640 | harmol | 16 µM | HL60 | 0.762 | 0.08 | -0.127 | 1750 |
| 93 | 741 | netilmicin | 3 µM | MCF7 | 0.761 | 0.104 | -0.103 | 6005 |
| 94 | 682 | ethisterone | 13 µM | PC3 | 0.76 | 0.111 | -0.096 | 3783 |
| 95 | 22a | fludrocortisone | 1 µM | MCF7 | 0.76 | 0.063 | -0.143 | 282 |
| 96 | 644 | glimepiride | 8 µM | HL60 | 0.759 | 0.116 | -0.09 | 2154 |
| 97 | 644 | benzocaine | 24 µM | HL60 | 0.759 | 0.071 | -0.136 | 2167 |
| 98 | 648 | butamben | 21 µM | HL60 | 0.757 | 0.085 | -0.12 | 2531 |
| 99 | 632 | amprolium | 13 µM | MCF7 | 0.757 | 0.113 | -0.093 | 1479 |
| 100 | 772 | bacitracin | 3 µM | MCF7 | 0.755 | 0.082 | -0.123 | 7448 |
| 101 | 651 | hexylcaine | 13 µM | HL60 | 0.753 | 0.08 | -0.124 | 2708 |
| 102 | 610 | captopril | 17 µM | PC3 | 0.753 | 0.109 | -0.096 | 1907 |
| 103 | 629 | betazole | 27 µM | HL60 | 0.753 | 0.11 | -0.095 | 1854 |
| 104 | 733 | ketotifen | 9 µM | PC3 | 0.753 | 0.072 | -0.133 | 5842 |
| 105 | 657 | etamivan | 18 µM | MCF7 | 0.753 | 0.122 | -0.083 | 2879 |
| 106 | 602 | fulvestrant | 10 nM | HL60 | 0.752 | 0.046 | -0.158 | 1179 |
| 107 | 729 | dexibuprofen | 19 µM | MCF7 | 0.751 | 0.108 | -0.096 | 5311 |
| 108 | 645 | trichostatin A | 100 nM | HL60 | 0.75 | 0.103 | -0.101 | 2208 |
| 109 | 632 | niclosamide | 12 µM | MCF7 | 0.75 | 0.068 | -0.136 | 1498 |
| 110 | 642 | nefopam | 14 µM | MCF7 | 0.746 | 0.084 | -0.119 | 2317 |
| 111 | 660 | prasterone | 12 µM | HL60 | 0.746 | 0.077 | -0.126 | 3097 |
| 112 | 725 | geldanamycin | 1 µM | MCF7 | 0.745 | 0.067 | -0.136 | 5225 |
| 113 | 707 | (+)-isoprenaline | 11 µM | MCF7 | 0.743 | 0.115 | -0.087 | 5009 |
| 114 | 660 | florfenicol | 11 µM | HL60 | 0.743 | 0.09 | -0.112 | 3083 |
| 115 | 641 | hydroquinine | 9 µM | HL60 | 0.742 | 0.067 | -0.134 | 1775 |
| 116 | 691 | Prestwick-691 | 14 µM | MCF7 | 0.742 | 0.073 | -0.129 | 4172 |
| 117 | 631 | N-acetylmuramic acid | 14 µM | HL60 | 0.742 | 0.065 | -0.137 | 1326 |
| 118 | 612 | sulfaguanidine | 19 µM | HL60 | 0.74 | 0.101 | -0.1 | 1995 |
| 119 | 688 | nafcillin | 9 µM | PC3 | 0.74 | 0.103 | -0.098 | 3983 |
| 120 | 645 | sulfamerazine | 15 µM | HL60 | 0.739 | 0.095 | -0.106 | 2181 |
| 121 | 1069 | MG-262 | 100 nM | PC3 | 0.739 | 0.083 | -0.118 | 7068 |
| 122 | 629 | sulfadiazine | 16 µM | HL60 | 0.737 | 0.09 | -0.11 | 1852 |
| 123 | 754 | Prestwick-1083 | 9 µM | PC3 | 0.736 | 0.058 | -0.142 | 6357 |
| 124 | 761 | articaine | 12 µM | PC3 | 0.735 | 0.087 | -0.113 | 7272 |
| 125 | 618 | griseofulvin | 11 µM | HL60 | 0.734 | 0.103 | -0.097 | 2332 |
| 126 | 745 | ciprofibrate | 14 µM | MCF7 | 0.734 | 0.144 | -0.055 | 6218 |
| 127 | 660 | altretamine | 19 µM | HL60 | 0.733 | 0.056 | -0.144 | 3090 |
| 128 | 662 | demecarium bromide | 6 µM | MCF7 | 0.733 | 0.076 | -0.123 | 2773 |
| 129 | 613 | erythromycin | 5 µM | HL60 | 0.733 | 0.106 | -0.093 | 2010 |
| 130 | 713 | methyldopa | 19 µM | PC3 | 0.732 | 0.076 | -0.123 | 4677 |
| 131 | 725 | haloperidol | 10 µM | MCF7 | 0.731 | 0.064 | -0.134 | 5241 |
| 132 | 612 | trimethobenzamide | 9 µM | HL60 | 0.729 | 0.076 | -0.122 | 2002 |
| 133 | 681 | ketoprofen | 16 µM | PC3 | 0.728 | 0.083 | -0.115 | 3729 |
| 134 | 632 | tolnaftate | 13 µM | MCF7 | 0.727 | 0.067 | -0.131 | 1501 |
| 135 | 735 | vigabatrin | 31 µM | MCF7 | 0.727 | 0.088 | -0.11 | 5415 |
| 136 | 655 | puromycin | 7 µM | MCF7 | 0.726 | 0.063 | -0.135 | 3310 |
| 137 | 764 | alprenolol | 14 µM | PC3 | 0.726 | 0.1 | -0.098 | 7141 |
| 138 | 663 | cinchonine | 14 µM | MCF7 | 0.725 | 0.071 | -0.126 | 2789 |
| 139 | 59 | arachidonic acid | 10 µM | MCF7 | 0.724 | 0.098 | -0.099 | 441 |
| 140 | 745 | (+)-chelidonine | 11 µM | MCF7 | 0.724 | 0.109 | -0.088 | 6236 |
| 141 | 612 | oxybuprocaine | 12 µM | HL60 | 0.722 | 0.099 | -0.097 | 1976 |
| 142 | 628 | acetazolamide | 18 µM | PC3 | 0.721 | 0.08 | -0.116 | 1808 |
| 143 | 629 | sulindac | 11 µM | HL60 | 0.72 | 0.066 | -0.129 | 1857 |
| 144 | 629 | amitriptyline | 13 µM | HL60 | 0.72 | 0.047 | -0.149 | 1865 |
| 145 | 635 | phenacetin | 22 µM | HL60 | 0.719 | 0.09 | -0.105 | 2471 |
| 146 | 505 | celastrol | 3 µM | MCF7 | 0.719 | 0.128 | -0.067 | 887 |
| 147 | 635 | sisomicin | 3 µM | HL60 | 0.718 | 0.067 | -0.129 | 2493 |
| 148 | 1021 | 0225151-0000 | 10 µM | PC3 | 0.718 | 0.09 | -0.105 | 6389 |
| 149 | 687 | chlorcyclizine | 12 µM | MCF7 | 0.718 | 0.122 | -0.073 | 3893 |
| 150 | 699 | PNU-0251126 | 1 µM | MCF7 | 0.717 | 0.057 | -0.138 | 4714 |
| 151 | 682 | sulfadimidine | 13 µM | PC3 | 0.716 | 0.076 | -0.119 | 3765 |
| 152 | 619 | disopyramide | 12 µM | HL60 | 0.715 | 0.078 | -0.116 | 2408 |
| 153 | 656 | nifuroxazide | 15 µM | MCF7 | 0.714 | 0.065 | -0.129 | 2850 |
| 154 | 613 | dehydrocholic acid | 10 µM | HL60 | 0.713 | 0.078 | -0.116 | 2023 |
| 155 | 730 | idazoxan | 17 µM | MCF7 | 0.713 | 0.092 | -0.102 | 5347 |
| 156 | 660 | pralidoxime | 23 µM | HL60 | 0.713 | 0.06 | -0.134 | 3066 |
| 157 | 613 | aciclovir | 18 µM | HL60 | 0.713 | 0.052 | -0.142 | 2044 |
| 158 | 641 | physostigmine | 6 µM | HL60 | 0.713 | 0.06 | -0.133 | 1776 |
| 159 | 755 | prasterone | 12 µM | MCF7 | 0.713 | 0.08 | -0.114 | 6474 |
| 160 | 630 | coralyne | 10 µM | HL60 | 0.712 | 0.066 | -0.127 | 1298 |
| 161 | 619 | isotretinoin | 13 µM | HL60 | 0.711 | 0.077 | -0.116 | 2407 |
| 162 | 631 | piperine | 14 µM | HL60 | 0.711 | 0.072 | -0.121 | 1327 |
| 163 | 733 | cefotaxime | 8 µM | PC3 | 0.711 | 0.101 | -0.092 | 5830 |
| 164 | 649 | spiramycin | 5 µM | HL60 | 0.71 | 0.066 | -0.127 | 2558 |
| 165 | 704 | diphemanil metilsulfate | 10 µM | PC3 | 0.71 | 0.083 | -0.11 | 4591 |
| 166 | 111 | genistein | 10 µM | MCF7 | 0.71 | 0.069 | -0.124 | 638 |
| 167 | 691 | gelsemine | 12 µM | MCF7 | 0.708 | 0.059 | -0.134 | 4177 |
| 168 | 736 | sulfasalazine | 10 µM | MCF7 | 0.707 | 0.119 | -0.073 | 5446 |
| 169 | 649 | cefazolin | 8 µM | HL60 | 0.707 | 0.055 | -0.137 | 2564 |
| 170 | 644 | Prestwick-692 | 7 µM | HL60 | 0.706 | 0.043 | -0.149 | 2165 |
| 171 | 661 | octopamine | 21 µM | HL60 | 0.705 | 0.072 | -0.12 | 3112 |
| 172 | 685 | phenformin | 17 µM | MCF7 | 0.705 | 0.076 | -0.115 | 3622 |
| 173 | 744 | kinetin | 19 µM | MCF7 | 0.705 | 0.063 | -0.129 | 6813 |
| 174 | 733 | kaempferol | 14 µM | PC3 | 0.704 | 0.07 | -0.122 | 5839 |
| 175 | 671 | cromoglicic acid | 8 µM | MCF7 | 0.704 | 0.084 | -0.108 | 3475 |
| 176 | 654 | calcium pantothenate | 8 µM | MCF7 | 0.704 | 0.094 | -0.098 | 3248 |
| 177 | 671 | ifosfamide | 15 µM | MCF7 | 0.703 | 0.09 | -0.101 | 3485 |
| 178 | 623 | enoxacin | 12 µM | HL60 | 0.703 | 0.075 | -0.117 | 1597 |
| 179 | 73 | SC-58125 | 10 µM | SKMEL5 | 0.702 | 0.097 | -0.094 | 507 |
| 180 | 612 | tolazoline | 20 µM | HL60 | 0.702 | 0.088 | -0.103 | 2000 |
| 181 | 637 | piroxicam | 12 µM | MCF7 | 0.702 | 0.089 | -0.102 | 2252 |
| 182 | 616 | midodrine | 14 µM | PC3 | 0.701 | 0.068 | -0.122 | 2087 |
| 183 | 622 | tretinoin | 13 µM | HL60 | 0.699 | 0.09 | -0.1 | 1548 |
| 184 | 743 | Prestwick-1084 | 16 µM | MCF7 | 0.699 | 0.1 | -0.09 | 6767 |
| 185 | 731 | trioxysalen | 18 µM | PC3 | 0.698 | 0.069 | -0.12 | 5736 |
| 186 | 513 | haloperidol | 10 µM | MCF7 | 0.698 | 0.083 | -0.107 | 1082 |
| 187 | 701 | etiocholanolone | 14 µM | PC3 | 0.698 | 0.088 | -0.102 | 4298 |
| 188 | 665 | Prestwick-1103 | 20 µM | HL60 | 0.697 | 0.051 | -0.138 | 2978 |
| 189 | 615 | ranitidine | 11 µM | HL60 | 0.697 | 0.103 | -0.086 | 1404 |
| 190 | 648 | terazosin | 9 µM | HL60 | 0.697 | 0.078 | -0.112 | 2530 |
| 191 | 618 | trimethoprim | 14 µM | HL60 | 0.697 | 0.092 | -0.097 | 2345 |
| 192 | 1061 | SB-203580 | 1 µM | MCF7 | 0.696 | 0.108 | -0.081 | 6915 |
| 193 | 632 | phenazone | 21 µM | MCF7 | 0.696 | 0.075 | -0.115 | 1489 |
| 194 | 687 | orphenadrine | 13 µM | MCF7 | 0.696 | 0.078 | -0.111 | 3883 |
| 195 | 738 | isoflupredone | 10 µM | MCF7 | 0.696 | 0.053 | -0.136 | 5545 |
| 196 | 764 | vinburnine | 14 µM | PC3 | 0.696 | 0.065 | -0.124 | 7154 |
| 197 | 626 | haloperidol | 10 µM | MCF7 | 0.696 | 0.104 | -0.085 | 1628 |
| 198 | 631 | lactobionic acid | 11 µM | HL60 | 0.695 | 0.072 | -0.117 | 1309 |
| 199 | 622 | praziquantel | 13 µM | HL60 | 0.695 | 0.068 | -0.121 | 1572 |
| 200 | 629 | mefenamic acid | 17 µM | HL60 | 0.694 | 0.06 | -0.129 | 1863 |
| 201 | 653 | estradiol | 15 µM | MCF7 | 0.694 | 0.063 | -0.125 | 2653 |
| 202 | 671 | alimemazine | 5 µM | MCF7 | 0.693 | 0.1 | -0.088 | 3478 |
| 203 | 619 | bumetanide | 11 µM | HL60 | 0.692 | 0.066 | -0.122 | 2409 |
| 204 | 744 | furosemide | 12 µM | MCF7 | 0.692 | 0.08 | -0.108 | 6841 |
| 205 | 644 | gelsemine | 12 µM | HL60 | 0.691 | 0.065 | -0.123 | 2162 |
| 206 | 711 | dicoumarol | 12 µM | MCF7 | 0.691 | 0.08 | -0.108 | 3941 |
| 207 | 749 | propoxycaine | 12 µM | HL60 | 0.691 | 0.087 | -0.101 | 6161 |
| 208 | 745 | calycanthine | 12 µM | MCF7 | 0.69 | 0.088 | -0.1 | 6221 |
| 209 | 654 | N-acetylmuramic acid | 14 µM | MCF7 | 0.69 | 0.097 | -0.091 | 3262 |
| 210 | 715 | zaprinast | 15 µM | PC3 | 0.689 | 0.122 | -0.066 | 6749 |
| 211 | 728 | dacarbazine | 22 µM | PC3 | 0.687 | 0.059 | -0.128 | 4480 |
| 212 | 706 | fursultiamine | 9 µM | MCF7 | 0.686 | 0.099 | -0.087 | 4975 |
| 213 | 614 | thioproperazine | 6 µM | HL60 | 0.685 | 0.062 | -0.125 | 1390 |
| 214 | 654 | lactobionic acid | 11 µM | MCF7 | 0.684 | 0.075 | -0.111 | 3246 |
| 215 | 729 | monobenzone | 20 µM | MCF7 | 0.683 | 0.111 | -0.075 | 5312 |
| 216 | 650 | rosiglitazone | 10 µM | HL60 | 0.683 | 0.075 | -0.111 | 2693 |
| 217 | 725 | acetylsalicylic acid | 100 µM | MCF7 | 0.683 | 0.121 | -0.065 | 5201 |
| 218 | 1 | metformin | 10 µM | MCF7 | 0.682 | 0.072 | -0.114 | 1 |
| 219 | 735 | amoxicillin | 11 µM | MCF7 | 0.681 | 0.109 | -0.077 | 5385 |
| 220 | 642 | metoclopramide | 12 µM | MCF7 | 0.681 | 0.076 | -0.109 | 2315 |
| 221 | 649 | isocarboxazid | 17 µM | HL60 | 0.68 | 0.067 | -0.118 | 2562 |
| 222 | 622 | oxytetracycline | 8 µM | HL60 | 0.68 | 0.069 | -0.116 | 1553 |
| 223 | 671 | pyrithyldione | 24 µM | MCF7 | 0.679 | 0.078 | -0.107 | 3482 |
| 224 | 702 | dicoumarol | 12 µM | PC3 | 0.679 | 0.089 | -0.095 | 4323 |
| 225 | 677 | piribedil | 12 µM | MCF7 | 0.678 | 0.074 | -0.11 | 3512 |
| 226 | 699 | sulfadimethoxine | 13 µM | MCF7 | 0.678 | 0.065 | -0.12 | 4724 |
| 227 | 656 | trimethadione | 28 µM | MCF7 | 0.677 | 0.066 | -0.118 | 2846 |
| 228 | 738 | sulfaphenazole | 13 µM | MCF7 | 0.677 | 0.065 | -0.119 | 5507 |
| 229 | 732 | naltrexone | 10 µM | PC3 | 0.677 | 0.116 | -0.068 | 5765 |
| 230 | 615 | piroxicam | 12 µM | HL60 | 0.676 | 0.069 | -0.115 | 1405 |
| 231 | 719 | cyclic adenosine monophosphate | 12 µM | PC3 | 0.676 | 0.055 | -0.129 | 5108 |
| 232 | 687 | Prestwick-1080 | 15 µM | MCF7 | 0.675 | 0.077 | -0.107 | 3878 |
| 233 | 756 | pirlindole | 12 µM | MCF7 | 0.675 | 0.067 | -0.116 | 6519 |
| 234 | 1073 | MG-262 | 100 nM | PC3 | 0.674 | 0.064 | -0.119 | 7079 |
| 235 | 505 | 5149715 | 10 µM | MCF7 | 0.674 | 0.102 | -0.081 | 890 |
| 236 | 725 | valproic acid | 500 µM | MCF7 | 0.674 | 0.086 | -0.097 | 5237 |
| 237 | 613 | glipizide | 9 µM | HL60 | 0.673 | 0.073 | -0.11 | 2008 |
| 238 | 671 | butacaine | 13 µM | MCF7 | 0.673 | 0.101 | -0.082 | 3469 |
| 239 | 641 | isocorydine | 12 µM | HL60 | 0.672 | 0.08 | -0.103 | 1787 |
| 240 | 650 | fulvestrant | 1 µM | HL60 | 0.672 | 0.088 | -0.095 | 2665 |
| 241 | 653 | fusidic acid | 7 µM | MCF7 | 0.671 | 0.055 | -0.128 | 2647 |
| 242 | 644 | co-dergocrine mesilate | 6 µM | HL60 | 0.671 | 0.079 | -0.104 | 2136 |
| 243 | 646 | furosemide | 12 µM | MCF7 | 0.67 | 0.072 | -0.11 | 3197 |
| 244 | 756 | halofantrine | 7 µM | MCF7 | 0.67 | 0.089 | -0.093 | 6509 |
| 245 | 644 | harmaline | 14 µM | HL60 | 0.67 | 0.053 | -0.129 | 2149 |
| 246 | 619 | pergolide | 10 µM | HL60 | 0.67 | 0.081 | -0.101 | 2403 |
| 247 | 627 | morantel | 11 µM | MCF7 | 0.669 | 0.095 | -0.087 | 1676 |
| 248 | 762 | Prestwick-1103 | 20 µM | PC3 | 0.669 | 0.092 | -0.09 | 7317 |
| 249 | 749 | chloropyrazine | 35 µM | HL60 | 0.669 | 0.082 | -0.1 | 6148 |
| 250 | 642 | dantrolene | 12 µM | MCF7 | 0.669 | 0.086 | -0.096 | 2329 |
| 251 | 772 | difenidol | 12 µM | MCF7 | 0.669 | 0.106 | -0.075 | 7447 |
| 252 | 688 | cinchonine | 14 µM | PC3 | 0.668 | 0.032 | -0.149 | 3988 |
| 253 | 661 | alfadolone | 10 µM | HL60 | 0.667 | 0.056 | -0.125 | 3127 |
| 254 | 662 | noscapine | 10 µM | MCF7 | 0.665 | 0.094 | -0.087 | 2745 |
| 255 | 756 | nipecotic acid | 31 µM | MCF7 | 0.664 | 0.072 | -0.109 | 6500 |
| 256 | 646 | terbutaline | 7 µM | MCF7 | 0.664 | 0.07 | -0.111 | 3202 |
| 257 | 771 | dimenhydrinate | 9 µM | MCF7 | 0.664 | 0.072 | -0.109 | 7431 |
| 258 | 636 | acenocoumarol | 11 µM | MCF7 | 0.664 | 0.043 | -0.138 | 2240 |
| 259 | 618 | lomefloxacin | 10 µM | HL60 | 0.664 | 0.085 | -0.095 | 2348 |
| 260 | 618 | metoclopramide | 12 µM | HL60 | 0.664 | 0.074 | -0.106 | 2353 |
| 261 | 713 | digoxigenin | 10 µM | PC3 | 0.663 | 0.09 | -0.09 | 4680 |
| 262 | 653 | arecoline | 17 µM | MCF7 | 0.663 | 0.085 | -0.095 | 2657 |
| 263 | 662 | convolamine | 12 µM | MCF7 | 0.663 | 0.06 | -0.12 | 2771 |
| 264 | 725 | trifluoperazine | 10 µM | MCF7 | 0.662 | 0.082 | -0.098 | 5221 |
| 265 | 61 | tanespimycin | 1 µM | PC3 | 0.662 | 0.076 | -0.104 | 450 |
| 266 | 733 | folic acid | 9 µM | PC3 | 0.662 | 0.1 | -0.08 | 5844 |
| 267 | 670 | medrysone | 12 µM | MCF7 | 0.662 | 0.091 | -0.089 | 3403 |
| 268 | 622 | bupropion | 14 µM | HL60 | 0.66 | 0.062 | -0.117 | 1564 |
| 269 | 673 | propofol | 22 µM | MCF7 | 0.66 | 0.088 | -0.091 | 3386 |
| 270 | 612 | levodopa | 20 µM | HL60 | 0.66 | 0.048 | -0.131 | 1972 |
| 271 | 738 | carbamazepine | 17 µM | MCF7 | 0.659 | 0.097 | -0.082 | 5518 |
| 272 | 506 | estradiol | 100 nM | MCF7 | 0.659 | 0.073 | -0.106 | 988 |
| 273 | 708 | dimethadione | 31 µM | MCF7 | 0.659 | 0.036 | -0.144 | 5668 |
| 274 | 1091 | BCB000039 | 10 µM | PC3 | 0.659 | 0.081 | -0.098 | 7536 |
| 275 | 661 | aminocaproic acid | 30 µM | HL60 | 0.658 | 0.071 | -0.108 | 3122 |
| 276 | 612 | diphemanil metilsulfate | 10 µM | HL60 | 0.657 | 0.078 | -0.1 | 1994 |
| 277 | 612 | minoxidil | 19 µM | HL60 | 0.656 | 0.066 | -0.113 | 1996 |
| 278 | 729 | nitrofural | 20 µM | MCF7 | 0.656 | 0.086 | -0.092 | 5321 |
| 279 | 1038 | STOCK1N-35696 | 15 µM | PC3 | 0.655 | 0.07 | -0.108 | 6564 |
| 280 | 704 | ethionamide | 24 µM | PC3 | 0.655 | 0.073 | -0.105 | 4593 |
| 281 | 661 | gliquidone | 8 µM | HL60 | 0.654 | 0.058 | -0.12 | 3126 |
| 282 | 622 | diperodon | 9 µM | HL60 | 0.654 | 0.044 | -0.134 | 1575 |
| 283 | 628 | isoxicam | 12 µM | PC3 | 0.653 | 0.075 | -0.103 | 1820 |
| 284 | 634 | serotonin | 19 µM | HL60 | 0.653 | 0.074 | -0.103 | 2449 |
| 285 | 623 | methotrexate | 9 µM | HL60 | 0.653 | 0.082 | -0.095 | 1599 |
| 286 | 771 | disopyramide | 12 µM | MCF7 | 0.652 | 0.097 | -0.08 | 7439 |
| 287 | 634 | ciclopirox | 15 µM | HL60 | 0.652 | 0.065 | -0.113 | 2456 |
| 288 | 692 | galantamine | 11 µM | PC3 | 0.652 | 0.075 | -0.103 | 4186 |
| 289 | 719 | metampicillin | 10 µM | PC3 | 0.65 | 0.122 | -0.055 | 5115 |
| 290 | 1082 | suramin sodium | 10 µM | MCF7 | 0.65 | 0.089 | -0.088 | 7496 |
| 291 | 738 | metampicillin | 10 µM | MCF7 | 0.65 | 0.07 | -0.106 | 5540 |
| 292 | 673 | thioperamide | 10 µM | MCF7 | 0.65 | 0.074 | -0.103 | 3392 |
| 293 | 614 | midodrine | 14 µM | HL60 | 0.65 | 0.073 | -0.104 | 1403 |
| 294 | 677 | levopropoxyphene | 7 µM | MCF7 | 0.649 | 0.072 | -0.104 | 3543 |
| 295 | 645 | Prestwick-685 | 11 µM | HL60 | 0.649 | 0.077 | -0.099 | 2188 |
| 296 | 753 | melatonin | 17 µM | PC3 | 0.649 | 0.076 | -0.101 | 6293 |
| 297 | 671 | doxycycline | 8 µM | MCF7 | 0.649 | 0.101 | -0.076 | 3479 |
| 298 | 685 | PNU-0230031 | 1 µM | MCF7 | 0.649 | 0.082 | -0.095 | 3632 |
| 299 | 718 | perhexiline | 10 µM | PC3 | 0.648 | 0.128 | -0.049 | 5081 |
| 300 | 614 | dihydrostreptomycin | 3 µM | HL60 | 0.648 | 0.054 | -0.122 | 1391 |
| 301 | 761 | morantel | 11 µM | PC3 | 0.648 | 0.052 | -0.125 | 7250 |
| 302 | 706 | retrorsine | 11 µM | MCF7 | 0.648 | 0.065 | -0.111 | 4946 |
| 303 | 747 | cefotaxime | 8 µM | MCF7 | 0.648 | 0.069 | -0.107 | 7186 |
| 304 | 636 | tetracycline | 8 µM | MCF7 | 0.647 | 0.075 | -0.101 | 2243 |
| 305 | 1047 | PHA-00767505E | 10 µM | MCF7 | 0.647 | 0.097 | -0.079 | 6596 |
| 306 | 629 | thiamphenicol | 11 µM | HL60 | 0.647 | 0.061 | -0.115 | 1867 |
| 307 | 653 | bisoprolol | 9 µM | MCF7 | 0.646 | 0.079 | -0.096 | 2642 |
| 308 | 628 | nifedipine | 12 µM | PC3 | 0.646 | 0.057 | -0.119 | 1814 |
| 309 | 672 | theobromine | 22 µM | MCF7 | 0.645 | 0.104 | -0.072 | 3334 |
| 310 | 632 | acebutolol | 11 µM | MCF7 | 0.645 | 0.08 | -0.096 | 1493 |
| 311 | 656 | sisomicin | 3 µM | MCF7 | 0.645 | 0.09 | -0.086 | 2853 |
| 312 | 654 | carcinine | 22 µM | MCF7 | 0.645 | 0.078 | -0.097 | 3242 |
| 313 | 726 | 3-hydroxy-DL-kynurenine | 18 µM | MCF7 | 0.645 | 0.08 | -0.095 | 5276 |
| 314 | 734 | gabapentin | 23 µM | PC3 | 0.644 | 0.095 | -0.08 | 5875 |
| 315 | 738 | oxolinic acid | 15 µM | MCF7 | 0.644 | 0.081 | -0.094 | 5519 |
| 316 | 744 | sulfanilamide | 23 µM | MCF7 | 0.644 | 0.084 | -0.091 | 6810 |
| 317 | 708 | betaxolol | 12 µM | MCF7 | 0.643 | 0.044 | -0.131 | 5669 |
| 318 | 725 | LY-294002 | 10 µM | MCF7 | 0.643 | 0.068 | -0.107 | 5233 |
| 319 | 1053 | carmustine | 100 µM | MCF7 | 0.641 | 0.077 | -0.097 | 6888 |
| 320 | 764 | pyrithyldione | 24 µM | PC3 | 0.641 | 0.089 | -0.085 | 7153 |
| 321 | 732 | ondansetron | 12 µM | PC3 | 0.641 | 0.097 | -0.077 | 5796 |
| 322 | 711 | fludrocortisone | 9 µM | MCF7 | 0.64 | 0.066 | -0.108 | 3977 |
| 323 | 752 | butirosin | 5 µM | MCF7 | 0.639 | 0.082 | -0.091 | 6080 |
| 324 | 754 | flavoxate | 9 µM | PC3 | 0.638 | 0.083 | -0.09 | 6326 |
| 325 | 757 | estradiol | 10 nM | MCF7 | 0.638 | 0.07 | -0.104 | 5601 |
| 326 | 650 | 15-delta prostaglandin J2 | 10 µM | HL60 | 0.638 | 0.075 | -0.098 | 2691 |
| 327 | 650 | wortmannin | 10 nM | HL60 | 0.637 | 0.06 | -0.113 | 2703 |
| 328 | 733 | isoetarine | 12 µM | PC3 | 0.637 | 0.087 | -0.087 | 5812 |
| 329 | 687 | lycorine | 12 µM | MCF7 | 0.637 | 0.059 | -0.114 | 3891 |
| 330 | 766 | Prestwick-1082 | 12 µM | MCF7 | 0.637 | 0.082 | -0.091 | 7027 |
| 331 | 1064 | lomustine | 100 µM | MCF7 | 0.637 | 0.076 | -0.097 | 7045 |
| 332 | 66 | celecoxib | 10 µM | PC3 | 0.636 | 0.084 | -0.089 | 482 |
| 333 | 642 | fludrocortisone | 9 µM | MCF7 | 0.635 | 0.059 | -0.114 | 2328 |
| 334 | 747 | ticarcillin | 9 µM | MCF7 | 0.635 | 0.105 | -0.068 | 7185 |
| 335 | 708 | perphenazine | 10 µM | MCF7 | 0.635 | 0.098 | -0.074 | 5698 |
| 336 | 646 | etacrynic acid | 13 µM | MCF7 | 0.634 | 0.053 | -0.119 | 3181 |
| 337 | 640 | dirithromycin | 5 µM | HL60 | 0.634 | 0.064 | -0.108 | 1712 |
| 338 | 1000 | AG-013608 | 10 µM | MCF7 | 0.633 | 0.06 | -0.112 | 5904 |
| 339 | 690 | nafcillin | 9 µM | MCF7 | 0.633 | 0.074 | -0.098 | 4103 |
| 340 | 616 | dihydroergotamine | 3 µM | PC3 | 0.633 | 0.123 | -0.049 | 2081 |
| 341 | 662 | vinburnine | 14 µM | MCF7 | 0.633 | 0.09 | -0.082 | 2781 |
| 342 | 649 | furaltadone | 11 µM | HL60 | 0.632 | 0.06 | -0.112 | 2554 |
| 343 | 644 | yohimbic acid | 11 µM | HL60 | 0.632 | 0.063 | -0.108 | 2147 |
| 344 | 704 | Prestwick-692 | 7 µM | PC3 | 0.632 | 0.068 | -0.104 | 4599 |
| 345 | 502 | 5230742 | 17 µM | MCF7 | 0.632 | 0.091 | -0.081 | 970 |
| 346 | 694 | epitiostanol | 13 µM | MCF7 | 0.631 | 0.079 | -0.093 | 4788 |
| 347 | 745 | fluorocurarine | 12 µM | MCF7 | 0.631 | 0.062 | -0.11 | 6219 |
| 348 | 505 | ikarugamycin | 2 µM | MCF7 | 0.631 | 0.106 | -0.066 | 918 |
| 349 | 654 | ciclosporin | 3 µM | MCF7 | 0.63 | 0.062 | -0.11 | 3267 |
| 350 | 654 | mebhydrolin | 5 µM | MCF7 | 0.63 | 0.106 | -0.065 | 3269 |
| 351 | 502 | pyrvinium | 1 µM | MCF7 | 0.629 | 0.097 | -0.074 | 978 |
| 352 | 731 | terbutaline | 7 µM | PC3 | 0.629 | 0.076 | -0.095 | 5764 |
| 353 | 731 | ciprofibrate | 14 µM | PC3 | 0.628 | 0.082 | -0.089 | 5740 |
| 354 | 713 | aciclovir | 18 µM | PC3 | 0.628 | 0.049 | -0.122 | 4683 |
| 355 | 720 | Prestwick-1080 | 15 µM | MCF7 | 0.628 | 0.071 | -0.1 | 4354 |
| 356 | 672 | liothyronine | 6 µM | MCF7 | 0.628 | 0.077 | -0.094 | 3324 |
| 357 | 766 | morantel | 11 µM | MCF7 | 0.627 | 0.098 | -0.072 | 7010 |
| 358 | 704 | 3-acetamidocoumarin | 20 µM | PC3 | 0.627 | 0.085 | -0.085 | 4601 |
| 359 | 662 | riboflavin | 11 µM | MCF7 | 0.627 | 0.088 | -0.083 | 2760 |
| 360 | 709 | sulfabenzamide | 14 µM | PC3 | 0.626 | 0.063 | -0.107 | 6634 |
| 361 | 610 | hexamethonium bromide | 10 µM | PC3 | 0.626 | 0.111 | -0.059 | 1901 |
| 362 | 653 | domperidone | 7 µM | MCF7 | 0.625 | 0.099 | -0.071 | 2655 |
| 363 | 695 | minaprine | 11 µM | MCF7 | 0.625 | 0.082 | -0.088 | 4814 |
| 364 | 613 | paclitaxel | 5 µM | HL60 | 0.625 | 0.048 | -0.121 | 2043 |
| 365 | 613 | metanephrine | 17 µM | HL60 | 0.625 | 0.079 | -0.091 | 2015 |
| 366 | 690 | amiprilose | 12 µM | MCF7 | 0.624 | 0.083 | -0.087 | 4119 |
| 367 | 634 | ergocalciferol | 10 µM | HL60 | 0.624 | 0.072 | -0.097 | 2442 |
| 368 | 656 | pregnenolone | 13 µM | MCF7 | 0.624 | 0.059 | -0.11 | 2856 |
| 369 | 658 | Prestwick-857 | 12 µM | HL60 | 0.624 | 0.057 | -0.113 | 3016 |
| 370 | 734 | acenocoumarol | 11 µM | PC3 | 0.624 | 0.094 | -0.076 | 5878 |
| 371 | 632 | miconazole | 10 µM | MCF7 | 0.623 | 0.074 | -0.095 | 1477 |
| 372 | 731 | mevalolactone | 31 µM | PC3 | 0.623 | 0.052 | -0.117 | 5738 |
| 373 | 69 | iloprost | 1 µM | SKMEL5 | 0.622 | 0.069 | -0.1 | 496 |
| 374 | 637 | metampicillin | 10 µM | MCF7 | 0.622 | 0.059 | -0.11 | 2286 |
| 375 | 704 | iocetamic acid | 7 µM | PC3 | 0.622 | 0.055 | -0.114 | 4600 |
| 376 | 680 | PNU-0251126 | 10 µM | PC3 | 0.621 | 0.099 | -0.07 | 3689 |
| 377 | 772 | atracurium besilate | 3 µM | MCF7 | 0.619 | 0.097 | -0.071 | 7477 |
| 378 | 745 | tranexamic acid | 25 µM | MCF7 | 0.619 | 0.055 | -0.114 | 6238 |
| 379 | 680 | atractyloside | 5 µM | PC3 | 0.619 | 0.061 | -0.108 | 3695 |
| 380 | 1057 | AH-23848 | 1 µM | MCF7 | 0.619 | 0.087 | -0.081 | 6903 |
| 381 | 632 | hydrochlorothiazide | 13 µM | MCF7 | 0.619 | 0.079 | -0.089 | 1487 |
| 382 | 754 | acemetacin | 10 µM | PC3 | 0.618 | 0.067 | -0.101 | 6361 |
| 383 | 708 | dioxybenzone | 16 µM | MCF7 | 0.618 | 0.1 | -0.068 | 5699 |
| 384 | 623 | practolol | 15 µM | HL60 | 0.618 | 0.076 | -0.092 | 1587 |
| 385 | 634 | ceforanide | 8 µM | HL60 | 0.618 | 0.06 | -0.108 | 2447 |
| 386 | 753 | arecoline | 17 µM | PC3 | 0.618 | 0.053 | -0.115 | 6322 |
| 387 | 729 | hesperidin | 7 µM | MCF7 | 0.617 | 0.102 | -0.066 | 5313 |
| 388 | 1008 | monorden | 100 nM | MCF7 | 0.617 | 0.096 | -0.072 | 5947 |
| 389 | 635 | tetramisole | 17 µM | HL60 | 0.616 | 0.085 | -0.083 | 2489 |
| 390 | 1088 | daunorubicin | 1 µM | MCF7 | 0.616 | 0.114 | -0.053 | 7525 |
| 391 | 661 | deptropine | 8 µM | HL60 | 0.616 | 0.083 | -0.084 | 3144 |
| 392 | 630 | 3-hydroxy-DL-kynurenine | 18 µM | HL60 | 0.616 | 0.06 | -0.108 | 1300 |
| 393 | 670 | spiramycin | 5 µM | MCF7 | 0.615 | 0.069 | -0.098 | 3419 |
| 394 | 714 | diltiazem | 9 µM | PC3 | 0.614 | 0.086 | -0.081 | 6710 |
| 395 | 735 | dinoprost | 8 µM | MCF7 | 0.614 | 0.087 | -0.08 | 5409 |
| 396 | 662 | hydroquinine | 9 µM | MCF7 | 0.614 | 0.063 | -0.103 | 2767 |
| 397 | 59 | clofibrate | 100 µM | MCF7 | 0.613 | 0.075 | -0.092 | 444 |
| 398 | 702 | flecainide | 8 µM | PC3 | 0.613 | 0.068 | -0.099 | 4318 |
| 399 | 654 | meclocycline | 6 µM | MCF7 | 0.613 | 0.082 | -0.085 | 3277 |
| 400 | 772 | levamisole | 17 µM | MCF7 | 0.612 | 0.068 | -0.099 | 7450 |
| 401 | 1032 | PHA-00767505E | 10 µM | PC3 | 0.611 | 0.062 | -0.104 | 6545 |
| 402 | 676 | epitiostanol | 13 µM | MCF7 | 0.61 | 0.078 | -0.088 | 7342 |
| 403 | 711 | proglumide | 12 µM | MCF7 | 0.61 | 0.099 | -0.067 | 3972 |
| 404 | 728 | clofilium tosylate | 8 µM | PC3 | 0.61 | 0.04 | -0.126 | 4492 |
| 405 | 708 | naringin | 7 µM | MCF7 | 0.61 | 0.069 | -0.097 | 5666 |
| 406 | 644 | gramine | 23 µM | HL60 | 0.61 | 0.063 | -0.103 | 2143 |
| 407 | 645 | pramocaine | 12 µM | HL60 | 0.61 | 0.067 | -0.099 | 2198 |
| 408 | 649 | flecainide | 8 µM | HL60 | 0.61 | 0.062 | -0.104 | 2557 |
| 409 | 686 | deferoxamine | 6 µM | MCF7 | 0.61 | 0.073 | -0.093 | 3842 |
| 410 | 766 | chloroquine | 8 µM | MCF7 | 0.61 | 0.074 | -0.092 | 7012 |
| 411 | 746 | hydroquinine | 9 µM | MCF7 | 0.609 | 0.073 | -0.092 | 6263 |
| 412 | 676 | fursultiamine | 9 µM | MCF7 | 0.608 | 0.052 | -0.113 | 7349 |
| 413 | 738 | flumequine | 15 µM | MCF7 | 0.608 | 0.076 | -0.089 | 5529 |
| 414 | 750 | trichostatin A | 100 nM | HL60 | 0.608 | 0.073 | -0.093 | 6171 |
| 415 | 662 | pseudopelletierine | 21 µM | MCF7 | 0.608 | 0.1 | -0.065 | 2766 |
| 416 | 513 | genistein | 10 µM | MCF7 | 0.607 | 0.086 | -0.079 | 1073 |
| 417 | 737 | hydrastinine | 16 µM | MCF7 | 0.607 | 0.098 | -0.067 | 5494 |
| 418 | 648 | naproxen | 16 µM | HL60 | 0.606 | 0.057 | -0.108 | 2533 |
| 419 | 95 | geldanamycin | 1 µM | MCF7 | 0.606 | 0.056 | -0.108 | 593 |
| 420 | 730 | bisoprolol | 9 µM | MCF7 | 0.605 | 0.067 | -0.098 | 5348 |
| 421 | 1047 | 16,16-dimethylprostaglandin E2 | 10 µM | MCF7 | 0.605 | 0.079 | -0.085 | 6597 |
| 422 | 771 | glafenine | 10 µM | MCF7 | 0.605 | 0.092 | -0.073 | 7418 |
| 423 | 654 | Chicago Sky Blue 6B | 4 µM | MCF7 | 0.605 | 0.054 | -0.111 | 3266 |
| 424 | 1030 | AG-013608 | 10 µM | MCF7 | 0.605 | 0.095 | -0.069 | 6435 |
| 425 | 40 | cobalt chloride | 100 µM | MCF7 | 0.604 | 0.075 | -0.089 | 383 |
| 426 | 715 | N6-methyladenosine | 14 µM | PC3 | 0.604 | 0.098 | -0.066 | 6732 |
| 427 | 681 | etiocholanolone | 14 µM | PC3 | 0.604 | 0.087 | -0.077 | 3742 |
| 428 | 743 | monocrotaline | 12 µM | MCF7 | 0.603 | 0.044 | -0.12 | 6771 |
| 429 | 733 | suxibuzone | 9 µM | PC3 | 0.603 | 0.063 | -0.101 | 5806 |
| 430 | 728 | iopromide | 5 µM | PC3 | 0.603 | 0.068 | -0.096 | 4504 |
| 431 | 505 | topiramate | 3 µM | MCF7 | 0.603 | 0.062 | -0.101 | 915 |
| 432 | 711 | dantrolene | 12 µM | MCF7 | 0.601 | 0.058 | -0.105 | 3978 |
| 433 | 647 | ambroxol | 10 µM | MCF7 | 0.601 | 0.084 | -0.079 | 3238 |
| 434 | 728 | altizide | 10 µM | PC3 | 0.601 | 0.08 | -0.083 | 4491 |
| 435 | 726 | clofazimine | 8 µM | MCF7 | 0.6 | 0.084 | -0.079 | 5277 |
| 436 | 1009 | rotenone | 1 µM | PC3 | 0.6 | 0.088 | -0.075 | 5948 |
| 437 | 610 | sulfathiazole | 16 µM | PC3 | 0.599 | 0.073 | -0.09 | 1883 |
| 438 | 1014 | sirolimus | 100 nM | MCF7 | 0.599 | 0.054 | -0.109 | 5975 |
| 439 | 17 | tomelukast | 1 µM | MCF7 | 0.599 | 0.1 | -0.063 | 222 |
| 440 | 22a | fludrocortisone | 1 µM | MCF7 | 0.599 | 0.089 | -0.074 | 281 |
| 441 | 725 | alpha-estradiol | 10 nM | MCF7 | 0.599 | 0.089 | -0.074 | 5207 |
| 442 | 673 | digoxigenin | 10 µM | MCF7 | 0.599 | 0.062 | -0.101 | 3397 |
| 443 | 1057 | orlistat | 10 µM | MCF7 | 0.599 | 0.077 | -0.085 | 6905 |
| 444 | 112 | tioguanine | 10 µM | MCF7 | 0.598 | 0.045 | -0.118 | 642 |
| 445 | 728 | kinetin | 19 µM | PC3 | 0.598 | 0.068 | -0.095 | 4477 |
| 446 | 628 | metrifonate | 16 µM | PC3 | 0.598 | 0.077 | -0.085 | 1797 |
| 447 | 685 | heliotrine | 13 µM | MCF7 | 0.598 | 0.097 | -0.066 | 3615 |
| 448 | 615 | metampicillin | 10 µM | HL60 | 0.597 | 0.073 | -0.089 | 1440 |
| 449 | 640 | scoulerine | 12 µM | HL60 | 0.596 | 0.071 | -0.091 | 1742 |
| 450 | 626 | valproic acid | 50 µM | MCF7 | 0.596 | 0.115 | -0.047 | 1647 |
| 451 | 612 | phenazone | 21 µM | HL60 | 0.595 | 0.049 | -0.113 | 1989 |
| 452 | 733 | Prestwick-689 | 10 µM | PC3 | 0.595 | 0.092 | -0.07 | 5816 |
| 453 | 677 | proguanil | 14 µM | MCF7 | 0.594 | 0.068 | -0.094 | 3505 |
| 454 | 681 | nefopam | 14 µM | PC3 | 0.594 | 0.077 | -0.085 | 3730 |
| 455 | 744 | metoprolol | 6 µM | MCF7 | 0.594 | 0.079 | -0.082 | 6846 |
| 456 | 636 | ampyrone | 20 µM | MCF7 | 0.593 | 0.063 | -0.098 | 2249 |
| 457 | 741 | thalidomide | 15 µM | MCF7 | 0.593 | 0.075 | -0.086 | 5990 |
| 458 | 96 | MK-886 | 1 µM | MCF7 | 0.591 | 0.08 | -0.08 | 601 |
| 459 | 623 | zaprinast | 15 µM | HL60 | 0.591 | 0.059 | -0.102 | 1611 |
| 460 | 766 | moxisylyte | 13 µM | MCF7 | 0.591 | 0.085 | -0.076 | 7015 |
| 461 | 705 | dicycloverine | 12 µM | MCF7 | 0.591 | 0.059 | -0.102 | 4405 |
| 462 | 1091 | fulvestrant | 1 µM | PC3 | 0.591 | 0.075 | -0.086 | 7539 |
| 463 | 628 | hydroflumethiazide | 12 µM | PC3 | 0.591 | 0.108 | -0.053 | 1809 |
| 464 | 695 | tolazoline | 20 µM | MCF7 | 0.59 | 0.083 | -0.077 | 4844 |
| 465 | 649 | guanadrel | 8 µM | HL60 | 0.59 | 0.064 | -0.096 | 2575 |
| 466 | 714 | florfenicol | 11 µM | PC3 | 0.589 | 0.059 | -0.101 | 6701 |
| 467 | 603 | tanespimycin | 1 µM | PC3 | 0.589 | 0.093 | -0.067 | 1218 |
| 468 | 725 | wortmannin | 10 nM | MCF7 | 0.588 | 0.074 | -0.086 | 5240 |
| 469 | 676 | proscillaridin | 8 µM | MCF7 | 0.588 | 0.049 | -0.11 | 7340 |
| 470 | 662 | yohimbine | 10 µM | MCF7 | 0.588 | 0.052 | -0.108 | 2755 |
| 471 | 1057 | STOCK1N-28457 | 20 µM | MCF7 | 0.587 | 0.092 | -0.068 | 6906 |
| 472 | 663 | Prestwick-642 | 14 µM | MCF7 | 0.587 | 0.049 | -0.11 | 2815 |
| 473 | 734 | sulfadoxine | 13 µM | PC3 | 0.587 | 0.093 | -0.067 | 5852 |
| 474 | 615 | pentetrazol | 29 µM | HL60 | 0.586 | 0.069 | -0.09 | 1408 |
| 475 | 718 | diprophylline | 16 µM | PC3 | 0.585 | 0.092 | -0.067 | 5063 |
| 476 | 771 | dyclonine | 12 µM | MCF7 | 0.585 | 0.082 | -0.077 | 7423 |
| 477 | 708 | domperidone | 7 µM | MCF7 | 0.585 | 0.062 | -0.098 | 5701 |
| 478 | 636 | nomifensine | 11 µM | MCF7 | 0.585 | 0.087 | -0.072 | 2224 |
| 479 | 636 | naltrexone | 10 µM | MCF7 | 0.585 | 0.047 | -0.112 | 2209 |
| 480 | 691 | alprostadil | 11 µM | MCF7 | 0.585 | 0.054 | -0.105 | 4179 |
| 481 | 654 | levothyroxine sodium | 5 µM | MCF7 | 0.584 | 0.078 | -0.081 | 3249 |
| 482 | 658 | nafcillin | 9 µM | HL60 | 0.584 | 0.04 | -0.119 | 2983 |
| 483 | 712 | josamycin | 5 µM | PC3 | 0.584 | 0.092 | -0.067 | 4631 |
| 484 | 755 | nitrendipine | 11 µM | MCF7 | 0.584 | 0.095 | -0.063 | 6464 |
| 485 | 772 | tetracaine | 13 µM | MCF7 | 0.584 | 0.079 | -0.08 | 7473 |
| 486 | 646 | streptomycin | 3 µM | MCF7 | 0.583 | 0.064 | -0.094 | 3195 |
| 487 | 691 | L-methionine sulfoximine | 22 µM | MCF7 | 0.583 | 0.054 | -0.104 | 4151 |
| 488 | 663 | diflorasone | 8 µM | MCF7 | 0.583 | 0.075 | -0.084 | 2798 |
| 489 | 1043 | PHA-00665752 | 10 µM | MCF7 | 0.583 | 0.08 | -0.078 | 6578 |
| 490 | 719 | flucloxacillin | 8 µM | PC3 | 0.582 | 0.081 | -0.077 | 5102 |
| 491 | 628 | thiamphenicol | 11 µM | PC3 | 0.582 | 0.085 | -0.073 | 1826 |
| 492 | 761 | adiphenine | 11 µM | PC3 | 0.582 | 0.084 | -0.074 | 7279 |
| 493 | 94 | probucol | 10 µM | MCF7 | 0.582 | 0.088 | -0.071 | 592 |
| 494 | 1061 | carmustine | 100 µM | MCF7 | 0.582 | 0.116 | -0.042 | 6914 |
| 495 | 756 | adrenosterone | 13 µM | MCF7 | 0.581 | 0.061 | -0.097 | 6486 |
| 496 | 616 | naltrexone | 10 µM | PC3 | 0.581 | 0.07 | -0.088 | 2047 |
| 497 | 725 | rosiglitazone | 10 µM | MCF7 | 0.581 | 0.064 | -0.093 | 5230 |
| 498 | 617 | mefexamide | 13 µM | PC3 | 0.58 | 0.051 | -0.107 | 2121 |
| 499 | 22a | tacrolimus | 1 µM | MCF7 | 0.58 | 0.076 | -0.082 | 284 |
| 500 | 633 | cyproheptadine | 12 µM | MCF7 | 0.58 | 0.053 | -0.105 | 1521 |
| 501 | 735 | amphotericin B | 4 µM | MCF7 | 0.579 | 0.071 | -0.087 | 5404 |
| 502 | 657 | tubocurarine chloride | 5 µM | MCF7 | 0.579 | 0.076 | -0.082 | 2887 |
| 503 | 610 | diphemanil metilsulfate | 10 µM | PC3 | 0.579 | 0.111 | -0.047 | 1912 |
| 504 | 1077 | fulvestrant | 1 µM | PC3 | 0.579 | 0.099 | -0.058 | 7096 |
| 505 | 645 | mepenzolate bromide | 10 µM | HL60 | 0.578 | 0.051 | -0.107 | 2169 |
| 506 | 648 | tetrandrine | 6 µM | HL60 | 0.578 | 0.081 | -0.076 | 2520 |
| 507 | 615 | nifenazone | 13 µM | HL60 | 0.578 | 0.052 | -0.105 | 1439 |
| 508 | 711 | piperacillin | 7 µM | MCF7 | 0.577 | 0.054 | -0.102 | 3939 |
| 509 | 628 | heptaminol | 22 µM | PC3 | 0.577 | 0.084 | -0.073 | 1825 |
| 510 | 741 | lansoprazole | 11 µM | MCF7 | 0.576 | 0.064 | -0.093 | 6009 |
| 511 | 732 | mianserin | 13 µM | PC3 | 0.576 | 0.053 | -0.104 | 5786 |
| 512 | 708 | dehydrocholic acid | 10 µM | MCF7 | 0.576 | 0.08 | -0.077 | 5681 |
| 513 | 745 | chloropyrazine | 35 µM | MCF7 | 0.576 | 0.091 | -0.065 | 6227 |
| 514 | 729 | chlorprothixene | 11 µM | MCF7 | 0.575 | 0.077 | -0.079 | 5291 |
| 515 | 1014 | PF-00539745-00 | 10 µM | MCF7 | 0.575 | 0.057 | -0.1 | 5974 |
| 516 | 1082 | meteneprost | 10 µM | MCF7 | 0.575 | 0.087 | -0.07 | 7500 |
| 517 | 659 | crotamiton | 20 µM | HL60 | 0.575 | 0.067 | -0.089 | 3050 |
| 518 | 648 | sulfamethizole | 15 µM | HL60 | 0.574 | 0.051 | -0.105 | 2536 |
| 519 | 772 | pimethixene | 10 µM | MCF7 | 0.574 | 0.082 | -0.074 | 7468 |
| 520 | 1088 | 15(S)-15-methylprostaglandin E2 | 10 µM | MCF7 | 0.574 | 0.099 | -0.057 | 7521 |
| 521 | 1005 | PF-00875133-00 | 10 µM | PC3 | 0.574 | 0.103 | -0.053 | 5928 |
| 522 | 87 | dimethyloxalylglycine | 1 mM | PC3 | 0.573 | 0.095 | -0.061 | 584 |
| 523 | 626 | rosiglitazone | 10 µM | MCF7 | 0.573 | 0.096 | -0.06 | 1658 |
| 524 | 602 | 15-delta prostaglandin J2 | 10 µM | HL60 | 0.573 | 0.05 | -0.106 | 1172 |
| 525 | 729 | profenamine | 11 µM | MCF7 | 0.573 | 0.055 | -0.1 | 5296 |
| 526 | 640 | Trolox C | 16 µM | HL60 | 0.572 | 0.067 | -0.088 | 1734 |
| 527 | 727 | genistein | 10 µM | PC3 | 0.572 | 0.066 | -0.089 | 4459 |
| 528 | 754 | iproniazid | 14 µM | PC3 | 0.572 | 0.067 | -0.088 | 6359 |
| 529 | 670 | calcium folinate | 8 µM | MCF7 | 0.571 | 0.049 | -0.107 | 3442 |
| 530 | 626 | genistein | 10 µM | MCF7 | 0.571 | 0.066 | -0.089 | 1660 |
| 531 | 632 | tolazoline | 20 µM | MCF7 | 0.571 | 0.055 | -0.1 | 1500 |
| 532 | 670 | tinidazole | 16 µM | MCF7 | 0.571 | 0.077 | -0.078 | 3430 |
| 533 | 718 | octopamine | 21 µM | PC3 | 0.57 | 0.108 | -0.048 | 5050 |
| 534 | 735 | carbimazole | 21 µM | MCF7 | 0.57 | 0.047 | -0.108 | 5399 |
| 535 | 627 | todralazine | 15 µM | MCF7 | 0.57 | 0.053 | -0.102 | 1677 |
| 536 | 628 | metformin | 24 µM | PC3 | 0.57 | 0.062 | -0.093 | 1816 |
| 537 | 700 | nefopam | 14 µM | MCF7 | 0.57 | 0.084 | -0.071 | 4752 |
| 538 | 702 | PHA-00851261E | 1 µM | PC3 | 0.569 | 0.057 | -0.098 | 4333 |
| 539 | 1091 | CP-319743 | 10 µM | PC3 | 0.569 | 0.08 | -0.075 | 7537 |
| 540 | 691 | myricetin | 13 µM | MCF7 | 0.568 | 0.065 | -0.089 | 4170 |
| 541 | 681 | karakoline | 11 µM | PC3 | 0.567 | 0.071 | -0.083 | 3741 |
| 542 | 726 | thioperamide | 10 µM | MCF7 | 0.567 | 0.056 | -0.098 | 5270 |
| 543 | 662 | racecadotril | 10 µM | MCF7 | 0.565 | 0.072 | -0.082 | 2774 |
| 544 | 712 | dehydrocholic acid | 10 µM | PC3 | 0.565 | 0.066 | -0.087 | 4620 |
| 545 | 700 | flumetasone | 10 µM | MCF7 | 0.565 | 0.048 | -0.106 | 4734 |
| 546 | 766 | mecamylamine | 20 µM | MCF7 | 0.565 | 0.062 | -0.092 | 7023 |
| 547 | 692 | isoxsuprine | 12 µM | PC3 | 0.565 | 0.093 | -0.061 | 4205 |
| 548 | 725 | estradiol | 10 nM | MCF7 | 0.565 | 0.047 | -0.106 | 5238 |
| 549 | 1019 | STOCK1N-35215 | 10 µM | PC3 | 0.564 | 0.069 | -0.085 | 6380 |
| 550 | 712 | felbinac | 19 µM | PC3 | 0.564 | 0.065 | -0.089 | 4639 |
| 551 | 758 | flurbiprofen | 16 µM | MCF7 | 0.564 | 0.07 | -0.084 | 5634 |
| 552 | 766 | levocabastine | 9 µM | MCF7 | 0.564 | 0.097 | -0.057 | 7009 |
| 553 | 636 | chlorphenamine | 10 µM | MCF7 | 0.564 | 0.067 | -0.086 | 2217 |
| 554 | 1012 | PF-00875133-00 | 10 µM | MCF7 | 0.564 | 0.077 | -0.077 | 5967 |
| 555 | 709 | retrorsine | 11 µM | PC3 | 0.563 | 0.071 | -0.083 | 6601 |
| 556 | 651 | proadifen | 10 µM | HL60 | 0.563 | 0.062 | -0.091 | 2707 |
| 557 | 756 | scopoletin | 21 µM | MCF7 | 0.562 | 0.088 | -0.065 | 6510 |
| 558 | 654 | dl-alpha tocopherol | 9 µM | MCF7 | 0.562 | 0.083 | -0.069 | 3256 |
| 559 | 772 | fenofibrate | 11 µM | MCF7 | 0.561 | 0.067 | -0.085 | 7474 |
| 560 | 687 | paroxetine | 1 µM | MCF7 | 0.561 | 0.066 | -0.086 | 3904 |
| 561 | 632 | minaprine | 11 µM | MCF7 | 0.561 | 0.055 | -0.097 | 1468 |
| 562 | 753 | nimodipine | 10 µM | PC3 | 0.561 | 0.076 | -0.076 | 6320 |
| 563 | 690 | etofenamate | 11 µM | MCF7 | 0.561 | 0.067 | -0.086 | 4108 |
| 564 | 651 | hecogenin | 9 µM | HL60 | 0.561 | 0.065 | -0.088 | 2716 |
| 565 | 706 | apramycin | 7 µM | MCF7 | 0.56 | 0.057 | -0.095 | 4959 |
| 566 | 650 | fluphenazine | 10 µM | HL60 | 0.56 | 0.047 | -0.106 | 2697 |
| 567 | 628 | benzonatate | 7 µM | PC3 | 0.56 | 0.098 | -0.054 | 1801 |
| 568 | 766 | glafenine | 10 µM | MCF7 | 0.559 | 0.062 | -0.091 | 7018 |
| 569 | 745 | alclometasone | 8 µM | MCF7 | 0.559 | 0.091 | -0.061 | 6229 |
| 570 | 614 | pindolol | 16 µM | HL60 | 0.559 | 0.055 | -0.097 | 1392 |
| 571 | 618 | hydralazine | 20 µM | HL60 | 0.559 | 0.078 | -0.074 | 2349 |
| 572 | 614 | terfenadine | 8 µM | HL60 | 0.559 | 0.057 | -0.095 | 1381 |
| 573 | 771 | triprolidine | 13 µM | MCF7 | 0.559 | 0.073 | -0.079 | 7408 |
| 574 | 677 | phensuximide | 21 µM | MCF7 | 0.558 | 0.063 | -0.089 | 3521 |
| 575 | 707 | etynodiol | 10 µM | MCF7 | 0.558 | 0.065 | -0.087 | 5024 |
| 576 | 505 | tanespimycin | 1 µM | MCF7 | 0.557 | 0.061 | -0.09 | 916 |
| 577 | 762 | fenspiride | 13 µM | PC3 | 0.557 | 0.069 | -0.082 | 7298 |
| 578 | 733 | cinnarizine | 11 µM | PC3 | 0.557 | 0.08 | -0.071 | 5817 |
| 579 | 680 | merbromin | 5 µM | PC3 | 0.557 | 0.099 | -0.053 | 3700 |
| 580 | 647 | maprotiline | 13 µM | MCF7 | 0.557 | 0.054 | -0.097 | 3236 |
| 581 | 730 | pilocarpine | 15 µM | MCF7 | 0.555 | 0.07 | -0.081 | 5341 |
| 582 | 772 | quinpirole | 16 µM | MCF7 | 0.555 | 0.082 | -0.069 | 7481 |
| 583 | 663 | harmaline | 14 µM | MCF7 | 0.555 | 0.102 | -0.049 | 2805 |
| 584 | 762 | ribavirin | 16 µM | PC3 | 0.555 | 0.086 | -0.065 | 7316 |
| 585 | 729 | ciprofloxacin | 11 µM | MCF7 | 0.554 | 0.078 | -0.073 | 5299 |
| 586 | 632 | cimetidine | 16 µM | MCF7 | 0.554 | 0.075 | -0.076 | 1464 |
| 587 | 650 | clozapine | 10 µM | HL60 | 0.554 | 0.079 | -0.071 | 2689 |
| 588 | 681 | gossypol | 8 µM | PC3 | 0.554 | 0.057 | -0.093 | 3740 |
| 589 | 681 | 0179445-0000 | 1 µM | PC3 | 0.554 | 0.041 | -0.109 | 3736 |
| 590 | 756 | pizotifen | 9 µM | MCF7 | 0.554 | 0.08 | -0.071 | 6513 |
| 591 | 734 | praziquantel | 13 µM | PC3 | 0.554 | 0.048 | -0.102 | 5874 |
| 592 | 712 | ritodrine | 12 µM | PC3 | 0.553 | 0.068 | -0.083 | 4619 |
| 593 | 623 | clorgiline | 13 µM | HL60 | 0.553 | 0.05 | -0.1 | 1604 |
| 594 | 747 | diazoxide | 17 µM | MCF7 | 0.553 | 0.06 | -0.09 | 7168 |
| 595 | 704 | paromomycin | 6 µM | PC3 | 0.553 | 0.086 | -0.064 | 4595 |
| 596 | 694 | pepstatin | 6 µM | MCF7 | 0.553 | 0.049 | -0.102 | 4790 |
| 597 | 672 | paromomycin | 6 µM | MCF7 | 0.553 | 0.053 | -0.097 | 3356 |
| 598 | 752 | cefmetazole | 8 µM | MCF7 | 0.553 | 0.053 | -0.098 | 6086 |
| 599 | 736 | remoxipride | 10 µM | MCF7 | 0.552 | 0.044 | -0.106 | 5443 |
| 600 | 741 | triamterene | 16 µM | MCF7 | 0.552 | 0.068 | -0.082 | 6010 |
| 601 | 744 | clidinium bromide | 9 µM | MCF7 | 0.552 | 0.05 | -0.1 | 6837 |
| 602 | 745 | tetracycline | 8 µM | MCF7 | 0.552 | 0.077 | -0.073 | 6233 |
| 603 | 603 | haloperidol | 10 µM | PC3 | 0.552 | 0.106 | -0.045 | 1244 |
| 604 | 648 | nadide | 6 µM | HL60 | 0.551 | 0.045 | -0.105 | 2529 |
| 605 | 514 | 3-hydroxy-DL-kynurenine | 9 µM | MCF7 | 0.551 | 0.085 | -0.065 | 1109 |
| 606 | 700 | meclofenoxate | 14 µM | MCF7 | 0.551 | 0.089 | -0.06 | 4729 |
| 607 | 1088 | pioglitazone | 10 µM | MCF7 | 0.551 | 0.09 | -0.06 | 7523 |
| 608 | 772 | dexpanthenol | 19 µM | MCF7 | 0.551 | 0.1 | -0.05 | 7455 |
| 609 | 626 | geldanamycin | 1 µM | MCF7 | 0.551 | 0.085 | -0.065 | 1653 |
| 610 | 672 | nadolol | 13 µM | MCF7 | 0.551 | 0.073 | -0.077 | 3359 |
| 611 | 762 | chenodeoxycholic acid | 10 µM | PC3 | 0.55 | 0.077 | -0.072 | 7310 |
| 612 | 615 | thalidomide | 15 µM | HL60 | 0.55 | 0.048 | -0.102 | 1411 |
| 613 | 676 | harpagoside | 8 µM | MCF7 | 0.548 | 0.075 | -0.074 | 7355 |
| 614 | 692 | idoxuridine | 11 µM | PC3 | 0.547 | 0.093 | -0.056 | 4200 |
| 615 | 662 | calycanthine | 12 µM | MCF7 | 0.547 | 0.06 | -0.089 | 2764 |
| 616 | 751 | karakoline | 11 µM | MCF7 | 0.547 | 0.06 | -0.089 | 6059 |
| 617 | 718 | allantoin | 25 µM | PC3 | 0.546 | 0.095 | -0.054 | 5052 |
| 618 | 1033 | 5155877 | 10 µM | MCF7 | 0.546 | 0.064 | -0.084 | 6549 |
| 619 | 1073 | PHA-00816795 | 10 µM | PC3 | 0.545 | 0.065 | -0.083 | 7076 |
| 620 | 729 | glycocholic acid | 9 µM | MCF7 | 0.545 | 0.089 | -0.059 | 5316 |
| 621 | 613 | lynestrenol | 14 µM | HL60 | 0.544 | 0.043 | -0.105 | 2037 |
| 622 | 741 | tranylcypromine | 24 µM | MCF7 | 0.544 | 0.07 | -0.077 | 5996 |
| 623 | 747 | strophanthidin | 10 µM | MCF7 | 0.544 | 0.077 | -0.071 | 7182 |
| 624 | 726 | digoxigenin | 10 µM | MCF7 | 0.544 | 0.063 | -0.085 | 5275 |
| 625 | 681 | sulfamerazine | 15 µM | PC3 | 0.544 | 0.076 | -0.071 | 3718 |
| 626 | 1086 | IC-86621 | 1 µM | MCF7 | 0.543 | 0.054 | -0.094 | 7513 |
| 627 | 726 | haloperidol | 11 µM | MCF7 | 0.543 | 0.06 | -0.088 | 5273 |
| 628 | 656 | pyrazinamide | 32 µM | MCF7 | 0.543 | 0.048 | -0.1 | 2839 |
| 629 | 687 | quinethazone | 14 µM | MCF7 | 0.543 | 0.062 | -0.086 | 3875 |
| 630 | 764 | oxedrine | 24 µM | PC3 | 0.543 | 0.085 | -0.062 | 7150 |
| 631 | 725 | troglitazone | 10 µM | MCF7 | 0.542 | 0.078 | -0.07 | 5229 |
| 632 | 709 | theobromine | 22 µM | PC3 | 0.542 | 0.068 | -0.08 | 6613 |
| 633 | 637 | cloxacillin | 9 µM | MCF7 | 0.542 | 0.054 | -0.093 | 2289 |
| 634 | 728 | diperodon | 9 µM | PC3 | 0.541 | 0.057 | -0.09 | 4498 |
| 635 | 658 | cefsulodin | 7 µM | HL60 | 0.541 | 0.047 | -0.1 | 2988 |
| 636 | 637 | flufenamic acid | 14 µM | MCF7 | 0.541 | 0.052 | -0.095 | 2267 |
| 637 | 712 | crotamiton | 20 µM | PC3 | 0.541 | 0.063 | -0.084 | 4628 |
| 638 | 1043 | STOCK1N-35696 | 15 µM | MCF7 | 0.54 | 0.06 | -0.086 | 6577 |
| 639 | 505 | 5162773 | 7 µM | MCF7 | 0.54 | 0.074 | -0.073 | 892 |
| 640 | 725 | tanespimycin | 1 µM | MCF7 | 0.54 | 0.06 | -0.087 | 5223 |
| 641 | 741 | nipecotic acid | 31 µM | MCF7 | 0.54 | 0.074 | -0.073 | 5999 |
| 642 | 725 | genistein | 10 µM | MCF7 | 0.54 | 0.076 | -0.07 | 5232 |
| 643 | 745 | dienestrol | 15 µM | MCF7 | 0.539 | 0.092 | -0.055 | 6208 |
| 644 | 637 | oxolinic acid | 15 µM | MCF7 | 0.539 | 0.094 | -0.052 | 2266 |
| 645 | 713 | clenbuterol | 13 µM | PC3 | 0.539 | 0.076 | -0.07 | 4671 |
| 646 | 650 | tanespimycin | 1 µM | HL60 | 0.538 | 0.078 | -0.068 | 2666 |
| 647 | 767 | valproic acid | 50 µM | MCF7 | 0.538 | 0.078 | -0.068 | 6941 |
| 648 | 732 | mephenytoin | 18 µM | PC3 | 0.538 | 0.069 | -0.077 | 5801 |
| 649 | 656 | rifampicin | 5 µM | MCF7 | 0.537 | 0.069 | -0.077 | 2847 |
| 650 | 622 | mepyramine | 10 µM | HL60 | 0.537 | 0.06 | -0.086 | 1568 |
| 651 | 753 | vigabatrin | 31 µM | PC3 | 0.537 | 0.063 | -0.083 | 6314 |
| 652 | 58 | copper sulfate | 100 µM | MCF7 | 0.536 | 0.099 | -0.046 | 438 |
| 653 | 692 | hydroxyachillin | 14 µM | PC3 | 0.536 | 0.061 | -0.085 | 4213 |
| 654 | 670 | flumetasone | 10 µM | MCF7 | 0.536 | 0.062 | -0.084 | 3410 |
| 655 | 678 | meropenem | 10 µM | MCF7 | 0.535 | 0.068 | -0.078 | 3564 |
| 656 | 713 | 3-hydroxy-DL-kynurenine | 18 µM | PC3 | 0.535 | 0.056 | -0.089 | 4681 |
| 657 | 610 | acebutolol | 11 µM | PC3 | 0.535 | 0.091 | -0.054 | 1911 |
| 658 | 688 | pheniramine | 11 µM | PC3 | 0.534 | 0.089 | -0.056 | 4012 |
| 659 | 762 | triamterene | 16 µM | PC3 | 0.534 | 0.068 | -0.077 | 7307 |
| 660 | 743 | cefapirin | 9 µM | MCF7 | 0.534 | 0.089 | -0.056 | 6790 |
| 661 | 756 | flucloxacillin | 8 µM | MCF7 | 0.534 | 0.06 | -0.085 | 6507 |
| 662 | 714 | phenylpropanolamine | 21 µM | PC3 | 0.533 | 0.065 | -0.08 | 6699 |
| 663 | 691 | sitosterol | 10 µM | MCF7 | 0.532 | 0.075 | -0.07 | 4154 |
| 664 | 628 | adiphenine | 11 µM | PC3 | 0.532 | 0.068 | -0.076 | 1831 |
| 665 | 640 | clorsulon | 11 µM | HL60 | 0.531 | 0.064 | -0.08 | 1735 |
| 666 | 22a | quercetin | 1 µM | MCF7 | 0.531 | 0.055 | -0.09 | 283 |
| 667 | 714 | benserazide | 14 µM | PC3 | 0.531 | 0.042 | -0.102 | 6722 |
| 668 | 729 | benserazide | 14 µM | MCF7 | 0.53 | 0.082 | -0.062 | 5322 |
| 669 | 743 | mebeverine | 9 µM | MCF7 | 0.53 | 0.061 | -0.083 | 6795 |
| 670 | 693 | tocainide | 17 µM | PC3 | 0.53 | 0.067 | -0.077 | 4256 |
| 671 | 670 | flecainide | 8 µM | MCF7 | 0.53 | 0.081 | -0.063 | 3418 |
| 672 | 726 | enoxacin | 12 µM | MCF7 | 0.529 | 0.07 | -0.074 | 5251 |
| 673 | 686 | pempidine | 13 µM | MCF7 | 0.529 | 0.084 | -0.06 | 3832 |
| 674 | 1061 | trichostatin A | 1 µM | MCF7 | 0.529 | 0.112 | -0.032 | 6916 |
| 675 | 614 | atropine oxide | 12 µM | HL60 | 0.529 | 0.074 | -0.07 | 1370 |
| 676 | 757 | valproic acid | 500 µM | MCF7 | 0.528 | 0.066 | -0.077 | 5600 |
| 677 | 715 | hesperetin | 13 µM | PC3 | 0.528 | 0.084 | -0.059 | 6750 |
| 678 | 654 | pepstatin | 6 µM | MCF7 | 0.527 | 0.054 | -0.09 | 3264 |
| 679 | 644 | trihexyphenidyl | 12 µM | HL60 | 0.527 | 0.061 | -0.083 | 2158 |
| 680 | 772 | mefexamide | 13 µM | MCF7 | 0.527 | 0.082 | -0.062 | 7478 |
| 681 | 706 | hexamethonium bromide | 10 µM | MCF7 | 0.527 | 0.082 | -0.061 | 4965 |
| 682 | 703 | ethotoin | 20 µM | PC3 | 0.526 | 0.067 | -0.076 | 4545 |
| 683 | 689 | trimethobenzamide | 9 µM | PC3 | 0.526 | 0.046 | -0.097 | 4100 |
| 684 | 690 | cinchonine | 14 µM | MCF7 | 0.526 | 0.056 | -0.087 | 4107 |
| 685 | 757 | alvespimycin | 100 nM | MCF7 | 0.526 | 0.079 | -0.064 | 5573 |
| 686 | 771 | acemetacin | 10 µM | MCF7 | 0.525 | 0.07 | -0.073 | 7442 |
| 687 | 766 | disopyramide | 12 µM | MCF7 | 0.525 | 0.066 | -0.077 | 7035 |
| 688 | 681 | metoclopramide | 12 µM | PC3 | 0.525 | 0.061 | -0.081 | 3728 |
| 689 | 753 | prazosin | 10 µM | PC3 | 0.523 | 0.091 | -0.052 | 6315 |
| 690 | 771 | econazole | 9 µM | MCF7 | 0.523 | 0.052 | -0.09 | 7427 |
| 691 | 772 | Prestwick-983 | 17 µM | MCF7 | 0.523 | 0.087 | -0.056 | 7480 |
| 692 | 754 | tolfenamic acid | 15 µM | PC3 | 0.523 | 0.066 | -0.076 | 6354 |
| 693 | 709 | harmaline | 14 µM | PC3 | 0.523 | 0.09 | -0.052 | 6623 |
| 694 | 646 | methylprednisolone | 11 µM | MCF7 | 0.522 | 0.068 | -0.073 | 3183 |
| 695 | 626 | fluphenazine | 10 µM | MCF7 | 0.521 | 0.075 | -0.067 | 1662 |
| 696 | 731 | cytisine | 21 µM | PC3 | 0.521 | 0.066 | -0.075 | 5739 |
| 697 | 725 | tanespimycin | 1 µM | MCF7 | 0.521 | 0.083 | -0.059 | 5203 |
| 698 | 704 | piromidic acid | 14 µM | PC3 | 0.521 | 0.04 | -0.102 | 4575 |
| 699 | 733 | dexpropranolol | 14 µM | PC3 | 0.52 | 0.055 | -0.087 | 5814 |
| 700 | 671 | dienestrol | 15 µM | MCF7 | 0.52 | 0.088 | -0.054 | 3448 |
| 701 | 656 | tolazamide | 13 µM | MCF7 | 0.52 | 0.05 | -0.091 | 2842 |
| 702 | 627 | imipramine | 13 µM | MCF7 | 0.519 | 0.069 | -0.072 | 1685 |
| 703 | 673 | esculin | 12 µM | MCF7 | 0.519 | 0.072 | -0.069 | 3390 |
| 704 | 646 | tremorine | 15 µM | MCF7 | 0.519 | 0.08 | -0.061 | 3196 |
| 705 | 654 | SR-95531 | 11 µM | MCF7 | 0.519 | 0.058 | -0.083 | 3253 |
| 706 | 1087 | 11-deoxy-16,16-dimethylprostaglandin E2 | 10 µM | PC3 | 0.519 | 0.04 | -0.101 | 7519 |
| 707 | 94 | bucladesine | 20 µM | MCF7 | 0.519 | 0.09 | -0.051 | 591 |
| 708 | 505 | quercetin | 1 µM | MCF7 | 0.518 | 0.069 | -0.071 | 917 |
| 709 | 766 | dyclonine | 12 µM | MCF7 | 0.518 | 0.078 | -0.063 | 7022 |
| 710 | 712 | androsterone | 14 µM | PC3 | 0.518 | 0.09 | -0.051 | 4635 |
| 711 | 731 | cromoglicic acid | 8 µM | PC3 | 0.517 | 0.054 | -0.087 | 5754 |
| 712 | 662 | pyridoxine | 19 µM | MCF7 | 0.517 | 0.081 | -0.06 | 2751 |
| 713 | 726 | lorglumide | 8 µM | MCF7 | 0.515 | 0.079 | -0.061 | 5254 |
| 714 | 733 | sulfamonomethoxine | 14 µM | PC3 | 0.515 | 0.074 | -0.066 | 5843 |
| 715 | 632 | cinchocaine | 12 µM | MCF7 | 0.515 | 0.059 | -0.081 | 1469 |
| 716 | 705 | N-acetylmuramic acid | 14 µM | MCF7 | 0.515 | 0.075 | -0.065 | 4406 |
| 717 | 648 | abamectin | 5 µM | HL60 | 0.514 | 0.054 | -0.085 | 2519 |
| 718 | 688 | memantine | 19 µM | PC3 | 0.514 | 0.053 | -0.087 | 4017 |
| 719 | 762 | pentoxifylline | 14 µM | PC3 | 0.513 | 0.089 | -0.05 | 7319 |
| 720 | 677 | prednicarbate | 8 µM | MCF7 | 0.513 | 0.049 | -0.09 | 3542 |
| 721 | 745 | trichostatin A | 100 nM | MCF7 | 0.513 | 0.088 | -0.052 | 6222 |
| 722 | 1025 | 3-nitropropionic acid | 10 µM | PC3 | 0.513 | 0.045 | -0.095 | 6407 |
| 723 | 671 | cinoxacin | 15 µM | MCF7 | 0.513 | 0.062 | -0.078 | 3463 |
| 724 | 743 | anabasine | 25 µM | MCF7 | 0.512 | 0.058 | -0.082 | 6774 |
| 725 | 632 | sulpiride | 12 µM | MCF7 | 0.512 | 0.083 | -0.056 | 1467 |
| 726 | 767 | LY-294002 | 100 nM | MCF7 | 0.512 | 0.041 | -0.098 | 6935 |
| 727 | 729 | methylergometrine | 9 µM | MCF7 | 0.512 | 0.062 | -0.077 | 5303 |
| 728 | 764 | chlortalidone | 12 µM | PC3 | 0.512 | 0.068 | -0.071 | 7152 |
| 729 | 712 | guanabenz | 14 µM | PC3 | 0.511 | 0.06 | -0.079 | 4642 |
| 730 | 732 | alpha-yohimbine | 10 µM | PC3 | 0.511 | 0.073 | -0.066 | 5800 |
| 731 | 646 | mebeverine | 9 µM | MCF7 | 0.511 | 0.06 | -0.079 | 3193 |
| 732 | 731 | calycanthine | 12 µM | PC3 | 0.511 | 0.095 | -0.044 | 5744 |
| 733 | 691 | furazolidone | 18 µM | MCF7 | 0.51 | 0.053 | -0.086 | 4178 |
| 734 | 618 | fenbendazole | 13 µM | HL60 | 0.51 | 0.057 | -0.082 | 2360 |
| 735 | 1074 | pioglitazone | 10 µM | MCF7 | 0.51 | 0.059 | -0.079 | 7083 |
| 736 | 633 | glipizide | 9 µM | MCF7 | 0.509 | 0.043 | -0.096 | 1508 |
| 737 | 641 | cytisine | 21 µM | HL60 | 0.509 | 0.041 | -0.098 | 1766 |
| 738 | 626 | alpha-estradiol | 10 nM | MCF7 | 0.509 | 0.077 | -0.061 | 1635 |
| 739 | 754 | benzonatate | 7 µM | PC3 | 0.508 | 0.071 | -0.067 | 6334 |
| 740 | 737 | loracarbef | 11 µM | MCF7 | 0.508 | 0.049 | -0.089 | 5492 |
| 741 | 726 | methyldopa | 19 µM | MCF7 | 0.507 | 0.078 | -0.06 | 5272 |
| 742 | 725 | clozapine | 10 µM | MCF7 | 0.507 | 0.045 | -0.092 | 5226 |
| 743 | 687 | chlorambucil | 13 µM | MCF7 | 0.506 | 0.066 | -0.071 | 3869 |
| 744 | 682 | piperacillin | 7 µM | PC3 | 0.506 | 0.079 | -0.058 | 3763 |
| 745 | 758 | haloperidol | 11 µM | MCF7 | 0.506 | 0.049 | -0.088 | 5638 |
| 746 | 654 | SR-95639A | 10 µM | MCF7 | 0.505 | 0.059 | -0.078 | 3272 |
| 747 | 678 | Prestwick-1084 | 16 µM | MCF7 | 0.505 | 0.078 | -0.059 | 3546 |
| 748 | 747 | pyridoxine | 19 µM | MCF7 | 0.505 | 0.064 | -0.073 | 7171 |
| 749 | 715 | erythromycin | 5 µM | PC3 | 0.505 | 0.057 | -0.08 | 6729 |
| 750 | 713 | pirenperone | 10 µM | PC3 | 0.505 | 0.043 | -0.095 | 4679 |
| 751 | 61 | TTNPB | 100 nM | PC3 | 0.505 | 0.071 | -0.066 | 451 |
| 752 | 744 | diperodon | 9 µM | MCF7 | 0.505 | 0.06 | -0.078 | 6836 |
| 753 | 1089 | 15(S)-15-methylprostaglandin E2 | 10 µM | PC3 | 0.504 | 0.071 | -0.066 | 7526 |
| 754 | 628 | dapsone | 16 µM | PC3 | 0.504 | 0.064 | -0.073 | 1827 |
| 755 | 703 | Prestwick-1080 | 15 µM | PC3 | 0.504 | 0.077 | -0.06 | 4532 |
| 756 | 708 | tyloxapol | 4 µM | MCF7 | 0.503 | 0.081 | -0.056 | 5672 |
| 757 | 764 | cefapirin | 9 µM | PC3 | 0.503 | 0.051 | -0.086 | 7142 |
| 758 | 632 | dicycloverine | 12 µM | MCF7 | 0.503 | 0.082 | -0.055 | 1483 |
| 759 | 653 | cefalexin | 11 µM | MCF7 | 0.503 | 0.077 | -0.059 | 2628 |
| 760 | 40 | tanespimycin | 1 µM | MCF7 | 0.502 | 0.063 | -0.074 | 381 |
| 761 | 662 | folic acid | 9 µM | MCF7 | 0.502 | 0.07 | -0.066 | 2783 |
| 762 | 634 | pilocarpine | 15 µM | HL60 | 0.502 | 0.064 | -0.072 | 2438 |
| 763 | 726 | biperiden | 11 µM | MCF7 | 0.501 | 0.06 | -0.077 | 5279 |
| 764 | 650 | sirolimus | 100 nM | HL60 | 0.501 | 0.052 | -0.084 | 2667 |
| 765 | 754 | procaine | 15 µM | PC3 | 0.501 | 0.064 | -0.072 | 6329 |
| 766 | 730 | hesperetin | 13 µM | MCF7 | 0.501 | 0.073 | -0.063 | 5350 |
| 767 | 736 | iopanoic acid | 7 µM | MCF7 | 0.501 | 0.047 | -0.089 | 5448 |
| 768 | 636 | triamcinolone | 10 µM | MCF7 | 0.501 | 0.058 | -0.078 | 2241 |
| 769 | 699 | fludroxycortide | 9 µM | MCF7 | 0.5 | 0.063 | -0.073 | 4702 |
| 770 | 677 | rimexolone | 11 µM | MCF7 | 0.499 | 0.074 | -0.061 | 3516 |
| 771 | 735 | melatonin | 17 µM | MCF7 | 0.499 | 0.06 | -0.075 | 5393 |
| 772 | 17 | TTNPB | 100 nM | MCF7 | 0.499 | 0.081 | -0.055 | 223 |
| 773 | 695 | orciprenaline | 8 µM | MCF7 | 0.498 | 0.048 | -0.088 | 4831 |
| 774 | 656 | vancomycin | 3 µM | MCF7 | 0.498 | 0.063 | -0.072 | 2858 |
| 775 | 706 | lactobionic acid | 11 µM | MCF7 | 0.497 | 0.068 | -0.067 | 4950 |
| 776 | 646 | lidoflazine | 8 µM | MCF7 | 0.497 | 0.05 | -0.085 | 3201 |
| 777 | 1062 | fulvestrant | 1 µM | PC3 | 0.497 | 0.082 | -0.053 | 6918 |
| 778 | 749 | meprylcaine | 15 µM | HL60 | 0.496 | 0.043 | -0.092 | 6123 |
| 779 | 1055 | NS-398 | 10 µM | MCF7 | 0.496 | 0.051 | -0.084 | 6897 |
| 780 | 670 | merbromin | 5 µM | MCF7 | 0.496 | 0.054 | -0.081 | 3439 |
| 781 | 636 | disulfiram | 13 µM | MCF7 | 0.496 | 0.07 | -0.065 | 2215 |
| 782 | 676 | memantine | 19 µM | MCF7 | 0.496 | 0.056 | -0.079 | 7354 |
| 783 | 687 | PHA-00745360 | 10 µM | MCF7 | 0.495 | 0.056 | -0.079 | 3907 |
| 784 | 689 | sotalol | 13 µM | PC3 | 0.495 | 0.053 | -0.082 | 4079 |
| 785 | 754 | alverine | 8 µM | PC3 | 0.495 | 0.054 | -0.08 | 6345 |
| 786 | 617 | metampicillin | 10 µM | PC3 | 0.495 | 0.073 | -0.062 | 2123 |
| 787 | 714 | apomorphine | 6 µM | PC3 | 0.495 | 0.071 | -0.064 | 6683 |
| 788 | 654 | cyanocobalamin | 3 µM | MCF7 | 0.494 | 0.078 | -0.056 | 3252 |
| 789 | 745 | dizocilpine | 12 µM | MCF7 | 0.494 | 0.065 | -0.069 | 6223 |
| 790 | 693 | salsolinol | 15 µM | PC3 | 0.494 | 0.065 | -0.069 | 4232 |
| 791 | 713 | testosterone | 12 µM | PC3 | 0.494 | 0.052 | -0.082 | 4676 |
| 792 | 672 | levobunolol | 12 µM | MCF7 | 0.494 | 0.054 | -0.081 | 3354 |
| 793 | 95 | arachidonyltrifluoromethane | 10 µM | MCF7 | 0.493 | 0.057 | -0.077 | 594 |
| 794 | 672 | mestranol | 13 µM | MCF7 | 0.493 | 0.06 | -0.074 | 3346 |
| 795 | 691 | carbenoxolone | 7 µM | MCF7 | 0.493 | 0.081 | -0.053 | 4173 |
| 796 | 663 | protoveratrine A | 5 µM | MCF7 | 0.493 | 0.071 | -0.063 | 2800 |
| 797 | 708 | eticlopride | 11 µM | MCF7 | 0.492 | 0.043 | -0.091 | 5695 |
| 798 | 628 | triflupromazine | 10 µM | PC3 | 0.492 | 0.089 | -0.045 | 1813 |
| 799 | 1050 | H-89 | 500 nM | PC3 | 0.492 | 0.042 | -0.092 | 6873 |
| 800 | 704 | beclometasone | 8 µM | PC3 | 0.491 | 0.048 | -0.085 | 4580 |
| 801 | 655 | velnacrine | 12 µM | MCF7 | 0.491 | 0.047 | -0.086 | 3292 |
| 802 | 731 | etanidazole | 19 µM | PC3 | 0.49 | 0.052 | -0.081 | 5730 |
| 803 | 718 | harmalol | 15 µM | PC3 | 0.489 | 0.063 | -0.07 | 5076 |
| 804 | 761 | viomycin | 5 µM | PC3 | 0.489 | 0.061 | -0.072 | 7278 |
| 805 | 628 | isoflupredone | 10 µM | PC3 | 0.489 | 0.046 | -0.086 | 1832 |
| 806 | 715 | idazoxan | 17 µM | PC3 | 0.489 | 0.078 | -0.055 | 6747 |
| 807 | 1017 | erastin | 20 µM | PC3 | 0.488 | 0.068 | -0.065 | 6369 |
| 808 | 743 | midodrine | 14 µM | MCF7 | 0.488 | 0.057 | -0.076 | 6804 |
| 809 | 758 | 3-hydroxy-DL-kynurenine | 18 µM | MCF7 | 0.488 | 0.064 | -0.069 | 5641 |
| 810 | 1093 | IC-86621 | 1 µM | PC3 | 0.487 | 0.064 | -0.068 | 7548 |
| 811 | 711 | helveticoside | 7 µM | MCF7 | 0.487 | 0.045 | -0.088 | 3945 |
| 812 | 730 | ergocalciferol | 10 µM | MCF7 | 0.487 | 0.059 | -0.074 | 5346 |
| 813 | 690 | fusaric acid | 22 µM | MCF7 | 0.487 | 0.069 | -0.064 | 4105 |
| 814 | 670 | cefamandole | 8 µM | MCF7 | 0.487 | 0.058 | -0.074 | 3436 |
| 815 | 505 | 5151277 | 14 µM | MCF7 | 0.487 | 0.077 | -0.055 | 903 |
| 816 | 65 | pirinixic acid | 100 µM | PC3 | 0.486 | 0.077 | -0.056 | 464 |
| 817 | 728 | epivincamine | 11 µM | PC3 | 0.486 | 0.05 | -0.082 | 4500 |
| 818 | 730 | N6-methyladenosine | 14 µM | MCF7 | 0.486 | 0.075 | -0.057 | 5332 |
| 819 | 764 | propoxycaine | 12 µM | PC3 | 0.486 | 0.059 | -0.073 | 7155 |
| 820 | 755 | terguride | 12 µM | MCF7 | 0.485 | 0.062 | -0.07 | 6459 |
| 821 | 640 | ajmaline | 12 µM | HL60 | 0.485 | 0.063 | -0.068 | 1749 |
| 822 | 1036 | STOCK1N-35874 | 14 µM | PC3 | 0.485 | 0.044 | -0.088 | 6561 |
| 823 | 756 | isradipine | 11 µM | MCF7 | 0.485 | 0.066 | -0.066 | 6508 |
| 824 | 677 | Prestwick-1082 | 12 µM | MCF7 | 0.485 | 0.064 | -0.067 | 3530 |
| 825 | 613 | metolazone | 11 µM | HL60 | 0.484 | 0.043 | -0.089 | 2014 |
| 826 | 87 | HNMPA-(AM)3 | 5 µM | PC3 | 0.484 | 0.092 | -0.039 | 583 |
| 827 | 743 | carbinoxamine | 10 µM | MCF7 | 0.483 | 0.066 | -0.065 | 6786 |
| 828 | 715 | anisomycin | 15 µM | PC3 | 0.483 | 0.065 | -0.067 | 6764 |
| 829 | 707 | ciclopirox | 15 µM | MCF7 | 0.483 | 0.091 | -0.04 | 5023 |
| 830 | 656 | tetramisole | 17 µM | MCF7 | 0.482 | 0.062 | -0.069 | 2849 |
| 831 | 701 | karakoline | 11 µM | PC3 | 0.482 | 0.063 | -0.068 | 4297 |
| 832 | 691 | carteolol | 12 µM | MCF7 | 0.482 | 0.046 | -0.085 | 4176 |
| 833 | 636 | astemizole | 9 µM | MCF7 | 0.482 | 0.066 | -0.065 | 2211 |
| 834 | 603 | alpha-estradiol | 10 nM | PC3 | 0.481 | 0.058 | -0.073 | 1210 |
| 835 | 709 | oxolamine | 9 µM | PC3 | 0.479 | 0.075 | -0.056 | 6624 |
| 836 | 616 | dexamethasone | 9 µM | PC3 | 0.479 | 0.071 | -0.059 | 2079 |
| 837 | 1059 | NS-398 | 10 µM | MCF7 | 0.478 | 0.076 | -0.054 | 6911 |
| 838 | 714 | ambroxol | 10 µM | PC3 | 0.478 | 0.047 | -0.083 | 6719 |
| 839 | 753 | methotrexate | 9 µM | PC3 | 0.478 | 0.056 | -0.074 | 6318 |
| 840 | 1078 | 6-bromoindirubin-3'-oxime | 500 nM | MCF7 | 0.478 | 0.048 | -0.082 | 7101 |
| 841 | 1078 | 0198306-0000 | 10 µM | MCF7 | 0.476 | 0.048 | -0.082 | 7099 |
| 842 | 757 | tanespimycin | 1 µM | MCF7 | 0.476 | 0.055 | -0.074 | 5585 |
| 843 | 733 | nalbuphine | 10 µM | PC3 | 0.476 | 0.059 | -0.07 | 5820 |
| 844 | 713 | pentetic acid | 10 µM | PC3 | 0.476 | 0.052 | -0.078 | 4669 |
| 845 | 613 | loxapine | 9 µM | HL60 | 0.475 | 0.063 | -0.066 | 2016 |
| 846 | 702 | sulfadimidine | 13 µM | PC3 | 0.475 | 0.061 | -0.068 | 4322 |
| 847 | 681 | isosorbide | 17 µM | PC3 | 0.475 | 0.078 | -0.051 | 3720 |
| 848 | 692 | amantadine | 10 µM | PC3 | 0.474 | 0.044 | -0.085 | 4222 |
| 849 | 704 | bicuculline | 11 µM | PC3 | 0.474 | 0.054 | -0.074 | 4574 |
| 850 | 670 | eucatropine | 12 µM | MCF7 | 0.474 | 0.08 | -0.048 | 3416 |
| 851 | 628 | imipramine | 13 µM | PC3 | 0.474 | 0.068 | -0.061 | 1807 |
| 852 | 33 | valproic acid | 2 mM | MCF7 | 0.473 | 0.082 | -0.047 | 346 |
| 853 | 616 | nalbuphine | 10 µM | PC3 | 0.473 | 0.084 | -0.045 | 2063 |
| 854 | 709 | omeprazole | 12 µM | PC3 | 0.473 | 0.06 | -0.069 | 6606 |
| 855 | 690 | boldine | 12 µM | MCF7 | 0.473 | 0.061 | -0.068 | 4122 |
| 856 | 616 | diazoxide | 17 µM | PC3 | 0.472 | 0.073 | -0.056 | 2052 |
| 857 | 626 | estradiol | 100 nM | MCF7 | 0.472 | 0.075 | -0.054 | 1633 |
| 858 | 678 | oxprenolol | 13 µM | MCF7 | 0.472 | 0.084 | -0.044 | 3568 |
| 859 | 704 | naringenin | 15 µM | PC3 | 0.471 | 0.062 | -0.066 | 4597 |
| 860 | 747 | cinnarizine | 11 µM | MCF7 | 0.471 | 0.08 | -0.048 | 7174 |
| 861 | 627 | adiphenine | 11 µM | MCF7 | 0.47 | 0.067 | -0.061 | 1709 |
| 862 | 657 | harmol | 16 µM | MCF7 | 0.47 | 0.04 | -0.087 | 2900 |
| 863 | 688 | tolazamide | 13 µM | PC3 | 0.469 | 0.065 | -0.062 | 4003 |
| 864 | 729 | amikacin | 7 µM | MCF7 | 0.469 | 0.052 | -0.076 | 5314 |
| 865 | 1067 | colforsin | 500 nM | PC3 | 0.469 | 0.054 | -0.073 | 7059 |
| 866 | 738 | rilmenidine | 8 µM | MCF7 | 0.469 | 0.053 | -0.074 | 5532 |
| 867 | 766 | pergolide | 10 µM | MCF7 | 0.469 | 0.066 | -0.062 | 7031 |
| 868 | 705 | etamsylate | 15 µM | MCF7 | 0.468 | 0.064 | -0.064 | 4399 |
| 869 | 729 | diclofenamide | 13 µM | MCF7 | 0.468 | 0.076 | -0.052 | 5286 |
| 870 | 733 | diazoxide | 17 µM | PC3 | 0.468 | 0.047 | -0.08 | 5810 |
| 871 | 659 | propranolol | 14 µM | HL60 | 0.467 | 0.065 | -0.062 | 3059 |
| 872 | 680 | cefamandole | 8 µM | PC3 | 0.467 | 0.052 | -0.076 | 3696 |
| 873 | 689 | alprostadil | 11 µM | PC3 | 0.467 | 0.062 | -0.065 | 4099 |
| 874 | 603 | tanespimycin | 1 µM | PC3 | 0.467 | 0.068 | -0.059 | 1226 |
| 875 | 1088 | CP-645525-01 | 10 µM | MCF7 | 0.467 | 0.066 | -0.061 | 7522 |
| 876 | 616 | spironolactone | 10 µM | PC3 | 0.467 | 0.08 | -0.047 | 2064 |
| 877 | 616 | nicergoline | 8 µM | PC3 | 0.466 | 0.074 | -0.053 | 2058 |
| 878 | 637 | nimesulide | 13 µM | MCF7 | 0.466 | 0.062 | -0.065 | 2275 |
| 879 | 743 | dipivefrine | 10 µM | MCF7 | 0.466 | 0.072 | -0.055 | 6766 |
| 880 | 611 | metanephrine | 17 µM | PC3 | 0.465 | 0.074 | -0.052 | 1933 |
| 881 | 633 | guanabenz | 14 µM | MCF7 | 0.464 | 0.063 | -0.063 | 1544 |
| 882 | 731 | (+)-chelidonine | 11 µM | PC3 | 0.463 | 0.05 | -0.076 | 5760 |
| 883 | 677 | viomycin | 5 µM | MCF7 | 0.463 | 0.045 | -0.081 | 3541 |
| 884 | 766 | hydroflumethiazide | 12 µM | MCF7 | 0.463 | 0.067 | -0.059 | 7019 |
| 885 | 719 | adipiodone | 4 µM | PC3 | 0.463 | 0.048 | -0.078 | 5085 |
| 886 | 718 | pizotifen | 9 µM | PC3 | 0.462 | 0.082 | -0.044 | 5072 |
| 887 | 1071 | CAY-10397 | 10 µM | PC3 | 0.462 | 0.063 | -0.063 | 7071 |
| 888 | 686 | famprofazone | 11 µM | MCF7 | 0.462 | 0.04 | -0.085 | 3834 |
| 889 | 70 | copper sulfate | 100 µM | ssMCF7 | 0.46 | 0.058 | -0.067 | 500 |
| 890 | 1033 | dinoprostone | 10 µM | MCF7 | 0.459 | 0.057 | -0.068 | 6552 |
| 891 | 712 | iohexol | 5 µM | PC3 | 0.459 | 0.066 | -0.059 | 4643 |
| 892 | 626 | tanespimycin | 1 µM | MCF7 | 0.459 | 0.064 | -0.061 | 1651 |
| 893 | 636 | acetylsalicylsalicylic acid | 13 µM | MCF7 | 0.458 | 0.066 | -0.059 | 2223 |
| 894 | 725 | fluphenazine | 10 µM | MCF7 | 0.458 | 0.054 | -0.071 | 5234 |
| 895 | 727 | estradiol | 10 nM | PC3 | 0.457 | 0.061 | -0.063 | 4465 |
| 896 | 611 | ciprofloxacin | 11 µM | PC3 | 0.457 | 0.082 | -0.042 | 1939 |
| 897 | 682 | flecainide | 8 µM | PC3 | 0.456 | 0.053 | -0.071 | 3761 |
| 898 | 636 | isoniazid | 29 µM | MCF7 | 0.456 | 0.069 | -0.055 | 2246 |
| 899 | 610 | tolnaftate | 13 µM | PC3 | 0.456 | 0.082 | -0.042 | 1919 |
| 900 | 706 | sulfabenzamide | 14 µM | MCF7 | 0.456 | 0.057 | -0.067 | 4979 |
| 901 | 626 | prochlorperazine | 10 µM | MCF7 | 0.455 | 0.07 | -0.054 | 1640 |
| 902 | 628 | diphenhydramine | 14 µM | PC3 | 0.455 | 0.06 | -0.064 | 1830 |
| 903 | 715 | digoxin | 5 µM | PC3 | 0.454 | 0.06 | -0.064 | 6724 |
| 904 | 54 | tanespimycin | 1 µM | MCF7 | 0.454 | 0.051 | -0.073 | 428 |
| 905 | 610 | levodopa | 20 µM | PC3 | 0.454 | 0.056 | -0.067 | 1892 |
| 906 | 754 | molsidomine | 17 µM | PC3 | 0.454 | 0.053 | -0.071 | 6325 |
| 907 | 1032 | dinoprostone | 10 µM | PC3 | 0.454 | 0.071 | -0.053 | 6547 |
| 908 | 714 | profenamine | 11 µM | PC3 | 0.453 | 0.069 | -0.054 | 6697 |
| 909 | 646 | pimozide | 9 µM | MCF7 | 0.453 | 0.07 | -0.053 | 3178 |
| 910 | 704 | terconazole | 8 µM | PC3 | 0.453 | 0.06 | -0.063 | 4583 |
| 911 | 681 | myosmine | 27 µM | PC3 | 0.453 | 0.053 | -0.07 | 3737 |
| 912 | 756 | esculetin | 22 µM | MCF7 | 0.453 | 0.066 | -0.057 | 6499 |
| 913 | 690 | metronidazole | 23 µM | MCF7 | 0.452 | 0.058 | -0.065 | 4141 |
| 914 | 678 | natamycin | 6 µM | MCF7 | 0.452 | 0.082 | -0.041 | 3548 |
| 915 | 702 | spiramycin | 5 µM | PC3 | 0.451 | 0.063 | -0.06 | 4319 |
| 916 | 733 | iopamidol | 5 µM | PC3 | 0.451 | 0.06 | -0.063 | 5832 |
| 917 | 690 | brompheniramine | 9 µM | MCF7 | 0.451 | 0.06 | -0.062 | 4131 |
| 918 | 1027 | PF-00539758-00 | 10 µM | PC3 | 0.451 | 0.053 | -0.07 | 6421 |
| 919 | 709 | benzthiazide | 9 µM | PC3 | 0.451 | 0.066 | -0.057 | 6607 |
| 920 | 692 | molindone | 13 µM | PC3 | 0.451 | 0.079 | -0.044 | 4199 |
| 921 | 733 | clindamycin | 9 µM | PC3 | 0.45 | 0.063 | -0.06 | 5815 |
| 922 | 682 | solasodine | 10 µM | PC3 | 0.45 | 0.043 | -0.079 | 3749 |
| 923 | 514 | 12,13-EODE | 200 nM | MCF7 | 0.45 | 0.074 | -0.049 | 1108 |
| 924 | 732 | nicergoline | 8 µM | PC3 | 0.449 | 0.058 | -0.064 | 5775 |
| 925 | 741 | heptaminol | 22 µM | MCF7 | 0.449 | 0.058 | -0.064 | 6015 |
| 926 | 734 | delsoline | 9 µM | PC3 | 0.449 | 0.054 | -0.068 | 5858 |
| 927 | 710 | doxazosin | 7 µM | PC3 | 0.449 | 0.048 | -0.074 | 6642 |
| 928 | 616 | picotamide | 10 µM | PC3 | 0.448 | 0.064 | -0.057 | 2070 |
| 929 | 654 | metrizamide | 5 µM | MCF7 | 0.447 | 0.073 | -0.049 | 3255 |
| 930 | 13 | sulfasalazine | 100 µM | MCF7 | 0.446 | 0.034 | -0.087 | 204 |
| 931 | 96 | arachidonic acid | 10 µM | MCF7 | 0.445 | 0.045 | -0.076 | 604 |
| 932 | 767 | tanespimycin | 1 µM | MCF7 | 0.445 | 0.042 | -0.079 | 6937 |
| 933 | 673 | ioversol | 5 µM | MCF7 | 0.444 | 0.059 | -0.061 | 3365 |
| 934 | 1025 | valdecoxib | 10 µM | PC3 | 0.444 | 0.042 | -0.079 | 6408 |
| 935 | 677 | mecamylamine | 20 µM | MCF7 | 0.444 | 0.059 | -0.062 | 3525 |
| 936 | 628 | mefenamic acid | 17 µM | PC3 | 0.444 | 0.056 | -0.065 | 1821 |
| 937 | 767 | clozapine | 10 µM | MCF7 | 0.444 | 0.038 | -0.083 | 6947 |
| 938 | 705 | sulpiride | 12 µM | MCF7 | 0.443 | 0.039 | -0.082 | 4389 |
| 939 | 746 | propidium iodide | 6 µM | MCF7 | 0.442 | 0.057 | -0.063 | 6277 |
| 940 | 690 | levobunolol | 12 µM | MCF7 | 0.442 | 0.051 | -0.069 | 4134 |
| 941 | 733 | streptozocin | 15 µM | PC3 | 0.441 | 0.058 | -0.062 | 5836 |
| 942 | 715 | chlortetracycline | 8 µM | PC3 | 0.44 | 0.053 | -0.067 | 6761 |
| 943 | 627 | allantoin | 25 µM | MCF7 | 0.44 | 0.058 | -0.061 | 1678 |
| 944 | 628 | triamterene | 16 µM | PC3 | 0.44 | 0.063 | -0.056 | 1819 |
| 945 | 753 | oxybenzone | 18 µM | PC3 | 0.44 | 0.038 | -0.082 | 6309 |
| 946 | 765 | tanespimycin | 1 µM | MCF7 | 0.439 | 0.047 | -0.072 | 6985 |
| 947 | 743 | tetryzoline | 17 µM | MCF7 | 0.439 | 0.058 | -0.062 | 6769 |
| 948 | 654 | lysergol | 16 µM | MCF7 | 0.439 | 0.054 | -0.066 | 3261 |
| 949 | 692 | calcium pantothenate | 8 µM | PC3 | 0.439 | 0.056 | -0.064 | 4189 |
| 950 | 771 | dropropizine | 17 µM | MCF7 | 0.438 | 0.05 | -0.07 | 7429 |
| 951 | 718 | isometheptene | 8 µM | PC3 | 0.438 | 0.058 | -0.061 | 5082 |
| 952 | 41 | raloxifene | 100 nM | HL60 | 0.437 | 0.064 | -0.055 | 388 |
| 953 | 729 | puromycin | 7 µM | MCF7 | 0.437 | 0.053 | -0.066 | 5310 |
| 954 | 513 | alpha-estradiol | 10 nM | MCF7 | 0.437 | 0.055 | -0.064 | 1048 |
| 955 | 694 | amantadine | 10 µM | MCF7 | 0.436 | 0.067 | -0.052 | 4806 |
| 956 | 758 | probucol | 8 µM | MCF7 | 0.436 | 0.046 | -0.073 | 5626 |
| 957 | 765 | acetylsalicylic acid | 100 µM | MCF7 | 0.436 | 0.04 | -0.078 | 6964 |
| 958 | 718 | hydrastinine | 16 µM | PC3 | 0.435 | 0.047 | -0.071 | 5075 |
| 959 | 751 | Prestwick-664 | 8 µM | MCF7 | 0.434 | 0.071 | -0.047 | 6033 |
| 960 | 637 | scopolamine N-oxide | 10 µM | MCF7 | 0.434 | 0.062 | -0.056 | 2262 |
| 961 | 771 | norethisterone | 13 µM | MCF7 | 0.434 | 0.069 | -0.049 | 7414 |
| 962 | 676 | etamsylate | 15 µM | MCF7 | 0.433 | 0.04 | -0.078 | 7335 |
| 963 | 682 | metamizole sodium | 12 µM | PC3 | 0.433 | 0.061 | -0.056 | 3754 |
| 964 | 654 | cyclobenzaprine | 13 µM | MCF7 | 0.431 | 0.059 | -0.059 | 3268 |
| 965 | 767 | valproic acid | 200 µM | MCF7 | 0.431 | 0.062 | -0.056 | 6934 |
| 966 | 502 | 5224221 | 12 µM | MCF7 | 0.431 | 0.054 | -0.063 | 956 |
| 967 | 642 | prilocaine | 16 µM | MCF7 | 0.431 | 0.051 | -0.066 | 2314 |
| 968 | 1053 | AH-23848 | 1 µM | MCF7 | 0.43 | 0.038 | -0.079 | 6890 |
| 969 | 710 | methapyrilene | 13 µM | PC3 | 0.43 | 0.062 | -0.055 | 6644 |
| 970 | 671 | sulfamonomethoxine | 14 µM | MCF7 | 0.429 | 0.055 | -0.062 | 3484 |
| 971 | 700 | fenoprofen | 7 µM | MCF7 | 0.429 | 0.072 | -0.044 | 4736 |
| 972 | 1031 | AG-013608 | 10 µM | PC3 | 0.428 | 0.049 | -0.067 | 6440 |
| 973 | 746 | methazolamide | 17 µM | MCF7 | 0.428 | 0.066 | -0.051 | 6268 |
| 974 | 730 | midecamycin | 5 µM | MCF7 | 0.428 | 0.062 | -0.054 | 5345 |
| 975 | 1033 | PHA-00767505E | 10 µM | MCF7 | 0.428 | 0.069 | -0.047 | 6550 |
| 976 | 628 | epirizole | 17 µM | PC3 | 0.427 | 0.087 | -0.029 | 1803 |
| 977 | 726 | azathioprine | 14 µM | MCF7 | 0.426 | 0.04 | -0.076 | 5262 |
| 978 | 713 | 3-acetylcoumarin | 21 µM | PC3 | 0.425 | 0.057 | -0.058 | 4664 |
| 979 | 730 | cefotiam | 7 µM | MCF7 | 0.423 | 0.054 | -0.061 | 5361 |
| 980 | 610 | isoxsuprine | 12 µM | PC3 | 0.423 | 0.081 | -0.034 | 1904 |
| 981 | 692 | naftopidil | 9 µM | PC3 | 0.422 | 0.049 | -0.066 | 4193 |
| 982 | 55 | rosiglitazone | 10 µM | PC3 | 0.422 | 0.074 | -0.041 | 430 |
| 983 | 685 | fenoprofen | 7 µM | MCF7 | 0.422 | 0.063 | -0.052 | 3612 |
| 984 | 671 | bergenin | 12 µM | MCF7 | 0.421 | 0.049 | -0.065 | 3467 |
| 985 | 752 | estropipate | 9 µM | MCF7 | 0.421 | 0.056 | -0.058 | 6068 |
| 986 | 67 | iloprost | 1 µM | MCF7 | 0.421 | 0.058 | -0.057 | 488 |
| 987 | 715 | metanephrine | 17 µM | PC3 | 0.42 | 0.05 | -0.065 | 6734 |
| 988 | 632 | 4-hydroxyphenazone | 20 µM | MCF7 | 0.419 | 0.069 | -0.045 | 1497 |
| 989 | 1071 | AR-A014418 | 10 µM | PC3 | 0.419 | 0.055 | -0.059 | 7070 |
| 990 | 655 | naringin | 7 µM | MCF7 | 0.419 | 0.038 | -0.076 | 3286 |
| 991 | 657 | hydrastine hydrochloride | 10 µM | MCF7 | 0.419 | 0.047 | -0.067 | 2889 |
| 992 | 1062 | AG-012559 | 10 µM | PC3 | 0.418 | 0.068 | -0.045 | 6920 |
| 993 | 513 | geldanamycin | 1 µM | MCF7 | 0.418 | 0.056 | -0.057 | 1066 |
| 994 | 699 | sulfamethoxazole | 16 µM | MCF7 | 0.418 | 0.053 | -0.061 | 4690 |
| 995 | 688 | pronetalol | 15 µM | PC3 | 0.418 | 0.055 | -0.059 | 3984 |
| 996 | 706 | betahistine | 17 µM | MCF7 | 0.416 | 0.053 | -0.06 | 4956 |
| 997 | 504 | 5211181 | 12 µM | MCF7 | 0.416 | 0.068 | -0.045 | 834 |
| 998 | 758 | equilin | 15 µM | MCF7 | 0.414 | 0.047 | -0.066 | 5620 |
| 999 | 728 | labetalol | 11 µM | PC3 | 0.414 | 0.053 | -0.06 | 4473 |
| 1000 | 1055 | 0297417-0002B | 10 µM | MCF7 | 0.414 | 0.05 | -0.062 | 6900 |
| 1001 | 513 | acetylsalicylic acid | 100 µM | MCF7 | 0.414 | 0.072 | -0.041 | 1042 |
| 1002 | 703 | orphenadrine | 13 µM | PC3 | 0.414 | 0.051 | -0.061 | 4537 |
| 1003 | 688 | chlorogenic acid | 11 µM | PC3 | 0.414 | 0.046 | -0.067 | 4024 |
| 1004 | 711 | PHA-00851261E | 1 µM | MCF7 | 0.413 | 0.054 | -0.059 | 3968 |
| 1005 | 745 | mefloquine | 10 µM | MCF7 | 0.412 | 0.058 | -0.054 | 6205 |
| 1006 | 647 | probucol | 8 µM | MCF7 | 0.412 | 0.056 | -0.056 | 3223 |
| 1007 | 692 | pepstatin | 6 µM | PC3 | 0.412 | 0.053 | -0.059 | 4206 |
| 1008 | 1081 | fulvestrant | 1 µM | PC3 | 0.412 | 0.053 | -0.059 | 7495 |
| 1009 | 728 | pipenzolate bromide | 9 µM | PC3 | 0.412 | 0.073 | -0.039 | 4484 |
| 1010 | 513 | 15-delta prostaglandin J2 | 10 µM | MCF7 | 0.41 | 0.069 | -0.043 | 1069 |
| 1011 | 743 | alprenolol | 14 µM | MCF7 | 0.41 | 0.047 | -0.064 | 6789 |
| 1012 | 682 | dantrolene | 12 µM | PC3 | 0.409 | 0.039 | -0.072 | 3786 |
| 1013 | 725 | valproic acid | 1 mM | MCF7 | 0.408 | 0.061 | -0.05 | 5206 |
| 1014 | 677 | Prestwick-1083 | 9 µM | MCF7 | 0.408 | 0.063 | -0.048 | 3538 |
| 1015 | 505 | rottlerin | 10 µM | MCF7 | 0.408 | 0.066 | -0.045 | 914 |
| 1016 | 647 | clorgiline | 13 µM | MCF7 | 0.407 | 0.055 | -0.056 | 3219 |
| 1017 | 683 | oxaprozin | 14 µM | PC3 | 0.405 | 0.041 | -0.069 | 3794 |
| 1018 | 725 | tanespimycin | 1 µM | MCF7 | 0.403 | 0.049 | -0.06 | 5222 |
| 1019 | 725 | chlorpromazine | 1 µM | MCF7 | 0.401 | 0.043 | -0.066 | 5214 |
| 1020 | 699 | cefazolin | 8 µM | MCF7 | 0.399 | 0.043 | -0.066 | 4708 |
| 1021 | 705 | bacampicillin | 8 µM | MCF7 | 0.399 | 0.044 | -0.065 | 4417 |
| 1022 | 1012 | fulvestrant | 1 µM | MCF7 | 0.398 | 0.054 | -0.054 | 5964 |
| 1023 | 735 | coralyne | 10 µM | MCF7 | 0.396 | 0.056 | -0.052 | 5418 |
| 1024 | 730 | caffeic acid | 22 µM | MCF7 | 0.395 | 0.05 | -0.057 | 5352 |
| 1025 | 762 | pivmecillinam | 8 µM | PC3 | 0.395 | 0.046 | -0.061 | 7312 |
| 1026 | 695 | salsolidin | 16 µM | MCF7 | 0.394 | 0.044 | -0.063 | 4810 |
| 1027 | 711 | citiolone | 25 µM | MCF7 | 0.394 | 0.042 | -0.065 | 3930 |
| 1028 | 745 | meprylcaine | 15 µM | MCF7 | 0.394 | 0.047 | -0.06 | 6204 |
| 1029 | 706 | hydrochlorothiazide | 13 µM | MCF7 | 0.393 | 0.064 | -0.043 | 4970 |
| 1030 | 755 | lorglumide | 8 µM | MCF7 | 0.393 | 0.052 | -0.054 | 6456 |
| 1031 | 761 | flunarizine | 8 µM | PC3 | 0.392 | 0.038 | -0.069 | 7252 |
| 1032 | 628 | sulindac | 11 µM | PC3 | 0.392 | 0.054 | -0.053 | 1815 |
| 1033 | 616 | isoconazole | 10 µM | PC3 | 0.391 | 0.051 | -0.055 | 2056 |
| 1034 | 1080 | semustine | 100 µM | MCF7 | 0.39 | 0.044 | -0.062 | 7487 |
| 1035 | 714 | debrisoquine | 9 µM | PC3 | 0.39 | 0.044 | -0.062 | 6688 |
| 1036 | 733 | streptomycin | 3 µM | PC3 | 0.389 | 0.048 | -0.057 | 5837 |
| 1037 | 714 | tacrine | 16 µM | PC3 | 0.389 | 0.045 | -0.061 | 6698 |
| 1038 | 1093 | 16-phenyltetranorprostaglandin E2 | 10 µM | PC3 | 0.389 | 0.055 | -0.051 | 7546 |
| 1039 | 1 | metformin | 1 mM | MCF7 | 0.389 | 0.051 | -0.054 | 4 |
| 1040 | 514 | demecolcine | 12 µM | MCF7 | 0.388 | 0.057 | -0.048 | 1103 |
| 1041 | 603 | troglitazone | 10 µM | PC3 | 0.388 | 0.051 | -0.054 | 1232 |
| 1042 | 656 | etifenin | 12 µM | MCF7 | 0.387 | 0.048 | -0.058 | 2838 |
| 1043 | 754 | tribenoside | 8 µM | PC3 | 0.385 | 0.045 | -0.06 | 6328 |
| 1044 | 637 | norfloxacin | 13 µM | MCF7 | 0.385 | 0.052 | -0.053 | 2253 |
| 1045 | 610 | lidocaine | 15 µM | PC3 | 0.385 | 0.052 | -0.052 | 1917 |
| 1046 | 704 | thiostrepton | 2 µM | PC3 | 0.384 | 0.042 | -0.063 | 4563 |
| 1047 | 691 | tiaprofenic acid | 15 µM | MCF7 | 0.384 | 0.051 | -0.053 | 4171 |
| 1048 | 703 | ciclacillin | 12 µM | PC3 | 0.383 | 0.067 | -0.037 | 4536 |
| 1049 | 698 | kawain | 17 µM | PC3 | 0.382 | 0.04 | -0.063 | 7369 |
| 1050 | 703 | homosalate | 15 µM | PC3 | 0.378 | 0.055 | -0.047 | 4533 |
| 1051 | 677 | risperidone | 10 µM | MCF7 | 0.377 | 0.047 | -0.056 | 3508 |
| 1052 | 67 | calmidazolium | 5 µM | MCF7 | 0.376 | 0.044 | -0.058 | 486 |
| 1053 | 1087 | IC-86621 | 1 µM | PC3 | 0.376 | 0.035 | -0.068 | 7518 |
| 1054 | 65 | rofecoxib | 10 µM | PC3 | 0.376 | 0.053 | -0.049 | 463 |
| 1055 | 690 | chlorogenic acid | 11 µM | MCF7 | 0.375 | 0.042 | -0.06 | 4142 |
| 1056 | 714 | amikacin | 7 µM | PC3 | 0.373 | 0.045 | -0.056 | 6715 |
| 1057 | 714 | felodipine | 10 µM | PC3 | 0.373 | 0.041 | -0.06 | 6695 |
| 1058 | 755 | florfenicol | 11 µM | MCF7 | 0.372 | 0.048 | -0.054 | 6460 |
| 1059 | 765 | alvespimycin | 100 nM | MCF7 | 0.372 | 0.047 | -0.054 | 6973 |
| 1060 | 746 | naltrexone | 10 µM | MCF7 | 0.369 | 0.057 | -0.043 | 6241 |
| 1061 | 757 | sirolimus | 100 nM | MCF7 | 0.367 | 0.047 | -0.053 | 5602 |
| 1062 | 94 | 3-aminobenzamide | 10 mM | MCF7 | 0.364 | 0.066 | -0.032 | 590 |
| 1063 | 718 | trimetazidine | 12 µM | PC3 | 0.363 | 0.05 | -0.049 | 5060 |
| 1064 | 772 | halofantrine | 7 µM | MCF7 | 0.361 | 0.039 | -0.059 | 7469 |
| 1065 | 1084 | CP-863187 | 10 µM | MCF7 | 0.357 | 0.041 | -0.056 | 7508 |
| 1066 | 703 | flutamide | 14 µM | PC3 | 0.345 | 0.038 | -0.056 | 4539 |
| 1067 | 743 | tamoxifen | 7 µM | MCF7 | 0.341 | 0.043 | -0.05 | 6768 |
| 1068 | 686 | dantrolene | 12 µM | MCF7 | 0.333 | 0.039 | -0.051 | 3867 |
| 1069 | 681 | betonicine | 25 µM | PC3 | 0.33 | 0.038 | -0.052 | 3745 |
| 1070 | 5 | estradiol | 10 nM | MCF7 | 0.33 | 0.04 | -0.05 | 121 |
| 1071 | 730 | chlortetracycline | 8 µM | MCF7 | 0.326 | 0.044 | -0.044 | 5360 |
| 1072 | 693 | salsolidin | 16 µM | PC3 | 0.316 | 0.034 | -0.052 | 4226 |
| 1073 | 754 | piribedil | 12 µM | PC3 | 0.304 | 0.036 | -0.047 | 6333 |
| 1074 | 712 | dioxybenzone | 16 µM | PC3 | 0.288 | 0.032 | -0.047 | 4638 |
| 1075 | 1071 | MS-275 | 10 µM | PC3 | 0 | 0.215 | 0.171 | 7074 |
| 1076 | 1075 | MS-275 | 10 µM | PC3 | 0 | 0.211 | 0.18 | 7084 |
| 1077 | 650 | valproic acid | 50 µM | HL60 | 0 | 0.169 | 0.116 | 2682 |
| 1078 | 634 | menadione | 23 µM | HL60 | 0 | 0.164 | 0.123 | 2439 |
| 1079 | 747 | trifluridine | 14 µM | MCF7 | 0 | 0.162 | 0.06 | 7176 |
| 1080 | 718 | trichostatin A | 100 nM | PC3 | 0 | 0.158 | 0.111 | 5065 |
| 1081 | 646 | mepyramine | 10 µM | MCF7 | 0 | 0.158 | 0.091 | 3184 |
| 1082 | 644 | galantamine | 11 µM | HL60 | 0 | 0.154 | 0.186 | 2131 |
| 1083 | 634 | felodipine | 10 µM | HL60 | 0 | 0.152 | 0.154 | 2433 |
| 1084 | 673 | rolitetracycline | 8 µM | MCF7 | 0 | 0.152 | 0.149 | 3369 |
| 1085 | 651 | iopamidol | 5 µM | HL60 | 0 | 0.151 | 0.107 | 2732 |
| 1086 | 610 | trichostatin A | 100 nM | PC3 | 0 | 0.15 | 0.103 | 1891 |
| 1087 | 646 | antazoline | 13 µM | MCF7 | 0 | 0.15 | 0.075 | 3173 |
| 1088 | 757 | thioridazine | 10 µM | MCF7 | 0 | 0.15 | 0.087 | 5590 |
| 1089 | 644 | succinylsulfathiazole | 11 µM | HL60 | 0 | 0.148 | 0.144 | 2166 |
| 1090 | 1049 | SC-560 | 10 µM | MCF7 | 0 | 0.148 | 0.09 | 6870 |
| 1091 | 505 | 5109870 | 25 µM | MCF7 | 0 | 0.147 | 0.079 | 904 |
| 1092 | 735 | methotrexate | 9 µM | MCF7 | 0 | 0.147 | 0.083 | 5419 |
| 1093 | 612 | ticlopidine | 13 µM | HL60 | 0 | 0.146 | 0.116 | 1975 |
| 1094 | 749 | trifluridine | 14 µM | HL60 | 0 | 0.146 | 0.172 | 6136 |
| 1095 | 656 | mycophenolic acid | 12 µM | MCF7 | 0 | 0.146 | 0.139 | 2857 |
| 1096 | 648 | altizide | 10 µM | HL60 | 0 | 0.144 | 0.105 | 2527 |
| 1097 | 647 | methotrexate | 9 µM | MCF7 | 0 | 0.144 | 0.106 | 3214 |
| 1098 | 626 | trichostatin A | 100 nM | MCF7 | 0 | 0.144 | 0.148 | 1637 |
| 1099 | 654 | lumicolchicine | 10 µM | MCF7 | 0 | 0.143 | 0.078 | 3254 |
| 1100 | 680 | trichostatin A | 100 nM | PC3 | 0 | 0.142 | 0.193 | 3688 |
| 1101 | 56 | sodium phenylbutyrate | 1 mM | PC3 | 0 | 0.142 | 0.041 | 434 |
| 1102 | 672 | suprofen | 15 µM | MCF7 | 0 | 0.141 | 0.086 | 3343 |
| 1103 | 712 | trichostatin A | 100 nM | PC3 | 0 | 0.14 | 0.116 | 4632 |
| 1104 | 650 | trichostatin A | 100 nM | HL60 | 0 | 0.137 | 0.069 | 2672 |
| 1105 | 635 | 6-azathymine | 31 µM | HL60 | 0 | 0.136 | 0.184 | 2466 |
| 1106 | 1007 | trichostatin A | 1 µM | PC3 | 0 | 0.136 | 0.176 | 5940 |
| 1107 | 672 | roxithromycin | 5 µM | MCF7 | 0 | 0.136 | 0.096 | 3331 |
| 1108 | 630 | N6-methyladenosine | 14 µM | HL60 | 0 | 0.136 | 0.168 | 1271 |
| 1109 | 762 | trichostatin A | 100 nM | PC3 | 0 | 0.136 | 0.109 | 7285 |
| 1110 | 711 | spiramycin | 5 µM | MCF7 | 0 | 0.135 | 0.088 | 3938 |
| 1111 | 691 | sanguinarine | 12 µM | MCF7 | 0 | 0.135 | 0.127 | 4168 |
| 1112 | 761 | clorsulon | 11 µM | PC3 | 0 | 0.135 | 0.08 | 7264 |
| 1113 | 650 | tretinoin | 1 µM | HL60 | 0 | 0.135 | 0.098 | 2671 |
| 1114 | 656 | trichostatin A | 100 nM | MCF7 | 0 | 0.134 | 0.107 | 2835 |
| 1115 | 1040 | PNU-0293363 | 10 µM | PC3 | 0 | 0.134 | 0.075 | 6568 |
| 1116 | 1055 | scriptaid | 10 µM | MCF7 | 0 | 0.134 | 0.079 | 6901 |
| 1117 | 1051 | trichostatin A | 1 µM | MCF7 | 0 | 0.134 | 0.143 | 6879 |
| 1118 | 628 | atracurium besilate | 3 µM | PC3 | 0 | 0.134 | 0.05 | 1824 |
| 1119 | 709 | trichostatin A | 100 nM | PC3 | 0 | 0.134 | 0.142 | 6609 |
| 1120 | 618 | trichostatin A | 100 nM | HL60 | 0 | 0.134 | 0.14 | 2370 |
| 1121 | 611 | trichostatin A | 100 nM | PC3 | 0 | 0.133 | 0.138 | 1951 |
| 1122 | 708 | hycanthone | 11 µM | MCF7 | 0 | 0.132 | 0.088 | 5691 |
| 1123 | 1012 | H-7 | 100 µM | MCF7 | 0 | 0.131 | 0.141 | 5963 |
| 1124 | 653 | naftidrofuryl | 8 µM | MCF7 | 0 | 0.131 | 0.097 | 2622 |
| 1125 | 649 | sulfadimidine | 13 µM | HL60 | 0 | 0.13 | 0.162 | 2560 |
| 1126 | 116 | estradiol | 10 nM | PC3 | 0 | 0.129 | 0.087 | 665 |
| 1127 | 653 | buspirone | 9 µM | MCF7 | 0 | 0.129 | 0.102 | 2637 |
| 1128 | 659 | rolitetracycline | 8 µM | HL60 | 0 | 0.129 | 0.155 | 3031 |
| 1129 | 715 | trichostatin A | 100 nM | PC3 | 0 | 0.129 | 0.136 | 6736 |
| 1130 | 687 | camptothecin | 11 µM | MCF7 | 0 | 0.129 | 0.176 | 3887 |
| 1131 | 772 | trichostatin A | 100 nM | MCF7 | 0 | 0.129 | 0.152 | 7453 |
| 1132 | 1009 | trichostatin A | 1 µM | PC3 | 0 | 0.129 | 0.126 | 5950 |
| 1133 | 727 | thioridazine | 10 µM | PC3 | 0 | 0.128 | 0.119 | 4454 |
| 1134 | 712 | dimethadione | 31 µM | PC3 | 0 | 0.128 | 0.062 | 4607 |
| 1135 | 715 | mitoxantrone | 8 µM | PC3 | 0 | 0.128 | 0.144 | 6755 |
| 1136 | 622 | trichostatin A | 100 nM | HL60 | 0 | 0.128 | 0.085 | 1561 |
| 1137 | 707 | trichostatin A | 100 nM | MCF7 | 0 | 0.127 | 0.093 | 5017 |
| 1138 | 82 | deferoxamine | 100 µM | MCF7 | 0 | 0.127 | 0.11 | 573 |
| 1139 | 708 | doxorubicin | 7 µM | MCF7 | 0 | 0.127 | 0.202 | 5671 |
| 1140 | 761 | levocabastine | 9 µM | PC3 | 0 | 0.127 | 0.079 | 7249 |
| 1141 | 729 | trichostatin A | 100 nM | MCF7 | 0 | 0.126 | 0.115 | 5308 |
| 1142 | 672 | azacitidine | 16 µM | MCF7 | 0 | 0.126 | 0.123 | 3348 |
| 1143 | 611 | hesperetin | 13 µM | PC3 | 0 | 0.126 | 0.128 | 1947 |
| 1144 | 67 | deferoxamine | 100 µM | MCF7 | 0 | 0.126 | 0.053 | 485 |
| 1145 | 728 | clidinium bromide | 9 µM | PC3 | 0 | 0.125 | 0.077 | 4499 |
| 1146 | 602 | trichostatin A | 100 nM | HL60 | 0 | 0.125 | 0.135 | 1153 |
| 1147 | 611 | verapamil | 8 µM | PC3 | 0 | 0.125 | 0.08 | 1927 |
| 1148 | 725 | trichostatin A | 100 nM | MCF7 | 0 | 0.124 | 0.115 | 5209 |
| 1149 | 618 | salbutamol | 17 µM | HL60 | 0 | 0.124 | 0.103 | 2344 |
| 1150 | 649 | flunixin | 8 µM | HL60 | 0 | 0.124 | 0.181 | 2552 |
| 1151 | 699 | 0175029-0000 | 10 µM | MCF7 | 0 | 0.124 | 0.165 | 4713 |
| 1152 | 657 | piracetam | 28 µM | MCF7 | 0 | 0.124 | 0.074 | 2861 |
| 1153 | 645 | Prestwick-675 | 10 µM | HL60 | 0 | 0.124 | 0.146 | 2187 |
| 1154 | 705 | tetramisole | 17 µM | MCF7 | 0 | 0.124 | 0.07 | 4412 |
| 1155 | 691 | tobramycin | 9 µM | MCF7 | 0 | 0.124 | 0.084 | 4162 |
| 1156 | 661 | Prestwick-981 | 11 µM | HL60 | 0 | 0.123 | 0.088 | 3125 |
| 1157 | 761 | Prestwick-1082 | 12 µM | PC3 | 0 | 0.123 | 0.141 | 7267 |
| 1158 | 726 | guanfacine | 14 µM | MCF7 | 0 | 0.123 | 0.08 | 5256 |
| 1159 | 603 | vorinostat | 10 µM | PC3 | 0 | 0.123 | 0.148 | 1220 |
| 1160 | 680 | 0173570-0000 | 10 µM | PC3 | 0 | 0.123 | 0.079 | 3690 |
| 1161 | 66 | imatinib | 10 µM | PC3 | 0 | 0.123 | 0.035 | 483 |
| 1162 | 651 | isopropamide iodide | 8 µM | HL60 | 0 | 0.122 | 0.117 | 2720 |
| 1163 | 703 | trichostatin A | 100 nM | PC3 | 0 | 0.122 | 0.116 | 4526 |
| 1164 | 719 | scoulerine | 12 µM | PC3 | 0 | 0.122 | 0.079 | 5111 |
| 1165 | 682 | trichostatin A | 100 nM | PC3 | 0 | 0.122 | 0.176 | 3787 |
| 1166 | 657 | chloroquine | 8 µM | MCF7 | 0 | 0.122 | 0.084 | 2869 |
| 1167 | 644 | picrotoxinin | 14 µM | HL60 | 0 | 0.122 | 0.16 | 2161 |
| 1168 | 623 | adenosine phosphate | 11 µM | HL60 | 0 | 0.122 | 0.142 | 1622 |
| 1169 | 651 | drofenine | 11 µM | HL60 | 0 | 0.121 | 0.096 | 2714 |
| 1170 | 665 | pivampicillin | 9 µM | HL60 | 0 | 0.121 | 0.242 | 2945 |
| 1171 | 649 | meclofenoxate | 14 µM | HL60 | 0 | 0.12 | 0.194 | 2546 |
| 1172 | 758 | trichostatin A | 100 nM | MCF7 | 0 | 0.12 | 0.145 | 5625 |
| 1173 | 712 | doxorubicin | 7 µM | PC3 | 0 | 0.12 | 0.129 | 4610 |
| 1174 | 649 | levonorgestrel | 13 µM | HL60 | 0 | 0.12 | 0.188 | 2547 |
| 1175 | 648 | napelline | 11 µM | HL60 | 0 | 0.12 | 0.155 | 2522 |
| 1176 | 706 | harmaline | 14 µM | MCF7 | 0 | 0.12 | 0.052 | 4968 |
| 1177 | 617 | etodolac | 14 µM | PC3 | 0 | 0.12 | 0.093 | 2091 |
| 1178 | 672 | propantheline bromide | 9 µM | MCF7 | 0 | 0.12 | 0.08 | 3352 |
| 1179 | 614 | nalbuphine | 10 µM | HL60 | 0 | 0.12 | 0.099 | 1379 |
| 1180 | 19 | rofecoxib | 10 µM | MCF7 | 0 | 0.119 | 0.087 | 256 |
| 1181 | 1073 | trichostatin A | 1 µM | PC3 | 0 | 0.119 | 0.125 | 7077 |
| 1182 | 743 | sulfapyridine | 16 µM | MCF7 | 0 | 0.119 | 0.075 | 6799 |
| 1183 | 649 | tinidazole | 16 µM | HL60 | 0 | 0.119 | 0.109 | 2568 |
| 1184 | 611 | loperamide | 8 µM | PC3 | 0 | 0.119 | 0.084 | 1949 |
| 1185 | 706 | carisoprodol | 15 µM | MCF7 | 0 | 0.118 | 0.077 | 4955 |
| 1186 | 612 | troleandomycin | 5 µM | HL60 | 0 | 0.118 | 0.103 | 1965 |
| 1187 | 662 | hydrocotarnine | 13 µM | MCF7 | 0 | 0.118 | 0.098 | 2765 |
| 1188 | 690 | mycophenolic acid | 12 µM | MCF7 | 0 | 0.118 | 0.154 | 4137 |
| 1189 | 611 | aciclovir | 18 µM | PC3 | 0 | 0.117 | 0.12 | 1960 |
| 1190 | 56 | valproic acid | 1 mM | PC3 | 0 | 0.117 | 0.091 | 433 |
| 1191 | 658 | reserpine | 7 µM | HL60 | 0 | 0.117 | 0.15 | 3003 |
| 1192 | 653 | clomifene | 7 µM | MCF7 | 0 | 0.117 | 0.074 | 2624 |
| 1193 | 612 | hydrochlorothiazide | 13 µM | HL60 | 0 | 0.117 | 0.099 | 1987 |
| 1194 | 616 | aminophenazone | 17 µM | PC3 | 0 | 0.117 | 0.066 | 2060 |
| 1195 | 618 | phentolamine | 13 µM | HL60 | 0 | 0.117 | 0.183 | 2362 |
| 1196 | 691 | trichostatin A | 100 nM | MCF7 | 0 | 0.117 | 0.114 | 4153 |
| 1197 | 761 | azapropazone | 13 µM | PC3 | 0 | 0.117 | 0.054 | 7277 |
| 1198 | 634 | epiandrosterone | 14 µM | HL60 | 0 | 0.116 | 0.116 | 2444 |
| 1199 | 749 | ticarcillin | 9 µM | HL60 | 0 | 0.116 | 0.217 | 6146 |
| 1200 | 651 | isoetarine | 12 µM | HL60 | 0 | 0.116 | 0.088 | 2711 |
| 1201 | 626 | sirolimus | 100 nM | MCF7 | 0 | 0.116 | 0.088 | 1632 |
| 1202 | 688 | etifenin | 12 µM | PC3 | 0 | 0.116 | 0.061 | 3998 |
| 1203 | 735 | trichostatin A | 100 nM | MCF7 | 0 | 0.116 | 0.098 | 5417 |
| 1204 | 673 | phthalylsulfathiazole | 10 µM | MCF7 | 0 | 0.116 | 0.073 | 3371 |
| 1205 | 1000 | trichostatin A | 1 µM | MCF7 | 0 | 0.116 | 0.085 | 5903 |
| 1206 | 502 | ikarugamycin | 2 µM | MCF7 | 0 | 0.116 | 0.08 | 974 |
| 1207 | 1033 | trichostatin A | 1 µM | MCF7 | 0 | 0.116 | 0.065 | 6551 |
| 1208 | 629 | dexpanthenol | 19 µM | HL60 | 0 | 0.115 | 0.133 | 1844 |
| 1209 | 634 | colecalciferol | 10 µM | HL60 | 0 | 0.115 | 0.191 | 2436 |
| 1210 | 744 | verteporfin | 3 µM | MCF7 | 0 | 0.115 | 0.079 | 6817 |
| 1211 | 673 | 3-acetylcoumarin | 21 µM | MCF7 | 0 | 0.115 | 0.086 | 3382 |
| 1212 | 642 | camptothecin | 11 µM | MCF7 | 0 | 0.115 | 0.17 | 2321 |
| 1213 | 708 | trichostatin A | 100 nM | MCF7 | 0 | 0.115 | 0.128 | 5693 |
| 1214 | 680 | levomepromazine | 9 µM | PC3 | 0 | 0.115 | 0.048 | 3701 |
| 1215 | 646 | quinidine | 11 µM | MCF7 | 0 | 0.115 | 0.109 | 3191 |
| 1216 | 761 | meclofenamic acid | 12 µM | PC3 | 0 | 0.114 | 0.063 | 7280 |
| 1217 | 736 | pyrvinium | 3 µM | MCF7 | 0 | 0.114 | 0.117 | 5439 |
| 1218 | 628 | amitriptyline | 13 µM | PC3 | 0 | 0.114 | 0.052 | 1823 |
| 1219 | 642 | kawain | 17 µM | MCF7 | 0 | 0.114 | 0.083 | 2299 |
| 1220 | 699 | 0175029-0000 | 1 µM | MCF7 | 0 | 0.114 | 0.111 | 4716 |
| 1221 | 704 | trichostatin A | 100 nM | PC3 | 0 | 0.114 | 0.144 | 4565 |
| 1222 | 705 | pentamidine | 7 µM | MCF7 | 0 | 0.114 | 0.088 | 4396 |
| 1223 | 612 | trichostatin A | 100 nM | HL60 | 0 | 0.114 | 0.134 | 1971 |
| 1224 | 708 | capsaicin | 13 µM | MCF7 | 0 | 0.113 | 0.103 | 5673 |
| 1225 | 719 | trichostatin A | 100 nM | PC3 | 0 | 0.113 | 0.175 | 5086 |
| 1226 | 637 | trichostatin A | 100 nM | MCF7 | 0 | 0.113 | 0.137 | 2268 |
| 1227 | 45 | nordihydroguaiaretic acid | 1 µM | ssMCF7 | 0 | 0.113 | 0.121 | 415 |
| 1228 | 655 | doxorubicin | 7 µM | MCF7 | 0 | 0.113 | 0.183 | 3291 |
| 1229 | 618 | tiapride | 11 µM | HL60 | 0 | 0.113 | 0.081 | 2331 |
| 1230 | 642 | hydralazine | 20 µM | MCF7 | 0 | 0.113 | 0.096 | 2311 |
| 1231 | 727 | trichostatin A | 1 µM | PC3 | 0 | 0.112 | 0.183 | 4458 |
| 1232 | 698 | trichostatin A | 100 nM | PC3 | 0 | 0.112 | 0.169 | 7387 |
| 1233 | 1059 | SB-202190 | 1 µM | MCF7 | 0 | 0.112 | 0.055 | 6909 |
| 1234 | 1059 | trichostatin A | 1 µM | MCF7 | 0 | 0.112 | 0.074 | 6910 |
| 1235 | 654 | buflomedil | 12 µM | MCF7 | 0 | 0.112 | 0.114 | 3274 |
| 1236 | 678 | sulfadoxine | 13 µM | MCF7 | 0 | 0.112 | 0.093 | 3547 |
| 1237 | 685 | levonorgestrel | 13 µM | MCF7 | 0 | 0.112 | 0.124 | 3606 |
| 1238 | 757 | trichostatin A | 100 nM | MCF7 | 0 | 0.112 | 0.145 | 5572 |
| 1239 | 695 | primaquine | 9 µM | MCF7 | 0 | 0.111 | 0.1 | 4845 |
| 1240 | 688 | sisomicin | 3 µM | PC3 | 0 | 0.111 | 0.092 | 4014 |
| 1241 | 685 | tropicamide | 14 µM | MCF7 | 0 | 0.111 | 0.126 | 3619 |
| 1242 | 687 | fenbendazole | 13 µM | MCF7 | 0 | 0.111 | 0.071 | 3888 |
| 1243 | 647 | mitoxantrone | 8 µM | MCF7 | 0 | 0.111 | 0.197 | 3232 |
| 1244 | 689 | trichostatin A | 100 nM | PC3 | 0 | 0.111 | 0.189 | 4072 |
| 1245 | 1074 | GW-8510 | 10 µM | MCF7 | 0 | 0.111 | 0.177 | 7080 |
| 1246 | 701 | trichostatin A | 100 nM | PC3 | 0 | 0.111 | 0.184 | 4302 |
| 1247 | 661 | proxymetacaine | 12 µM | HL60 | 0 | 0.11 | 0.147 | 3113 |
| 1248 | 1049 | STOCK1N-28457 | 20 µM | MCF7 | 0 | 0.11 | 0.091 | 6869 |
| 1249 | 672 | trichostatin A | 100 nM | MCF7 | 0 | 0.11 | 0.1 | 3332 |
| 1250 | 660 | pipemidic acid | 13 µM | HL60 | 0 | 0.11 | 0.157 | 3093 |
| 1251 | 629 | meticrane | 15 µM | HL60 | 0 | 0.11 | 0.186 | 1834 |
| 1252 | 642 | sulfamethoxazole | 16 µM | MCF7 | 0 | 0.11 | 0.066 | 2296 |
| 1253 | 73 | staurosporine | 10 nM | SKMEL5 | 0 | 0.11 | 0.081 | 508 |
| 1254 | 611 | guanabenz | 14 µM | PC3 | 0 | 0.11 | 0.109 | 1961 |
| 1255 | 629 | epirizole | 17 µM | HL60 | 0 | 0.11 | 0.123 | 1845 |
| 1256 | 730 | trichostatin A | 100 nM | MCF7 | 0 | 0.109 | 0.133 | 5336 |
| 1257 | 720 | rifabutin | 5 µM | MCF7 | 0 | 0.109 | 0.099 | 4349 |
| 1258 | 619 | enalapril | 8 µM | HL60 | 0 | 0.109 | 0.122 | 2397 |
| 1259 | 687 | 2,6-dimethylpiperidine | 27 µM | MCF7 | 0 | 0.109 | 0.107 | 3889 |
| 1260 | 670 | guaifenesin | 20 µM | MCF7 | 0 | 0.109 | 0.084 | 3431 |
| 1261 | 618 | ketoprofen | 16 µM | HL60 | 0 | 0.109 | 0.142 | 2354 |
| 1262 | 690 | N-acetyl-L-aspartic acid | 23 µM | MCF7 | 0 | 0.109 | 0.1 | 4125 |
| 1263 | 648 | metoprolol | 6 µM | HL60 | 0 | 0.109 | 0.144 | 2543 |
| 1264 | 616 | ampyrone | 20 µM | PC3 | 0 | 0.109 | 0.075 | 2086 |
| 1265 | 635 | trichostatin A | 100 nM | HL60 | 0 | 0.109 | 0.172 | 2474 |
| 1266 | 712 | metyrapone | 18 µM | PC3 | 0 | 0.108 | 0.077 | 4606 |
| 1267 | 748 | isoconazole | 10 µM | MCF7 | 0 | 0.108 | 0.121 | 7211 |
| 1268 | 749 | oxyphenbutazone | 12 µM | HL60 | 0 | 0.108 | 0.129 | 6160 |
| 1269 | 619 | fenoterol | 10 µM | HL60 | 0 | 0.108 | 0.144 | 2378 |
| 1270 | 729 | primidone | 18 µM | MCF7 | 0 | 0.108 | 0.084 | 5323 |
| 1271 | 743 | trichostatin A | 100 nM | MCF7 | 0 | 0.108 | 0.122 | 6784 |
| 1272 | 678 | ronidazole | 20 µM | MCF7 | 0 | 0.108 | 0.084 | 3557 |
| 1273 | 673 | crotamiton | 20 µM | MCF7 | 0 | 0.108 | 0.109 | 3388 |
| 1274 | 757 | sirolimus | 100 nM | MCF7 | 0 | 0.108 | 0.074 | 5581 |
| 1275 | 755 | trichostatin A | 100 nM | MCF7 | 0 | 0.108 | 0.101 | 6454 |
| 1276 | 514 | tyrphostin AG-825 | 25 µM | MCF7 | 0 | 0.108 | 0.12 | 1114 |
| 1277 | 695 | oxetacaine | 9 µM | MCF7 | 0 | 0.108 | 0.115 | 4829 |
| 1278 | 754 | trichostatin A | 100 nM | PC3 | 0 | 0.108 | 0.171 | 6340 |
| 1279 | 677 | trichostatin A | 100 nM | MCF7 | 0 | 0.108 | 0.137 | 3510 |
| 1280 | 754 | raubasine | 10 µM | PC3 | 0 | 0.108 | 0.064 | 6360 |
| 1281 | 623 | trichostatin A | 100 nM | HL60 | 0 | 0.108 | 0.165 | 1612 |
| 1282 | 692 | gabexate | 10 µM | PC3 | 0 | 0.108 | 0.095 | 4220 |
| 1283 | 24 | staurosporine | 1 µM | MCF7 | 0 | 0.107 | 0.11 | 312 |
| 1284 | 651 | trichostatin A | 100 nM | HL60 | 0 | 0.107 | 0.167 | 2721 |
| 1285 | 713 | menadione | 23 µM | PC3 | 0 | 0.107 | 0.121 | 4662 |
| 1286 | 654 | trichostatin A | 100 nM | MCF7 | 0 | 0.107 | 0.093 | 3243 |
| 1287 | 658 | trichlormethiazide | 11 µM | HL60 | 0 | 0.107 | 0.134 | 2998 |
| 1288 | 1022 | F0447-0125 | 10 µM | MCF7 | 0 | 0.107 | 0.075 | 6396 |
| 1289 | 657 | resveratrol | 18 µM | MCF7 | 0 | 0.107 | 0.134 | 2865 |
| 1290 | 1019 | PF-00539758-00 | 10 µM | PC3 | 0 | 0.106 | 0.086 | 6379 |
| 1291 | 630 | flupentixol | 8 µM | HL60 | 0 | 0.106 | 0.189 | 1288 |
| 1292 | 626 | tretinoin | 1 µM | MCF7 | 0 | 0.106 | 0.074 | 1636 |
| 1293 | 772 | trazodone | 10 µM | MCF7 | 0 | 0.106 | 0.099 | 7452 |
| 1294 | 657 | imipenem | 13 µM | MCF7 | 0 | 0.106 | 0.1 | 2873 |
| 1295 | 603 | valproic acid | 1 mM | PC3 | 0 | 0.105 | 0.118 | 1209 |
| 1296 | 711 | trichostatin A | 100 nM | MCF7 | 0 | 0.105 | 0.098 | 3979 |
| 1297 | 726 | bephenium hydroxynaphthoate | 9 µM | MCF7 | 0 | 0.105 | 0.081 | 5263 |
| 1298 | 728 | ampyrone | 20 µM | PC3 | 0 | 0.105 | 0.105 | 4507 |
| 1299 | 732 | ellipticine | 16 µM | PC3 | 0 | 0.105 | 0.184 | 5779 |
| 1300 | 673 | monobenzone | 20 µM | MCF7 | 0 | 0.105 | 0.062 | 3391 |
| 1301 | 514 | DL-PPMP | 2 µM | MCF7 | 0 | 0.105 | 0.062 | 1121 |
| 1302 | 611 | lisuride | 12 µM | PC3 | 0 | 0.105 | 0.074 | 1962 |
| 1303 | 734 | trichostatin A | 100 nM | PC3 | 0 | 0.105 | 0.128 | 5882 |
| 1304 | 659 | esculin | 12 µM | HL60 | 0 | 0.105 | 0.145 | 3052 |
| 1305 | 603 | trichostatin A | 1 µM | PC3 | 0 | 0.105 | 0.187 | 1234 |
| 1306 | 1068 | GW-8510 | 10 µM | MCF7 | 0 | 0.105 | 0.148 | 7062 |
| 1307 | 735 | terguride | 12 µM | MCF7 | 0 | 0.105 | 0.054 | 5400 |
| 1308 | 767 | troglitazone | 10 µM | MCF7 | 0 | 0.105 | 0.054 | 6949 |
| 1309 | 683 | trichostatin A | 100 nM | PC3 | 0 | 0.105 | 0.173 | 3791 |
| 1310 | 632 | trichostatin A | 100 nM | MCF7 | 0 | 0.104 | 0.105 | 1471 |
| 1311 | 700 | hydralazine | 20 µM | MCF7 | 0 | 0.104 | 0.074 | 4746 |
| 1312 | 711 | methylbenzethonium chloride | 9 µM | MCF7 | 0 | 0.104 | 0.09 | 3943 |
| 1313 | 678 | ciprofibrate | 14 µM | MCF7 | 0 | 0.104 | 0.068 | 3561 |
| 1314 | 659 | doxazosin | 7 µM | HL60 | 0 | 0.104 | 0.156 | 3024 |
| 1315 | 764 | ramipril | 10 µM | PC3 | 0 | 0.104 | 0.082 | 7144 |
| 1316 | 754 | fenoterol | 10 µM | PC3 | 0 | 0.104 | 0.063 | 6331 |
| 1317 | 757 | 15-delta prostaglandin J2 | 10 µM | MCF7 | 0 | 0.104 | 0.054 | 5591 |
| 1318 | 703 | rifabutin | 5 µM | PC3 | 0 | 0.104 | 0.153 | 4527 |
| 1319 | 626 | trichostatin A | 1 µM | MCF7 | 0 | 0.104 | 0.073 | 1659 |
| 1320 | 635 | betahistine | 17 µM | HL60 | 0 | 0.104 | 0.091 | 2472 |
| 1321 | 1013 | H-7 | 100 µM | PC3 | 0 | 0.104 | 0.148 | 5968 |
| 1322 | 28 | mercaptopurine | 100 µM | MCF7 | 0 | 0.104 | 0.075 | 334 |
| 1323 | 618 | kawain | 17 µM | HL60 | 0 | 0.104 | 0.106 | 2337 |
| 1324 | 651 | ebselen | 15 µM | HL60 | 0 | 0.104 | 0.187 | 2717 |
| 1325 | 718 | antimycin A | 7 µM | PC3 | 0 | 0.104 | 0.063 | 5053 |
| 1326 | 1066 | alsterpaullone | 10 µM | MCF7 | 0 | 0.104 | 0.185 | 7051 |
| 1327 | 662 | ellipticine | 16 µM | MCF7 | 0 | 0.104 | 0.142 | 2758 |
| 1328 | 771 | difenidol | 12 µM | MCF7 | 0 | 0.103 | 0.069 | 7406 |
| 1329 | 743 | oxedrine | 24 µM | MCF7 | 0 | 0.103 | 0.055 | 6798 |
| 1330 | 623 | cefoperazone | 6 µM | HL60 | 0 | 0.103 | 0.124 | 1627 |
| 1331 | 746 | quinidine | 11 µM | MCF7 | 0 | 0.103 | 0.099 | 6267 |
| 1332 | 611 | midecamycin | 5 µM | PC3 | 0 | 0.103 | 0.082 | 1943 |
| 1333 | 738 | resveratrol | 18 µM | MCF7 | 0 | 0.103 | 0.067 | 5509 |
| 1334 | 710 | ketanserin | 7 µM | PC3 | 0 | 0.103 | 0.069 | 6649 |
| 1335 | 646 | amodiaquine | 9 µM | MCF7 | 0 | 0.103 | 0.062 | 3186 |
| 1336 | 707 | methotrexate | 9 µM | MCF7 | 0 | 0.103 | 0.102 | 5000 |
| 1337 | 631 | bacampicillin | 8 µM | HL60 | 0 | 0.103 | 0.117 | 1337 |
| 1338 | 651 | cinoxacin | 15 µM | HL60 | 0 | 0.103 | 0.159 | 2722 |
| 1339 | 616 | trichostatin A | 100 nM | PC3 | 0 | 0.103 | 0.112 | 2084 |
| 1340 | 681 | trichostatin A | 100 nM | PC3 | 0 | 0.103 | 0.111 | 3746 |
| 1341 | 659 | nicotinic acid | 32 µM | HL60 | 0 | 0.103 | 0.125 | 3043 |
| 1342 | 645 | myosmine | 27 µM | HL60 | 0 | 0.102 | 0.118 | 2199 |
| 1343 | 631 | ginkgolide A | 10 µM | HL60 | 0 | 0.102 | 0.19 | 1324 |
| 1344 | 731 | trichostatin A | 100 nM | PC3 | 0 | 0.102 | 0.171 | 5745 |
| 1345 | 692 | mestranol | 13 µM | PC3 | 0 | 0.102 | 0.064 | 4208 |
| 1346 | 623 | etoposide | 7 µM | HL60 | 0 | 0.102 | 0.16 | 1626 |
| 1347 | 1076 | 0316684-0000 | 10 µM | MCF7 | 0 | 0.102 | 0.132 | 7093 |
| 1348 | 746 | ellipticine | 16 µM | MCF7 | 0 | 0.102 | 0.13 | 6253 |
| 1349 | 712 | hycanthone | 11 µM | PC3 | 0 | 0.102 | 0.069 | 4630 |
| 1350 | 730 | mitoxantrone | 8 µM | MCF7 | 0 | 0.102 | 0.115 | 5354 |
| 1351 | 631 | fusaric acid | 22 µM | HL60 | 0 | 0.102 | 0.154 | 1308 |
| 1352 | 646 | trichostatin A | 100 nM | MCF7 | 0 | 0.102 | 0.089 | 3177 |
| 1353 | 117 | monastrol | 100 µM | MCF7 | 0 | 0.102 | 0.085 | 681 |
| 1354 | 630 | tacrine | 16 µM | HL60 | 0 | 0.102 | 0.13 | 1278 |
| 1355 | 726 | 3-acetylcoumarin | 21 µM | MCF7 | 0 | 0.102 | 0.108 | 5259 |
| 1356 | 704 | tetramisole | 17 µM | PC3 | 0 | 0.102 | 0.061 | 4587 |
| 1357 | 689 | tolmetin | 13 µM | PC3 | 0 | 0.101 | 0.075 | 4088 |
| 1358 | 657 | laudanosine | 11 µM | MCF7 | 0 | 0.101 | 0.098 | 2890 |
| 1359 | 658 | beclometasone | 8 µM | HL60 | 0 | 0.101 | 0.141 | 3001 |
| 1360 | 653 | dextromethorphan | 11 µM | MCF7 | 0 | 0.101 | 0.075 | 2636 |
| 1361 | 693 | phenazone | 21 µM | PC3 | 0 | 0.101 | 0.071 | 4251 |
| 1362 | 712 | zimeldine | 10 µM | PC3 | 0 | 0.101 | 0.062 | 4609 |
| 1363 | 617 | trichostatin A | 100 nM | PC3 | 0 | 0.101 | 0.142 | 2105 |
| 1364 | 613 | verapamil | 8 µM | HL60 | 0 | 0.101 | 0.1 | 2009 |
| 1365 | 648 | cefalotin | 10 µM | HL60 | 0 | 0.101 | 0.203 | 2517 |
| 1366 | 644 | retrorsine | 11 µM | HL60 | 0 | 0.1 | 0.146 | 2129 |
| 1367 | 694 | idoxuridine | 11 µM | MCF7 | 0 | 0.1 | 0.128 | 4785 |
| 1368 | 646 | fipexide | 9 µM | MCF7 | 0 | 0.1 | 0.062 | 3176 |
| 1369 | 619 | doxepin | 13 µM | HL60 | 0 | 0.1 | 0.15 | 2384 |
| 1370 | 755 | clioquinol | 13 µM | MCF7 | 0 | 0.1 | 0.075 | 6461 |
| 1371 | 644 | conessine | 11 µM | HL60 | 0 | 0.1 | 0.122 | 2135 |
| 1372 | 745 | butacaine | 13 µM | MCF7 | 0 | 0.1 | 0.09 | 6225 |
| 1373 | 671 | ebselen | 15 µM | MCF7 | 0 | 0.1 | 0.067 | 3458 |
| 1374 | 692 | epitiostanol | 13 µM | PC3 | 0 | 0.1 | 0.068 | 4204 |
| 1375 | 688 | trichostatin A | 100 nM | PC3 | 0 | 0.1 | 0.136 | 3993 |
| 1376 | 654 | N-acetyl-L-aspartic acid | 23 µM | MCF7 | 0 | 0.1 | 0.062 | 3265 |
| 1377 | 744 | clomipramine | 11 µM | MCF7 | 0 | 0.1 | 0.059 | 6825 |
| 1378 | 658 | spectinomycin | 10 µM | HL60 | 0 | 0.1 | 0.114 | 2987 |
| 1379 | 690 | phenacetin | 22 µM | MCF7 | 0 | 0.1 | 0.08 | 4111 |
| 1380 | 741 | trichostatin A | 100 nM | MCF7 | 0 | 0.1 | 0.106 | 5987 |
| 1381 | 634 | naringin | 7 µM | HL60 | 0 | 0.1 | 0.169 | 2425 |
| 1382 | 611 | lynestrenol | 14 µM | PC3 | 0 | 0.1 | 0.132 | 1953 |
| 1383 | 1031 | trichostatin A | 1 µM | PC3 | 0 | 0.1 | 0.103 | 6439 |
| 1384 | 715 | neostigmine bromide | 13 µM | PC3 | 0 | 0.099 | 0.106 | 6735 |
| 1385 | 762 | netilmicin | 3 µM | PC3 | 0 | 0.099 | 0.068 | 7302 |
| 1386 | 761 | trichostatin A | 100 nM | PC3 | 0 | 0.099 | 0.209 | 7245 |
| 1387 | 654 | brompheniramine | 9 µM | MCF7 | 0 | 0.099 | 0.087 | 3271 |
| 1388 | 1065 | trichostatin A | 1 µM | PC3 | 0 | 0.099 | 0.135 | 7047 |
| 1389 | 692 | benzocaine | 24 µM | PC3 | 0 | 0.099 | 0.056 | 4224 |
| 1390 | 734 | vidarabine | 15 µM | PC3 | 0 | 0.099 | 0.063 | 5850 |
| 1391 | 648 | trichostatin A | 100 nM | HL60 | 0 | 0.099 | 0.144 | 2523 |
| 1392 | 767 | vorinostat | 10 µM | MCF7 | 0 | 0.099 | 0.133 | 6939 |
| 1393 | 730 | cyproheptadine | 12 µM | MCF7 | 0 | 0.099 | 0.08 | 5340 |
| 1394 | 613 | methotrexate | 9 µM | HL60 | 0 | 0.099 | 0.103 | 2041 |
| 1395 | 727 | trichostatin A | 100 nM | PC3 | 0 | 0.099 | 0.142 | 4436 |
| 1396 | 649 | demeclocycline | 8 µM | HL60 | 0 | 0.099 | 0.109 | 2545 |
| 1397 | 687 | diethylstilbestrol | 15 µM | MCF7 | 0 | 0.099 | 0.078 | 3895 |
| 1398 | 658 | arcaine | 15 µM | HL60 | 0 | 0.099 | 0.153 | 3010 |
| 1399 | 735 | chlorpropamide | 14 µM | MCF7 | 0 | 0.099 | 0.064 | 5391 |
| 1400 | 761 | thiamphenicol | 11 µM | PC3 | 0 | 0.099 | 0.065 | 7274 |
| 1401 | 737 | octopamine | 21 µM | MCF7 | 0 | 0.099 | 0.079 | 5469 |
| 1402 | 711 | phentolamine | 13 µM | MCF7 | 0 | 0.099 | 0.118 | 3971 |
| 1403 | 614 | isoniazid | 29 µM | HL60 | 0 | 0.099 | 0.111 | 1399 |
| 1404 | 1004 | sirolimus | 100 nM | MCF7 | 0 | 0.099 | 0.082 | 5927 |
| 1405 | 651 | cefapirin | 9 µM | HL60 | 0 | 0.099 | 0.116 | 2730 |
| 1406 | 708 | ketoconazole | 8 µM | MCF7 | 0 | 0.098 | 0.078 | 5685 |
| 1407 | 744 | trichostatin A | 100 nM | MCF7 | 0 | 0.098 | 0.119 | 6820 |
| 1408 | 653 | bezafibrate | 11 µM | MCF7 | 0 | 0.098 | 0.111 | 2630 |
| 1409 | 706 | daunorubicin | 7 µM | MCF7 | 0 | 0.098 | 0.155 | 4983 |
| 1410 | 657 | bromperidol | 10 µM | MCF7 | 0 | 0.098 | 0.079 | 2872 |
| 1411 | 635 | norcyclobenzaprine | 15 µM | HL60 | 0 | 0.098 | 0.143 | 2469 |
| 1412 | 705 | trichostatin A | 100 nM | MCF7 | 0 | 0.098 | 0.101 | 4388 |
| 1413 | 686 | ethisterone | 13 µM | MCF7 | 0 | 0.098 | 0.108 | 3864 |
| 1414 | 732 | trichostatin A | 100 nM | PC3 | 0 | 0.098 | 0.154 | 5802 |
| 1415 | 504 | oxaprozin | 300 µM | MCF7 | 0 | 0.098 | 0.114 | 863 |
| 1416 | 670 | furaltadone | 11 µM | MCF7 | 0 | 0.098 | 0.106 | 3413 |
| 1417 | 670 | piperacillin | 7 µM | MCF7 | 0 | 0.098 | 0.08 | 3420 |
| 1418 | 657 | propafenone | 11 µM | MCF7 | 0 | 0.098 | 0.091 | 2871 |
| 1419 | 602 | valproic acid | 500 µM | HL60 | 0 | 0.098 | 0.116 | 1181 |
| 1420 | 641 | epivincamine | 11 µM | HL60 | 0 | 0.098 | 0.139 | 1783 |
| 1421 | 682 | spiramycin | 5 µM | PC3 | 0 | 0.098 | 0.094 | 3762 |
| 1422 | 55 | tanespimycin | 1 µM | PC3 | 0 | 0.098 | 0.055 | 432 |
| 1423 | 619 | aminoglutethimide | 17 µM | HL60 | 0 | 0.098 | 0.109 | 2390 |
| 1424 | 1013 | fulvestrant | 1 µM | PC3 | 0 | 0.097 | 0.061 | 5969 |
| 1425 | 626 | acetylsalicylic acid | 100 µM | MCF7 | 0 | 0.097 | 0.084 | 1629 |
| 1426 | 657 | phenindione | 18 µM | MCF7 | 0 | 0.097 | 0.095 | 2868 |
| 1427 | 703 | camptothecin | 11 µM | PC3 | 0 | 0.097 | 0.194 | 4541 |
| 1428 | 731 | meprylcaine | 15 µM | PC3 | 0 | 0.097 | 0.089 | 5723 |
| 1429 | 647 | trichostatin A | 100 nM | MCF7 | 0 | 0.097 | 0.132 | 3227 |
| 1430 | 664 | sanguinarine | 12 µM | HL60 | 0 | 0.097 | 0.229 | 2927 |
| 1431 | 766 | triprolidine | 13 µM | MCF7 | 0 | 0.097 | 0.051 | 7008 |
| 1432 | 626 | LY-294002 | 100 nM | MCF7 | 0 | 0.097 | 0.095 | 1641 |
| 1433 | 658 | piromidic acid | 14 µM | HL60 | 0 | 0.097 | 0.117 | 2996 |
| 1434 | 631 | cefadroxil | 11 µM | HL60 | 0 | 0.097 | 0.109 | 1323 |
| 1435 | 689 | L-methionine sulfoximine | 22 µM | PC3 | 0 | 0.097 | 0.055 | 4070 |
| 1436 | 655 | amphotericin B | 4 µM | MCF7 | 0 | 0.097 | 0.083 | 3303 |
| 1437 | 644 | trichostatin A | 100 nM | HL60 | 0 | 0.097 | 0.141 | 2137 |
| 1438 | 681 | hydralazine | 20 µM | PC3 | 0 | 0.097 | 0.109 | 3724 |
| 1439 | 698 | 0173570-0000 | 10 µM | PC3 | 0 | 0.097 | 0.076 | 7389 |
| 1440 | 642 | phenformin | 17 µM | MCF7 | 0 | 0.097 | 0.091 | 2312 |
| 1441 | 1071 | trichostatin A | 1 µM | PC3 | 0 | 0.097 | 0.124 | 7073 |
| 1442 | 705 | terconazole | 8 µM | MCF7 | 0 | 0.097 | 0.078 | 4407 |
| 1443 | 653 | zoxazolamine | 24 µM | MCF7 | 0 | 0.097 | 0.065 | 2625 |
| 1444 | 694 | sulfathiazole | 16 µM | MCF7 | 0 | 0.097 | 0.071 | 4769 |
| 1445 | 728 | (-)-isoprenaline | 16 µM | PC3 | 0 | 0.097 | 0.056 | 4495 |
| 1446 | 618 | prilocaine | 16 µM | HL60 | 0 | 0.097 | 0.106 | 2352 |
| 1447 | 757 | acetylsalicylic acid | 100 µM | MCF7 | 0 | 0.097 | 0.077 | 5564 |
| 1448 | 756 | protriptyline | 13 µM | MCF7 | 0 | 0.097 | 0.05 | 6498 |
| 1449 | 688 | rifampicin | 5 µM | PC3 | 0 | 0.097 | 0.045 | 4008 |
| 1450 | 657 | triflusal | 16 µM | MCF7 | 0 | 0.097 | 0.077 | 2867 |
| 1451 | 642 | clemizole | 11 µM | MCF7 | 0 | 0.097 | 0.102 | 2301 |
| 1452 | 737 | minocycline | 8 µM | MCF7 | 0 | 0.097 | 0.113 | 5496 |
| 1453 | 513 | sirolimus | 100 nM | MCF7 | 0 | 0.096 | 0.082 | 1045 |
| 1454 | 1064 | trichostatin A | 1 µM | MCF7 | 0 | 0.096 | 0.11 | 7043 |
| 1455 | 727 | estradiol | 100 nM | PC3 | 0 | 0.096 | 0.099 | 4432 |
| 1456 | 626 | fulvestrant | 1 µM | MCF7 | 0 | 0.096 | 0.071 | 1630 |
| 1457 | 672 | oxolamine | 9 µM | MCF7 | 0 | 0.096 | 0.066 | 3344 |
| 1458 | 755 | desoxycortone | 12 µM | MCF7 | 0 | 0.096 | 0.064 | 6476 |
| 1459 | 737 | imidurea | 10 µM | MCF7 | 0 | 0.096 | 0.068 | 5481 |
| 1460 | 631 | cyanocobalamin | 3 µM | HL60 | 0 | 0.096 | 0.14 | 1315 |
| 1461 | 612 | captopril | 17 µM | HL60 | 0 | 0.096 | 0.097 | 1988 |
| 1462 | 630 | androsterone | 14 µM | HL60 | 0 | 0.096 | 0.179 | 1296 |
| 1463 | 646 | cinnarizine | 11 µM | MCF7 | 0 | 0.096 | 0.095 | 3175 |
| 1464 | 653 | androsterone | 14 µM | MCF7 | 0 | 0.096 | 0.076 | 2650 |
| 1465 | 663 | trichostatin A | 100 nM | MCF7 | 0 | 0.096 | 0.101 | 2794 |
| 1466 | 504 | depudecin | 1 µM | MCF7 | 0 | 0.096 | 0.118 | 874 |
| 1467 | 752 | napelline | 11 µM | MCF7 | 0 | 0.096 | 0.077 | 6084 |
| 1468 | 685 | prilocaine | 16 µM | MCF7 | 0 | 0.096 | 0.064 | 3624 |
| 1469 | 699 | alexidine | 7 µM | MCF7 | 0 | 0.096 | 0.072 | 4721 |
| 1470 | 658 | indoprofen | 14 µM | HL60 | 0 | 0.096 | 0.089 | 3007 |
| 1471 | 637 | thalidomide | 15 µM | MCF7 | 0 | 0.095 | 0.058 | 2258 |
| 1472 | 603 | trichostatin A | 100 nM | PC3 | 0 | 0.095 | 0.13 | 1212 |
| 1473 | 732 | sulfamethizole | 15 µM | PC3 | 0 | 0.095 | 0.059 | 5798 |
| 1474 | 649 | mephentermine | 9 µM | HL60 | 0 | 0.095 | 0.092 | 2563 |
| 1475 | 657 | Trolox C | 16 µM | MCF7 | 0 | 0.095 | 0.117 | 2883 |
| 1476 | 727 | alpha-estradiol | 10 nM | PC3 | 0 | 0.095 | 0.126 | 4434 |
| 1477 | 654 | primaquine | 9 µM | MCF7 | 0 | 0.095 | 0.077 | 3279 |
| 1478 | 636 | thioproperazine | 6 µM | MCF7 | 0 | 0.095 | 0.094 | 2236 |
| 1479 | 631 | metrizamide | 5 µM | HL60 | 0 | 0.095 | 0.129 | 1318 |
| 1480 | 642 | salbutamol | 17 µM | MCF7 | 0 | 0.095 | 0.08 | 2306 |
| 1481 | 631 | 7-aminocephalosporanic acid | 15 µM | HL60 | 0 | 0.095 | 0.144 | 1322 |
| 1482 | 660 | tyloxapol | 4 µM | HL60 | 0 | 0.095 | 0.148 | 3074 |
| 1483 | 741 | dosulepin | 12 µM | MCF7 | 0 | 0.095 | 0.138 | 5986 |
| 1484 | 695 | trichostatin A | 100 nM | MCF7 | 0 | 0.095 | 0.07 | 4821 |
| 1485 | 710 | ciclopirox | 15 µM | PC3 | 0 | 0.095 | 0.076 | 6677 |
| 1486 | 654 | fusaric acid | 22 µM | MCF7 | 0 | 0.095 | 0.043 | 3245 |
| 1487 | 729 | debrisoquine | 9 µM | MCF7 | 0 | 0.095 | 0.05 | 5288 |
| 1488 | 619 | glafenine | 10 µM | HL60 | 0 | 0.095 | 0.135 | 2387 |
| 1489 | 614 | acenocoumarol | 11 µM | HL60 | 0 | 0.095 | 0.13 | 1394 |
| 1490 | 672 | trichlormethiazide | 11 µM | MCF7 | 0 | 0.095 | 0.104 | 3337 |
| 1491 | 644 | dydrogesterone | 13 µM | HL60 | 0 | 0.095 | 0.13 | 2156 |
| 1492 | 672 | cefsulodin | 7 µM | MCF7 | 0 | 0.095 | 0.044 | 3328 |
| 1493 | 752 | benzethonium chloride | 9 µM | MCF7 | 0 | 0.095 | 0.109 | 6070 |
| 1494 | 662 | trichostatin A | 100 nM | MCF7 | 0 | 0.094 | 0.091 | 2777 |
| 1495 | 613 | cotinine | 23 µM | HL60 | 0 | 0.094 | 0.091 | 2011 |
| 1496 | 748 | vidarabine | 15 µM | MCF7 | 0 | 0.094 | 0.055 | 7203 |
| 1497 | 60 | trichostatin A | 100 nM | PC3 | 0 | 0.094 | 0.106 | 448 |
| 1498 | 699 | clemizole | 11 µM | MCF7 | 0 | 0.094 | 0.107 | 4695 |
| 1499 | 660 | urapidil | 9 µM | HL60 | 0 | 0.094 | 0.176 | 3078 |
| 1500 | 107 | 4,5-dianilinophthalimide | 10 µM | MCF7 | 0 | 0.094 | 0.079 | 624 |
| 1501 | 672 | benzthiazide | 9 µM | MCF7 | 0 | 0.094 | 0.072 | 3329 |
| 1502 | 646 | benzydamine | 12 µM | MCF7 | 0 | 0.094 | 0.139 | 3169 |
| 1503 | 725 | valproic acid | 50 µM | MCF7 | 0 | 0.094 | 0.085 | 5219 |
| 1504 | 657 | tetracaine | 13 µM | MCF7 | 0 | 0.094 | 0.09 | 2888 |
| 1505 | 653 | ketoconazole | 8 µM | MCF7 | 0 | 0.094 | 0.146 | 2640 |
| 1506 | 755 | butyl hydroxybenzoate | 21 µM | MCF7 | 0 | 0.094 | 0.088 | 6446 |
| 1507 | 750 | alvespimycin | 100 nM | HL60 | 0 | 0.094 | 0.205 | 6172 |
| 1508 | 618 | mephenesin | 22 µM | HL60 | 0 | 0.094 | 0.124 | 2342 |
| 1509 | 502 | 5252917 | 14 µM | MCF7 | 0 | 0.094 | 0.089 | 944 |
| 1510 | 670 | fluocinonide | 8 µM | MCF7 | 0 | 0.094 | 0.09 | 3414 |
| 1511 | 611 | edrophonium chloride | 20 µM | PC3 | 0 | 0.094 | 0.126 | 1936 |
| 1512 | 506 | vorinostat | 10 µM | MCF7 | 0 | 0.094 | 0.079 | 1000 |
| 1513 | 691 | trimipramine | 10 µM | MCF7 | 0 | 0.094 | 0.118 | 4163 |
| 1514 | 661 | trichostatin A | 100 nM | HL60 | 0 | 0.094 | 0.14 | 3114 |
| 1515 | 1095 | trichostatin A | 1 µM | PC3 | 0 | 0.094 | 0.13 | 7555 |
| 1516 | 687 | Prestwick-1100 | 9 µM | MCF7 | 0 | 0.094 | 0.092 | 3880 |
| 1517 | 726 | trichostatin A | 100 nM | MCF7 | 0 | 0.094 | 0.094 | 5260 |
| 1518 | 671 | cefoxitin | 9 µM | MCF7 | 0 | 0.094 | 0.102 | 3477 |
| 1519 | 648 | estropipate | 9 µM | HL60 | 0 | 0.094 | 0.156 | 2506 |
| 1520 | 504 | trichostatin A | 1 µM | MCF7 | 0 | 0.094 | 0.086 | 873 |
| 1521 | 748 | nadide | 6 µM | MCF7 | 0 | 0.093 | 0.074 | 7227 |
| 1522 | 762 | moracizine | 9 µM | PC3 | 0 | 0.093 | 0.077 | 7297 |
| 1523 | 635 | tobramycin | 9 µM | HL60 | 0 | 0.093 | 0.14 | 2481 |
| 1524 | 644 | bicuculline | 11 µM | HL60 | 0 | 0.093 | 0.118 | 2139 |
| 1525 | 751 | citiolone | 25 µM | MCF7 | 0 | 0.093 | 0.098 | 6031 |
| 1526 | 502 | 5211181 | 12 µM | MCF7 | 0 | 0.093 | 0.13 | 950 |
| 1527 | 752 | delsoline | 9 µM | MCF7 | 0 | 0.093 | 0.067 | 6075 |
| 1528 | 648 | kinetin | 19 µM | HL60 | 0 | 0.093 | 0.147 | 2511 |
| 1529 | 1062 | scriptaid | 10 µM | PC3 | 0 | 0.093 | 0.114 | 6919 |
| 1530 | 1006 | H-7 | 100 µM | MCF7 | 0 | 0.093 | 0.135 | 5936 |
| 1531 | 750 | trifluoperazine | 10 µM | HL60 | 0 | 0.093 | 0.143 | 6183 |
| 1532 | 623 | clopamide | 12 µM | HL60 | 0 | 0.093 | 0.09 | 1605 |
| 1533 | 623 | clenbuterol | 13 µM | HL60 | 0 | 0.093 | 0.094 | 1613 |
| 1534 | 671 | amrinone | 21 µM | MCF7 | 0 | 0.093 | 0.094 | 3465 |
| 1535 | 708 | practolol | 15 µM | MCF7 | 0 | 0.093 | 0.099 | 5664 |
| 1536 | 653 | oxantel | 7 µM | MCF7 | 0 | 0.093 | 0.092 | 2632 |
| 1537 | 718 | homatropine | 11 µM | PC3 | 0 | 0.093 | 0.066 | 5058 |
| 1538 | 757 | valproic acid | 200 µM | MCF7 | 0 | 0.093 | 0.08 | 5574 |
| 1539 | 693 | quercetin | 12 µM | PC3 | 0 | 0.093 | 0.057 | 4264 |
| 1540 | 631 | pepstatin | 6 µM | HL60 | 0 | 0.093 | 0.121 | 1328 |
| 1541 | 713 | meclozine | 9 µM | PC3 | 0 | 0.092 | 0.092 | 4646 |
| 1542 | 750 | alpha-estradiol | 10 nM | HL60 | 0 | 0.092 | 0.199 | 6169 |
| 1543 | 748 | trichostatin A | 100 nM | MCF7 | 0 | 0.092 | 0.108 | 7236 |
| 1544 | 736 | acemetacin | 10 µM | MCF7 | 0 | 0.092 | 0.066 | 5460 |
| 1545 | 95 | resveratrol | 50 µM | MCF7 | 0 | 0.092 | 0.074 | 595 |
| 1546 | 650 | tanespimycin | 1 µM | HL60 | 0 | 0.092 | 0.097 | 2678 |
| 1547 | 646 | mepacrine | 8 µM | MCF7 | 0 | 0.092 | 0.141 | 3179 |
| 1548 | 619 | norethisterone | 13 µM | HL60 | 0 | 0.092 | 0.16 | 2383 |
| 1549 | 506 | sirolimus | 100 nM | MCF7 | 0 | 0.092 | 0.086 | 1001 |
| 1550 | 730 | adenosine phosphate | 11 µM | MCF7 | 0 | 0.092 | 0.071 | 5359 |
| 1551 | 749 | thiethylperazine | 6 µM | HL60 | 0 | 0.092 | 0.105 | 6154 |
| 1552 | 692 | nystatin | 4 µM | PC3 | 0 | 0.092 | 0.079 | 4223 |
| 1553 | 711 | fluocinonide | 8 µM | MCF7 | 0 | 0.092 | 0.098 | 3933 |
| 1554 | 727 | fulvestrant | 1 µM | PC3 | 0 | 0.092 | 0.132 | 4429 |
| 1555 | 665 | iopanoic acid | 7 µM | HL60 | 0 | 0.092 | 0.221 | 2965 |
| 1556 | 505 | HC toxin | 100 nM | MCF7 | 0 | 0.092 | 0.138 | 909 |
| 1557 | 714 | chlorprothixene | 11 µM | PC3 | 0 | 0.092 | 0.104 | 6692 |
| 1558 | 749 | cyclopentolate | 12 µM | HL60 | 0 | 0.092 | 0.111 | 6132 |
| 1559 | 513 | trichostatin A | 100 nM | MCF7 | 0 | 0.092 | 0.099 | 1050 |
| 1560 | 749 | verteporfin | 3 µM | HL60 | 0 | 0.092 | 0.226 | 6133 |
| 1561 | 766 | trichostatin A | 100 nM | MCF7 | 0 | 0.092 | 0.1 | 7005 |
| 1562 | 757 | valproic acid | 1 mM | MCF7 | 0 | 0.092 | 0.079 | 5569 |
| 1563 | 611 | moroxydine | 19 µM | PC3 | 0 | 0.092 | 0.05 | 1944 |
| 1564 | 755 | tonzonium bromide | 7 µM | MCF7 | 0 | 0.092 | 0.078 | 6457 |
| 1565 | 636 | pindolol | 16 µM | MCF7 | 0 | 0.092 | 0.081 | 2238 |
| 1566 | 711 | PHA-00851261E | 10 µM | MCF7 | 0 | 0.092 | 0.062 | 3965 |
| 1567 | 44 | valproic acid | 1 mM | HL60 | 0 | 0.091 | 0.06 | 409 |
| 1568 | 664 | cefalonium | 9 µM | HL60 | 0 | 0.091 | 0.176 | 2921 |
| 1569 | 750 | fulvestrant | 1 µM | HL60 | 0 | 0.091 | 0.189 | 6165 |
| 1570 | 671 | cefapirin | 9 µM | MCF7 | 0 | 0.091 | 0.112 | 3471 |
| 1571 | 755 | flurbiprofen | 16 µM | MCF7 | 0 | 0.091 | 0.068 | 6472 |
| 1572 | 701 | phenformin | 17 µM | PC3 | 0 | 0.091 | 0.129 | 4283 |
| 1573 | 611 | azacyclonol | 15 µM | PC3 | 0 | 0.091 | 0.097 | 1937 |
| 1574 | 1066 | SB-202190 | 1 µM | MCF7 | 0 | 0.091 | 0.108 | 7054 |
| 1575 | 612 | naphazoline | 16 µM | HL60 | 0 | 0.091 | 0.1 | 1966 |
| 1576 | 645 | foliosidine | 13 µM | HL60 | 0 | 0.091 | 0.103 | 2201 |
| 1577 | 737 | diprophylline | 16 µM | MCF7 | 0 | 0.091 | 0.067 | 5482 |
| 1578 | 755 | fluspirilene | 8 µM | MCF7 | 0 | 0.091 | 0.127 | 6463 |
| 1579 | 705 | vitexin | 9 µM | MCF7 | 0 | 0.091 | 0.102 | 4413 |
| 1580 | 753 | verapamil | 8 µM | PC3 | 0 | 0.091 | 0.083 | 6287 |
| 1581 | 678 | cycloserine | 39 µM | MCF7 | 0 | 0.091 | 0.082 | 3562 |
| 1582 | 692 | zalcitabine | 19 µM | PC3 | 0 | 0.091 | 0.07 | 4215 |
| 1583 | 611 | chlorhexidine | 8 µM | PC3 | 0 | 0.09 | 0.064 | 1942 |
| 1584 | 692 | propantheline bromide | 9 µM | PC3 | 0 | 0.09 | 0.053 | 4214 |
| 1585 | 502 | resveratrol | 10 µM | MCF7 | 0 | 0.09 | 0.091 | 958 |
| 1586 | 661 | remoxipride | 10 µM | HL60 | 0 | 0.09 | 0.121 | 3124 |
| 1587 | 649 | dicoumarol | 12 µM | HL60 | 0 | 0.09 | 0.132 | 2561 |
| 1588 | 658 | paromomycin | 6 µM | HL60 | 0 | 0.09 | 0.09 | 3017 |
| 1589 | 505 | 5114445 | 10 µM | MCF7 | 0 | 0.09 | 0.062 | 901 |
| 1590 | 671 | trichostatin A | 100 nM | MCF7 | 0 | 0.09 | 0.102 | 3462 |
| 1591 | 710 | (+)-isoprenaline | 11 µM | PC3 | 0 | 0.09 | 0.072 | 6663 |
| 1592 | 634 | neostigmine bromide | 13 µM | HL60 | 0 | 0.09 | 0.134 | 2432 |
| 1593 | 1017 | 3-nitropropionic acid | 10 µM | PC3 | 0 | 0.09 | 0.042 | 6372 |
| 1594 | 502 | quinostatin | 10 µM | MCF7 | 0 | 0.09 | 0.094 | 973 |
| 1595 | 761 | isoxicam | 12 µM | PC3 | 0 | 0.09 | 0.074 | 7268 |
| 1596 | 502 | 5253409 | 17 µM | MCF7 | 0 | 0.09 | 0.136 | 961 |
| 1597 | 736 | ranitidine | 11 µM | MCF7 | 0 | 0.09 | 0.142 | 5425 |
| 1598 | 680 | salbutamol | 17 µM | PC3 | 0 | 0.09 | 0.065 | 3677 |
| 1599 | 640 | thiamine | 12 µM | HL60 | 0 | 0.09 | 0.09 | 1744 |
| 1600 | 750 | genistein | 10 µM | HL60 | 0 | 0.09 | 0.19 | 6194 |
| 1601 | 654 | chlorogenic acid | 11 µM | MCF7 | 0 | 0.09 | 0.062 | 3282 |
| 1602 | 670 | cloperastine | 11 µM | MCF7 | 0 | 0.09 | 0.09 | 3408 |
| 1603 | 630 | amoxicillin | 11 µM | HL60 | 0 | 0.09 | 0.127 | 1265 |
| 1604 | 670 | bendroflumethiazide | 9 µM | MCF7 | 0 | 0.09 | 0.072 | 3415 |
| 1605 | 660 | cantharidin | 20 µM | HL60 | 0 | 0.09 | 0.183 | 3075 |
| 1606 | 1066 | 0316684-0000 | 10 µM | MCF7 | 0 | 0.089 | 0.092 | 7052 |
| 1607 | 772 | dirithromycin | 5 µM | MCF7 | 0 | 0.089 | 0.067 | 7446 |
| 1608 | 645 | pempidine | 13 µM | HL60 | 0 | 0.089 | 0.15 | 2172 |
| 1609 | 631 | lysergol | 16 µM | HL60 | 0 | 0.089 | 0.118 | 1325 |
| 1610 | 664 | zardaverine | 15 µM | HL60 | 0 | 0.089 | 0.188 | 2926 |
| 1611 | 688 | oxamniquine | 14 µM | PC3 | 0 | 0.089 | 0.067 | 4006 |
| 1612 | 665 | Prestwick-1083 | 9 µM | HL60 | 0 | 0.089 | 0.2 | 2976 |
| 1613 | 677 | S-propranolol | 14 µM | MCF7 | 0 | 0.089 | 0.096 | 3523 |
| 1614 | 657 | raubasine | 10 µM | MCF7 | 0 | 0.089 | 0.135 | 2898 |
| 1615 | 8 | tacrolimus | 1 µM | MCF7 | 0 | 0.089 | 0.084 | 169 |
| 1616 | 626 | chlorpromazine | 1 µM | MCF7 | 0 | 0.089 | 0.06 | 1642 |
| 1617 | 673 | dimethadione | 31 µM | MCF7 | 0 | 0.089 | 0.095 | 3367 |
| 1618 | 691 | solanine | 5 µM | MCF7 | 0 | 0.089 | 0.083 | 4166 |
| 1619 | 750 | valproic acid | 500 µM | HL60 | 0 | 0.089 | 0.143 | 6199 |
| 1620 | 635 | propylthiouracil | 23 µM | HL60 | 0 | 0.089 | 0.126 | 2476 |
| 1621 | 749 | dorzolamide | 11 µM | HL60 | 0 | 0.089 | 0.091 | 6142 |
| 1622 | 700 | metoclopramide | 12 µM | MCF7 | 0 | 0.089 | 0.098 | 4750 |
| 1623 | 743 | nocodazole | 13 µM | MCF7 | 0 | 0.089 | 0.059 | 6793 |
| 1624 | 658 | roxithromycin | 5 µM | HL60 | 0 | 0.089 | 0.159 | 2992 |
| 1625 | 686 | acacetin | 14 µM | MCF7 | 0 | 0.089 | 0.055 | 3849 |
| 1626 | 672 | Prestwick-864 | 35 µM | MCF7 | 0 | 0.089 | 0.058 | 3333 |
| 1627 | 1008 | rotenone | 1 µM | MCF7 | 0 | 0.089 | 0.118 | 5943 |
| 1628 | 505 | 5152487 | 10 µM | MCF7 | 0 | 0.089 | 0.043 | 896 |
| 1629 | 655 | ciclopirox | 15 µM | MCF7 | 0 | 0.089 | 0.09 | 3317 |
| 1630 | 635 | tolazamide | 13 µM | HL60 | 0 | 0.089 | 0.139 | 2482 |
| 1631 | 1079 | trichostatin A | 1 µM | PC3 | 0 | 0.089 | 0.135 | 7105 |
| 1632 | 610 | dicycloverine | 12 µM | PC3 | 0 | 0.089 | 0.074 | 1902 |
| 1633 | 700 | Prestwick-664 | 8 µM | MCF7 | 0 | 0.088 | 0.054 | 4737 |
| 1634 | 736 | raubasine | 10 µM | MCF7 | 0 | 0.088 | 0.071 | 5459 |
| 1635 | 678 | dexpropranolol | 14 µM | MCF7 | 0 | 0.088 | 0.064 | 3553 |
| 1636 | 1034 | 5194442 | 20 µM | PC3 | 0 | 0.088 | 0.092 | 6553 |
| 1637 | 1090 | 11-deoxy-16,16-dimethylprostaglandin E2 | 10 µM | MCF7 | 0 | 0.088 | 0.123 | 7533 |
| 1638 | 658 | fosfosal | 18 µM | HL60 | 0 | 0.088 | 0.143 | 2997 |
| 1639 | 752 | fluorocurarine | 12 µM | MCF7 | 0 | 0.088 | 0.068 | 6083 |
| 1640 | 1032 | trichostatin A | 1 µM | PC3 | 0 | 0.088 | 0.125 | 6546 |
| 1641 | 757 | fulvestrant | 10 nM | MCF7 | 0 | 0.088 | 0.107 | 5598 |
| 1642 | 502 | carbamazepine | 100 nM | MCF7 | 0 | 0.088 | 0.11 | 952 |
| 1643 | 613 | trichostatin A | 100 nM | HL60 | 0 | 0.088 | 0.11 | 2035 |
| 1644 | 611 | azathioprine | 14 µM | PC3 | 0 | 0.088 | 0.071 | 1945 |
| 1645 | 612 | pyrimethamine | 16 µM | HL60 | 0 | 0.088 | 0.1 | 1974 |
| 1646 | 618 | dantrolene | 12 µM | HL60 | 0 | 0.088 | 0.144 | 2369 |
| 1647 | 751 | eldeline | 8 µM | MCF7 | 0 | 0.088 | 0.085 | 6026 |
| 1648 | 678 | trichostatin A | 100 nM | MCF7 | 0 | 0.088 | 0.08 | 3566 |
| 1649 | 755 | dexibuprofen | 19 µM | MCF7 | 0 | 0.088 | 0.069 | 6471 |
| 1650 | 506 | fluphenazine | 10 µM | MCF7 | 0 | 0.088 | 0.105 | 1017 |
| 1651 | 672 | iocetamic acid | 7 µM | MCF7 | 0 | 0.088 | 0.089 | 3361 |
| 1652 | 756 | isometheptene | 8 µM | MCF7 | 0 | 0.088 | 0.051 | 6524 |
| 1653 | 28 | trichostatin A | 100 nM | MCF7 | 0 | 0.088 | 0.087 | 331 |
| 1654 | 20 | clofibrate | 150 µM | MCF7 | 0 | 0.088 | 0.075 | 263 |
| 1655 | 659 | phthalylsulfathiazole | 10 µM | HL60 | 0 | 0.088 | 0.189 | 3033 |
| 1656 | 706 | tracazolate | 12 µM | MCF7 | 0 | 0.088 | 0.065 | 4964 |
| 1657 | 699 | mephentermine | 9 µM | MCF7 | 0 | 0.088 | 0.106 | 4707 |
| 1658 | 612 | niclosamide | 12 µM | HL60 | 0 | 0.088 | 0.155 | 1998 |
| 1659 | 690 | Prestwick-864 | 35 µM | MCF7 | 0 | 0.088 | 0.089 | 4113 |
| 1660 | 504 | pyrvinium | 1 µM | MCF7 | 0 | 0.088 | 0.074 | 870 |
| 1661 | 765 | trichostatin A | 1 µM | MCF7 | 0 | 0.088 | 0.112 | 6993 |
| 1662 | 610 | metronidazole | 23 µM | PC3 | 0 | 0.087 | 0.11 | 1921 |
| 1663 | 662 | epivincamine | 11 µM | MCF7 | 0 | 0.087 | 0.089 | 2775 |
| 1664 | 648 | fluorocurarine | 12 µM | HL60 | 0 | 0.087 | 0.115 | 2521 |
| 1665 | 757 | trichostatin A | 1 µM | MCF7 | 0 | 0.087 | 0.102 | 5594 |
| 1666 | 660 | meglumine | 20 µM | HL60 | 0 | 0.087 | 0.129 | 3068 |
| 1667 | 694 | conessine | 11 µM | MCF7 | 0 | 0.087 | 0.098 | 4777 |
| 1668 | 673 | profenamine | 11 µM | MCF7 | 0 | 0.087 | 0.104 | 3376 |
| 1669 | 637 | oxymetazoline | 13 µM | MCF7 | 0 | 0.087 | 0.05 | 2278 |
| 1670 | 765 | valproic acid | 50 µM | MCF7 | 0 | 0.087 | 0.064 | 6982 |
| 1671 | 602 | tretinoin | 1 µM | HL60 | 0 | 0.087 | 0.12 | 1152 |
| 1672 | 635 | cetirizine | 9 µM | HL60 | 0 | 0.087 | 0.163 | 2468 |
| 1673 | 502 | 5255229 | 13 µM | MCF7 | 0 | 0.087 | 0.087 | 949 |
| 1674 | 693 | trichostatin A | 100 nM | PC3 | 0 | 0.087 | 0.176 | 4237 |
| 1675 | 1030 | trichostatin A | 1 µM | MCF7 | 0 | 0.087 | 0.083 | 6434 |
| 1676 | 748 | abamectin | 5 µM | MCF7 | 0 | 0.087 | 0.115 | 7218 |
| 1677 | 772 | ajmaline | 12 µM | MCF7 | 0 | 0.087 | 0.051 | 7484 |
| 1678 | 610 | tolazoline | 20 µM | PC3 | 0 | 0.087 | 0.08 | 1918 |
| 1679 | 502 | oxaprozin | 300 µM | MCF7 | 0 | 0.087 | 0.095 | 971 |
| 1680 | 729 | apomorphine | 6 µM | MCF7 | 0 | 0.087 | 0.074 | 5283 |
| 1681 | 661 | nabumetone | 18 µM | HL60 | 0 | 0.087 | 0.111 | 3108 |
| 1682 | 618 | nefopam | 14 µM | HL60 | 0 | 0.087 | 0.092 | 2355 |
| 1683 | 682 | phentolamine | 13 µM | PC3 | 0 | 0.087 | 0.046 | 3779 |
| 1684 | 714 | trichostatin A | 100 nM | PC3 | 0 | 0.087 | 0.131 | 6709 |
| 1685 | 762 | econazole | 9 µM | PC3 | 0 | 0.087 | 0.084 | 7305 |
| 1686 | 705 | levodopa | 20 µM | MCF7 | 0 | 0.087 | 0.058 | 4394 |
| 1687 | 630 | benfluorex | 10 µM | HL60 | 0 | 0.087 | 0.164 | 1266 |
| 1688 | 766 | mesoridazine | 7 µM | MCF7 | 0 | 0.087 | 0.069 | 7017 |
| 1689 | 688 | carbarsone | 15 µM | PC3 | 0 | 0.087 | 0.08 | 3991 |
| 1690 | 658 | suprofen | 15 µM | HL60 | 0 | 0.087 | 0.066 | 3005 |
| 1691 | 506 | trichostatin A | 100 nM | MCF7 | 0 | 0.087 | 0.11 | 992 |
| 1692 | 661 | alfaxalone | 12 µM | HL60 | 0 | 0.087 | 0.109 | 3135 |
| 1693 | 653 | mimosine | 20 µM | MCF7 | 0 | 0.087 | 0.09 | 2638 |
| 1694 | 613 | diltiazem | 9 µM | HL60 | 0 | 0.086 | 0.091 | 2032 |
| 1695 | 751 | trichostatin A | 100 nM | MCF7 | 0 | 0.086 | 0.098 | 6064 |
| 1696 | 709 | carisoprodol | 15 µM | PC3 | 0 | 0.086 | 0.073 | 6610 |
| 1697 | 513 | fulvestrant | 10 nM | MCF7 | 0 | 0.086 | 0.068 | 1076 |
| 1698 | 753 | ganciclovir | 16 µM | PC3 | 0 | 0.086 | 0.082 | 6289 |
| 1699 | 734 | nadide | 6 µM | PC3 | 0 | 0.086 | 0.056 | 5873 |
| 1700 | 644 | sparteine | 17 µM | HL60 | 0 | 0.086 | 0.09 | 2134 |
| 1701 | 1049 | PF-00562151-00 | 10 µM | MCF7 | 0 | 0.086 | 0.07 | 6868 |
| 1702 | 619 | bufexamac | 18 µM | HL60 | 0 | 0.086 | 0.093 | 2382 |
| 1703 | 1005 | sirolimus | 100 nM | PC3 | 0 | 0.086 | 0.091 | 5932 |
| 1704 | 725 | nordihydroguaiaretic acid | 1 µM | MCF7 | 0 | 0.086 | 0.06 | 5220 |
| 1705 | 765 | valproic acid | 200 µM | MCF7 | 0 | 0.086 | 0.083 | 6974 |
| 1706 | 646 | clonidine | 15 µM | MCF7 | 0 | 0.086 | 0.065 | 3172 |
| 1707 | 642 | trimethoprim | 14 µM | MCF7 | 0 | 0.086 | 0.079 | 2307 |
| 1708 | 686 | metamizole sodium | 12 µM | MCF7 | 0 | 0.086 | 0.082 | 3835 |
| 1709 | 695 | quercetin | 12 µM | MCF7 | 0 | 0.086 | 0.134 | 4846 |
| 1710 | 733 | trichostatin A | 100 nM | PC3 | 0 | 0.086 | 0.139 | 5822 |
| 1711 | 505 | sirolimus | 100 nM | MCF7 | 0 | 0.086 | 0.067 | 921 |
| 1712 | 767 | fulvestrant | 1 µM | MCF7 | 0 | 0.086 | 0.126 | 6925 |
| 1713 | 670 | deferoxamine | 6 µM | MCF7 | 0 | 0.086 | 0.086 | 3417 |
| 1714 | 690 | carbarsone | 15 µM | MCF7 | 0 | 0.086 | 0.124 | 4110 |
| 1715 | 719 | hyoscyamine | 14 µM | PC3 | 0 | 0.086 | 0.088 | 5099 |
| 1716 | 17 | tretinoin | 1 µM | MCF7 | 0 | 0.086 | 0.088 | 224 |
| 1717 | 623 | dobutamine | 12 µM | HL60 | 0 | 0.086 | 0.116 | 1589 |
| 1718 | 764 | oxybutynin | 10 µM | PC3 | 0 | 0.086 | 0.109 | 7126 |
| 1719 | 618 | mexiletine | 19 µM | HL60 | 0 | 0.086 | 0.101 | 2364 |
| 1720 | 670 | sulfamethoxypyridazine | 14 µM | MCF7 | 0 | 0.086 | 0.105 | 3409 |
| 1721 | 44 | sodium phenylbutyrate | 1 mM | HL60 | 0 | 0.086 | 0.118 | 411 |
| 1722 | 603 | thioridazine | 10 µM | PC3 | 0 | 0.085 | 0.105 | 1230 |
| 1723 | 630 | cefalexin | 11 µM | HL60 | 0 | 0.085 | 0.18 | 1273 |
| 1724 | 628 | betazole | 27 µM | PC3 | 0 | 0.085 | 0.061 | 1812 |
| 1725 | 653 | clebopride | 8 µM | MCF7 | 0 | 0.085 | 0.081 | 2646 |
| 1726 | 738 | mometasone | 8 µM | MCF7 | 0 | 0.085 | 0.091 | 5541 |
| 1727 | 663 | hydroxyachillin | 14 µM | MCF7 | 0 | 0.085 | 0.087 | 2812 |
| 1728 | 653 | testosterone | 12 µM | MCF7 | 0 | 0.085 | 0.118 | 2649 |
| 1729 | 709 | Chicago Sky Blue 6B | 4 µM | PC3 | 0 | 0.085 | 0.106 | 6626 |
| 1730 | 757 | prochlorperazine | 10 µM | MCF7 | 0 | 0.085 | 0.104 | 5575 |
| 1731 | 691 | metacycline | 8 µM | MCF7 | 0 | 0.085 | 0.084 | 4143 |
| 1732 | 700 | 0179445-0000 | 10 µM | MCF7 | 0 | 0.085 | 0.1 | 4755 |
| 1733 | 708 | bepridil | 10 µM | MCF7 | 0 | 0.085 | 0.093 | 5674 |
| 1734 | 662 | monocrotaline | 12 µM | MCF7 | 0 | 0.085 | 0.059 | 2749 |
| 1735 | 663 | galantamine | 11 µM | MCF7 | 0 | 0.085 | 0.081 | 2787 |
| 1736 | 757 | tanespimycin | 1 µM | MCF7 | 0 | 0.085 | 0.074 | 5586 |
| 1737 | 737 | trichostatin A | 100 nM | MCF7 | 0 | 0.085 | 0.118 | 5484 |
| 1738 | 725 | fulvestrant | 1 µM | MCF7 | 0 | 0.085 | 0.108 | 5202 |
| 1739 | 1082 | trichostatin A | 1 µM | MCF7 | 0 | 0.085 | 0.122 | 7499 |
| 1740 | 673 | trichostatin A | 100 nM | MCF7 | 0 | 0.085 | 0.128 | 3395 |
| 1741 | 630 | suloctidil | 12 µM | HL60 | 0 | 0.085 | 0.111 | 1297 |
| 1742 | 749 | natamycin | 6 µM | HL60 | 0 | 0.085 | 0.159 | 6126 |
| 1743 | 659 | aminophylline | 10 µM | HL60 | 0 | 0.085 | 0.135 | 3036 |
| 1744 | 1024 | PF-01378883-00 | 10 µM | MCF7 | 0 | 0.085 | 0.081 | 6405 |
| 1745 | 757 | haloperidol | 10 µM | MCF7 | 0 | 0.085 | 0.065 | 5604 |
| 1746 | 685 | sulfamethoxypyridazine | 14 µM | MCF7 | 0 | 0.085 | 0.099 | 3609 |
| 1747 | 619 | difenidol | 12 µM | HL60 | 0 | 0.085 | 0.111 | 2374 |
| 1748 | 673 | 2-aminobenzenesulfonamide | 23 µM | MCF7 | 0 | 0.085 | 0.111 | 3400 |
| 1749 | 765 | troglitazone | 10 µM | MCF7 | 0 | 0.085 | 0.101 | 6991 |
| 1750 | 629 | imipramine | 13 µM | HL60 | 0 | 0.085 | 0.147 | 1849 |
| 1751 | 736 | amitriptyline | 13 µM | MCF7 | 0 | 0.085 | 0.136 | 5453 |
| 1752 | 651 | sulfametoxydiazine | 14 µM | HL60 | 0 | 0.084 | 0.138 | 2712 |
| 1753 | 645 | chlorcyclizine | 12 µM | HL60 | 0 | 0.084 | 0.093 | 2197 |
| 1754 | 718 | norethisterone | 13 µM | PC3 | 0 | 0.084 | 0.076 | 5055 |
| 1755 | 664 | tracazolate | 12 µM | HL60 | 0 | 0.084 | 0.23 | 2919 |
| 1756 | 707 | etoposide | 7 µM | MCF7 | 0 | 0.084 | 0.061 | 5027 |
| 1757 | 708 | altretamine | 19 µM | MCF7 | 0 | 0.084 | 0.083 | 5688 |
| 1758 | 664 | zalcitabine | 19 µM | HL60 | 0 | 0.084 | 0.158 | 2932 |
| 1759 | 749 | cycloserine | 39 µM | HL60 | 0 | 0.084 | 0.123 | 6139 |
| 1760 | 757 | valproic acid | 50 µM | MCF7 | 0 | 0.084 | 0.074 | 5582 |
| 1761 | 603 | valproic acid | 500 µM | PC3 | 0 | 0.084 | 0.116 | 1240 |
| 1762 | 728 | stachydrine | 22 µM | PC3 | 0 | 0.084 | 0.109 | 4469 |
| 1763 | 603 | valproic acid | 200 µM | PC3 | 0 | 0.084 | 0.087 | 1214 |
| 1764 | 662 | tomatidine | 10 µM | MCF7 | 0 | 0.084 | 0.094 | 2746 |
| 1765 | 657 | gliclazide | 12 µM | MCF7 | 0 | 0.084 | 0.121 | 2870 |
| 1766 | 650 | trichostatin A | 1 µM | HL60 | 0 | 0.084 | 0.168 | 2694 |
| 1767 | 757 | tretinoin | 1 µM | MCF7 | 0 | 0.084 | 0.055 | 5571 |
| 1768 | 502 | trichostatin A | 1 µM | MCF7 | 0 | 0.084 | 0.102 | 981 |
| 1769 | 630 | bezafibrate | 11 µM | HL60 | 0 | 0.084 | 0.159 | 1275 |
| 1770 | 676 | metacycline | 8 µM | MCF7 | 0 | 0.084 | 0.069 | 7321 |
| 1771 | 659 | fluvastatin | 9 µM | HL60 | 0 | 0.084 | 0.121 | 3032 |
| 1772 | 756 | deptropine | 8 µM | MCF7 | 0 | 0.084 | 0.078 | 6523 |
| 1773 | 656 | thiostrepton | 2 µM | MCF7 | 0 | 0.084 | 0.057 | 2823 |
| 1774 | 713 | equilin | 15 µM | PC3 | 0 | 0.084 | 0.088 | 4659 |
| 1775 | 647 | kanamycin | 7 µM | MCF7 | 0 | 0.084 | 0.118 | 3224 |
| 1776 | 644 | protoveratrine A | 5 µM | HL60 | 0 | 0.084 | 0.133 | 2144 |
| 1777 | 656 | orciprenaline | 8 µM | MCF7 | 0 | 0.084 | 0.084 | 2845 |
| 1778 | 719 | resveratrol | 18 µM | PC3 | 0 | 0.084 | 0.088 | 5084 |
| 1779 | 765 | sirolimus | 100 nM | MCF7 | 0 | 0.084 | 0.109 | 6967 |
| 1780 | 650 | alpha-estradiol | 10 nM | HL60 | 0 | 0.084 | 0.121 | 2670 |
| 1781 | 761 | alfadolone | 10 µM | PC3 | 0 | 0.084 | 0.107 | 7262 |
| 1782 | 699 | trichostatin A | 100 nM | MCF7 | 0 | 0.084 | 0.08 | 4710 |
| 1783 | 634 | dicloxacillin | 8 µM | HL60 | 0 | 0.084 | 0.159 | 2445 |
| 1784 | 765 | fulvestrant | 1 µM | MCF7 | 0 | 0.083 | 0.103 | 6965 |
| 1785 | 735 | aminophylline | 10 µM | MCF7 | 0 | 0.083 | 0.09 | 5395 |
| 1786 | 658 | mestranol | 13 µM | HL60 | 0 | 0.083 | 0.134 | 3008 |
| 1787 | 714 | monobenzone | 20 µM | PC3 | 0 | 0.083 | 0.06 | 6713 |
| 1788 | 646 | glibenclamide | 8 µM | MCF7 | 0 | 0.083 | 0.071 | 3163 |
| 1789 | 767 | sirolimus | 100 nM | MCF7 | 0 | 0.083 | 0.129 | 6927 |
| 1790 | 663 | seneciphylline | 12 µM | MCF7 | 0 | 0.083 | 0.096 | 2797 |
| 1791 | 655 | trichostatin A | 100 nM | MCF7 | 0 | 0.083 | 0.106 | 3312 |
| 1792 | 1061 | SC-560 | 10 µM | MCF7 | 0 | 0.083 | 0.044 | 6913 |
| 1793 | 687 | trichostatin A | 100 nM | MCF7 | 0 | 0.083 | 0.113 | 3872 |
| 1794 | 107 | resveratrol | 50 µM | MCF7 | 0 | 0.083 | 0.077 | 622 |
| 1795 | 755 | urapidil | 9 µM | MCF7 | 0 | 0.083 | 0.087 | 6455 |
| 1796 | 707 | ketanserin | 7 µM | MCF7 | 0 | 0.083 | 0.084 | 4995 |
| 1797 | 651 | pipenzolate bromide | 9 µM | HL60 | 0 | 0.083 | 0.12 | 2719 |
| 1798 | 757 | fluphenazine | 10 µM | MCF7 | 0 | 0.083 | 0.11 | 5597 |
| 1799 | 619 | flavoxate | 9 µM | HL60 | 0 | 0.083 | 0.092 | 2373 |
| 1800 | 506 | valproic acid | 1 mM | MCF7 | 0 | 0.083 | 0.133 | 989 |
| 1801 | 715 | benfluorex | 10 µM | PC3 | 0 | 0.083 | 0.064 | 6727 |
| 1802 | 632 | oxybuprocaine | 12 µM | MCF7 | 0 | 0.083 | 0.102 | 1476 |
| 1803 | 664 | gibberellic acid | 12 µM | HL60 | 0 | 0.083 | 0.189 | 2910 |
| 1804 | 729 | florfenicol | 11 µM | MCF7 | 0 | 0.083 | 0.079 | 5300 |
| 1805 | 634 | butoconazole | 8 µM | HL60 | 0 | 0.083 | 0.11 | 2427 |
| 1806 | 504 | carbamazepine | 100 nM | MCF7 | 0 | 0.083 | 0.09 | 835 |
| 1807 | 663 | quinisocaine | 13 µM | MCF7 | 0 | 0.083 | 0.067 | 2807 |
| 1808 | 619 | dyclonine | 12 µM | HL60 | 0 | 0.083 | 0.117 | 2392 |
| 1809 | 645 | ethotoin | 20 µM | HL60 | 0 | 0.083 | 0.194 | 2196 |
| 1810 | 719 | enilconazole | 13 µM | PC3 | 0 | 0.082 | 0.096 | 5113 |
| 1811 | 693 | buflomedil | 12 µM | PC3 | 0 | 0.082 | 0.11 | 4258 |
| 1812 | 659 | sulconazole | 9 µM | HL60 | 0 | 0.082 | 0.108 | 3035 |
| 1813 | 603 | tretinoin | 1 µM | PC3 | 0 | 0.082 | 0.06 | 1211 |
| 1814 | 748 | corbadrine | 22 µM | MCF7 | 0 | 0.082 | 0.088 | 7208 |
| 1815 | 603 | sirolimus | 100 nM | PC3 | 0 | 0.082 | 0.057 | 1242 |
| 1816 | 681 | fenbufen | 16 µM | PC3 | 0 | 0.082 | 0.113 | 3721 |
| 1817 | 16 | SC-58125 | 10 µM | MCF7 | 0 | 0.082 | 0.061 | 208 |
| 1818 | 767 | valproic acid | 1 mM | MCF7 | 0 | 0.082 | 0.079 | 6929 |
| 1819 | 618 | indapamide | 11 µM | HL60 | 0 | 0.082 | 0.136 | 2361 |
| 1820 | 731 | syrosingopine | 6 µM | PC3 | 0 | 0.082 | 0.133 | 5733 |
| 1821 | 680 | isocarboxazid | 17 µM | PC3 | 0 | 0.082 | 0.061 | 3684 |
| 1822 | 702 | PHA-00851261E | 10 µM | PC3 | 0 | 0.082 | 0.077 | 4330 |
| 1823 | 649 | medrysone | 12 µM | HL60 | 0 | 0.082 | 0.216 | 2544 |
| 1824 | 651 | gabapentin | 23 µM | HL60 | 0 | 0.082 | 0.07 | 2731 |
| 1825 | 1077 | SC-19220 | 10 µM | PC3 | 0 | 0.082 | 0.042 | 7095 |
| 1826 | 614 | trichostatin A | 100 nM | HL60 | 0 | 0.082 | 0.113 | 1400 |
| 1827 | 504 | resveratrol | 10 µM | MCF7 | 0 | 0.082 | 0.098 | 841 |
| 1828 | 513 | thioridazine | 10 µM | MCF7 | 0 | 0.082 | 0.141 | 1068 |
| 1829 | 737 | perhexiline | 10 µM | MCF7 | 0 | 0.081 | 0.099 | 5501 |
| 1830 | 603 | valproic acid | 50 µM | PC3 | 0 | 0.081 | 0.164 | 1222 |
| 1831 | 748 | pirenzepine | 9 µM | MCF7 | 0 | 0.081 | 0.116 | 7226 |
| 1832 | 665 | piribedil | 12 µM | HL60 | 0 | 0.081 | 0.208 | 2951 |
| 1833 | 629 | trichostatin A | 100 nM | HL60 | 0 | 0.081 | 0.218 | 1835 |
| 1834 | 657 | budesonide | 9 µM | MCF7 | 0 | 0.081 | 0.088 | 2866 |
| 1835 | 671 | sulfanilamide | 23 µM | MCF7 | 0 | 0.081 | 0.075 | 3449 |
| 1836 | 664 | memantine | 19 µM | HL60 | 0 | 0.081 | 0.169 | 2934 |
| 1837 | 626 | valproic acid | 200 µM | MCF7 | 0 | 0.081 | 0.081 | 1639 |
| 1838 | 670 | meclofenoxate | 14 µM | MCF7 | 0 | 0.081 | 0.1 | 3405 |
| 1839 | 727 | vorinostat | 10 µM | PC3 | 0 | 0.081 | 0.125 | 4444 |
| 1840 | 610 | cimetidine | 16 µM | PC3 | 0 | 0.081 | 0.101 | 1884 |
| 1841 | 750 | troglitazone | 10 µM | HL60 | 0 | 0.081 | 0.149 | 6191 |
| 1842 | 630 | pentoxyverine | 8 µM | HL60 | 0 | 0.081 | 0.143 | 1268 |
| 1843 | 693 | succinylsulfathiazole | 11 µM | PC3 | 0 | 0.081 | 0.095 | 4265 |
| 1844 | 623 | hycanthone | 11 µM | HL60 | 0 | 0.081 | 0.127 | 1614 |
| 1845 | 630 | thioguanosine | 13 µM | HL60 | 0 | 0.081 | 0.16 | 1264 |
| 1846 | 757 | haloperidol | 10 µM | MCF7 | 0 | 0.081 | 0.045 | 5563 |
| 1847 | 513 | trichostatin A | 1 µM | MCF7 | 0 | 0.081 | 0.131 | 1072 |
| 1848 | 642 | phentolamine | 13 µM | MCF7 | 0 | 0.081 | 0.075 | 2323 |
| 1849 | 651 | mevalolactone | 31 µM | HL60 | 0 | 0.081 | 0.172 | 2718 |
| 1850 | 755 | dioxybenzone | 16 µM | MCF7 | 0 | 0.081 | 0.047 | 6478 |
| 1851 | 745 | terbutaline | 7 µM | MCF7 | 0 | 0.081 | 0.12 | 6240 |
| 1852 | 664 | decamethonium bromide | 10 µM | HL60 | 0 | 0.081 | 0.213 | 2933 |
| 1853 | 662 | harmine | 16 µM | MCF7 | 0 | 0.081 | 0.094 | 2750 |
| 1854 | 753 | trichostatin A | 100 nM | PC3 | 0 | 0.081 | 0.181 | 6316 |
| 1855 | 707 | methapyrilene | 13 µM | MCF7 | 0 | 0.081 | 0.073 | 4990 |
| 1856 | 729 | felodipine | 10 µM | MCF7 | 0 | 0.081 | 0.067 | 5294 |
| 1857 | 678 | sertaconazole | 8 µM | MCF7 | 0 | 0.081 | 0.112 | 3550 |
| 1858 | 736 | trichostatin A | 100 nM | MCF7 | 0 | 0.081 | 0.125 | 5441 |
| 1859 | 616 | tamoxifen | 7 µM | PC3 | 0 | 0.081 | 0.056 | 2050 |
| 1860 | 35 | trichostatin A | 100 nM | HL60 | 0 | 0.081 | 0.102 | 364 |
| 1861 | 653 | flupentixol | 8 µM | MCF7 | 0 | 0.081 | 0.081 | 2643 |
| 1862 | 737 | gemfibrozil | 16 µM | MCF7 | 0 | 0.081 | 0.091 | 5488 |
| 1863 | 765 | chlorpromazine | 1 µM | MCF7 | 0 | 0.081 | 0.073 | 6977 |
| 1864 | 738 | trichostatin A | 100 nM | MCF7 | 0 | 0.081 | 0.12 | 5511 |
| 1865 | 636 | isoconazole | 10 µM | MCF7 | 0 | 0.081 | 0.076 | 2218 |
| 1866 | 687 | beta-escin | 3 µM | MCF7 | 0 | 0.081 | 0.091 | 3890 |
| 1867 | 757 | LY-294002 | 100 nM | MCF7 | 0 | 0.081 | 0.091 | 5576 |
| 1868 | 612 | sulpiride | 12 µM | HL60 | 0 | 0.08 | 0.096 | 1967 |
| 1869 | 708 | colchicine | 10 µM | MCF7 | 0 | 0.08 | 0.071 | 5675 |
| 1870 | 622 | clomipramine | 11 µM | HL60 | 0 | 0.08 | 0.172 | 1566 |
| 1871 | 678 | methanthelinium bromide | 10 µM | MCF7 | 0 | 0.08 | 0.067 | 3560 |
| 1872 | 718 | diethylcarbamazine | 10 µM | PC3 | 0 | 0.08 | 0.075 | 5066 |
| 1873 | 710 | trichostatin A | 100 nM | PC3 | 0 | 0.08 | 0.146 | 6671 |
| 1874 | 694 | colistin | 3 µM | MCF7 | 0 | 0.08 | 0.114 | 4796 |
| 1875 | 748 | pridinol | 10 µM | MCF7 | 0 | 0.08 | 0.122 | 7214 |
| 1876 | 637 | antimycin A | 7 µM | MCF7 | 0 | 0.08 | 0.097 | 2261 |
| 1877 | 707 | progesterone | 13 µM | MCF7 | 0 | 0.08 | 0.078 | 4992 |
| 1878 | 712 | perphenazine | 10 µM | PC3 | 0 | 0.08 | 0.051 | 4637 |
| 1879 | 708 | kanamycin | 7 µM | MCF7 | 0 | 0.08 | 0.06 | 5686 |
| 1880 | 751 | pramocaine | 12 µM | MCF7 | 0 | 0.08 | 0.115 | 6054 |
| 1881 | 706 | benzthiazide | 9 µM | MCF7 | 0 | 0.08 | 0.055 | 4952 |
| 1882 | 42 | wortmannin | 10 nM | ssMCF7 | 0 | 0.08 | 0.077 | 404 |
| 1883 | 619 | niflumic acid | 14 µM | HL60 | 0 | 0.08 | 0.087 | 2399 |
| 1884 | 720 | pramocaine | 12 µM | MCF7 | 0 | 0.08 | 0.112 | 4368 |
| 1885 | 664 | oxamniquine | 14 µM | HL60 | 0 | 0.08 | 0.208 | 2924 |
| 1886 | 756 | adipiodone | 4 µM | MCF7 | 0 | 0.08 | 0.071 | 6490 |
| 1887 | 651 | carbinoxamine | 10 µM | HL60 | 0 | 0.08 | 0.092 | 2725 |
| 1888 | 650 | vorinostat | 10 µM | HL60 | 0 | 0.08 | 0.124 | 2680 |
| 1889 | 751 | lanatoside C | 4 µM | MCF7 | 0 | 0.08 | 0.136 | 6048 |
| 1890 | 736 | budesonide | 9 µM | MCF7 | 0 | 0.08 | 0.09 | 5431 |
| 1891 | 755 | oxybenzone | 18 µM | MCF7 | 0 | 0.08 | 0.043 | 6469 |
| 1892 | 741 | Trolox C | 16 µM | MCF7 | 0 | 0.08 | 0.079 | 6007 |
| 1893 | 647 | ketanserin | 7 µM | MCF7 | 0 | 0.08 | 0.115 | 3209 |
| 1894 | 611 | erythromycin | 5 µM | PC3 | 0 | 0.08 | 0.034 | 1928 |
| 1895 | 741 | norfloxacin | 13 µM | MCF7 | 0 | 0.08 | 0.081 | 5985 |
| 1896 | 662 | piperlongumine | 13 µM | MCF7 | 0 | 0.08 | 0.062 | 2757 |
| 1897 | 648 | ricinine | 24 µM | HL60 | 0 | 0.08 | 0.117 | 2505 |
| 1898 | 686 | furaltadone | 11 µM | MCF7 | 0 | 0.08 | 0.206 | 3838 |
| 1899 | 1001 | trichostatin A | 1 µM | PC3 | 0 | 0.08 | 0.1 | 5908 |
| 1900 | 635 | probenecid | 14 µM | HL60 | 0 | 0.08 | 0.14 | 2464 |
| 1901 | 1053 | trichostatin A | 1 µM | MCF7 | 0 | 0.08 | 0.1 | 6891 |
| 1902 | 633 | trichostatin A | 100 nM | MCF7 | 0 | 0.08 | 0.125 | 1535 |
| 1903 | 677 | etomidate | 16 µM | MCF7 | 0 | 0.08 | 0.097 | 3519 |
| 1904 | 1043 | trichostatin A | 1 µM | MCF7 | 0 | 0.08 | 0.127 | 6579 |
| 1905 | 764 | trichostatin A | 100 nM | PC3 | 0 | 0.08 | 0.104 | 7136 |
| 1906 | 765 | nordihydroguaiaretic acid | 1 µM | MCF7 | 0 | 0.079 | 0.088 | 6983 |
| 1907 | 676 | etofenamate | 11 µM | MCF7 | 0 | 0.079 | 0.069 | 7327 |
| 1908 | 715 | oxantel | 7 µM | PC3 | 0 | 0.079 | 0.078 | 6738 |
| 1909 | 659 | Prestwick-860 | 35 µM | HL60 | 0 | 0.079 | 0.128 | 3040 |
| 1910 | 660 | (-)-atenolol | 15 µM | HL60 | 0 | 0.079 | 0.164 | 3067 |
| 1911 | 611 | cyproheptadine | 12 µM | PC3 | 0 | 0.079 | 0.067 | 1938 |
| 1912 | 690 | etifenin | 12 µM | MCF7 | 0 | 0.079 | 0.099 | 4117 |
| 1913 | 627 | trichostatin A | 100 nM | MCF7 | 0 | 0.079 | 0.121 | 1672 |
| 1914 | 626 | tanespimycin | 1 µM | MCF7 | 0 | 0.079 | 0.112 | 1650 |
| 1915 | 687 | rifabutin | 5 µM | MCF7 | 0 | 0.079 | 0.122 | 3873 |
| 1916 | 673 | hymecromone | 23 µM | MCF7 | 0 | 0.079 | 0.13 | 3383 |
| 1917 | 691 | cefsulodin | 7 µM | MCF7 | 0 | 0.079 | 0.059 | 4148 |
| 1918 | 680 | mephenesin | 22 µM | PC3 | 0 | 0.079 | 0.112 | 3675 |
| 1919 | 720 | quinethazone | 14 µM | MCF7 | 0 | 0.079 | 0.091 | 4351 |
| 1920 | 752 | kinetin | 19 µM | MCF7 | 0 | 0.079 | 0.078 | 6073 |
| 1921 | 629 | morantel | 11 µM | HL60 | 0 | 0.079 | 0.188 | 1840 |
| 1922 | 703 | quinethazone | 14 µM | PC3 | 0 | 0.079 | 0.049 | 4529 |
| 1923 | 622 | ketotifen | 9 µM | HL60 | 0 | 0.079 | 0.124 | 1583 |
| 1924 | 657 | trichostatin A | 100 nM | MCF7 | 0 | 0.079 | 0.141 | 2881 |
| 1925 | 726 | pentetic acid | 10 µM | MCF7 | 0 | 0.079 | 0.107 | 5264 |
| 1926 | 747 | albendazole | 15 µM | MCF7 | 0 | 0.079 | 0.048 | 7164 |
| 1927 | 637 | levamisole | 17 µM | MCF7 | 0 | 0.079 | 0.069 | 2257 |
| 1928 | 751 | mepenzolate bromide | 10 µM | MCF7 | 0 | 0.079 | 0.084 | 6024 |
| 1929 | 708 | metyrapone | 18 µM | MCF7 | 0 | 0.079 | 0.122 | 5667 |
| 1930 | 505 | pararosaniline | 10 µM | MCF7 | 0 | 0.079 | 0.107 | 893 |
| 1931 | 686 | mepenzolate bromide | 10 µM | MCF7 | 0 | 0.079 | 0.064 | 3829 |
| 1932 | 662 | tetrahydroalstonine | 11 µM | MCF7 | 0 | 0.079 | 0.096 | 2748 |
| 1933 | 672 | etidronic acid | 16 µM | MCF7 | 0 | 0.079 | 0.09 | 3325 |
| 1934 | 629 | noretynodrel | 13 µM | HL60 | 0 | 0.079 | 0.148 | 1860 |
| 1935 | 752 | Prestwick-689 | 10 µM | MCF7 | 0 | 0.079 | 0.099 | 6076 |
| 1936 | 670 | mephentermine | 9 µM | MCF7 | 0 | 0.079 | 0.086 | 3425 |
| 1937 | 513 | chlorpromazine | 1 µM | MCF7 | 0 | 0.078 | 0.059 | 1055 |
| 1938 | 692 | trichostatin A | 100 nM | PC3 | 0 | 0.078 | 0.125 | 4184 |
| 1939 | 757 | alpha-estradiol | 10 nM | MCF7 | 0 | 0.078 | 0.063 | 5570 |
| 1940 | 650 | valproic acid | 500 µM | HL60 | 0 | 0.078 | 0.113 | 2700 |
| 1941 | 648 | sulfapyridine | 16 µM | HL60 | 0 | 0.078 | 0.08 | 2538 |
| 1942 | 646 | labetalol | 11 µM | MCF7 | 0 | 0.078 | 0.075 | 3167 |
| 1943 | 651 | securinine | 18 µM | HL60 | 0 | 0.078 | 0.127 | 2729 |
| 1944 | 755 | pralidoxime | 23 µM | MCF7 | 0 | 0.078 | 0.078 | 6443 |
| 1945 | 748 | estriol | 14 µM | MCF7 | 0 | 0.078 | 0.099 | 7220 |
| 1946 | 702 | bendroflumethiazide | 9 µM | PC3 | 0 | 0.078 | 0.081 | 4315 |
| 1947 | 618 | tropicamide | 14 µM | HL60 | 0 | 0.078 | 0.156 | 2347 |
| 1948 | 513 | fulvestrant | 1 µM | MCF7 | 0 | 0.078 | 0.087 | 1043 |
| 1949 | 727 | valproic acid | 500 µM | PC3 | 0 | 0.078 | 0.078 | 4464 |
| 1950 | 1077 | 0316684-0000 | 10 µM | PC3 | 0 | 0.078 | 0.059 | 7098 |
| 1951 | 730 | metanephrine | 17 µM | MCF7 | 0 | 0.078 | 0.061 | 5334 |
| 1952 | 673 | equilin | 15 µM | MCF7 | 0 | 0.078 | 0.058 | 3377 |
| 1953 | 695 | nifuroxazide | 15 µM | MCF7 | 0 | 0.078 | 0.112 | 4835 |
| 1954 | 636 | trichostatin A | 100 nM | MCF7 | 0 | 0.078 | 0.118 | 2247 |
| 1955 | 765 | tretinoin | 1 µM | MCF7 | 0 | 0.078 | 0.108 | 6971 |
| 1956 | 636 | gallamine triethiodide | 4 µM | MCF7 | 0 | 0.078 | 0.091 | 2221 |
| 1957 | 680 | PNU-0251126 | 1 µM | PC3 | 0 | 0.078 | 0.061 | 3692 |
| 1958 | 642 | mebendazole | 14 µM | MCF7 | 0 | 0.078 | 0.109 | 2300 |
| 1959 | 649 | sulfamethoxypyridazine | 14 µM | HL60 | 0 | 0.078 | 0.169 | 2550 |
| 1960 | 1008 | trichostatin A | 1 µM | MCF7 | 0 | 0.078 | 0.137 | 5945 |
| 1961 | 653 | chlorprothixene | 11 µM | MCF7 | 0 | 0.078 | 0.076 | 2627 |
| 1962 | 626 | sirolimus | 100 nM | MCF7 | 0 | 0.078 | 0.101 | 1646 |
| 1963 | 686 | promazine | 12 µM | MCF7 | 0 | 0.078 | 0.125 | 3833 |
| 1964 | 641 | trichostatin A | 100 nM | HL60 | 0 | 0.078 | 0.087 | 1785 |
| 1965 | 1015 | trichostatin A | 1 µM | PC3 | 0 | 0.078 | 0.146 | 5981 |
| 1966 | 767 | alpha-estradiol | 10 nM | MCF7 | 0 | 0.078 | 0.063 | 6930 |
| 1967 | 718 | torasemide | 11 µM | PC3 | 0 | 0.078 | 0.054 | 5057 |
| 1968 | 671 | zomepirac | 13 µM | MCF7 | 0 | 0.078 | 0.099 | 3454 |
| 1969 | 729 | nicotinic acid | 32 µM | MCF7 | 0 | 0.078 | 0.055 | 5301 |
| 1970 | 671 | pridinol | 10 µM | MCF7 | 0 | 0.078 | 0.091 | 3456 |
| 1971 | 603 | fulvestrant | 1 µM | PC3 | 0 | 0.078 | 0.072 | 1205 |
| 1972 | 686 | indapamide | 11 µM | MCF7 | 0 | 0.078 | 0.056 | 3859 |
| 1973 | 725 | sirolimus | 100 nM | MCF7 | 0 | 0.077 | 0.107 | 5218 |
| 1974 | 656 | pentamidine | 7 µM | MCF7 | 0 | 0.077 | 0.097 | 2834 |
| 1975 | 713 | trichostatin A | 100 nM | PC3 | 0 | 0.077 | 0.19 | 4665 |
| 1976 | 725 | fulvestrant | 10 nM | MCF7 | 0 | 0.077 | 0.091 | 5235 |
| 1977 | 712 | vanoxerine | 8 µM | PC3 | 0 | 0.077 | 0.056 | 4641 |
| 1978 | 712 | terguride | 9 µM | PC3 | 0 | 0.077 | 0.122 | 4633 |
| 1979 | 662 | acepromazine | 9 µM | MCF7 | 0 | 0.077 | 0.042 | 2769 |
| 1980 | 688 | 6-azathymine | 31 µM | PC3 | 0 | 0.077 | 0.068 | 3987 |
| 1981 | 62 | prochlorperazine | 10 µM | MCF7 | 0 | 0.077 | 0.071 | 455 |
| 1982 | 734 | pridinol | 10 µM | PC3 | 0 | 0.077 | 0.096 | 5860 |
| 1983 | 506 | genistein | 10 µM | MCF7 | 0 | 0.077 | 0.127 | 1015 |
| 1984 | 690 | oxybuprocaine | 12 µM | MCF7 | 0 | 0.077 | 0.078 | 4115 |
| 1985 | 711 | lanatoside C | 4 µM | MCF7 | 0 | 0.077 | 0.074 | 3963 |
| 1986 | 603 | prochlorperazine | 10 µM | PC3 | 0 | 0.077 | 0.08 | 1215 |
| 1987 | 702 | ethoxyquin | 18 µM | PC3 | 0 | 0.077 | 0.074 | 4321 |
| 1988 | 659 | latamoxef | 7 µM | HL60 | 0 | 0.077 | 0.109 | 3028 |
| 1989 | 614 | ivermectin | 5 µM | HL60 | 0 | 0.077 | 0.112 | 1367 |
| 1990 | 603 | tanespimycin | 1 µM | PC3 | 0 | 0.077 | 0.062 | 1225 |
| 1991 | 632 | minoxidil | 19 µM | MCF7 | 0 | 0.077 | 0.064 | 1496 |
| 1992 | 743 | propoxycaine | 12 µM | MCF7 | 0 | 0.077 | 0.073 | 6803 |
| 1993 | 616 | triamcinolone | 10 µM | PC3 | 0 | 0.077 | 0.058 | 2078 |
| 1994 | 720 | trichostatin A | 100 nM | MCF7 | 0 | 0.077 | 0.083 | 4348 |
| 1995 | 622 | clonidine | 15 µM | HL60 | 0 | 0.077 | 0.133 | 1555 |
| 1996 | 502 | tretinoin | 1 µM | MCF7 | 0 | 0.077 | 0.087 | 966 |
| 1997 | 1075 | GW-8510 | 10 µM | PC3 | 0 | 0.077 | 0.123 | 7085 |
| 1998 | 13 | 15-delta prostaglandin J2 | 10 µM | MCF7 | 0 | 0.077 | 0.068 | 201 |
| 1999 | 764 | dipivefrine | 10 µM | PC3 | 0 | 0.077 | 0.058 | 7124 |
| 2000 | 627 | procaine | 15 µM | MCF7 | 0 | 0.077 | 0.089 | 1674 |
| 2001 | 513 | valproic acid | 50 µM | MCF7 | 0 | 0.077 | 0.08 | 1060 |
| 2002 | 25 | flufenamic acid | 10 µM | MCF7 | 0 | 0.077 | 0.056 | 316 |
| 2003 | 626 | vorinostat | 10 µM | MCF7 | 0 | 0.077 | 0.096 | 1645 |
| 2004 | 749 | meropenem | 10 µM | HL60 | 0 | 0.077 | 0.144 | 6141 |
| 2005 | 654 | carbarsone | 15 µM | MCF7 | 0 | 0.077 | 0.063 | 3250 |
| 2006 | 637 | pargyline | 20 µM | MCF7 | 0 | 0.077 | 0.053 | 2265 |
| 2007 | 653 | droperidol | 11 µM | MCF7 | 0 | 0.077 | 0.085 | 2645 |
| 2008 | 677 | imidurea | 10 µM | MCF7 | 0 | 0.077 | 0.081 | 3522 |
| 2009 | 614 | dexamethasone | 9 µM | HL60 | 0 | 0.077 | 0.08 | 1396 |
| 2010 | 53 | thioridazine | 1 µM | MCF7 | 0 | 0.077 | 0.055 | 422 |
| 2011 | 694 | spectinomycin | 10 µM | MCF7 | 0 | 0.077 | 0.064 | 4773 |
| 2012 | 1005 | fulvestrant | 1 µM | PC3 | 0 | 0.076 | 0.07 | 5931 |
| 2013 | 645 | citiolone | 25 µM | HL60 | 0 | 0.076 | 0.158 | 2176 |
| 2014 | 646 | albendazole | 15 µM | MCF7 | 0 | 0.076 | 0.101 | 3164 |
| 2015 | 746 | tropine | 28 µM | MCF7 | 0 | 0.076 | 0.046 | 6264 |
| 2016 | 699 | atractyloside | 5 µM | MCF7 | 0 | 0.076 | 0.11 | 4717 |
| 2017 | 732 | lidoflazine | 8 µM | PC3 | 0 | 0.076 | 0.049 | 5804 |
| 2018 | 636 | nocodazole | 13 µM | MCF7 | 0 | 0.076 | 0.06 | 2239 |
| 2019 | 726 | clioquinol | 13 µM | MCF7 | 0 | 0.076 | 0.076 | 5258 |
| 2020 | 647 | practolol | 15 µM | MCF7 | 0 | 0.076 | 0.102 | 3204 |
| 2021 | 1007 | H-7 | 100 µM | PC3 | 0 | 0.076 | 0.175 | 5941 |
| 2022 | 705 | alcuronium chloride | 5 µM | MCF7 | 0 | 0.076 | 0.052 | 4409 |
| 2023 | 745 | dihydrostreptomycin | 3 µM | MCF7 | 0 | 0.076 | 0.062 | 6228 |
| 2024 | 670 | alpha-ergocryptine | 7 µM | MCF7 | 0 | 0.076 | 0.104 | 3434 |
| 2025 | 695 | cetirizine | 9 µM | MCF7 | 0 | 0.076 | 0.079 | 4815 |
| 2026 | 725 | prochlorperazine | 10 µM | MCF7 | 0 | 0.076 | 0.071 | 5212 |
| 2027 | 646 | vinpocetine | 11 µM | MCF7 | 0 | 0.076 | 0.072 | 3174 |
| 2028 | 736 | benzonatate | 7 µM | MCF7 | 0 | 0.076 | 0.097 | 5435 |
| 2029 | 737 | etofylline | 18 µM | MCF7 | 0 | 0.076 | 0.079 | 5467 |
| 2030 | 109 | valproic acid | 1 mM | SKMEL5 | 0 | 0.076 | 0.101 | 629 |
| 2031 | 664 | Prestwick-967 | 26 µM | HL60 | 0 | 0.076 | 0.169 | 2925 |
| 2032 | 603 | estradiol | 10 nM | PC3 | 0 | 0.076 | 0.062 | 1241 |
| 2033 | 661 | adrenosterone | 13 µM | HL60 | 0 | 0.076 | 0.126 | 3107 |
| 2034 | 752 | hexestrol | 15 µM | MCF7 | 0 | 0.076 | 0.099 | 6077 |
| 2035 | 707 | sulconazole | 9 µM | MCF7 | 0 | 0.076 | 0.075 | 4998 |
| 2036 | 737 | ceftazidime | 6 µM | MCF7 | 0 | 0.076 | 0.09 | 5473 |
| 2037 | 650 | prochlorperazine | 10 µM | HL60 | 0 | 0.076 | 0.1 | 2675 |
| 2038 | 665 | tridihexethyl | 11 µM | HL60 | 0 | 0.076 | 0.106 | 2964 |
| 2039 | 718 | ceftazidime | 6 µM | PC3 | 0 | 0.076 | 0.048 | 5054 |
| 2040 | 701 | fenbufen | 16 µM | PC3 | 0 | 0.076 | 0.137 | 4279 |
| 2041 | 504 | bucladesine | 2 µM | MCF7 | 0 | 0.076 | 0.085 | 842 |
| 2042 | 732 | hexestrol | 15 µM | PC3 | 0 | 0.076 | 0.058 | 5776 |
| 2043 | 616 | dizocilpine | 12 µM | PC3 | 0 | 0.076 | 0.062 | 2069 |
| 2044 | 618 | vincamine | 11 µM | HL60 | 0 | 0.076 | 0.077 | 2367 |
| 2045 | 687 | CP-690334-01 | 1 µM | MCF7 | 0 | 0.076 | 0.069 | 3909 |
| 2046 | 654 | ginkgolide A | 10 µM | MCF7 | 0 | 0.076 | 0.098 | 3260 |
| 2047 | 658 | scopolamine | 12 µM | HL60 | 0 | 0.075 | 0.113 | 3018 |
| 2048 | 505 | calmidazolium | 5 µM | MCF7 | 0 | 0.075 | 0.052 | 906 |
| 2049 | 676 | moxonidine | 17 µM | MCF7 | 0 | 0.075 | 0.083 | 7343 |
| 2050 | 729 | promethazine | 12 µM | MCF7 | 0 | 0.075 | 0.109 | 5317 |
| 2051 | 736 | alfaxalone | 12 µM | MCF7 | 0 | 0.075 | 0.082 | 5451 |
| 2052 | 672 | procyclidine | 12 µM | MCF7 | 0 | 0.075 | 0.068 | 3330 |
| 2053 | 661 | tiletamine | 15 µM | HL60 | 0 | 0.075 | 0.073 | 3137 |
| 2054 | 659 | carbachol | 22 µM | HL60 | 0 | 0.075 | 0.142 | 3042 |
| 2055 | 659 | dimethadione | 31 µM | HL60 | 0 | 0.075 | 0.118 | 3029 |
| 2056 | 711 | pempidine | 13 µM | MCF7 | 0 | 0.075 | 0.073 | 3926 |
| 2057 | 725 | haloperidol | 10 µM | MCF7 | 0 | 0.075 | 0.068 | 5200 |
| 2058 | 753 | 2-aminobenzenesulfonamide | 23 µM | PC3 | 0 | 0.075 | 0.07 | 6321 |
| 2059 | 670 | trichostatin A | 100 nM | MCF7 | 0 | 0.075 | 0.065 | 3428 |
| 2060 | 637 | alverine | 8 µM | MCF7 | 0 | 0.075 | 0.103 | 2273 |
| 2061 | 747 | Prestwick-689 | 10 µM | MCF7 | 0 | 0.075 | 0.048 | 7173 |
| 2062 | 647 | amikacin | 7 µM | MCF7 | 0 | 0.075 | 0.067 | 3233 |
| 2063 | 659 | propofol | 22 µM | HL60 | 0 | 0.075 | 0.106 | 3048 |
| 2064 | 631 | trichostatin A | 100 nM | HL60 | 0 | 0.075 | 0.149 | 1306 |
| 2065 | 671 | isopropamide iodide | 8 µM | MCF7 | 0 | 0.075 | 0.095 | 3461 |
| 2066 | 712 | capsaicin | 13 µM | PC3 | 0 | 0.075 | 0.056 | 4612 |
| 2067 | 676 | gabexate | 10 µM | MCF7 | 0 | 0.075 | 0.064 | 7357 |
| 2068 | 109 | benserazide | 10 µM | SKMEL5 | 0 | 0.075 | 0.167 | 631 |
| 2069 | 504 | felodipine | 10 µM | MCF7 | 0 | 0.075 | 0.079 | 848 |
| 2070 | 642 | pentolonium | 7 µM | MCF7 | 0 | 0.075 | 0.111 | 2305 |
| 2071 | 764 | 1,4-chrysenequinone | 15 µM | PC3 | 0 | 0.075 | 0.083 | 7139 |
| 2072 | 650 | valproic acid | 1 mM | HL60 | 0 | 0.075 | 0.095 | 2669 |
| 2073 | 513 | tanespimycin | 1 µM | MCF7 | 0 | 0.075 | 0.07 | 1044 |
| 2074 | 734 | azaperone | 12 µM | PC3 | 0 | 0.075 | 0.08 | 5877 |
| 2075 | 615 | meclofenamic acid | 12 µM | HL60 | 0 | 0.075 | 0.09 | 1445 |
| 2076 | 712 | droperidol | 11 µM | PC3 | 0 | 0.075 | 0.099 | 4629 |
| 2077 | 705 | thiostrepton | 2 µM | MCF7 | 0 | 0.075 | 0.06 | 4385 |
| 2078 | 502 | bucladesine | 2 µM | MCF7 | 0 | 0.075 | 0.068 | 959 |
| 2079 | 612 | doxylamine | 10 µM | HL60 | 0 | 0.075 | 0.118 | 1973 |
| 2080 | 736 | imipramine | 13 µM | MCF7 | 0 | 0.075 | 0.078 | 5440 |
| 2081 | 718 | chlorpromazine | 11 µM | PC3 | 0 | 0.075 | 0.081 | 5074 |
| 2082 | 632 | idoxuridine | 11 µM | MCF7 | 0 | 0.075 | 0.065 | 1480 |
| 2083 | 726 | cotinine | 23 µM | MCF7 | 0 | 0.075 | 0.064 | 5246 |
| 2084 | 670 | demeclocycline | 8 µM | MCF7 | 0 | 0.075 | 0.109 | 3404 |
| 2085 | 617 | iproniazid | 14 µM | PC3 | 0 | 0.075 | 0.065 | 2125 |
| 2086 | 731 | cyclopentolate | 12 µM | PC3 | 0 | 0.075 | 0.061 | 5734 |
| 2087 | 745 | syrosingopine | 6 µM | MCF7 | 0 | 0.074 | 0.055 | 6213 |
| 2088 | 622 | sulfinpyrazone | 10 µM | HL60 | 0 | 0.074 | 0.118 | 1574 |
| 2089 | 692 | tolnaftate | 13 µM | PC3 | 0 | 0.074 | 0.104 | 4221 |
| 2090 | 751 | metamizole sodium | 12 µM | MCF7 | 0 | 0.074 | 0.097 | 6030 |
| 2091 | 678 | piperacetazine | 10 µM | MCF7 | 0 | 0.074 | 0.062 | 3574 |
| 2092 | 653 | dequalinium chloride | 8 µM | MCF7 | 0 | 0.074 | 0.061 | 2631 |
| 2093 | 757 | vorinostat | 10 µM | MCF7 | 0 | 0.074 | 0.14 | 5580 |
| 2094 | 636 | nicergoline | 8 µM | MCF7 | 0 | 0.074 | 0.055 | 2220 |
| 2095 | 646 | clofilium tosylate | 8 µM | MCF7 | 0 | 0.074 | 0.067 | 3187 |
| 2096 | 1024 | valdecoxib | 10 µM | MCF7 | 0 | 0.074 | 0.065 | 6403 |
| 2097 | 676 | nifurtimox | 14 µM | MCF7 | 0 | 0.074 | 0.068 | 7328 |
| 2098 | 691 | selegiline | 18 µM | MCF7 | 0 | 0.074 | 0.091 | 4146 |
| 2099 | 741 | chenodeoxycholic acid | 10 µM | MCF7 | 0 | 0.074 | 0.075 | 6012 |
| 2100 | 761 | glafenine | 10 µM | PC3 | 0 | 0.074 | 0.073 | 7257 |
| 2101 | 762 | dosulepin | 12 µM | PC3 | 0 | 0.074 | 0.058 | 7284 |
| 2102 | 612 | sulfathiazole | 16 µM | HL60 | 0 | 0.074 | 0.107 | 1963 |
| 2103 | 671 | drofenine | 11 µM | MCF7 | 0 | 0.074 | 0.091 | 3455 |
| 2104 | 725 | thioridazine | 10 µM | MCF7 | 0 | 0.074 | 0.053 | 5227 |
| 2105 | 685 | 6-benzylaminopurine | 18 µM | MCF7 | 0 | 0.074 | 0.127 | 3623 |
| 2106 | 719 | dropropizine | 17 µM | PC3 | 0 | 0.074 | 0.076 | 5106 |
| 2107 | 671 | vidarabine | 15 µM | MCF7 | 0 | 0.074 | 0.12 | 3445 |
| 2108 | 642 | trichostatin A | 100 nM | MCF7 | 0 | 0.074 | 0.067 | 2330 |
| 2109 | 670 | levonorgestrel | 13 µM | MCF7 | 0 | 0.074 | 0.126 | 3406 |
| 2110 | 757 | chlorpromazine | 1 µM | MCF7 | 0 | 0.074 | 0.12 | 5577 |
| 2111 | 748 | harmine | 16 µM | MCF7 | 0 | 0.074 | 0.104 | 7209 |
| 2112 | 680 | 0175029-0000 | 10 µM | PC3 | 0 | 0.074 | 0.181 | 3691 |
| 2113 | 744 | riboflavin | 11 µM | MCF7 | 0 | 0.074 | 0.06 | 6822 |
| 2114 | 732 | tropine | 28 µM | PC3 | 0 | 0.074 | 0.049 | 5790 |
| 2115 | 761 | enalapril | 8 µM | PC3 | 0 | 0.074 | 0.07 | 7265 |
| 2116 | 648 | hexestrol | 15 µM | HL60 | 0 | 0.074 | 0.091 | 2515 |
| 2117 | 637 | mefexamide | 13 µM | MCF7 | 0 | 0.074 | 0.101 | 2284 |
| 2118 | 741 | ozagrel | 15 µM | MCF7 | 0 | 0.073 | 0.087 | 5983 |
| 2119 | 1051 | H-89 | 500 nM | MCF7 | 0 | 0.073 | 0.147 | 6878 |
| 2120 | 725 | vorinostat | 10 µM | MCF7 | 0 | 0.073 | 0.079 | 5217 |
| 2121 | 748 | cefmetazole | 8 µM | MCF7 | 0 | 0.073 | 0.086 | 7222 |
| 2122 | 610 | oxetacaine | 9 µM | PC3 | 0 | 0.073 | 0.074 | 1903 |
| 2123 | 82 | novobiocin | 100 µM | MCF7 | 0 | 0.073 | 0.083 | 576 |
| 2124 | 1016 | 3-nitropropionic acid | 10 µM | MCF7 | 0 | 0.073 | 0.086 | 6367 |
| 2125 | 622 | labetalol | 11 µM | HL60 | 0 | 0.073 | 0.205 | 1550 |
| 2126 | 756 | azapropazone | 13 µM | MCF7 | 0 | 0.073 | 0.055 | 6522 |
| 2127 | 635 | salsolidin | 16 µM | HL60 | 0 | 0.073 | 0.131 | 2463 |
| 2128 | 677 | lymecycline | 7 µM | MCF7 | 0 | 0.073 | 0.094 | 3514 |
| 2129 | 700 | medrysone | 12 µM | MCF7 | 0 | 0.073 | 0.1 | 4727 |
| 2130 | 649 | cloperastine | 11 µM | HL60 | 0 | 0.073 | 0.134 | 2549 |
| 2131 | 751 | halcinonide | 9 µM | MCF7 | 0 | 0.073 | 0.133 | 6040 |
| 2132 | 1048 | PF-00562151-00 | 10 µM | PC3 | 0 | 0.073 | 0.06 | 6863 |
| 2133 | 731 | gallamine triethiodide | 4 µM | PC3 | 0 | 0.073 | 0.066 | 5735 |
| 2134 | 659 | paracetamol | 26 µM | HL60 | 0 | 0.073 | 0.098 | 3025 |
| 2135 | 618 | fenbufen | 16 µM | HL60 | 0 | 0.073 | 0.108 | 2346 |
| 2136 | 658 | trichostatin A | 100 nM | HL60 | 0 | 0.073 | 0.117 | 2993 |
| 2137 | 676 | alcuronium chloride | 5 µM | MCF7 | 0 | 0.073 | 0.078 | 7345 |
| 2138 | 646 | guanethidine | 13 µM | MCF7 | 0 | 0.073 | 0.136 | 3171 |
| 2139 | 672 | tetroquinone | 21 µM | MCF7 | 0 | 0.073 | 0.075 | 3338 |
| 2140 | 636 | pirenzepine | 9 µM | MCF7 | 0 | 0.073 | 0.094 | 2234 |
| 2141 | 1090 | irinotecan | 100 µM | MCF7 | 0 | 0.073 | 0.163 | 7530 |
| 2142 | 628 | trichostatin A | 100 nM | PC3 | 0 | 0.073 | 0.108 | 1793 |
| 2143 | 751 | myosmine | 27 µM | MCF7 | 0 | 0.073 | 0.075 | 6055 |
| 2144 | 619 | trazodone | 10 µM | HL60 | 0 | 0.073 | 0.166 | 2379 |
| 2145 | 645 | Prestwick-674 | 14 µM | HL60 | 0 | 0.073 | 0.156 | 2179 |
| 2146 | 647 | methyldopa | 19 µM | MCF7 | 0 | 0.073 | 0.117 | 3234 |
| 2147 | 677 | levocabastine | 9 µM | MCF7 | 0 | 0.073 | 0.082 | 3509 |
| 2148 | 610 | trimethobenzamide | 9 µM | PC3 | 0 | 0.073 | 0.064 | 1920 |
| 2149 | 614 | naltrexone | 10 µM | HL60 | 0 | 0.073 | 0.117 | 1363 |
| 2150 | 711 | eldeline | 8 µM | MCF7 | 0 | 0.073 | 0.101 | 3925 |
| 2151 | 744 | epivincamine | 11 µM | MCF7 | 0 | 0.073 | 0.048 | 6838 |
| 2152 | 706 | palmatine | 10 µM | MCF7 | 0 | 0.073 | 0.082 | 4957 |
| 2153 | 1020 | 0225151-0000 | 10 µM | MCF7 | 0 | 0.073 | 0.099 | 6384 |
| 2154 | 726 | flurbiprofen | 16 µM | MCF7 | 0 | 0.073 | 0.081 | 5269 |
| 2155 | 506 | chlorpromazine | 1 µM | MCF7 | 0 | 0.073 | 0.102 | 997 |
| 2156 | 632 | prednisone | 11 µM | MCF7 | 0 | 0.073 | 0.09 | 1478 |
| 2157 | 1073 | alsterpaullone | 10 µM | PC3 | 0 | 0.073 | 0.137 | 7078 |
| 2158 | 506 | acetylsalicylic acid | 100 µM | MCF7 | 0 | 0.073 | 0.087 | 984 |
| 2159 | 677 | quinpirole | 16 µM | MCF7 | 0 | 0.073 | 0.076 | 3539 |
| 2160 | 663 | retrorsine | 11 µM | MCF7 | 0 | 0.073 | 0.093 | 2784 |
| 2161 | 750 | haloperidol | 10 µM | HL60 | 0 | 0.073 | 0.193 | 6163 |
| 2162 | 33 | valproic acid | 10 mM | MCF7 | 0 | 0.073 | 0.066 | 345 |
| 2163 | 662 | lobeline | 11 µM | MCF7 | 0 | 0.073 | 0.057 | 2763 |
| 2164 | 602 | alvespimycin | 100 nM | HL60 | 0 | 0.073 | 0.109 | 1154 |
| 2165 | 640 | trichostatin A | 100 nM | HL60 | 0 | 0.073 | 0.12 | 1732 |
| 2166 | 635 | omeprazole | 12 µM | HL60 | 0 | 0.073 | 0.189 | 2467 |
| 2167 | 682 | indapamide | 11 µM | PC3 | 0 | 0.072 | 0.108 | 3778 |
| 2168 | 673 | pentetic acid | 10 µM | MCF7 | 0 | 0.072 | 0.062 | 3387 |
| 2169 | 506 | valproic acid | 200 µM | MCF7 | 0 | 0.072 | 0.07 | 994 |
| 2170 | 506 | tanespimycin | 1 µM | MCF7 | 0 | 0.072 | 0.052 | 986 |
| 2171 | 709 | arcaine | 15 µM | PC3 | 0 | 0.072 | 0.088 | 6629 |
| 2172 | 706 | trichostatin A | 100 nM | MCF7 | 0 | 0.072 | 0.122 | 4954 |
| 2173 | 707 | (-)-MK-801 | 12 µM | MCF7 | 0 | 0.072 | 0.075 | 5003 |
| 2174 | 630 | dextromethorphan | 11 µM | HL60 | 0 | 0.072 | 0.17 | 1281 |
| 2175 | 42 | sirolimus | 100 nM | ssMCF7 | 0 | 0.072 | 0.071 | 402 |
| 2176 | 687 | flutamide | 14 µM | MCF7 | 0 | 0.072 | 0.055 | 3885 |
| 2177 | 677 | cyclic adenosine monophosphate | 12 µM | MCF7 | 0 | 0.072 | 0.102 | 3531 |
| 2178 | 665 | trichostatin A | 100 nM | HL60 | 0 | 0.072 | 0.173 | 2949 |
| 2179 | 705 | apigenin | 15 µM | MCF7 | 0 | 0.072 | 0.119 | 4401 |
| 2180 | 771 | cortisone | 11 µM | MCF7 | 0 | 0.072 | 0.091 | 7416 |
| 2181 | 712 | betaxolol | 12 µM | PC3 | 0 | 0.072 | 0.126 | 4608 |
| 2182 | 1059 | BAS-012416453 | 38 µM | MCF7 | 0 | 0.072 | 0.055 | 6908 |
| 2183 | 751 | Prestwick-674 | 14 µM | MCF7 | 0 | 0.072 | 0.127 | 6034 |
| 2184 | 626 | monorden | 100 nM | MCF7 | 0 | 0.072 | 0.077 | 1644 |
| 2185 | 692 | scopolamine | 12 µM | PC3 | 0 | 0.072 | 0.078 | 4219 |
| 2186 | 758 | phthalylsulfathiazole | 10 µM | MCF7 | 0 | 0.072 | 0.089 | 5614 |
| 2187 | 729 | fluvastatin | 9 µM | MCF7 | 0 | 0.072 | 0.061 | 5290 |
| 2188 | 629 | sulfaphenazole | 13 µM | HL60 | 0 | 0.072 | 0.194 | 1836 |
| 2189 | 644 | seneciphylline | 12 µM | HL60 | 0 | 0.072 | 0.112 | 2140 |
| 2190 | 110b | genistein | 10 µM | PC3 | 0 | 0.072 | 0.055 | 703 |
| 2191 | 653 | bepridil | 10 µM | MCF7 | 0 | 0.072 | 0.064 | 2629 |
| 2192 | 680 | tiapride | 11 µM | PC3 | 0 | 0.072 | 0.054 | 3663 |
| 2193 | 728 | clonidine | 15 µM | PC3 | 0 | 0.072 | 0.036 | 4478 |
| 2194 | 659 | ganciclovir | 16 µM | HL60 | 0 | 0.072 | 0.069 | 3030 |
| 2195 | 634 | trichostatin A | 100 nM | HL60 | 0 | 0.072 | 0.099 | 2450 |
| 2196 | 682 | eucatropine | 12 µM | PC3 | 0 | 0.072 | 0.085 | 3759 |
| 2197 | 1079 | colforsin | 500 nM | PC3 | 0 | 0.072 | 0.068 | 7104 |
| 2198 | 28 | trichostatin A | 100 nM | MCF7 | 0 | 0.072 | 0.081 | 332 |
| 2199 | 685 | flumetasone | 10 µM | MCF7 | 0 | 0.072 | 0.106 | 3610 |
| 2200 | 712 | colchicine | 10 µM | PC3 | 0 | 0.072 | 0.061 | 4614 |
| 2201 | 1045 | AG-028671 | 10 µM | MCF7 | 0 | 0.072 | 0.079 | 6587 |
| 2202 | 671 | iopamidol | 5 µM | MCF7 | 0 | 0.072 | 0.074 | 3473 |
| 2203 | 749 | trichostatin A | 100 nM | HL60 | 0 | 0.072 | 0.108 | 6143 |
| 2204 | 657 | mesoridazine | 7 µM | MCF7 | 0 | 0.072 | 0.073 | 2874 |
| 2205 | 664 | naftopidil | 9 µM | HL60 | 0 | 0.072 | 0.21 | 2911 |
| 2206 | 693 | zuclopenthixol | 9 µM | PC3 | 0 | 0.072 | 0.126 | 4261 |
| 2207 | 757 | sirolimus | 100 nM | MCF7 | 0 | 0.072 | 0.132 | 5567 |
| 2208 | 513 | tretinoin | 1 µM | MCF7 | 0 | 0.071 | 0.09 | 1049 |
| 2209 | 642 | tolbutamide | 15 µM | MCF7 | 0 | 0.071 | 0.06 | 2320 |
| 2210 | 110b | alpha-estradiol | 10 nM | PC3 | 0 | 0.071 | 0.057 | 702 |
| 2211 | 660 | trichostatin A | 100 nM | HL60 | 0 | 0.071 | 0.154 | 3077 |
| 2212 | 627 | sulfacetamide | 16 µM | MCF7 | 0 | 0.071 | 0.063 | 1695 |
| 2213 | 720 | flutamide | 14 µM | MCF7 | 0 | 0.071 | 0.059 | 4361 |
| 2214 | 715 | zidovudine | 15 µM | PC3 | 0 | 0.071 | 0.063 | 6733 |
| 2215 | 730 | hemicholinium | 7 µM | MCF7 | 0 | 0.071 | 0.06 | 5339 |
| 2216 | 730 | ioversol | 5 µM | MCF7 | 0 | 0.071 | 0.05 | 5326 |
| 2217 | 745 | etanidazole | 19 µM | MCF7 | 0 | 0.071 | 0.065 | 6211 |
| 2218 | 756 | nabumetone | 18 µM | MCF7 | 0 | 0.071 | 0.064 | 6487 |
| 2219 | 651 | ribostamycin | 7 µM | HL60 | 0 | 0.071 | 0.143 | 2705 |
| 2220 | 765 | vorinostat | 10 µM | MCF7 | 0 | 0.071 | 0.097 | 6980 |
| 2221 | 623 | brinzolamide | 10 µM | HL60 | 0 | 0.071 | 0.117 | 1615 |
| 2222 | 727 | valproic acid | 1 mM | PC3 | 0 | 0.071 | 0.072 | 4433 |
| 2223 | 737 | dapsone | 16 µM | MCF7 | 0 | 0.071 | 0.066 | 5498 |
| 2224 | 693 | cefalonium | 9 µM | PC3 | 0 | 0.071 | 0.067 | 4245 |
| 2225 | 654 | apigenin | 15 µM | MCF7 | 0 | 0.071 | 0.084 | 3257 |
| 2226 | 110b | fulvestrant | 1 µM | PC3 | 0 | 0.071 | 0.061 | 704 |
| 2227 | 53 | trifluoperazine | 10 µM | MCF7 | 0 | 0.071 | 0.046 | 421 |
| 2228 | 623 | clofazimine | 8 µM | HL60 | 0 | 0.071 | 0.163 | 1624 |
| 2229 | 765 | haloperidol | 10 µM | MCF7 | 0 | 0.071 | 0.057 | 6963 |
| 2230 | 506 | trichostatin A | 1 µM | MCF7 | 0 | 0.071 | 0.123 | 1014 |
| 2231 | 750 | fulvestrant | 10 nM | HL60 | 0 | 0.071 | 0.118 | 6197 |
| 2232 | 771 | flavoxate | 9 µM | MCF7 | 0 | 0.071 | 0.072 | 7405 |
| 2233 | 704 | captopril | 17 µM | PC3 | 0 | 0.071 | 0.084 | 4585 |
| 2234 | 737 | chloramphenicol | 12 µM | MCF7 | 0 | 0.071 | 0.131 | 5466 |
| 2235 | 651 | zomepirac | 13 µM | HL60 | 0 | 0.071 | 0.168 | 2713 |
| 2236 | 733 | trifluridine | 14 µM | PC3 | 0 | 0.071 | 0.117 | 5819 |
| 2237 | 664 | fluvoxamine | 9 µM | HL60 | 0 | 0.071 | 0.248 | 2913 |
| 2238 | 618 | phenformin | 17 µM | HL60 | 0 | 0.071 | 0.102 | 2350 |
| 2239 | 653 | trichostatin A | 100 nM | MCF7 | 0 | 0.071 | 0.122 | 2639 |
| 2240 | 688 | mycophenolic acid | 12 µM | PC3 | 0 | 0.071 | 0.092 | 4019 |
| 2241 | 714 | flucytosine | 31 µM | PC3 | 0 | 0.071 | 0.077 | 6690 |
| 2242 | 660 | aminohippuric acid | 21 µM | HL60 | 0 | 0.071 | 0.168 | 3076 |
| 2243 | 703 | lithocholic acid | 11 µM | PC3 | 0 | 0.071 | 0.063 | 4551 |
| 2244 | 701 | Prestwick-674 | 14 µM | PC3 | 0 | 0.071 | 0.056 | 4276 |
| 2245 | 660 | bephenium hydroxynaphthoate | 9 µM | HL60 | 0 | 0.071 | 0.095 | 3089 |
| 2246 | 633 | hesperetin | 13 µM | MCF7 | 0 | 0.07 | 0.105 | 1531 |
| 2247 | 712 | bepridil | 10 µM | PC3 | 0 | 0.07 | 0.089 | 4613 |
| 2248 | 653 | prochlorperazine | 7 µM | MCF7 | 0 | 0.07 | 0.115 | 2641 |
| 2249 | 752 | trichostatin A | 100 nM | MCF7 | 0 | 0.07 | 0.121 | 6085 |
| 2250 | 746 | tretinoin | 13 µM | MCF7 | 0 | 0.07 | 0.091 | 6243 |
| 2251 | 632 | captopril | 17 µM | MCF7 | 0 | 0.07 | 0.094 | 1488 |
| 2252 | 68 | fluphenazine | 10 µM | MCF7 | 0 | 0.07 | 0.1 | 490 |
| 2253 | 748 | vinpocetine | 11 µM | MCF7 | 0 | 0.07 | 0.054 | 7213 |
| 2254 | 755 | pipemidic acid | 13 µM | MCF7 | 0 | 0.07 | 0.074 | 6470 |
| 2255 | 1082 | irinotecan | 100 µM | MCF7 | 0 | 0.07 | 0.129 | 7498 |
| 2256 | 502 | blebbistatin | 17 µM | MCF7 | 0 | 0.07 | 0.079 | 954 |
| 2257 | 693 | primaquine | 9 µM | PC3 | 0 | 0.07 | 0.125 | 4263 |
| 2258 | 673 | primidone | 18 µM | MCF7 | 0 | 0.07 | 0.054 | 3402 |
| 2259 | 611 | danazol | 12 µM | PC3 | 0 | 0.07 | 0.117 | 1954 |
| 2260 | 657 | lobelanidine | 11 µM | MCF7 | 0 | 0.07 | 0.096 | 2897 |
| 2261 | 738 | prednisolone | 11 µM | MCF7 | 0 | 0.07 | 0.086 | 5526 |
| 2262 | 648 | tetryzoline | 17 µM | HL60 | 0 | 0.07 | 0.107 | 2507 |
| 2263 | 708 | droperidol | 11 µM | MCF7 | 0 | 0.07 | 0.08 | 5690 |
| 2264 | 664 | trichostatin A | 100 nM | HL60 | 0 | 0.07 | 0.177 | 2904 |
| 2265 | 665 | talampicillin | 8 µM | HL60 | 0 | 0.07 | 0.213 | 2954 |
| 2266 | 619 | flunarizine | 8 µM | HL60 | 0 | 0.07 | 0.111 | 2381 |
| 2267 | 673 | eticlopride | 11 µM | MCF7 | 0 | 0.07 | 0.056 | 3393 |
| 2268 | 672 | carbenoxolone | 7 µM | MCF7 | 0 | 0.07 | 0.047 | 3353 |
| 2269 | 677 | ozagrel | 15 µM | MCF7 | 0 | 0.07 | 0.083 | 3503 |
| 2270 | 618 | nitrofurantoin | 17 µM | HL60 | 0 | 0.07 | 0.124 | 2341 |
| 2271 | 685 | metoclopramide | 12 µM | MCF7 | 0 | 0.07 | 0.057 | 3625 |
| 2272 | 644 | solanine | 5 µM | HL60 | 0 | 0.07 | 0.192 | 2152 |
| 2273 | 660 | terguride | 9 µM | HL60 | 0 | 0.07 | 0.156 | 3096 |
| 2274 | 730 | rolitetracycline | 8 µM | MCF7 | 0 | 0.07 | 0.077 | 5331 |
| 2275 | 672 | scopolamine | 12 µM | MCF7 | 0 | 0.07 | 0.117 | 3357 |
| 2276 | 616 | mianserin | 13 µM | PC3 | 0 | 0.07 | 0.081 | 2068 |
| 2277 | 699 | nalidixic acid | 15 µM | MCF7 | 0 | 0.07 | 0.082 | 4691 |
| 2278 | 734 | leflunomide | 15 µM | PC3 | 0 | 0.07 | 0.051 | 5884 |
| 2279 | 665 | lymecycline | 7 µM | HL60 | 0 | 0.07 | 0.176 | 2953 |
| 2280 | 660 | lorglumide | 8 µM | HL60 | 0 | 0.07 | 0.142 | 3079 |
| 2281 | 630 | trichostatin A | 100 nM | HL60 | 0 | 0.07 | 0.134 | 1284 |
| 2282 | 626 | trifluoperazine | 10 µM | MCF7 | 0 | 0.07 | 0.078 | 1649 |
| 2283 | 752 | trioxysalen | 18 µM | MCF7 | 0 | 0.07 | 0.101 | 6078 |
| 2284 | 751 | bromopride | 12 µM | MCF7 | 0 | 0.07 | 0.093 | 6037 |
| 2285 | 693 | cyclopenthiazide | 11 µM | PC3 | 0 | 0.069 | 0.112 | 4229 |
| 2286 | 506 | tanespimycin | 1 µM | MCF7 | 0 | 0.069 | 0.06 | 1006 |
| 2287 | 680 | glycopyrronium bromide | 10 µM | PC3 | 0 | 0.069 | 0.064 | 3687 |
| 2288 | 715 | rolipram | 15 µM | PC3 | 0 | 0.069 | 0.073 | 6730 |
| 2289 | 719 | bufexamac | 18 µM | PC3 | 0 | 0.069 | 0.118 | 5090 |
| 2290 | 637 | pentoxifylline | 14 µM | MCF7 | 0 | 0.069 | 0.078 | 2290 |
| 2291 | 692 | quinisocaine | 13 µM | PC3 | 0 | 0.069 | 0.115 | 4207 |
| 2292 | 662 | ipratropium bromide | 10 µM | MCF7 | 0 | 0.069 | 0.086 | 2762 |
| 2293 | 1036 | Gly-His-Lys | 1 µM | PC3 | 0 | 0.069 | 0.085 | 6560 |
| 2294 | 692 | meptazinol | 15 µM | PC3 | 0 | 0.069 | 0.105 | 4188 |
| 2295 | 707 | suloctidil | 12 µM | MCF7 | 0 | 0.069 | 0.065 | 5021 |
| 2296 | 707 | prochlorperazine | 7 µM | MCF7 | 0 | 0.069 | 0.066 | 5010 |
| 2297 | 506 | sirolimus | 100 nM | MCF7 | 0 | 0.069 | 0.086 | 1022 |
| 2298 | 614 | nicergoline | 8 µM | HL60 | 0 | 0.069 | 0.102 | 1374 |
| 2299 | 743 | canrenoic acid | 10 µM | MCF7 | 0 | 0.069 | 0.07 | 6783 |
| 2300 | 663 | rescinnamine | 6 µM | MCF7 | 0 | 0.069 | 0.105 | 2785 |
| 2301 | 757 | rosiglitazone | 10 µM | MCF7 | 0 | 0.069 | 0.05 | 5593 |
| 2302 | 43 | sodium phenylbutyrate | 1 mM | MCF7 | 0 | 0.069 | 0.077 | 407 |
| 2303 | 1040 | Gly-His-Lys | 1 µM | PC3 | 0 | 0.069 | 0.075 | 6570 |
| 2304 | 735 | azacyclonol | 15 µM | MCF7 | 0 | 0.069 | 0.122 | 5398 |
| 2305 | 611 | josamycin | 5 µM | PC3 | 0 | 0.069 | 0.072 | 1950 |
| 2306 | 752 | strophanthidin | 10 µM | MCF7 | 0 | 0.069 | 0.106 | 6087 |
| 2307 | 659 | caffeic acid | 22 µM | HL60 | 0 | 0.069 | 0.084 | 3053 |
| 2308 | 744 | atropine oxide | 12 µM | MCF7 | 0 | 0.069 | 0.071 | 6812 |
| 2309 | 619 | bupivacaine | 12 µM | HL60 | 0 | 0.069 | 0.102 | 2404 |
| 2310 | 514 | cytochalasin B | 21 µM | MCF7 | 0 | 0.069 | 0.101 | 1122 |
| 2311 | 686 | bendroflumethiazide | 9 µM | MCF7 | 0 | 0.069 | 0.092 | 3840 |
| 2312 | 676 | pancuronium bromide | 5 µM | MCF7 | 0 | 0.069 | 0.087 | 7329 |
| 2313 | 708 | crotamiton | 20 µM | MCF7 | 0 | 0.069 | 0.101 | 5689 |
| 2314 | 705 | beclometasone | 8 µM | MCF7 | 0 | 0.069 | 0.142 | 4403 |
| 2315 | 771 | trichostatin A | 100 nM | MCF7 | 0 | 0.069 | 0.133 | 7407 |
| 2316 | 618 | proglumide | 12 µM | HL60 | 0 | 0.069 | 0.088 | 2363 |
| 2317 | 1058 | PF-00562151-00 | 10 µM | PC3 | 0 | 0.069 | 0.1 | 6907 |
| 2318 | 1014 | trichostatin A | 1 µM | MCF7 | 0 | 0.069 | 0.053 | 5976 |
| 2319 | 729 | hydrocortisone | 11 µM | MCF7 | 0 | 0.069 | 0.061 | 5284 |
| 2320 | 755 | terguride | 9 µM | MCF7 | 0 | 0.068 | 0.058 | 6473 |
| 2321 | 699 | Prestwick-665 | 12 µM | MCF7 | 0 | 0.068 | 0.076 | 4704 |
| 2322 | 694 | trichlormethiazide | 11 µM | MCF7 | 0 | 0.068 | 0.093 | 4783 |
| 2323 | 613 | azacyclonol | 15 µM | HL60 | 0 | 0.068 | 0.058 | 2020 |
| 2324 | 662 | syrosingopine | 6 µM | MCF7 | 0 | 0.068 | 0.063 | 2753 |
| 2325 | 685 | trichostatin A | 100 nM | MCF7 | 0 | 0.068 | 0.121 | 3643 |
| 2326 | 741 | diphenhydramine | 14 µM | MCF7 | 0 | 0.068 | 0.073 | 6020 |
| 2327 | 692 | zardaverine | 15 µM | PC3 | 0 | 0.068 | 0.033 | 4209 |
| 2328 | 636 | dizocilpine | 12 µM | MCF7 | 0 | 0.068 | 0.095 | 2232 |
| 2329 | 751 | isosorbide | 17 µM | MCF7 | 0 | 0.068 | 0.09 | 6038 |
| 2330 | 756 | alfadolone | 10 µM | MCF7 | 0 | 0.068 | 0.07 | 6506 |
| 2331 | 637 | tranylcypromine | 24 µM | MCF7 | 0 | 0.068 | 0.073 | 2264 |
| 2332 | 636 | atropine oxide | 12 µM | MCF7 | 0 | 0.068 | 0.102 | 2216 |
| 2333 | 737 | niflumic acid | 14 µM | MCF7 | 0 | 0.068 | 0.065 | 5490 |
| 2334 | 732 | saquinavir | 5 µM | PC3 | 0 | 0.068 | 0.082 | 5770 |
| 2335 | 752 | butamben | 21 µM | MCF7 | 0 | 0.068 | 0.077 | 6093 |
| 2336 | 513 | vorinostat | 10 µM | MCF7 | 0 | 0.068 | 0.102 | 1058 |
| 2337 | 614 | cefotaxime | 8 µM | HL60 | 0 | 0.068 | 0.121 | 1389 |
| 2338 | 762 | hydrastine hydrochloride | 10 µM | PC3 | 0 | 0.068 | 0.054 | 7309 |
| 2339 | 694 | trichostatin A | 100 nM | MCF7 | 0 | 0.068 | 0.1 | 4770 |
| 2340 | 513 | tanespimycin | 1 µM | MCF7 | 0 | 0.068 | 0.065 | 1064 |
| 2341 | 682 | citiolone | 25 µM | PC3 | 0 | 0.068 | 0.056 | 3755 |
| 2342 | 680 | metaraminol | 9 µM | PC3 | 0 | 0.068 | 0.112 | 3669 |
| 2343 | 719 | sulfadiazine | 16 µM | PC3 | 0 | 0.068 | 0.115 | 5098 |
| 2344 | 1055 | pioglitazone | 10 µM | MCF7 | 0 | 0.068 | 0.084 | 6898 |
| 2345 | 719 | phensuximide | 21 µM | PC3 | 0 | 0.068 | 0.08 | 5097 |
| 2346 | 751 | famprofazone | 11 µM | MCF7 | 0 | 0.068 | 0.1 | 6029 |
| 2347 | 729 | danazol | 12 µM | MCF7 | 0 | 0.068 | 0.095 | 5315 |
| 2348 | 505 | trifluoperazine | 10 µM | MCF7 | 0 | 0.068 | 0.06 | 910 |
| 2349 | 708 | bromocriptine | 5 µM | MCF7 | 0 | 0.068 | 0.094 | 5665 |
| 2350 | 699 | ofloxacin | 11 µM | MCF7 | 0 | 0.068 | 0.089 | 4696 |
| 2351 | 630 | chlorprothixene | 11 µM | HL60 | 0 | 0.068 | 0.116 | 1272 |
| 2352 | 671 | ribostamycin | 7 µM | MCF7 | 0 | 0.068 | 0.146 | 3444 |
| 2353 | 513 | valproic acid | 1 mM | MCF7 | 0 | 0.067 | 0.082 | 1047 |
| 2354 | 719 | bumetanide | 11 µM | PC3 | 0 | 0.067 | 0.072 | 5117 |
| 2355 | 677 | pyrvinium | 3 µM | MCF7 | 0 | 0.067 | 0.085 | 3518 |
| 2356 | 753 | esculin | 12 µM | PC3 | 0 | 0.067 | 0.066 | 6310 |
| 2357 | 505 | ionomycin | 2 µM | MCF7 | 0 | 0.067 | 0.069 | 882 |
| 2358 | 619 | trichostatin A | 100 nM | HL60 | 0 | 0.067 | 0.15 | 2375 |
| 2359 | 751 | helveticoside | 7 µM | MCF7 | 0 | 0.067 | 0.14 | 6047 |
| 2360 | 732 | lobeline | 11 µM | PC3 | 0 | 0.067 | 0.079 | 5784 |
| 2361 | 743 | acetylsalicylsalicylic acid | 13 µM | MCF7 | 0 | 0.067 | 0.057 | 6778 |
| 2362 | 659 | thioperamide | 10 µM | HL60 | 0 | 0.067 | 0.091 | 3055 |
| 2363 | 736 | trifluoperazine | 8 µM | MCF7 | 0 | 0.067 | 0.155 | 5442 |
| 2364 | 42 | alpha-estradiol | 10 nM | ssMCF7 | 0 | 0.067 | 0.071 | 403 |
| 2365 | 670 | monensin | 6 µM | MCF7 | 0 | 0.067 | 0.074 | 3443 |
| 2366 | 682 | bendroflumethiazide | 9 µM | PC3 | 0 | 0.067 | 0.083 | 3758 |
| 2367 | 678 | dorzolamide | 11 µM | MCF7 | 0 | 0.067 | 0.092 | 3565 |
| 2368 | 660 | rolipram | 15 µM | HL60 | 0 | 0.067 | 0.118 | 3072 |
| 2369 | 661 | protriptyline | 13 µM | HL60 | 0 | 0.067 | 0.167 | 3119 |
| 2370 | 650 | sirolimus | 100 nM | HL60 | 0 | 0.067 | 0.061 | 2681 |
| 2371 | 1030 | 5707885 | 50 µM | MCF7 | 0 | 0.067 | 0.083 | 6433 |
| 2372 | 603 | nordihydroguaiaretic acid | 1 µM | PC3 | 0 | 0.067 | 0.064 | 1223 |
| 2373 | 761 | asiaticoside | 4 µM | PC3 | 0 | 0.067 | 0.105 | 7244 |
| 2374 | 720 | parbendazole | 16 µM | MCF7 | 0 | 0.067 | 0.101 | 4357 |
| 2375 | 636 | terfenadine | 8 µM | MCF7 | 0 | 0.067 | 0.11 | 2227 |
| 2376 | 747 | tomatidine | 10 µM | MCF7 | 0 | 0.067 | 0.099 | 7166 |
| 2377 | 695 | 7-aminocephalosporanic acid | 15 µM | MCF7 | 0 | 0.067 | 0.059 | 4826 |
| 2378 | 629 | benzonatate | 7 µM | HL60 | 0 | 0.067 | 0.178 | 1843 |
| 2379 | 752 | clobetasol | 9 µM | MCF7 | 0 | 0.067 | 0.106 | 6095 |
| 2380 | 673 | fluvastatin | 9 µM | MCF7 | 0 | 0.067 | 0.115 | 3370 |
| 2381 | 690 | memantine | 19 µM | MCF7 | 0 | 0.067 | 0.067 | 4135 |
| 2382 | 645 | flunisolide | 9 µM | HL60 | 0 | 0.067 | 0.173 | 2168 |
| 2383 | 720 | ciclacillin | 12 µM | MCF7 | 0 | 0.067 | 0.091 | 4358 |
| 2384 | 693 | tolazoline | 20 µM | PC3 | 0 | 0.067 | 0.117 | 4262 |
| 2385 | 708 | bisacodyl | 11 µM | MCF7 | 0 | 0.067 | 0.067 | 5677 |
| 2386 | 1046 | PHA-00767505E | 10 µM | PC3 | 0 | 0.067 | 0.079 | 6591 |
| 2387 | 1091 | irinotecan | 100 µM | PC3 | 0 | 0.066 | 0.077 | 7535 |
| 2388 | 767 | trichostatin A | 100 nM | MCF7 | 0 | 0.066 | 0.121 | 6932 |
| 2389 | 758 | phenoxybenzamine | 12 µM | MCF7 | 0 | 0.066 | 0.09 | 5613 |
| 2390 | 767 | tanespimycin | 1 µM | MCF7 | 0 | 0.066 | 0.086 | 6944 |
| 2391 | 758 | enoxacin | 12 µM | MCF7 | 0 | 0.066 | 0.149 | 5616 |
| 2392 | 634 | bisacodyl | 11 µM | HL60 | 0 | 0.066 | 0.159 | 2435 |
| 2393 | 728 | trichostatin A | 100 nM | PC3 | 0 | 0.066 | 0.112 | 4483 |
| 2394 | 705 | etidronic acid | 16 µM | MCF7 | 0 | 0.066 | 0.125 | 4387 |
| 2395 | 98 | butein | 10 µM | MCF7 | 0 | 0.066 | 0.069 | 607 |
| 2396 | 506 | troglitazone | 10 µM | MCF7 | 0 | 0.066 | 0.077 | 1012 |
| 2397 | 771 | nortriptyline | 13 µM | MCF7 | 0 | 0.066 | 0.091 | 7422 |
| 2398 | 772 | cloxacillin | 9 µM | MCF7 | 0 | 0.066 | 0.081 | 7483 |
| 2399 | 1003 | thioridazine | 10 µM | PC3 | 0 | 0.066 | 0.073 | 5921 |
| 2400 | 1008 | AG-013608 | 10 µM | MCF7 | 0 | 0.066 | 0.078 | 5944 |
| 2401 | 694 | norcyclobenzaprine | 15 µM | MCF7 | 0 | 0.066 | 0.124 | 4776 |
| 2402 | 622 | streptomycin | 3 µM | HL60 | 0 | 0.066 | 0.126 | 1578 |
| 2403 | 657 | prenylamine | 10 µM | MCF7 | 0 | 0.066 | 0.091 | 2886 |
| 2404 | 755 | rolipram | 15 µM | MCF7 | 0 | 0.066 | 0.077 | 6449 |
| 2405 | 504 | monorden | 100 nM | MCF7 | 0 | 0.066 | 0.077 | 836 |
| 2406 | 513 | monorden | 100 nM | MCF7 | 0 | 0.066 | 0.036 | 1057 |
| 2407 | 55 | troglitazone | 10 µM | PC3 | 0 | 0.066 | 0.051 | 431 |
| 2408 | 736 | protriptyline | 13 µM | MCF7 | 0 | 0.066 | 0.109 | 5438 |
| 2409 | 673 | paracetamol | 26 µM | MCF7 | 0 | 0.066 | 0.15 | 3364 |
| 2410 | 682 | methylbenzethonium chloride | 9 µM | PC3 | 0 | 0.066 | 0.118 | 3768 |
| 2411 | 715 | lynestrenol | 14 µM | PC3 | 0 | 0.066 | 0.091 | 6756 |
| 2412 | 630 | dequalinium chloride | 8 µM | HL60 | 0 | 0.066 | 0.186 | 1276 |
| 2413 | 642 | nitrofurantoin | 17 µM | MCF7 | 0 | 0.066 | 0.114 | 2303 |
| 2414 | 755 | idazoxan | 17 µM | MCF7 | 0 | 0.066 | 0.053 | 6465 |
| 2415 | 738 | proguanil | 14 µM | MCF7 | 0 | 0.066 | 0.097 | 5506 |
| 2416 | 750 | valproic acid | 1 mM | HL60 | 0 | 0.066 | 0.153 | 6168 |
| 2417 | 708 | N-acetyl-L-leucine | 23 µM | MCF7 | 0 | 0.066 | 0.095 | 5683 |
| 2418 | 629 | sulfacetamide | 16 µM | HL60 | 0 | 0.066 | 0.171 | 1859 |
| 2419 | 673 | ganciclovir | 16 µM | MCF7 | 0 | 0.066 | 0.094 | 3368 |
| 2420 | 616 | neomycin | 4 µM | PC3 | 0 | 0.066 | 0.052 | 2066 |
| 2421 | 612 | 4-hydroxyphenazone | 20 µM | HL60 | 0 | 0.066 | 0.138 | 1997 |
| 2422 | 672 | furazolidone | 18 µM | MCF7 | 0 | 0.066 | 0.076 | 3358 |
| 2423 | 756 | articaine | 12 µM | MCF7 | 0 | 0.066 | 0.095 | 6517 |
| 2424 | 751 | beta-escin | 3 µM | MCF7 | 0 | 0.066 | 0.074 | 6050 |
| 2425 | 654 | digitoxigenin | 11 µM | MCF7 | 0 | 0.066 | 0.083 | 3275 |
| 2426 | 641 | pyridoxine | 19 µM | HL60 | 0 | 0.066 | 0.15 | 1759 |
| 2427 | 738 | bufexamac | 18 µM | MCF7 | 0 | 0.066 | 0.097 | 5515 |
| 2428 | 660 | N-acetyl-L-leucine | 23 µM | HL60 | 0 | 0.066 | 0.156 | 3085 |
| 2429 | 653 | amoxicillin | 11 µM | MCF7 | 0 | 0.066 | 0.115 | 2620 |
| 2430 | 629 | chloramphenicol | 12 µM | HL60 | 0 | 0.065 | 0.192 | 1837 |
| 2431 | 704 | bacampicillin | 8 µM | PC3 | 0 | 0.065 | 0.097 | 4592 |
| 2432 | 612 | oxetacaine | 9 µM | HL60 | 0 | 0.065 | 0.113 | 1984 |
| 2433 | 756 | proxymetacaine | 12 µM | MCF7 | 0 | 0.065 | 0.073 | 6492 |
| 2434 | 630 | bisoprolol | 9 µM | HL60 | 0 | 0.065 | 0.157 | 1287 |
| 2435 | 699 | merbromin | 5 µM | MCF7 | 0 | 0.065 | 0.104 | 4722 |
| 2436 | 702 | deferoxamine | 6 µM | PC3 | 0 | 0.065 | 0.128 | 4317 |
| 2437 | 745 | phenazopyridine | 16 µM | MCF7 | 0 | 0.065 | 0.096 | 6234 |
| 2438 | 1038 | 16,16-dimethylprostaglandin E2 | 10 µM | PC3 | 0 | 0.065 | 0.068 | 6562 |
| 2439 | 664 | meptazinol | 15 µM | HL60 | 0 | 0.065 | 0.144 | 2906 |
| 2440 | 627 | triflupromazine | 10 µM | MCF7 | 0 | 0.065 | 0.087 | 1691 |
| 2441 | 690 | gramine | 23 µM | MCF7 | 0 | 0.065 | 0.08 | 4118 |
| 2442 | 663 | xylazine | 18 µM | MCF7 | 0 | 0.065 | 0.087 | 2788 |
| 2443 | 610 | naphazoline | 16 µM | PC3 | 0 | 0.065 | 0.059 | 1886 |
| 2444 | 680 | 0173570-0000 | 1 µM | PC3 | 0 | 0.065 | 0.08 | 3693 |
| 2445 | 109 | colchicine | 1 µM | SKMEL5 | 0 | 0.065 | 0.148 | 630 |
| 2446 | 1067 | alsterpaullone | 10 µM | PC3 | 0 | 0.065 | 0.126 | 7056 |
| 2447 | 737 | pirlindole | 12 µM | MCF7 | 0 | 0.065 | 0.107 | 5497 |
| 2448 | 690 | trichostatin A | 100 nM | MCF7 | 0 | 0.065 | 0.104 | 4112 |
| 2449 | 632 | doxylamine | 10 µM | MCF7 | 0 | 0.065 | 0.072 | 1473 |
| 2450 | 753 | chlorpropamide | 14 µM | PC3 | 0 | 0.065 | 0.083 | 6291 |
| 2451 | 704 | sparteine | 17 µM | PC3 | 0 | 0.065 | 0.067 | 4568 |
| 2452 | 504 | 5224221 | 12 µM | MCF7 | 0 | 0.065 | 0.08 | 839 |
| 2453 | 758 | 3-acetylcoumarin | 21 µM | MCF7 | 0 | 0.065 | 0.059 | 5624 |
| 2454 | 732 | dexamethasone | 9 µM | PC3 | 0 | 0.065 | 0.094 | 5797 |
| 2455 | 764 | mebeverine | 9 µM | PC3 | 0 | 0.065 | 0.074 | 7147 |
| 2456 | 1020 | orlistat | 10 µM | MCF7 | 0 | 0.065 | 0.052 | 6383 |
| 2457 | 504 | rottlerin | 10 µM | MCF7 | 0 | 0.065 | 0.056 | 825 |
| 2458 | 688 | amiloride | 13 µM | PC3 | 0 | 0.065 | 0.076 | 3990 |
| 2459 | 708 | ritodrine | 12 µM | MCF7 | 0 | 0.065 | 0.133 | 5680 |
| 2460 | 691 | tolmetin | 13 µM | MCF7 | 0 | 0.065 | 0.052 | 4167 |
| 2461 | 746 | alpha-yohimbine | 10 µM | MCF7 | 0 | 0.065 | 0.055 | 6274 |
| 2462 | 683 | thiamazole | 35 µM | PC3 | 0 | 0.065 | 0.056 | 3815 |
| 2463 | 741 | imipenem | 13 µM | MCF7 | 0 | 0.065 | 0.049 | 5997 |
| 2464 | 708 | androsterone | 14 µM | MCF7 | 0 | 0.065 | 0.059 | 5696 |
| 2465 | 1052 | trichostatin A | 1 µM | PC3 | 0 | 0.065 | 0.107 | 6886 |
| 2466 | 664 | letrozole | 14 µM | HL60 | 0 | 0.065 | 0.219 | 2916 |
| 2467 | 633 | ciprofloxacin | 11 µM | MCF7 | 0 | 0.065 | 0.066 | 1522 |
| 2468 | 748 | leflunomide | 15 µM | MCF7 | 0 | 0.064 | 0.094 | 7238 |
| 2469 | 678 | saquinavir | 5 µM | MCF7 | 0 | 0.064 | 0.105 | 3549 |
| 2470 | 662 | dipivefrine | 10 µM | MCF7 | 0 | 0.064 | 0.07 | 2744 |
| 2471 | 694 | galantamine | 11 µM | MCF7 | 0 | 0.064 | 0.097 | 4772 |
| 2472 | 506 | trifluoperazine | 10 µM | MCF7 | 0 | 0.064 | 0.06 | 1004 |
| 2473 | 611 | methotrexate | 9 µM | PC3 | 0 | 0.064 | 0.075 | 1957 |
| 2474 | 673 | luteolin | 14 µM | MCF7 | 0 | 0.064 | 0.114 | 3379 |
| 2475 | 748 | sulfadoxine | 13 µM | MCF7 | 0 | 0.064 | 0.057 | 7205 |
| 2476 | 731 | guanethidine | 13 µM | PC3 | 0 | 0.064 | 0.082 | 5731 |
| 2477 | 645 | fludroxycortide | 9 µM | HL60 | 0 | 0.064 | 0.129 | 2184 |
| 2478 | 1053 | SB-202190 | 1 µM | MCF7 | 0 | 0.064 | 0.085 | 6887 |
| 2479 | 664 | molindone | 13 µM | HL60 | 0 | 0.064 | 0.221 | 2917 |
| 2480 | 663 | trimethylcolchicinic acid | 12 µM | MCF7 | 0 | 0.064 | 0.104 | 2802 |
| 2481 | 672 | trimipramine | 10 µM | MCF7 | 0 | 0.064 | 0.082 | 3342 |
| 2482 | 726 | butyl hydroxybenzoate | 21 µM | MCF7 | 0 | 0.064 | 0.04 | 5245 |
| 2483 | 623 | probucol | 8 µM | HL60 | 0 | 0.064 | 0.143 | 1608 |
| 2484 | 747 | ursolic acid | 9 µM | MCF7 | 0 | 0.064 | 0.065 | 7181 |
| 2485 | 729 | flupentixol | 8 µM | MCF7 | 0 | 0.064 | 0.074 | 5307 |
| 2486 | 603 | trifluoperazine | 10 µM | PC3 | 0 | 0.064 | 0.093 | 1224 |
| 2487 | 762 | lansoprazole | 11 µM | PC3 | 0 | 0.064 | 0.081 | 7306 |
| 2488 | 706 | lysergol | 16 µM | MCF7 | 0 | 0.064 | 0.106 | 4966 |
| 2489 | 659 | luteolin | 14 µM | HL60 | 0 | 0.064 | 0.142 | 3041 |
| 2490 | 676 | trichostatin A | 100 nM | MCF7 | 0 | 0.064 | 0.099 | 7324 |
| 2491 | 615 | pentoxifylline | 14 µM | HL60 | 0 | 0.064 | 0.125 | 1444 |
| 2492 | 659 | equilin | 15 µM | HL60 | 0 | 0.064 | 0.112 | 3039 |
| 2493 | 1095 | CP-863187 | 10 µM | PC3 | 0 | 0.064 | 0.088 | 7558 |
| 2494 | 642 | ofloxacin | 11 µM | MCF7 | 0 | 0.064 | 0.087 | 2302 |
| 2495 | 648 | etanidazole | 19 µM | HL60 | 0 | 0.064 | 0.101 | 2510 |
| 2496 | 753 | clebopride | 8 µM | PC3 | 0 | 0.064 | 0.077 | 6311 |
| 2497 | 633 | apomorphine | 6 µM | MCF7 | 0 | 0.064 | 0.111 | 1505 |
| 2498 | 690 | azacitidine | 16 µM | MCF7 | 0 | 0.064 | 0.119 | 4128 |
| 2499 | 1011 | PF-00562151-00 | 10 µM | PC3 | 0 | 0.064 | 0.093 | 5959 |
| 2500 | 1005 | pioglitazone | 10 µM | PC3 | 0 | 0.064 | 0.059 | 5930 |
| 2501 | 690 | ginkgolide A | 10 µM | MCF7 | 0 | 0.064 | 0.131 | 4121 |
| 2502 | 730 | rolipram | 15 µM | MCF7 | 0 | 0.063 | 0.06 | 5330 |
| 2503 | 772 | trapidil | 19 µM | MCF7 | 0 | 0.063 | 0.054 | 7475 |
| 2504 | 1046 | 16,16-dimethylprostaglandin E2 | 10 µM | PC3 | 0 | 0.063 | 0.075 | 6592 |
| 2505 | 673 | sulconazole | 9 µM | MCF7 | 0 | 0.063 | 0.182 | 3373 |
| 2506 | 1029 | 0225151-0000 | 10 µM | PC3 | 0 | 0.063 | 0.111 | 6426 |
| 2507 | 714 | ciprofloxacin | 11 µM | PC3 | 0 | 0.063 | 0.058 | 6700 |
| 2508 | 642 | 6-benzylaminopurine | 18 µM | MCF7 | 0 | 0.063 | 0.132 | 2313 |
| 2509 | 1028 | C-75 | 10 µM | MCF7 | 0 | 0.063 | 0.091 | 6423 |
| 2510 | 691 | thioridazine | 10 µM | MCF7 | 0 | 0.063 | 0.139 | 4164 |
| 2511 | 734 | fluphenazine | 8 µM | PC3 | 0 | 0.063 | 0.111 | 5880 |
| 2512 | 1024 | 3-nitropropionic acid | 10 µM | MCF7 | 0 | 0.063 | 0.056 | 6402 |
| 2513 | 116 | butirosin | 10 µM | PC3 | 0 | 0.063 | 0.096 | 666 |
| 2514 | 762 | harmol | 16 µM | PC3 | 0 | 0.063 | 0.083 | 7320 |
| 2515 | 661 | isradipine | 11 µM | HL60 | 0 | 0.063 | 0.189 | 3129 |
| 2516 | 705 | chloropyramine | 12 µM | MCF7 | 0 | 0.063 | 0.076 | 4414 |
| 2517 | 767 | sirolimus | 100 nM | MCF7 | 0 | 0.063 | 0.059 | 6940 |
| 2518 | 663 | harman | 18 µM | MCF7 | 0 | 0.063 | 0.099 | 2806 |
| 2519 | 713 | amiodarone | 6 µM | PC3 | 0 | 0.063 | 0.118 | 4657 |
| 2520 | 613 | hesperetin | 13 µM | HL60 | 0 | 0.063 | 0.096 | 2031 |
| 2521 | 657 | trimetazidine | 12 µM | MCF7 | 0 | 0.063 | 0.104 | 2876 |
| 2522 | 743 | piperidolate | 11 µM | MCF7 | 0 | 0.063 | 0.072 | 6772 |
| 2523 | 513 | prochlorperazine | 10 µM | MCF7 | 0 | 0.063 | 0.114 | 1053 |
| 2524 | 41 | estradiol | 10 nM | HL60 | 0 | 0.063 | 0.091 | 387 |
| 2525 | 736 | 10-methoxyharmalan | 19 µM | MCF7 | 0 | 0.063 | 0.062 | 5455 |
| 2526 | 656 | betahistine | 17 µM | MCF7 | 0 | 0.063 | 0.059 | 2833 |
| 2527 | 750 | chlorpromazine | 1 µM | HL60 | 0 | 0.063 | 0.142 | 6176 |
| 2528 | 58 | oxamic acid | 10 mM | MCF7 | 0 | 0.063 | 0.055 | 439 |
| 2529 | 637 | hyoscyamine | 14 µM | MCF7 | 0 | 0.063 | 0.082 | 2271 |
| 2530 | 613 | apomorphine | 6 µM | HL60 | 0 | 0.063 | 0.123 | 2005 |
| 2531 | 680 | cefazolin | 8 µM | PC3 | 0 | 0.063 | 0.055 | 3686 |
| 2532 | 751 | fludroxycortide | 9 µM | MCF7 | 0 | 0.063 | 0.059 | 6039 |
| 2533 | 720 | orphenadrine | 13 µM | MCF7 | 0 | 0.063 | 0.113 | 4359 |
| 2534 | 1026 | PF-00539758-00 | 10 µM | MCF7 | 0 | 0.063 | 0.118 | 6416 |
| 2535 | 753 | cefoperazone | 6 µM | PC3 | 0 | 0.063 | 0.054 | 6323 |
| 2536 | 612 | amylocaine | 15 µM | HL60 | 0 | 0.063 | 0.166 | 1991 |
| 2537 | 737 | metformin | 24 µM | MCF7 | 0 | 0.063 | 0.066 | 5487 |
| 2538 | 750 | tanespimycin | 1 µM | HL60 | 0 | 0.062 | 0.143 | 6166 |
| 2539 | 654 | carisoprodol | 15 µM | MCF7 | 0 | 0.062 | 0.053 | 3251 |
| 2540 | 713 | clioquinol | 13 µM | PC3 | 0 | 0.062 | 0.092 | 4663 |
| 2541 | 705 | pancuronium bromide | 5 µM | MCF7 | 0 | 0.062 | 0.079 | 4393 |
| 2542 | 648 | anabasine | 25 µM | HL60 | 0 | 0.062 | 0.093 | 2512 |
| 2543 | 665 | pyrvinium | 3 µM | HL60 | 0 | 0.062 | 0.203 | 2957 |
| 2544 | 766 | talampicillin | 8 µM | MCF7 | 0 | 0.062 | 0.051 | 7014 |
| 2545 | 650 | LY-294002 | 10 µM | HL60 | 0 | 0.062 | 0.107 | 2687 |
| 2546 | 646 | alprenolol | 14 µM | MCF7 | 0 | 0.062 | 0.049 | 3188 |
| 2547 | 749 | oxprenolol | 13 µM | HL60 | 0 | 0.062 | 0.14 | 6145 |
| 2548 | 614 | dizocilpine | 12 µM | HL60 | 0 | 0.062 | 0.127 | 1386 |
| 2549 | 714 | urapidil | 9 µM | PC3 | 0 | 0.062 | 0.048 | 6696 |
| 2550 | 706 | protoveratrine A | 5 µM | MCF7 | 0 | 0.062 | 0.064 | 4963 |
| 2551 | 1007 | PF-00539745-00 | 10 µM | PC3 | 0 | 0.062 | 0.078 | 5939 |
| 2552 | 698 | PNU-0251126 | 10 µM | PC3 | 0 | 0.062 | 0.053 | 7388 |
| 2553 | 646 | oxytetracycline | 8 µM | MCF7 | 0 | 0.062 | 0.076 | 3170 |
| 2554 | 602 | geldanamycin | 1 µM | HL60 | 0 | 0.062 | 0.153 | 1169 |
| 2555 | 98 | monastrol | 20 µM | MCF7 | 0 | 0.062 | 0.065 | 605 |
| 2556 | 648 | fluorometholone | 11 µM | HL60 | 0 | 0.062 | 0.173 | 2509 |
| 2557 | 749 | mesalazine | 26 µM | HL60 | 0 | 0.062 | 0.188 | 6162 |
| 2558 | 1035 | 5194442 | 20 µM | MCF7 | 0 | 0.062 | 0.075 | 6558 |
| 2559 | 751 | promazine | 12 µM | MCF7 | 0 | 0.062 | 0.103 | 6028 |
| 2560 | 732 | hydroquinine | 9 µM | PC3 | 0 | 0.062 | 0.063 | 5789 |
| 2561 | 726 | probucol | 8 µM | MCF7 | 0 | 0.062 | 0.117 | 5261 |
| 2562 | 16 | LM-1685 | 10 µM | MCF7 | 0 | 0.062 | 0.112 | 207 |
| 2563 | 653 | clozapine | 12 µM | MCF7 | 0 | 0.062 | 0.077 | 2644 |
| 2564 | 614 | mianserin | 13 µM | HL60 | 0 | 0.062 | 0.115 | 1385 |
| 2565 | 648 | skimmianine | 15 µM | HL60 | 0 | 0.062 | 0.172 | 2504 |
| 2566 | 623 | chlorpropamide | 14 µM | HL60 | 0 | 0.062 | 0.13 | 1594 |
| 2567 | 1003 | rotenone | 1 µM | PC3 | 0 | 0.062 | 0.097 | 5920 |
| 2568 | 712 | altretamine | 19 µM | PC3 | 0 | 0.062 | 0.091 | 4627 |
| 2569 | 642 | tiapride | 11 µM | MCF7 | 0 | 0.062 | 0.073 | 2292 |
| 2570 | 645 | famprofazone | 11 µM | HL60 | 0 | 0.062 | 0.196 | 2174 |
| 2571 | 514 | (-)-catechin | 11 µM | MCF7 | 0 | 0.062 | 0.061 | 1101 |
| 2572 | 700 | sulfamethoxypyridazine | 14 µM | MCF7 | 0 | 0.062 | 0.102 | 4733 |
| 2573 | 750 | LY-294002 | 100 nM | HL60 | 0 | 0.062 | 0.169 | 6175 |
| 2574 | 1047 | 5194442 | 20 µM | MCF7 | 0 | 0.062 | 0.057 | 6599 |
| 2575 | 753 | dobutamine | 12 µM | PC3 | 0 | 0.062 | 0.08 | 6286 |
| 2576 | 664 | etamsylate | 15 µM | HL60 | 0 | 0.062 | 0.224 | 2915 |
| 2577 | 506 | thioridazine | 10 µM | MCF7 | 0 | 0.062 | 0.053 | 1010 |
| 2578 | 750 | acetylsalicylic acid | 100 µM | HL60 | 0 | 0.062 | 0.189 | 6164 |
| 2579 | 617 | aztreonam | 9 µM | PC3 | 0 | 0.062 | 0.073 | 2118 |
| 2580 | 654 | corticosterone | 12 µM | MCF7 | 0 | 0.061 | 0.067 | 3244 |
| 2581 | 616 | pindolol | 16 µM | PC3 | 0 | 0.061 | 0.056 | 2075 |
| 2582 | 664 | fursultiamine | 9 µM | HL60 | 0 | 0.061 | 0.196 | 2929 |
| 2583 | 720 | chlorambucil | 13 µM | MCF7 | 0 | 0.061 | 0.107 | 4345 |
| 2584 | 771 | homochlorcyclizine | 10 µM | MCF7 | 0 | 0.061 | 0.111 | 7417 |
| 2585 | 109 | novobiocin | 100 µM | SKMEL5 | 0 | 0.061 | 0.14 | 632 |
| 2586 | 747 | piperacetazine | 10 µM | MCF7 | 0 | 0.061 | 0.093 | 7191 |
| 2587 | 753 | metolazone | 11 µM | PC3 | 0 | 0.061 | 0.054 | 6292 |
| 2588 | 734 | vinpocetine | 11 µM | PC3 | 0 | 0.061 | 0.117 | 5859 |
| 2589 | 1054 | scriptaid | 10 µM | PC3 | 0 | 0.061 | 0.166 | 6896 |
| 2590 | 693 | simvastatin | 10 µM | PC3 | 0 | 0.061 | 0.124 | 4244 |
| 2591 | 695 | seneciphylline | 12 µM | MCF7 | 0 | 0.061 | 0.067 | 4822 |
| 2592 | 627 | sulfaphenazole | 13 µM | MCF7 | 0 | 0.061 | 0.101 | 1673 |
| 2593 | 695 | cyclobenzaprine | 13 µM | MCF7 | 0 | 0.061 | 0.087 | 4834 |
| 2594 | 708 | chlormezanone | 15 µM | MCF7 | 0 | 0.061 | 0.059 | 5697 |
| 2595 | 753 | chlorhexidine | 8 µM | PC3 | 0 | 0.061 | 0.104 | 6302 |
| 2596 | 715 | fluoxetine | 12 µM | PC3 | 0 | 0.061 | 0.074 | 6757 |
| 2597 | 729 | estradiol | 15 µM | MCF7 | 0 | 0.061 | 0.067 | 5318 |
| 2598 | 662 | physostigmine | 6 µM | MCF7 | 0 | 0.061 | 0.086 | 2768 |
| 2599 | 616 | isoniazid | 29 µM | PC3 | 0 | 0.061 | 0.085 | 2083 |
| 2600 | 750 | valproic acid | 200 µM | HL60 | 0 | 0.061 | 0.143 | 6173 |
| 2601 | 611 | famotidine | 12 µM | PC3 | 0 | 0.061 | 0.075 | 1946 |
| 2602 | 682 | sulfachlorpyridazine | 14 µM | PC3 | 0 | 0.061 | 0.072 | 3769 |
| 2603 | 734 | gentamicin | 3 µM | PC3 | 0 | 0.061 | 0.068 | 5883 |
| 2604 | 737 | trimetazidine | 12 µM | MCF7 | 0 | 0.061 | 0.097 | 5479 |
| 2605 | 665 | risperidone | 10 µM | HL60 | 0 | 0.061 | 0.148 | 2947 |
| 2606 | 612 | idoxuridine | 11 µM | HL60 | 0 | 0.061 | 0.17 | 1980 |
| 2607 | 18 | LM-1685 | 10 µM | MCF7 | 0 | 0.061 | 0.079 | 253 |
| 2608 | 616 | acetylsalicylsalicylic acid | 13 µM | PC3 | 0 | 0.061 | 0.069 | 2061 |
| 2609 | 655 | ergocalciferol | 10 µM | MCF7 | 0 | 0.061 | 0.091 | 3304 |
| 2610 | 709 | cefaclor | 10 µM | PC3 | 0 | 0.061 | 0.11 | 6622 |
| 2611 | 655 | colecalciferol | 10 µM | MCF7 | 0 | 0.061 | 0.055 | 3298 |
| 2612 | 710 | fluspirilene | 8 µM | PC3 | 0 | 0.061 | 0.112 | 6662 |
| 2613 | 1069 | GW-8510 | 10 µM | PC3 | 0 | 0.061 | 0.13 | 7067 |
| 2614 | 1029 | C-75 | 10 µM | PC3 | 0 | 0.061 | 0.06 | 6428 |
| 2615 | 685 | Prestwick-674 | 14 µM | MCF7 | 0 | 0.061 | 0.103 | 3614 |
| 2616 | 627 | dexpanthenol | 19 µM | MCF7 | 0 | 0.061 | 0.066 | 1680 |
| 2617 | 744 | altizide | 10 µM | MCF7 | 0 | 0.061 | 0.084 | 6829 |
| 2618 | 645 | promazine | 12 µM | HL60 | 0 | 0.061 | 0.176 | 2173 |
| 2619 | 730 | metergoline | 10 µM | MCF7 | 0 | 0.061 | 0.044 | 5344 |
| 2620 | 700 | tropicamide | 14 µM | MCF7 | 0 | 0.061 | 0.077 | 4744 |
| 2621 | 708 | procainamide | 15 µM | MCF7 | 0 | 0.061 | 0.153 | 5663 |
| 2622 | 730 | neostigmine bromide | 13 µM | MCF7 | 0 | 0.061 | 0.065 | 5335 |
| 2623 | 2 | phenformin | 10 µM | MCF7 | 0 | 0.061 | 0.063 | 21 |
| 2624 | 731 | dienestrol | 15 µM | PC3 | 0 | 0.06 | 0.053 | 5727 |
| 2625 | 700 | levonorgestrel | 13 µM | MCF7 | 0 | 0.06 | 0.073 | 4730 |
| 2626 | 681 | diphenylpyraline | 13 µM | PC3 | 0 | 0.06 | 0.054 | 3743 |
| 2627 | 657 | scoulerine | 12 µM | MCF7 | 0 | 0.06 | 0.109 | 2891 |
| 2628 | 671 | pipenzolate bromide | 9 µM | MCF7 | 0 | 0.06 | 0.081 | 3460 |
| 2629 | 714 | flupentixol | 8 µM | PC3 | 0 | 0.06 | 0.123 | 6708 |
| 2630 | 612 | ethambutol | 14 µM | HL60 | 0 | 0.06 | 0.113 | 1981 |
| 2631 | 672 | (+/-)-catechin | 14 µM | MCF7 | 0 | 0.06 | 0.068 | 3351 |
| 2632 | 729 | loxapine | 9 µM | MCF7 | 0 | 0.06 | 0.073 | 5293 |
| 2633 | 746 | methacholine chloride | 20 µM | MCF7 | 0 | 0.06 | 0.08 | 6248 |
| 2634 | 659 | digoxigenin | 10 µM | HL60 | 0 | 0.06 | 0.128 | 3060 |
| 2635 | 737 | antimycin A | 7 µM | MCF7 | 0 | 0.06 | 0.104 | 5472 |
| 2636 | 751 | ethotoin | 20 µM | MCF7 | 0 | 0.06 | 0.076 | 6052 |
| 2637 | 748 | glibenclamide | 8 µM | MCF7 | 0 | 0.06 | 0.097 | 7202 |
| 2638 | 640 | laudanosine | 11 µM | HL60 | 0 | 0.06 | 0.134 | 1741 |
| 2639 | 82 | tetraethylenepentamine | 100 µM | MCF7 | 0 | 0.06 | 0.081 | 574 |
| 2640 | 718 | lobelanidine | 11 µM | PC3 | 0 | 0.06 | 0.081 | 5080 |
| 2641 | 694 | digitoxigenin | 11 µM | MCF7 | 0 | 0.06 | 0.091 | 4801 |
| 2642 | 719 | cyclizine | 13 µM | PC3 | 0 | 0.06 | 0.057 | 5100 |
| 2643 | 709 | protoveratrine A | 5 µM | PC3 | 0 | 0.06 | 0.054 | 6618 |
| 2644 | 615 | etodolac | 14 µM | HL60 | 0 | 0.06 | 0.101 | 1407 |
| 2645 | 504 | ikarugamycin | 2 µM | MCF7 | 0 | 0.06 | 0.077 | 866 |
| 2646 | 655 | meclozine | 9 µM | MCF7 | 0 | 0.06 | 0.062 | 3285 |
| 2647 | 513 | LY-294002 | 100 nM | MCF7 | 0 | 0.06 | 0.099 | 1054 |
| 2648 | 63 | copper sulfate | 100 µM | PC3 | 0 | 0.06 | 0.1 | 459 |
| 2649 | 729 | tacrine | 16 µM | MCF7 | 0 | 0.06 | 0.06 | 5297 |
| 2650 | 691 | cimetidine | 16 µM | MCF7 | 0 | 0.06 | 0.107 | 4144 |
| 2651 | 1002 | thioridazine | 10 µM | MCF7 | 0 | 0.06 | 0.045 | 5916 |
| 2652 | 766 | xylometazoline | 14 µM | MCF7 | 0 | 0.06 | 0.068 | 7020 |
| 2653 | 505 | 5213008 | 18 µM | MCF7 | 0 | 0.06 | 0.054 | 898 |
| 2654 | 651 | azlocillin | 8 µM | HL60 | 0 | 0.059 | 0.087 | 2727 |
| 2655 | 754 | scopolamine N-oxide | 10 µM | PC3 | 0 | 0.059 | 0.075 | 6335 |
| 2656 | 751 | benfotiamine | 9 µM | MCF7 | 0 | 0.059 | 0.136 | 6032 |
| 2657 | 653 | pentoxyverine | 8 µM | MCF7 | 0 | 0.059 | 0.103 | 2623 |
| 2658 | 762 | metrifonate | 16 µM | PC3 | 0 | 0.059 | 0.06 | 7287 |
| 2659 | 692 | pyrimethamine | 16 µM | PC3 | 0 | 0.059 | 0.098 | 4194 |
| 2660 | 670 | cefazolin | 8 µM | MCF7 | 0 | 0.059 | 0.101 | 3426 |
| 2661 | 698 | mebendazole | 14 µM | PC3 | 0 | 0.059 | 0.078 | 7370 |
| 2662 | 705 | piromidic acid | 14 µM | MCF7 | 0 | 0.059 | 0.077 | 4398 |
| 2663 | 737 | homatropine | 11 µM | MCF7 | 0 | 0.059 | 0.073 | 5477 |
| 2664 | 663 | dydrogesterone | 13 µM | MCF7 | 0 | 0.059 | 0.105 | 2811 |
| 2665 | 672 | nafcillin | 9 µM | MCF7 | 0 | 0.059 | 0.097 | 3323 |
| 2666 | 761 | pergolide | 10 µM | PC3 | 0 | 0.059 | 0.061 | 7271 |
| 2667 | 640 | tubocurarine chloride | 5 µM | HL60 | 0 | 0.059 | 0.102 | 1738 |
| 2668 | 758 | cotinine | 23 µM | MCF7 | 0 | 0.059 | 0.124 | 5611 |
| 2669 | 660 | metyrapone | 18 µM | HL60 | 0 | 0.059 | 0.155 | 3070 |
| 2670 | 1081 | BCB000040 | 10 µM | PC3 | 0 | 0.059 | 0.065 | 7493 |
| 2671 | 646 | bupropion | 14 µM | MCF7 | 0 | 0.059 | 0.135 | 3180 |
| 2672 | 707 | bezafibrate | 11 µM | MCF7 | 0 | 0.059 | 0.068 | 4999 |
| 2673 | 749 | kaempferol | 14 µM | HL60 | 0 | 0.059 | 0.123 | 6157 |
| 2674 | 671 | methacholine chloride | 20 µM | MCF7 | 0 | 0.059 | 0.072 | 3452 |
| 2675 | 744 | cyproterone | 10 µM | MCF7 | 0 | 0.059 | 0.09 | 6806 |
| 2676 | 729 | mimosine | 20 µM | MCF7 | 0 | 0.059 | 0.082 | 5302 |
| 2677 | 1092 | IC-86621 | 1 µM | MCF7 | 0 | 0.059 | 0.1 | 7543 |
| 2678 | 772 | chlorzoxazone | 24 µM | MCF7 | 0 | 0.059 | 0.122 | 7456 |
| 2679 | 709 | miconazole | 10 µM | PC3 | 0 | 0.059 | 0.078 | 6615 |
| 2680 | 743 | butirosin | 5 µM | MCF7 | 0 | 0.059 | 0.074 | 6779 |
| 2681 | 650 | valproic acid | 200 µM | HL60 | 0 | 0.059 | 0.134 | 2674 |
| 2682 | 682 | proglumide | 12 µM | PC3 | 0 | 0.059 | 0.059 | 3780 |
| 2683 | 704 | pentamidine | 7 µM | PC3 | 0 | 0.059 | 0.063 | 4573 |
| 2684 | 616 | ivermectin | 5 µM | PC3 | 0 | 0.059 | 0.094 | 2051 |
| 2685 | 646 | mifepristone | 9 µM | MCF7 | 0 | 0.059 | 0.126 | 3185 |
| 2686 | 671 | carbinoxamine | 10 µM | MCF7 | 0 | 0.059 | 0.131 | 3466 |
| 2687 | 660 | prazosin | 10 µM | HL60 | 0 | 0.059 | 0.137 | 3098 |
| 2688 | 709 | lovastatin | 10 µM | PC3 | 0 | 0.058 | 0.06 | 6633 |
| 2689 | 751 | solasodine | 10 µM | MCF7 | 0 | 0.058 | 0.106 | 6025 |
| 2690 | 637 | nifenazone | 13 µM | MCF7 | 0 | 0.058 | 0.07 | 2285 |
| 2691 | 709 | betahistine | 17 µM | PC3 | 0 | 0.058 | 0.054 | 6611 |
| 2692 | 660 | desoxycortone | 12 µM | HL60 | 0 | 0.058 | 0.168 | 3099 |
| 2693 | 725 | LY-294002 | 100 nM | MCF7 | 0 | 0.058 | 0.065 | 5213 |
| 2694 | 505 | colforsin | 50 µM | MCF7 | 0 | 0.058 | 0.063 | 913 |
| 2695 | 756 | nisoxetine | 13 µM | MCF7 | 0 | 0.058 | 0.107 | 6496 |
| 2696 | 695 | (+/-)-catechin | 14 µM | MCF7 | 0 | 0.058 | 0.074 | 4837 |
| 2697 | 730 | zidovudine | 15 µM | MCF7 | 0 | 0.058 | 0.055 | 5333 |
| 2698 | 681 | tropicamide | 14 µM | PC3 | 0 | 0.058 | 0.096 | 3722 |
| 2699 | 738 | adipiodone | 4 µM | MCF7 | 0 | 0.058 | 0.077 | 5510 |
| 2700 | 747 | cinchonidine | 14 µM | MCF7 | 0 | 0.058 | 0.09 | 7190 |
| 2701 | 685 | meclofenoxate | 14 µM | MCF7 | 0 | 0.058 | 0.155 | 3605 |
| 2702 | 1092 | BCB000038 | 10 µM | MCF7 | 0 | 0.058 | 0.063 | 7542 |
| 2703 | 615 | chlorzoxazone | 24 µM | HL60 | 0 | 0.058 | 0.072 | 1416 |
| 2704 | 736 | proxymetacaine | 12 µM | MCF7 | 0 | 0.058 | 0.076 | 5433 |
| 2705 | 749 | cyproterone | 10 µM | HL60 | 0 | 0.058 | 0.103 | 6124 |
| 2706 | 662 | dacarbazine | 22 µM | MCF7 | 0 | 0.058 | 0.101 | 2754 |
| 2707 | 35 | sirolimus | 100 nM | HL60 | 0 | 0.058 | 0.151 | 362 |
| 2708 | 649 | guaifenesin | 20 µM | HL60 | 0 | 0.058 | 0.117 | 2569 |
| 2709 | 748 | delsoline | 9 µM | MCF7 | 0 | 0.058 | 0.08 | 7212 |
| 2710 | 664 | pronetalol | 15 µM | HL60 | 0 | 0.058 | 0.213 | 2902 |
| 2711 | 59 | oligomycin | 1 µM | MCF7 | 0 | 0.058 | 0.076 | 442 |
| 2712 | 690 | amiloride | 13 µM | MCF7 | 0 | 0.058 | 0.078 | 4109 |
| 2713 | 714 | puromycin | 7 µM | PC3 | 0 | 0.058 | 0.046 | 6711 |
| 2714 | 629 | acetazolamide | 18 µM | HL60 | 0 | 0.058 | 0.12 | 1850 |
| 2715 | 642 | lomefloxacin | 10 µM | MCF7 | 0 | 0.058 | 0.078 | 2310 |
| 2716 | 613 | zimeldine | 10 µM | HL60 | 0 | 0.058 | 0.119 | 2012 |
| 2717 | 703 | santonin | 16 µM | PC3 | 0 | 0.058 | 0.072 | 4531 |
| 2718 | 708 | hymecromone | 23 µM | MCF7 | 0 | 0.058 | 0.111 | 5684 |
| 2719 | 750 | valproic acid | 50 µM | HL60 | 0 | 0.058 | 0.173 | 6181 |
| 2720 | 710 | famotidine | 12 µM | PC3 | 0 | 0.058 | 0.084 | 6665 |
| 2721 | 749 | benzylpenicillin | 11 µM | HL60 | 0 | 0.058 | 0.16 | 6155 |
| 2722 | 749 | dexpropranolol | 14 µM | HL60 | 0 | 0.058 | 0.19 | 6130 |
| 2723 | 642 | griseofulvin | 11 µM | MCF7 | 0 | 0.058 | 0.085 | 2293 |
| 2724 | 736 | scopolamine N-oxide | 10 µM | MCF7 | 0 | 0.057 | 0.071 | 5436 |
| 2725 | 657 | molsidomine | 17 µM | MCF7 | 0 | 0.057 | 0.068 | 2862 |
| 2726 | 757 | troglitazone | 10 µM | MCF7 | 0 | 0.057 | 0.057 | 5592 |
| 2727 | 682 | famprofazone | 11 µM | PC3 | 0 | 0.057 | 0.072 | 3753 |
| 2728 | 726 | phenoxybenzamine | 12 µM | MCF7 | 0 | 0.057 | 0.095 | 5248 |
| 2729 | 738 | lisinopril | 9 µM | MCF7 | 0 | 0.057 | 0.069 | 5504 |
| 2730 | 738 | bethanechol | 20 µM | MCF7 | 0 | 0.057 | 0.093 | 5539 |
| 2731 | 612 | isoxsuprine | 12 µM | HL60 | 0 | 0.057 | 0.107 | 1985 |
| 2732 | 650 | haloperidol | 10 µM | HL60 | 0 | 0.057 | 0.096 | 2663 |
| 2733 | 647 | sulfafurazole | 15 µM | MCF7 | 0 | 0.057 | 0.088 | 3218 |
| 2734 | 743 | ramipril | 10 µM | MCF7 | 0 | 0.057 | 0.075 | 6792 |
| 2735 | 710 | benzbromarone | 9 µM | PC3 | 0 | 0.057 | 0.092 | 6669 |
| 2736 | 761 | aminocaproic acid | 30 µM | PC3 | 0 | 0.057 | 0.073 | 7258 |
| 2737 | 738 | bupivacaine | 12 µM | MCF7 | 0 | 0.057 | 0.109 | 5537 |
| 2738 | 748 | azaperone | 12 µM | MCF7 | 0 | 0.057 | 0.132 | 7231 |
| 2739 | 664 | apramycin | 7 µM | HL60 | 0 | 0.057 | 0.205 | 2914 |
| 2740 | 720 | chlorcyclizine | 12 µM | MCF7 | 0 | 0.057 | 0.088 | 4367 |
| 2741 | 676 | Prestwick-967 | 26 µM | MCF7 | 0 | 0.057 | 0.066 | 7346 |
| 2742 | 1050 | 0175029-0000 | 10 µM | PC3 | 0 | 0.057 | 0.115 | 6875 |
| 2743 | 506 | tretinoin | 1 µM | MCF7 | 0 | 0.057 | 0.052 | 991 |
| 2744 | 767 | tanespimycin | 1 µM | MCF7 | 0 | 0.057 | 0.064 | 6926 |
| 2745 | 680 | clemizole | 11 µM | PC3 | 0 | 0.057 | 0.089 | 3672 |
| 2746 | 641 | dacarbazine | 22 µM | HL60 | 0 | 0.057 | 0.158 | 1762 |
| 2747 | 629 | allantoin | 25 µM | HL60 | 0 | 0.057 | 0.186 | 1842 |
| 2748 | 662 | papaverine | 11 µM | MCF7 | 0 | 0.057 | 0.097 | 2747 |
| 2749 | 614 | tetracycline | 8 µM | HL60 | 0 | 0.057 | 0.081 | 1397 |
| 2750 | 757 | tanespimycin | 1 µM | MCF7 | 0 | 0.057 | 0.076 | 5566 |
| 2751 | 622 | bambuterol | 10 µM | HL60 | 0 | 0.057 | 0.128 | 1582 |
| 2752 | 655 | serotonin | 19 µM | MCF7 | 0 | 0.057 | 0.069 | 3311 |
| 2753 | 758 | pentoxyverine | 8 µM | MCF7 | 0 | 0.057 | 0.054 | 5610 |
| 2754 | 725 | trichostatin A | 1 µM | MCF7 | 0 | 0.057 | 0.095 | 5231 |
| 2755 | 1040 | alprostadil | 10 µM | PC3 | 0 | 0.057 | 0.108 | 6571 |
| 2756 | 650 | chlorpromazine | 1 µM | HL60 | 0 | 0.057 | 0.11 | 2677 |
| 2757 | 772 | Prestwick-559 | 8 µM | MCF7 | 0 | 0.057 | 0.118 | 7462 |
| 2758 | 616 | acenocoumarol | 11 µM | PC3 | 0 | 0.057 | 0.034 | 2077 |
| 2759 | 1090 | CP-319743 | 10 µM | MCF7 | 0 | 0.057 | 0.113 | 7532 |
| 2760 | 746 | hexestrol | 15 µM | MCF7 | 0 | 0.057 | 0.084 | 6252 |
| 2761 | 700 | cloperastine | 11 µM | MCF7 | 0 | 0.057 | 0.139 | 4732 |
| 2762 | 693 | theophylline | 20 µM | PC3 | 0 | 0.057 | 0.069 | 4228 |
| 2763 | 694 | pyrimethamine | 16 µM | MCF7 | 0 | 0.057 | 0.058 | 4779 |
| 2764 | 750 | vorinostat | 10 µM | HL60 | 0 | 0.056 | 0.143 | 6179 |
| 2765 | 711 | ethisterone | 13 µM | MCF7 | 0 | 0.056 | 0.058 | 3975 |
| 2766 | 26b | sirolimus | 100 nM | MCF7 | 0 | 0.056 | 0.102 | 326 |
| 2767 | 687 | phenelzine | 17 µM | MCF7 | 0 | 0.056 | 0.075 | 3884 |
| 2768 | 708 | guanabenz | 14 µM | MCF7 | 0 | 0.056 | 0.052 | 5703 |
| 2769 | 719 | telenzepine | 9 µM | PC3 | 0 | 0.056 | 0.097 | 5096 |
| 2770 | 660 | fluspirilene | 8 µM | HL60 | 0 | 0.056 | 0.204 | 3086 |
| 2771 | 748 | neomycin | 4 µM | MCF7 | 0 | 0.056 | 0.069 | 7221 |
| 2772 | 634 | cisapride | 9 µM | HL60 | 0 | 0.056 | 0.098 | 2443 |
| 2773 | 656 | 6-azathymine | 31 µM | MCF7 | 0 | 0.056 | 0.081 | 2827 |
| 2774 | 688 | N-acetyl-L-aspartic acid | 23 µM | PC3 | 0 | 0.056 | 0.044 | 4007 |
| 2775 | 613 | chlortetracycline | 8 µM | HL60 | 0 | 0.056 | 0.068 | 2042 |
| 2776 | 626 | fulvestrant | 10 nM | MCF7 | 0 | 0.056 | 0.077 | 1663 |
| 2777 | 682 | deferoxamine | 6 µM | PC3 | 0 | 0.056 | 0.08 | 3760 |
| 2778 | 631 | brompheniramine | 9 µM | HL60 | 0 | 0.056 | 0.131 | 1335 |
| 2779 | 67 | pirinixic acid | 100 µM | MCF7 | 0 | 0.056 | 0.058 | 487 |
| 2780 | 1048 | SC-560 | 10 µM | PC3 | 0 | 0.056 | 0.079 | 6865 |
| 2781 | 720 | santonin | 16 µM | MCF7 | 0 | 0.056 | 0.105 | 4353 |
| 2782 | 706 | harpagoside | 8 µM | MCF7 | 0 | 0.056 | 0.065 | 4981 |
| 2783 | 715 | ergocalciferol | 10 µM | PC3 | 0 | 0.056 | 0.045 | 6746 |
| 2784 | 750 | haloperidol | 10 µM | HL60 | 0 | 0.056 | 0.164 | 6203 |
| 2785 | 514 | BW-B70C | 32 µM | MCF7 | 0 | 0.056 | 0.068 | 1132 |
| 2786 | 614 | tamoxifen | 7 µM | HL60 | 0 | 0.056 | 0.129 | 1366 |
| 2787 | 502 | monorden | 100 nM | MCF7 | 0 | 0.056 | 0.086 | 953 |
| 2788 | 119 | alpha-estradiol | 10 nM | MCF7 | 0 | 0.056 | 0.066 | 762 |
| 2789 | 635 | L-methionine sulfoximine | 22 µM | HL60 | 0 | 0.056 | 0.173 | 2470 |
| 2790 | 767 | acetylsalicylic acid | 100 µM | MCF7 | 0 | 0.056 | 0.086 | 6924 |
| 2791 | 738 | prednicarbate | 8 µM | MCF7 | 0 | 0.056 | 0.102 | 5544 |
| 2792 | 630 | buspirone | 9 µM | HL60 | 0 | 0.056 | 0.119 | 1282 |
| 2793 | 661 | esculetin | 22 µM | HL60 | 0 | 0.056 | 0.123 | 3120 |
| 2794 | 602 | tanespimycin | 1 µM | HL60 | 0 | 0.056 | 0.199 | 1147 |
| 2795 | 645 | Prestwick-665 | 12 µM | HL60 | 0 | 0.056 | 0.171 | 2186 |
| 2796 | 707 | doxazosin | 7 µM | MCF7 | 0 | 0.056 | 0.117 | 4988 |
| 2797 | 663 | corynanthine | 10 µM | MCF7 | 0 | 0.056 | 0.104 | 2786 |
| 2798 | 656 | norcyclobenzaprine | 15 µM | MCF7 | 0 | 0.056 | 0.062 | 2830 |
| 2799 | 709 | harpagoside | 8 µM | PC3 | 0 | 0.056 | 0.084 | 6636 |
| 2800 | 756 | aminocaproic acid | 30 µM | MCF7 | 0 | 0.056 | 0.085 | 6501 |
| 2801 | 672 | tolmetin | 13 µM | MCF7 | 0 | 0.056 | 0.08 | 3347 |
| 2802 | 762 | ozagrel | 15 µM | PC3 | 0 | 0.055 | 0.09 | 7281 |
| 2803 | 736 | pinacidil | 16 µM | MCF7 | 0 | 0.055 | 0.072 | 5456 |
| 2804 | 726 | testosterone | 12 µM | MCF7 | 0 | 0.055 | 0.08 | 5271 |
| 2805 | 701 | hydralazine | 20 µM | PC3 | 0 | 0.055 | 0.118 | 4282 |
| 2806 | 681 | benzamil | 11 µM | PC3 | 0 | 0.055 | 0.093 | 3738 |
| 2807 | 719 | mometasone | 8 µM | PC3 | 0 | 0.055 | 0.093 | 5116 |
| 2808 | 514 | trichostatin A | 100 nM | MCF7 | 0 | 0.055 | 0.123 | 1112 |
| 2809 | 613 | oleandomycin | 5 µM | HL60 | 0 | 0.055 | 0.106 | 2018 |
| 2810 | 1022 | C-75 | 10 µM | MCF7 | 0 | 0.055 | 0.085 | 6394 |
| 2811 | 2 | phenyl biguanide | 10 µM | MCF7 | 0 | 0.055 | 0.079 | 22 |
| 2812 | 1050 | trichostatin A | 1 µM | PC3 | 0 | 0.055 | 0.086 | 6874 |
| 2813 | 680 | nalidixic acid | 15 µM | PC3 | 0 | 0.055 | 0.049 | 3668 |
| 2814 | 761 | aceclofenac | 11 µM | PC3 | 0 | 0.055 | 0.074 | 7269 |
| 2815 | 744 | terfenadine | 8 µM | MCF7 | 0 | 0.055 | 0.08 | 6823 |
| 2816 | 62 | cobalt chloride | 100 µM | MCF7 | 0 | 0.055 | 0.065 | 454 |
| 2817 | 633 | danazol | 12 µM | MCF7 | 0 | 0.055 | 0.074 | 1538 |
| 2818 | 685 | cloperastine | 11 µM | MCF7 | 0 | 0.055 | 0.189 | 3608 |
| 2819 | 686 | trichostatin A | 100 nM | MCF7 | 0 | 0.055 | 0.079 | 3868 |
| 2820 | 622 | glibenclamide | 8 µM | HL60 | 0 | 0.055 | 0.074 | 1546 |
| 2821 | 698 | calcium folinate | 8 µM | PC3 | 0 | 0.055 | 0.068 | 7401 |
| 2822 | 767 | genistein | 10 µM | MCF7 | 0 | 0.055 | 0.075 | 6952 |
| 2823 | 502 | depudecin | 1 µM | MCF7 | 0 | 0.055 | 0.099 | 982 |
| 2824 | 642 | tropicamide | 14 µM | MCF7 | 0 | 0.055 | 0.088 | 2309 |
| 2825 | 695 | corynanthine | 10 µM | MCF7 | 0 | 0.055 | 0.049 | 4811 |
| 2826 | 631 | cefotetan | 7 µM | HL60 | 0 | 0.055 | 0.127 | 1319 |
| 2827 | 685 | PNU-0230031 | 10 µM | MCF7 | 0 | 0.055 | 0.048 | 3629 |
| 2828 | 741 | iobenguane | 11 µM | MCF7 | 0 | 0.054 | 0.064 | 6002 |
| 2829 | 765 | alpha-estradiol | 10 nM | MCF7 | 0 | 0.054 | 0.079 | 6970 |
| 2830 | 767 | trichostatin A | 1 µM | MCF7 | 0 | 0.054 | 0.134 | 6951 |
| 2831 | 655 | hexetidine | 12 µM | MCF7 | 0 | 0.054 | 0.066 | 3318 |
| 2832 | 730 | digoxin | 5 µM | MCF7 | 0 | 0.054 | 0.078 | 5324 |
| 2833 | 502 | 5279552 | 22 µM | MCF7 | 0 | 0.054 | 0.075 | 960 |
| 2834 | 734 | pirenzepine | 9 µM | PC3 | 0 | 0.054 | 0.075 | 5872 |
| 2835 | 618 | ethisterone | 13 µM | HL60 | 0 | 0.054 | 0.148 | 2366 |
| 2836 | 660 | flurbiprofen | 16 µM | HL60 | 0 | 0.054 | 0.097 | 3095 |
| 2837 | 637 | xylometazoline | 14 µM | MCF7 | 0 | 0.054 | 0.091 | 2270 |
| 2838 | 738 | sulindac | 11 µM | MCF7 | 0 | 0.054 | 0.063 | 5528 |
| 2839 | 657 | sulfasalazine | 10 µM | MCF7 | 0 | 0.054 | 0.083 | 2882 |
| 2840 | 633 | oleandomycin | 5 µM | MCF7 | 0 | 0.054 | 0.121 | 1518 |
| 2841 | 613 | edrophonium chloride | 20 µM | HL60 | 0 | 0.054 | 0.108 | 2019 |
| 2842 | 701 | prilocaine | 16 µM | PC3 | 0 | 0.054 | 0.116 | 4284 |
| 2843 | 653 | 3-hydroxy-DL-kynurenine | 18 µM | MCF7 | 0 | 0.054 | 0.068 | 2654 |
| 2844 | 707 | glipizide | 9 µM | MCF7 | 0 | 0.054 | 0.088 | 4991 |
| 2845 | 504 | tanespimycin | 1 µM | MCF7 | 0 | 0.054 | 0.042 | 831 |
| 2846 | 682 | lanatoside C | 4 µM | PC3 | 0 | 0.054 | 0.069 | 3771 |
| 2847 | 671 | clidinium bromide | 9 µM | MCF7 | 0 | 0.054 | 0.086 | 3476 |
| 2848 | 602 | valproic acid | 1 mM | HL60 | 0 | 0.054 | 0.141 | 1150 |
| 2849 | 626 | thioridazine | 10 µM | MCF7 | 0 | 0.054 | 0.112 | 1655 |
| 2850 | 709 | hexamethonium bromide | 10 µM | PC3 | 0 | 0.054 | 0.102 | 6620 |
| 2851 | 694 | quinisocaine | 13 µM | MCF7 | 0 | 0.054 | 0.076 | 4791 |
| 2852 | 772 | 8-azaguanine | 26 µM | MCF7 | 0 | 0.054 | 0.084 | 7444 |
| 2853 | 701 | demeclocycline | 8 µM | PC3 | 0 | 0.054 | 0.082 | 4267 |
| 2854 | 706 | miconazole | 10 µM | MCF7 | 0 | 0.054 | 0.098 | 4960 |
| 2855 | 756 | remoxipride | 10 µM | MCF7 | 0 | 0.054 | 0.073 | 6503 |
| 2856 | 660 | nimodipine | 10 µM | HL60 | 0 | 0.054 | 0.153 | 3103 |
| 2857 | 1002 | PF-00562151-00 | 10 µM | MCF7 | 0 | 0.054 | 0.062 | 5917 |
| 2858 | 672 | reserpine | 7 µM | MCF7 | 0 | 0.054 | 0.119 | 3341 |
| 2859 | 711 | famprofazone | 11 µM | MCF7 | 0 | 0.054 | 0.1 | 3928 |
| 2860 | 1083 | trichostatin A | 1 µM | PC3 | 0 | 0.053 | 0.119 | 7503 |
| 2861 | 653 | procainamide | 15 µM | MCF7 | 0 | 0.053 | 0.098 | 2618 |
| 2862 | 709 | dl-alpha tocopherol | 9 µM | PC3 | 0 | 0.053 | 0.063 | 6616 |
| 2863 | 762 | tranylcypromine | 24 µM | PC3 | 0 | 0.053 | 0.062 | 7293 |
| 2864 | 661 | denatonium benzoate | 9 µM | HL60 | 0 | 0.053 | 0.126 | 3123 |
| 2865 | 751 | acacetin | 14 µM | MCF7 | 0 | 0.053 | 0.1 | 6044 |
| 2866 | 732 | propidium iodide | 6 µM | PC3 | 0 | 0.053 | 0.069 | 5803 |
| 2867 | 505 | celecoxib | 10 µM | MCF7 | 0 | 0.053 | 0.062 | 922 |
| 2868 | 694 | tolnaftate | 13 µM | MCF7 | 0 | 0.053 | 0.07 | 4805 |
| 2869 | 710 | clorgiline | 13 µM | PC3 | 0 | 0.053 | 0.09 | 6659 |
| 2870 | 745 | cicloheximide | 14 µM | MCF7 | 0 | 0.053 | 0.097 | 6220 |
| 2871 | 616 | thioproperazine | 6 µM | PC3 | 0 | 0.053 | 0.049 | 2073 |
| 2872 | 746 | trichostatin A | 100 nM | MCF7 | 0 | 0.053 | 0.08 | 6276 |
| 2873 | 502 | geldanamycin | 1 µM | MCF7 | 0 | 0.053 | 0.075 | 972 |
| 2874 | 658 | simvastatin | 10 µM | HL60 | 0 | 0.053 | 0.065 | 3002 |
| 2875 | 617 | piroxicam | 12 µM | PC3 | 0 | 0.053 | 0.054 | 2089 |
| 2876 | 20 | MK-886 | 1 µM | MCF7 | 0 | 0.053 | 0.068 | 264 |
| 2877 | 645 | eldeline | 8 µM | HL60 | 0 | 0.053 | 0.144 | 2171 |
| 2878 | 614 | diazoxide | 17 µM | HL60 | 0 | 0.053 | 0.075 | 1368 |
| 2879 | 680 | trimethoprim | 14 µM | PC3 | 0 | 0.053 | 0.093 | 3678 |
| 2880 | 611 | dehydrocholic acid | 10 µM | PC3 | 0 | 0.053 | 0.053 | 1940 |
| 2881 | 657 | Prestwick-559 | 8 µM | MCF7 | 0 | 0.053 | 0.095 | 2877 |
| 2882 | 732 | butamben | 21 µM | PC3 | 0 | 0.053 | 0.105 | 5792 |
| 2883 | 628 | sulfaphenazole | 13 µM | PC3 | 0 | 0.053 | 0.056 | 1794 |
| 2884 | 1048 | STOCK1N-28457 | 20 µM | PC3 | 0 | 0.053 | 0.117 | 6864 |
| 2885 | 708 | iohexol | 5 µM | MCF7 | 0 | 0.053 | 0.058 | 5704 |
| 2886 | 664 | pancuronium bromide | 5 µM | HL60 | 0 | 0.053 | 0.198 | 2909 |
| 2887 | 26b | monorden | 100 nM | MCF7 | 0 | 0.053 | 0.059 | 325 |
| 2888 | 748 | convolamine | 12 µM | MCF7 | 0 | 0.053 | 0.092 | 7230 |
| 2889 | 1084 | daunorubicin | 1 µM | MCF7 | 0 | 0.053 | 0.105 | 7507 |
| 2890 | 663 | picrotoxinin | 14 µM | MCF7 | 0 | 0.053 | 0.079 | 2816 |
| 2891 | 658 | propantheline bromide | 9 µM | HL60 | 0 | 0.053 | 0.154 | 3013 |
| 2892 | 752 | podophyllotoxin | 10 µM | MCF7 | 0 | 0.053 | 0.055 | 6103 |
| 2893 | 642 | metaraminol | 9 µM | MCF7 | 0 | 0.052 | 0.063 | 2298 |
| 2894 | 689 | cimetidine | 16 µM | PC3 | 0 | 0.052 | 0.087 | 4063 |
| 2895 | 506 | tanespimycin | 1 µM | MCF7 | 0 | 0.052 | 0.056 | 1005 |
| 2896 | 741 | nortriptyline | 13 µM | MCF7 | 0 | 0.052 | 0.12 | 6003 |
| 2897 | 689 | levothyroxine sodium | 5 µM | PC3 | 0 | 0.052 | 0.075 | 4069 |
| 2898 | 754 | oxymetazoline | 13 µM | PC3 | 0 | 0.052 | 0.087 | 6350 |
| 2899 | 767 | fluphenazine | 10 µM | MCF7 | 0 | 0.052 | 0.075 | 6954 |
| 2900 | 712 | bromocriptine | 5 µM | PC3 | 0 | 0.052 | 0.092 | 4604 |
| 2901 | 623 | methyldopa | 19 µM | HL60 | 0 | 0.052 | 0.16 | 1619 |
| 2902 | 513 | alvespimycin | 100 nM | MCF7 | 0 | 0.052 | 0.061 | 1051 |
| 2903 | 614 | neomycin | 4 µM | HL60 | 0 | 0.052 | 0.09 | 1383 |
| 2904 | 708 | tonzonium bromide | 7 µM | MCF7 | 0 | 0.052 | 0.086 | 5678 |
| 2905 | 1094 | trichostatin A | 1 µM | MCF7 | 0 | 0.052 | 0.101 | 7550 |
| 2906 | 1090 | BCB000039 | 10 µM | MCF7 | 0 | 0.052 | 0.093 | 7531 |
| 2907 | 641 | ellipticine | 16 µM | HL60 | 0 | 0.052 | 0.121 | 1765 |
| 2908 | 713 | phthalylsulfathiazole | 10 µM | PC3 | 0 | 0.052 | 0.082 | 4653 |
| 2909 | 726 | equilin | 15 µM | MCF7 | 0 | 0.052 | 0.082 | 5255 |
| 2910 | 705 | proscillaridin | 8 µM | MCF7 | 0 | 0.052 | 0.126 | 4404 |
| 2911 | 756 | ketorolac | 11 µM | MCF7 | 0 | 0.052 | 0.068 | 6489 |
| 2912 | 758 | bephenium hydroxynaphthoate | 9 µM | MCF7 | 0 | 0.052 | 0.1 | 5628 |
| 2913 | 58 | novobiocin | 100 µM | MCF7 | 0 | 0.052 | 0.061 | 437 |
| 2914 | 614 | picotamide | 10 µM | HL60 | 0 | 0.052 | 0.112 | 1387 |
| 2915 | 736 | dimenhydrinate | 9 µM | MCF7 | 0 | 0.052 | 0.088 | 5450 |
| 2916 | 751 | sulfachlorpyridazine | 14 µM | MCF7 | 0 | 0.052 | 0.083 | 6046 |
| 2917 | 686 | eucatropine | 12 µM | MCF7 | 0 | 0.052 | 0.058 | 3841 |
| 2918 | 1051 | BAS-012416453 | 38 µM | MCF7 | 0 | 0.052 | 0.11 | 6880 |
| 2919 | 748 | gabapentin | 23 µM | MCF7 | 0 | 0.052 | 0.087 | 7229 |
| 2920 | 711 | mexiletine | 19 µM | MCF7 | 0 | 0.052 | 0.074 | 3973 |
| 2921 | 657 | cyclizine | 13 µM | MCF7 | 0 | 0.052 | 0.106 | 2880 |
| 2922 | 692 | reserpine | 7 µM | PC3 | 0 | 0.052 | 0.041 | 4203 |
| 2923 | 741 | proxyphylline | 17 µM | MCF7 | 0 | 0.052 | 0.074 | 5993 |
| 2924 | 504 | 5279552 | 22 µM | MCF7 | 0 | 0.052 | 0.086 | 843 |
| 2925 | 690 | niclosamide | 12 µM | MCF7 | 0 | 0.052 | 0.067 | 4136 |
| 2926 | 629 | triamterene | 16 µM | HL60 | 0 | 0.052 | 0.145 | 1861 |
| 2927 | 701 | tropicamide | 14 µM | PC3 | 0 | 0.052 | 0.136 | 4280 |
| 2928 | 751 | Prestwick-685 | 11 µM | MCF7 | 0 | 0.052 | 0.076 | 6043 |
| 2929 | 629 | 8-azaguanine | 26 µM | HL60 | 0 | 0.052 | 0.191 | 1833 |
| 2930 | 711 | eucatropine | 12 µM | MCF7 | 0 | 0.051 | 0.114 | 3935 |
| 2931 | 685 | 0179445-0000 | 10 µM | MCF7 | 0 | 0.051 | 0.155 | 3630 |
| 2932 | 771 | trifluoperazine | 8 µM | MCF7 | 0 | 0.051 | 0.105 | 7420 |
| 2933 | 673 | diclofenamide | 13 µM | MCF7 | 0 | 0.051 | 0.148 | 3366 |
| 2934 | 63 | tetraethylenepentamine | 100 µM | PC3 | 0 | 0.051 | 0.061 | 457 |
| 2935 | 41 | tretinoin | 1 µM | HL60 | 0 | 0.051 | 0.081 | 390 |
| 2936 | 637 | fenspiride | 13 µM | MCF7 | 0 | 0.051 | 0.041 | 2269 |
| 2937 | 670 | guanadrel | 8 µM | MCF7 | 0 | 0.051 | 0.121 | 3438 |
| 2938 | 651 | doxycycline | 8 µM | HL60 | 0 | 0.051 | 0.123 | 2737 |
| 2939 | 736 | procarbazine | 16 µM | MCF7 | 0 | 0.051 | 0.083 | 5452 |
| 2940 | 748 | oxprenolol | 13 µM | MCF7 | 0 | 0.051 | 0.103 | 7225 |
| 2941 | 623 | methylergometrine | 9 µM | HL60 | 0 | 0.051 | 0.121 | 1607 |
| 2942 | 735 | dequalinium chloride | 8 µM | MCF7 | 0 | 0.051 | 0.065 | 5396 |
| 2943 | 749 | repaglinide | 9 µM | HL60 | 0 | 0.051 | 0.174 | 6135 |
| 2944 | 680 | sulfadimethoxine | 13 µM | PC3 | 0 | 0.051 | 0.077 | 3702 |
| 2945 | 706 | dl-alpha tocopherol | 9 µM | MCF7 | 0 | 0.051 | 0.064 | 4961 |
| 2946 | 701 | Prestwick-664 | 8 µM | PC3 | 0 | 0.051 | 0.08 | 4275 |
| 2947 | 633 | edrophonium chloride | 20 µM | MCF7 | 0 | 0.051 | 0.075 | 1519 |
| 2948 | 734 | mesalazine | 26 µM | PC3 | 0 | 0.051 | 0.067 | 5888 |
| 2949 | 53 | LY-294002 | 100 nM | MCF7 | 0 | 0.051 | 0.051 | 424 |
| 2950 | 703 | oxaprozin | 14 µM | PC3 | 0 | 0.051 | 0.06 | 4530 |
| 2951 | 649 | ethoxyquin | 18 µM | HL60 | 0 | 0.051 | 0.16 | 2559 |
| 2952 | 745 | clotrimazole | 12 µM | MCF7 | 0 | 0.051 | 0.092 | 6207 |
| 2953 | 719 | oxolinic acid | 15 µM | PC3 | 0 | 0.05 | 0.068 | 5094 |
| 2954 | 729 | propofol | 22 µM | MCF7 | 0 | 0.05 | 0.08 | 5306 |
| 2955 | 736 | tribenoside | 8 µM | MCF7 | 0 | 0.05 | 0.075 | 5429 |
| 2956 | 1090 | fulvestrant | 1 µM | MCF7 | 0 | 0.05 | 0.108 | 7534 |
| 2957 | 735 | cefoperazone | 6 µM | MCF7 | 0 | 0.05 | 0.079 | 5424 |
| 2958 | 695 | buflomedil | 12 µM | MCF7 | 0 | 0.05 | 0.068 | 4840 |
| 2959 | 1085 | daunorubicin | 1 µM | PC3 | 0 | 0.05 | 0.071 | 7511 |
| 2960 | 758 | testosterone | 12 µM | MCF7 | 0 | 0.05 | 0.08 | 5636 |
| 2961 | 16 | rofecoxib | 10 µM | MCF7 | 0 | 0.05 | 0.05 | 205 |
| 2962 | 746 | nicergoline | 8 µM | MCF7 | 0 | 0.05 | 0.065 | 6251 |
| 2963 | 702 | pempidine | 13 µM | PC3 | 0 | 0.05 | 0.066 | 4307 |
| 2964 | 745 | tetrahydroalstonine | 11 µM | MCF7 | 0 | 0.05 | 0.052 | 6209 |
| 2965 | 657 | thiocolchicoside | 7 µM | MCF7 | 0 | 0.05 | 0.127 | 2875 |
| 2966 | 709 | palmatine | 10 µM | PC3 | 0 | 0.05 | 0.09 | 6612 |
| 2967 | 16 | celecoxib | 10 µM | MCF7 | 0 | 0.05 | 0.065 | 206 |
| 2968 | 642 | mephenesin | 22 µM | MCF7 | 0 | 0.05 | 0.095 | 2304 |
| 2969 | 640 | gliclazide | 12 µM | HL60 | 0 | 0.05 | 0.111 | 1720 |
| 2970 | 506 | alpha-estradiol | 10 nM | MCF7 | 0 | 0.05 | 0.053 | 990 |
| 2971 | 694 | pregnenolone | 13 µM | MCF7 | 0 | 0.05 | 0.067 | 4802 |
| 2972 | 63 | valproic acid | 1 mM | PC3 | 0 | 0.05 | 0.096 | 458 |
| 2973 | 1026 | orlistat | 10 µM | MCF7 | 0 | 0.05 | 0.117 | 6415 |
| 2974 | 751 | Prestwick-665 | 12 µM | MCF7 | 0 | 0.05 | 0.096 | 6041 |
| 2975 | 700 | trichostatin A | 100 nM | MCF7 | 0 | 0.049 | 0.116 | 4768 |
| 2976 | 711 | deferoxamine | 6 µM | MCF7 | 0 | 0.049 | 0.08 | 3936 |
| 2977 | 653 | hesperidin | 7 µM | MCF7 | 0 | 0.049 | 0.109 | 2648 |
| 2978 | 718 | roxarsone | 15 µM | PC3 | 0 | 0.049 | 0.067 | 5051 |
| 2979 | 767 | fulvestrant | 10 nM | MCF7 | 0 | 0.049 | 0.097 | 6955 |
| 2980 | 764 | methylprednisolone | 11 µM | PC3 | 0 | 0.049 | 0.067 | 7137 |
| 2981 | 699 | mebendazole | 14 µM | MCF7 | 0 | 0.049 | 0.125 | 4694 |
| 2982 | 695 | SR-95531 | 11 µM | MCF7 | 0 | 0.049 | 0.054 | 4820 |
| 2983 | 703 | diethylstilbestrol | 15 µM | PC3 | 0 | 0.049 | 0.047 | 4547 |
| 2984 | 1081 | CP-319743 | 10 µM | PC3 | 0 | 0.049 | 0.085 | 7491 |
| 2985 | 43 | tetraethylenepentamine | 10 µM | MCF7 | 0 | 0.049 | 0.074 | 405 |
| 2986 | 700 | fenbufen | 16 µM | MCF7 | 0 | 0.049 | 0.089 | 4743 |
| 2987 | 506 | fulvestrant | 1 µM | MCF7 | 0 | 0.049 | 0.118 | 985 |
| 2988 | 45 | trichostatin A | 100 nM | ssMCF7 | 0 | 0.049 | 0.092 | 413 |
| 2989 | 615 | ornidazole | 18 µM | HL60 | 0 | 0.049 | 0.087 | 1425 |
| 2990 | 702 | solasodine | 10 µM | PC3 | 0 | 0.049 | 0.066 | 4305 |
| 2991 | 685 | fenbufen | 16 µM | MCF7 | 0 | 0.049 | 0.1 | 3618 |
| 2992 | 719 | flumequine | 15 µM | PC3 | 0 | 0.049 | 0.068 | 5104 |
| 2993 | 715 | fusidic acid | 7 µM | PC3 | 0 | 0.049 | 0.082 | 6754 |
| 2994 | 661 | ketorolac | 11 µM | HL60 | 0 | 0.048 | 0.106 | 3110 |
| 2995 | 504 | 5255229 | 13 µM | MCF7 | 0 | 0.048 | 0.085 | 833 |
| 2996 | 752 | tetryzoline | 17 µM | MCF7 | 0 | 0.048 | 0.085 | 6069 |
| 2997 | 718 | tridihexethyl | 11 µM | PC3 | 0 | 0.048 | 0.098 | 5067 |
| 2998 | 1010 | PF-00562151-00 | 10 µM | MCF7 | 0 | 0.048 | 0.064 | 5954 |
| 2999 | 758 | digoxigenin | 10 µM | MCF7 | 0 | 0.048 | 0.124 | 5640 |
| 3000 | 692 | digitoxigenin | 11 µM | PC3 | 0 | 0.048 | 0.081 | 4217 |
| 3001 | 617 | mafenide | 18 µM | PC3 | 0 | 0.048 | 0.051 | 2124 |
| 3002 | 1077 | AR-A014418 | 10 µM | PC3 | 0 | 0.048 | 0.078 | 7097 |
| 3003 | 1086 | BCB000038 | 10 µM | MCF7 | 0 | 0.048 | 0.06 | 7516 |
| 3004 | 1034 | alprostadil | 10 µM | PC3 | 0 | 0.048 | 0.061 | 6555 |
| 3005 | 710 | clomifene | 7 µM | PC3 | 0 | 0.048 | 0.078 | 6648 |
| 3006 | 8 | sulindac | 100 µM | MCF7 | 0 | 0.048 | 0.061 | 168 |
| 3007 | 732 | thioproperazine | 6 µM | PC3 | 0 | 0.048 | 0.059 | 5791 |
| 3008 | 762 | nifenazone | 13 µM | PC3 | 0 | 0.048 | 0.066 | 7314 |
| 3009 | 730 | oxantel | 7 µM | MCF7 | 0 | 0.048 | 0.102 | 5338 |
| 3010 | 1084 | pioglitazone | 10 µM | MCF7 | 0 | 0.048 | 0.088 | 7506 |
| 3011 | 613 | moroxydine | 19 µM | HL60 | 0 | 0.047 | 0.135 | 2027 |
| 3012 | 726 | dipyridamole | 8 µM | MCF7 | 0 | 0.047 | 0.11 | 5252 |
| 3013 | 753 | azacyclonol | 15 µM | PC3 | 0 | 0.047 | 0.078 | 6298 |
| 3014 | 693 | cetirizine | 9 µM | PC3 | 0 | 0.047 | 0.073 | 4231 |
| 3015 | 709 | nifurtimox | 14 µM | PC3 | 0 | 0.047 | 0.053 | 6608 |
| 3016 | 757 | LY-294002 | 10 µM | MCF7 | 0 | 0.047 | 0.055 | 5596 |
| 3017 | 744 | napelline | 11 µM | MCF7 | 0 | 0.047 | 0.053 | 6824 |
| 3018 | 646 | fluphenazine | 8 µM | MCF7 | 0 | 0.047 | 0.09 | 3194 |
| 3019 | 755 | phenoxybenzamine | 12 µM | MCF7 | 0 | 0.047 | 0.083 | 6451 |
| 3020 | 622 | terbutaline | 7 µM | HL60 | 0 | 0.047 | 0.134 | 1585 |
| 3021 | 712 | ketoconazole | 8 µM | PC3 | 0 | 0.047 | 0.064 | 4624 |
| 3022 | 702 | trichostatin A | 100 nM | PC3 | 0 | 0.047 | 0.12 | 4344 |
| 3023 | 692 | pregnenolone | 13 µM | PC3 | 0 | 0.047 | 0.074 | 4218 |
| 3024 | 765 | tanespimycin | 1 µM | MCF7 | 0 | 0.047 | 0.071 | 6966 |
| 3025 | 622 | antazoline | 13 µM | HL60 | 0 | 0.047 | 0.095 | 1556 |
| 3026 | 686 | helveticoside | 7 µM | MCF7 | 0 | 0.047 | 0.073 | 3851 |
| 3027 | 678 | mephenytoin | 18 µM | MCF7 | 0 | 0.047 | 0.106 | 3580 |
| 3028 | 688 | boldine | 12 µM | PC3 | 0 | 0.047 | 0.058 | 4004 |
| 3029 | 713 | clozapine | 12 µM | PC3 | 0 | 0.047 | 0.077 | 4670 |
| 3030 | 630 | hesperidin | 7 µM | HL60 | 0 | 0.047 | 0.113 | 1294 |
| 3031 | 614 | pirenzepine | 9 µM | HL60 | 0 | 0.047 | 0.103 | 1388 |
| 3032 | 670 | dicoumarol | 12 µM | MCF7 | 0 | 0.047 | 0.074 | 3423 |
| 3033 | 603 | LY-294002 | 100 nM | PC3 | 0 | 0.047 | 0.041 | 1216 |
| 3034 | 663 | vitexin | 9 µM | MCF7 | 0 | 0.047 | 0.075 | 2810 |
| 3035 | 758 | clioquinol | 13 µM | MCF7 | 0 | 0.047 | 0.119 | 5623 |
| 3036 | 629 | todralazine | 15 µM | HL60 | 0 | 0.047 | 0.206 | 1841 |
| 3037 | 730 | lynestrenol | 14 µM | MCF7 | 0 | 0.047 | 0.097 | 5355 |
| 3038 | 602 | sirolimus | 100 nM | HL60 | 0 | 0.047 | 0.184 | 1148 |
| 3039 | 711 | furaltadone | 11 µM | MCF7 | 0 | 0.047 | 0.071 | 3932 |
| 3040 | 671 | proadifen | 10 µM | MCF7 | 0 | 0.046 | 0.071 | 3446 |
| 3041 | 691 | tetroquinone | 21 µM | MCF7 | 0 | 0.046 | 0.078 | 4159 |
| 3042 | 708 | zimeldine | 10 µM | MCF7 | 0 | 0.046 | 0.105 | 5670 |
| 3043 | 725 | sirolimus | 100 nM | MCF7 | 0 | 0.046 | 0.091 | 5204 |
| 3044 | 53 | staurosporine | 100 nM | MCF7 | 0 | 0.046 | 0.071 | 423 |
| 3045 | 694 | zardaverine | 15 µM | MCF7 | 0 | 0.046 | 0.062 | 4793 |
| 3046 | 603 | wortmannin | 10 nM | PC3 | 0 | 0.046 | 0.083 | 1243 |
| 3047 | 663 | solanine | 5 µM | MCF7 | 0 | 0.046 | 0.104 | 2808 |
| 3048 | 662 | (+)-chelidonine | 11 µM | MCF7 | 0 | 0.046 | 0.102 | 2779 |
| 3049 | 1040 | 5155877 | 10 µM | PC3 | 0 | 0.046 | 0.092 | 6569 |
| 3050 | 689 | ticlopidine | 13 µM | PC3 | 0 | 0.046 | 0.088 | 4074 |
| 3051 | 690 | suprofen | 15 µM | MCF7 | 0 | 0.046 | 0.083 | 4123 |
| 3052 | 615 | trichostatin A | 100 nM | HL60 | 0 | 0.046 | 0.159 | 1421 |
| 3053 | 613 | perphenazine | 10 µM | HL60 | 0 | 0.046 | 0.125 | 2040 |
| 3054 | 741 | phenindione | 18 µM | MCF7 | 0 | 0.046 | 0.068 | 5991 |
| 3055 | 693 | sulfaguanidine | 19 µM | PC3 | 0 | 0.046 | 0.085 | 4257 |
| 3056 | 719 | gliclazide | 12 µM | PC3 | 0 | 0.046 | 0.067 | 5089 |
| 3057 | 709 | pyrazinamide | 32 µM | PC3 | 0 | 0.046 | 0.067 | 6617 |
| 3058 | 715 | metergoline | 10 µM | PC3 | 0 | 0.046 | 0.088 | 6744 |
| 3059 | 1004 | PF-00875133-00 | 10 µM | MCF7 | 0 | 0.046 | 0.049 | 5923 |
| 3060 | 683 | homosalate | 15 µM | PC3 | 0 | 0.045 | 0.135 | 3797 |
| 3061 | 735 | ascorbic acid | 22 µM | MCF7 | 0 | 0.045 | 0.077 | 5407 |
| 3062 | 615 | gemfibrozil | 16 µM | HL60 | 0 | 0.045 | 0.103 | 1430 |
| 3063 | 680 | riluzole | 15 µM | PC3 | 0 | 0.045 | 0.07 | 3666 |
| 3064 | 615 | norfloxacin | 13 µM | HL60 | 0 | 0.045 | 0.143 | 1406 |
| 3065 | 734 | ivermectin | 5 µM | PC3 | 0 | 0.045 | 0.093 | 5853 |
| 3066 | 616 | chlorphenamine | 10 µM | PC3 | 0 | 0.045 | 0.047 | 2055 |
| 3067 | 694 | molindone | 13 µM | MCF7 | 0 | 0.045 | 0.064 | 4784 |
| 3068 | 765 | trifluoperazine | 10 µM | MCF7 | 0 | 0.045 | 0.054 | 6984 |
| 3069 | 702 | indapamide | 11 µM | PC3 | 0 | 0.045 | 0.074 | 4335 |
| 3070 | 714 | hydrocortisone | 11 µM | PC3 | 0 | 0.045 | 0.072 | 6684 |
| 3071 | 748 | bergenin | 12 µM | MCF7 | 0 | 0.045 | 0.115 | 7224 |
| 3072 | 756 | trapidil | 19 µM | MCF7 | 0 | 0.045 | 0.059 | 6515 |
| 3073 | 752 | propidium iodide | 6 µM | MCF7 | 0 | 0.045 | 0.053 | 6104 |
| 3074 | 647 | phenylpropanolamine | 21 µM | MCF7 | 0 | 0.045 | 0.061 | 3217 |
| 3075 | 704 | tiabendazole | 20 µM | PC3 | 0 | 0.045 | 0.056 | 4579 |
| 3076 | 1080 | CP-319743 | 10 µM | MCF7 | 0 | 0.045 | 0.095 | 7486 |
| 3077 | 733 | proadifen | 10 µM | PC3 | 0 | 0.045 | 0.079 | 5807 |
| 3078 | 753 | amoxicillin | 11 µM | PC3 | 0 | 0.045 | 0.063 | 6285 |
| 3079 | 718 | minocycline | 8 µM | PC3 | 0 | 0.045 | 0.102 | 5077 |
| 3080 | 68 | dopamine | 1 µM | MCF7 | 0 | 0.045 | 0.119 | 491 |
| 3081 | 68 | monorden | 100 nM | MCF7 | 0 | 0.045 | 0.084 | 489 |
| 3082 | 690 | pronetalol | 15 µM | MCF7 | 0 | 0.045 | 0.102 | 4104 |
| 3083 | 719 | rilmenidine | 8 µM | PC3 | 0 | 0.044 | 0.058 | 5107 |
| 3084 | 663 | sulfabenzamide | 14 µM | MCF7 | 0 | 0.044 | 0.078 | 2814 |
| 3085 | 1052 | AH-23848 | 1 µM | PC3 | 0 | 0.044 | 0.061 | 6885 |
| 3086 | 687 | CP-690334-01 | 10 µM | MCF7 | 0 | 0.044 | 0.044 | 3906 |
| 3087 | 707 | clorgiline | 13 µM | MCF7 | 0 | 0.044 | 0.103 | 5005 |
| 3088 | 736 | isradipine | 11 µM | MCF7 | 0 | 0.044 | 0.075 | 5447 |
| 3089 | 733 | natamycin | 6 µM | PC3 | 0 | 0.044 | 0.059 | 5809 |
| 3090 | 623 | ascorbic acid | 22 µM | HL60 | 0 | 0.044 | 0.118 | 1610 |
| 3091 | 657 | dosulepin | 12 µM | MCF7 | 0 | 0.044 | 0.084 | 2864 |
| 3092 | 657 | parthenolide | 16 µM | MCF7 | 0 | 0.044 | 0.08 | 2885 |
| 3093 | 613 | josamycin | 5 µM | HL60 | 0 | 0.044 | 0.114 | 2034 |
| 3094 | 701 | metoclopramide | 12 µM | PC3 | 0 | 0.044 | 0.12 | 4285 |
| 3095 | 713 | butyl hydroxybenzoate | 21 µM | PC3 | 0 | 0.044 | 0.045 | 4647 |
| 3096 | 25 | acetylsalicylic acid | 100 µM | MCF7 | 0 | 0.044 | 0.106 | 315 |
| 3097 | 647 | betaxolol | 12 µM | MCF7 | 0 | 0.044 | 0.095 | 3208 |
| 3098 | 761 | triprolidine | 13 µM | PC3 | 0 | 0.044 | 0.077 | 7248 |
| 3099 | 616 | cefotaxime | 8 µM | PC3 | 0 | 0.044 | 0.076 | 2072 |
| 3100 | 727 | chlorpromazine | 1 µM | PC3 | 0 | 0.044 | 0.076 | 4441 |
| 3101 | 676 | Prestwick-984 | 9 µM | MCF7 | 0 | 0.044 | 0.06 | 7323 |
| 3102 | 761 | Prestwick-972 | 23 µM | PC3 | 0 | 0.044 | 0.069 | 7266 |
| 3103 | 707 | clomifene | 7 µM | MCF7 | 0 | 0.044 | 0.117 | 4994 |
| 3104 | 714 | estradiol | 15 µM | PC3 | 0 | 0.044 | 0.041 | 6718 |
| 3105 | 614 | spironolactone | 10 µM | HL60 | 0 | 0.044 | 0.097 | 1380 |
| 3106 | 616 | tranexamic acid | 25 µM | PC3 | 0 | 0.043 | 0.06 | 2085 |
| 3107 | 734 | isoconazole | 10 µM | PC3 | 0 | 0.043 | 0.101 | 5857 |
| 3108 | 732 | quinidine | 11 µM | PC3 | 0 | 0.043 | 0.049 | 5793 |
| 3109 | 700 | 6-benzylaminopurine | 18 µM | MCF7 | 0 | 0.043 | 0.115 | 4748 |
| 3110 | 751 | pempidine | 13 µM | MCF7 | 0 | 0.043 | 0.115 | 6027 |
| 3111 | 756 | trichostatin A | 100 nM | MCF7 | 0 | 0.043 | 0.09 | 6493 |
| 3112 | 661 | scopoletin | 21 µM | HL60 | 0 | 0.043 | 0.148 | 3131 |
| 3113 | 752 | anabasine | 25 µM | MCF7 | 0 | 0.043 | 0.087 | 6074 |
| 3114 | 710 | bemegride | 26 µM | PC3 | 0 | 0.043 | 0.093 | 6668 |
| 3115 | 750 | sirolimus | 100 nM | HL60 | 0 | 0.042 | 0.165 | 6201 |
| 3116 | 718 | mafenide | 18 µM | PC3 | 0 | 0.042 | 0.053 | 5079 |
| 3117 | 687 | ethotoin | 20 µM | MCF7 | 0 | 0.042 | 0.088 | 3892 |
| 3118 | 685 | Prestwick-664 | 8 µM | MCF7 | 0 | 0.042 | 0.11 | 3613 |
| 3119 | 44 | valproic acid | 10 mM | HL60 | 0 | 0.042 | 0.082 | 410 |
| 3120 | 690 | 6-azathymine | 31 µM | MCF7 | 0 | 0.042 | 0.09 | 4106 |
| 3121 | 704 | N-acetylmuramic acid | 14 µM | PC3 | 0 | 0.042 | 0.081 | 4582 |
| 3122 | 690 | fluvoxamine | 9 µM | MCF7 | 0 | 0.042 | 0.098 | 4114 |
| 3123 | 725 | tretinoin | 1 µM | MCF7 | 0 | 0.042 | 0.065 | 5208 |
| 3124 | 688 | azacitidine | 16 µM | PC3 | 0 | 0.042 | 0.157 | 4010 |
| 3125 | 754 | propafenone | 11 µM | PC3 | 0 | 0.042 | 0.058 | 6336 |
| 3126 | 744 | clobetasol | 9 µM | MCF7 | 0 | 0.042 | 0.069 | 6835 |
| 3127 | 748 | nomifensine | 11 µM | MCF7 | 0 | 0.042 | 0.09 | 7217 |
| 3128 | 706 | lasalocid | 7 µM | MCF7 | 0 | 0.042 | 0.069 | 4985 |
| 3129 | 695 | salsolinol | 15 µM | MCF7 | 0 | 0.042 | 0.089 | 4816 |
| 3130 | 691 | cinchocaine | 12 µM | MCF7 | 0 | 0.042 | 0.064 | 4149 |
| 3131 | 681 | cloperastine | 11 µM | PC3 | 0 | 0.042 | 0.103 | 3710 |
| 3132 | 689 | thioridazine | 10 µM | PC3 | 0 | 0.041 | 0.126 | 4085 |
| 3133 | 714 | nicotinic acid | 32 µM | PC3 | 0 | 0.041 | 0.061 | 6702 |
| 3134 | 714 | meglumine | 20 µM | PC3 | 0 | 0.041 | 0.073 | 6685 |
| 3135 | 659 | ioversol | 5 µM | HL60 | 0 | 0.041 | 0.126 | 3026 |
| 3136 | 1081 | 15(S)-15-methylprostaglandin E2 | 10 µM | PC3 | 0 | 0.041 | 0.065 | 7494 |
| 3137 | 734 | ramifenazone | 14 µM | PC3 | 0 | 0.041 | 0.06 | 5879 |
| 3138 | 641 | yohimbine | 10 µM | HL60 | 0 | 0.041 | 0.116 | 1763 |
| 3139 | 680 | nitrofurantoin | 17 µM | PC3 | 0 | 0.041 | 0.106 | 3674 |
| 3140 | 635 | terconazole | 8 µM | HL60 | 0 | 0.04 | 0.104 | 2484 |
| 3141 | 681 | finasteride | 11 µM | PC3 | 0 | 0.04 | 0.084 | 3744 |
| 3142 | 698 | mephentermine | 9 µM | PC3 | 0 | 0.04 | 0.072 | 7384 |
| 3143 | 712 | N-acetyl-L-leucine | 23 µM | PC3 | 0 | 0.04 | 0.098 | 4622 |
| 3144 | 632 | oxetacaine | 9 µM | MCF7 | 0 | 0.04 | 0.085 | 1484 |
| 3145 | 725 | estradiol | 100 nM | MCF7 | 0 | 0.04 | 0.047 | 5205 |
| 3146 | 691 | xylazine | 18 µM | MCF7 | 0 | 0.04 | 0.097 | 4147 |
| 3147 | 1034 | AG-028671 | 10 µM | PC3 | 0 | 0.04 | 0.064 | 6557 |
| 3148 | 622 | mebeverine | 9 µM | HL60 | 0 | 0.039 | 0.106 | 1576 |
| 3149 | 753 | nizatidine | 12 µM | PC3 | 0 | 0.039 | 0.068 | 6305 |
| 3150 | 711 | promazine | 12 µM | MCF7 | 0 | 0.039 | 0.134 | 3927 |
| 3151 | 732 | Prestwick-1085 | 15 µM | PC3 | 0 | 0.039 | 0.085 | 5774 |
| 3152 | 685 | medrysone | 12 µM | MCF7 | 0 | 0.039 | 0.111 | 3603 |
| 3153 | 727 | valproic acid | 200 µM | PC3 | 0 | 0.039 | 0.07 | 4438 |
| 3154 | 692 | roxithromycin | 5 µM | PC3 | 0 | 0.038 | 0.066 | 4192 |
| 3155 | 1026 | erastin | 20 µM | MCF7 | 0 | 0.038 | 0.072 | 6412 |
| 3156 | 715 | pipemidic acid | 13 µM | PC3 | 0 | 0.038 | 0.086 | 6752 |
| 3157 | 736 | naproxen | 17 µM | MCF7 | 0 | 0.038 | 0.06 | 5457 |
| 3158 | 677 | moracizine | 9 µM | MCF7 | 0 | 0.038 | 0.134 | 3520 |
| 3159 | 761 | disopyramide | 12 µM | PC3 | 0 | 0.038 | 0.077 | 7276 |
| 3160 | 661 | ursodeoxycholic acid | 10 µM | HL60 | 0 | 0.038 | 0.114 | 3105 |
| 3161 | 680 | kawain | 17 µM | PC3 | 0 | 0.038 | 0.078 | 3670 |
| 3162 | 692 | minoxidil | 19 µM | PC3 | 0 | 0.037 | 0.062 | 4216 |
| 3163 | 762 | iobenguane | 11 µM | PC3 | 0 | 0.037 | 0.061 | 7299 |
| 3164 | 700 | bromopride | 12 µM | MCF7 | 0 | 0.037 | 0.065 | 4741 |
| 3165 | 627 | meticrane | 15 µM | MCF7 | 0 | 0.037 | 0.077 | 1671 |
| 3166 | 619 | prednisolone | 11 µM | HL60 | 0 | 0.037 | 0.15 | 2393 |
| 3167 | 623 | alfuzosin | 9 µM | HL60 | 0 | 0.036 | 0.113 | 1586 |
| 3168 | 647 | hemicholinium | 7 µM | MCF7 | 0 | 0.036 | 0.083 | 3216 |
| 3169 | 623 | sulfafurazole | 15 µM | HL60 | 0 | 0.035 | 0.122 | 1603 |
| 3170 | 709 | khellin | 15 µM | PC3 | 0 | 0.035 | 0.05 | 6641 |
| 3171 | 758 | latamoxef | 7 µM | MCF7 | 0 | 0.035 | 0.116 | 5609 |
| 3172 | 688 | ethambutol | 14 µM | PC3 | 0 | 0.034 | 0.082 | 4001 |
| 3173 | 727 | haloperidol | 10 µM | PC3 | 0 | 0.034 | 0.06 | 4468 |
| 3174 | 751 | betonicine | 25 µM | MCF7 | 0 | 0.034 | 0.085 | 6063 |
| 3175 | 709 | meclocycline | 6 µM | PC3 | 0 | 0.033 | 0.06 | 6637 |
| 3176 | 1048 | fulvestrant | 1 µM | PC3 | 0 | 0.032 | 0.086 | 6867 |
| 3177 | 701 | meclofenoxate | 14 µM | PC3 | 0 | 0.031 | 0.097 | 4268 |
| 3178 | 737 | lobelanidine | 11 µM | MCF7 | 0 | -0.028 | -0.101 | 5500 |
| 3179 | 738 | pyrantel | 11 µM | MCF7 | 0 | -0.028 | -0.072 | 5513 |
| 3180 | 703 | CP-320650-01 | 1 µM | PC3 | 0 | -0.029 | -0.075 | 4560 |
| 3181 | 703 | chlorambucil | 13 µM | PC3 | 0 | -0.031 | -0.045 | 4523 |
| 3182 | 714 | paclitaxel | 5 µM | PC3 | 0 | -0.032 | -0.125 | 6720 |
| 3183 | 751 | Prestwick-675 | 10 µM | MCF7 | 0 | -0.035 | -0.111 | 6042 |
| 3184 | 693 | Prestwick-967 | 26 µM | PC3 | 0 | -0.035 | -0.052 | 4250 |
| 3185 | 754 | amitriptyline | 13 µM | PC3 | 0 | -0.036 | -0.135 | 6353 |
| 3186 | 728 | acepromazine | 9 µM | PC3 | 0 | -0.036 | -0.067 | 4494 |
| 3187 | 622 | benzydamine | 12 µM | HL60 | 0 | -0.037 | -0.1 | 1552 |
| 3188 | 712 | kanamycin | 7 µM | PC3 | 0 | -0.037 | -0.052 | 4625 |
| 3189 | 752 | metoprolol | 6 µM | MCF7 | 0 | -0.037 | -0.094 | 6106 |
| 3190 | 613 | chlorhexidine | 8 µM | HL60 | 0 | -0.037 | -0.12 | 2025 |
| 3191 | 637 | methocarbamol | 17 µM | MCF7 | 0 | -0.037 | -0.059 | 2274 |
| 3192 | 86 | 4,5-dianilinophthalimide | 10 µM | PC3 | 0 | -0.037 | -0.065 | 578 |
| 3193 | 715 | ethaverine | 9 µM | PC3 | 0 | -0.037 | -0.08 | 6737 |
| 3194 | 730 | xamoterol | 5 µM | MCF7 | 0 | -0.037 | -0.071 | 5363 |
| 3195 | 24 | monastrol | 100 µM | MCF7 | 0 | -0.037 | -0.041 | 311 |
| 3196 | 693 | letrozole | 14 µM | PC3 | 0 | -0.038 | -0.063 | 4240 |
| 3197 | 695 | doxylamine | 10 µM | MCF7 | 0 | -0.038 | -0.108 | 4819 |
| 3198 | 706 | Prestwick-984 | 9 µM | MCF7 | 0 | -0.038 | -0.115 | 4948 |
| 3199 | 754 | naproxen | 17 µM | PC3 | 0 | -0.039 | -0.104 | 6358 |
| 3200 | 764 | berberine | 11 µM | PC3 | 0 | -0.039 | -0.051 | 7143 |
| 3201 | 743 | ribostamycin | 7 µM | MCF7 | 0 | -0.039 | -0.114 | 6765 |
| 3202 | 699 | 0173570-0000 | 1 µM | MCF7 | 0 | -0.039 | -0.054 | 4715 |
| 3203 | 628 | noretynodrel | 13 µM | PC3 | 0 | -0.039 | -0.077 | 1818 |
| 3204 | 622 | oxybutynin | 10 µM | HL60 | 0 | -0.039 | -0.084 | 1551 |
| 3205 | 647 | nicardipine | 8 µM | MCF7 | 0 | -0.039 | -0.087 | 3215 |
| 3206 | 633 | midecamycin | 5 µM | MCF7 | 0 | -0.039 | -0.093 | 1526 |
| 3207 | 688 | fusaric acid | 22 µM | PC3 | 0 | -0.039 | -0.055 | 3986 |
| 3208 | 685 | benzamil | 11 µM | MCF7 | 0 | -0.039 | -0.076 | 3635 |
| 3209 | 713 | thioperamide | 10 µM | PC3 | 0 | -0.039 | -0.092 | 4675 |
| 3210 | 678 | estriol | 14 µM | MCF7 | 0 | -0.039 | -0.098 | 3563 |
| 3211 | 734 | bambuterol | 10 µM | PC3 | 0 | -0.04 | -0.066 | 5885 |
| 3212 | 719 | bupivacaine | 12 µM | PC3 | 0 | -0.04 | -0.051 | 5112 |
| 3213 | 615 | mefexamide | 13 µM | HL60 | 0 | -0.04 | -0.101 | 1438 |
| 3214 | 754 | betazole | 27 µM | PC3 | 0 | -0.04 | -0.064 | 6344 |
| 3215 | 704 | levodopa | 20 µM | PC3 | 0 | -0.04 | -0.07 | 4571 |
| 3216 | 749 | mephenytoin | 18 µM | HL60 | 0 | -0.04 | -0.137 | 6158 |
| 3217 | 613 | bromocriptine | 5 µM | HL60 | 0 | -0.04 | -0.081 | 2007 |
| 3218 | 704 | prednisone | 11 µM | PC3 | 0 | -0.041 | -0.133 | 4577 |
| 3219 | 7 | dexverapamil | 10 µM | MCF7 | 0 | -0.041 | -0.043 | 164 |
| 3220 | 24 | NU-1025 | 100 µM | MCF7 | 0 | -0.041 | -0.064 | 313 |
| 3221 | 692 | trimethylcolchicinic acid | 12 µM | PC3 | 0 | -0.041 | -0.094 | 4202 |
| 3222 | 707 | colecalciferol | 10 µM | MCF7 | 0 | -0.041 | -0.106 | 5002 |
| 3223 | 688 | suprofen | 15 µM | PC3 | 0 | -0.041 | -0.084 | 4005 |
| 3224 | 747 | podophyllotoxin | 10 µM | MCF7 | 0 | -0.041 | -0.106 | 7198 |
| 3225 | 708 | terguride | 9 µM | MCF7 | 0 | -0.042 | -0.081 | 5694 |
| 3226 | 682 | mepenzolate bromide | 10 µM | PC3 | 0 | -0.042 | -0.068 | 3748 |
| 3227 | 731 | clotrimazole | 12 µM | PC3 | 0 | -0.042 | -0.06 | 5726 |
| 3228 | 743 | aconitine | 6 µM | MCF7 | 0 | -0.042 | -0.049 | 6797 |
| 3229 | 602 | LY-294002 | 10 µM | HL60 | 0 | -0.042 | -0.111 | 1177 |
| 3230 | 731 | dihydrostreptomycin | 3 µM | PC3 | 0 | -0.042 | -0.083 | 5751 |
| 3231 | 715 | carbachol | 22 µM | PC3 | 0 | -0.042 | -0.065 | 6742 |
| 3232 | 689 | furazolidone | 18 µM | PC3 | 0 | -0.042 | -0.058 | 4098 |
| 3233 | 1080 | 15(S)-15-methylprostaglandin E2 | 10 µM | MCF7 | 0 | -0.042 | -0.053 | 7489 |
| 3234 | 707 | velnacrine | 12 µM | MCF7 | 0 | -0.042 | -0.065 | 4997 |
| 3235 | 693 | emetine | 7 µM | PC3 | 0 | -0.042 | -0.119 | 4243 |
| 3236 | 688 | canadine | 12 µM | PC3 | 0 | -0.043 | -0.092 | 4020 |
| 3237 | 731 | butacaine | 13 µM | PC3 | 0 | -0.043 | -0.059 | 5748 |
| 3238 | 700 | isosorbide | 17 µM | MCF7 | 0 | -0.043 | -0.1 | 4742 |
| 3239 | 720 | diethylstilbestrol | 15 µM | MCF7 | 0 | -0.043 | -0.076 | 4369 |
| 3240 | 1087 | BCB000038 | 10 µM | PC3 | 0 | -0.043 | -0.125 | 7520 |
| 3241 | 758 | clenbuterol | 13 µM | MCF7 | 0 | -0.043 | -0.081 | 5631 |
| 3242 | 1075 | CAY-10397 | 10 µM | PC3 | 0 | -0.043 | -0.068 | 7087 |
| 3243 | 641 | lobeline | 11 µM | HL60 | 0 | -0.044 | -0.069 | 1770 |
| 3244 | 1 | metformin | 100 nM | MCF7 | 0 | -0.044 | -0.062 | 3 |
| 3245 | 665 | tribenoside | 8 µM | HL60 | 0 | -0.044 | -0.151 | 2946 |
| 3246 | 738 | nisoxetine | 13 µM | MCF7 | 0 | -0.044 | -0.095 | 5516 |
| 3247 | 733 | podophyllotoxin | 10 µM | PC3 | 0 | -0.044 | -0.083 | 5841 |
| 3248 | 756 | rilmenidine | 8 µM | MCF7 | 0 | -0.044 | -0.064 | 6512 |
| 3249 | 730 | anisomycin | 15 µM | MCF7 | 0 | -0.044 | -0.121 | 5364 |
| 3250 | 718 | levopropoxyphene | 7 µM | PC3 | 0 | -0.044 | -0.044 | 5083 |
| 3251 | 681 | prilocaine | 16 µM | PC3 | 0 | -0.044 | -0.155 | 3727 |
| 3252 | 628 | morantel | 11 µM | PC3 | 0 | -0.044 | -0.066 | 1798 |
| 3253 | 677 | lansoprazole | 11 µM | MCF7 | 0 | -0.045 | -0.074 | 3529 |
| 3254 | 648 | strophanthidin | 10 µM | HL60 | 0 | -0.045 | -0.08 | 2525 |
| 3255 | 663 | Prestwick-691 | 14 µM | MCF7 | 0 | -0.045 | -0.096 | 2813 |
| 3256 | 38 | raloxifene | 100 nM | ssMCF7 | 0 | -0.045 | -0.058 | 376 |
| 3257 | 1028 | STOCK1N-35215 | 10 µM | MCF7 | 0 | -0.045 | -0.057 | 6422 |
| 3258 | 640 | bromperidol | 10 µM | HL60 | 0 | -0.045 | -0.104 | 1723 |
| 3259 | 641 | atropine | 6 µM | HL60 | 0 | -0.045 | -0.153 | 1768 |
| 3260 | 623 | nicardipine | 8 µM | HL60 | 0 | -0.045 | -0.096 | 1600 |
| 3261 | 719 | parthenolide | 16 µM | PC3 | 0 | -0.045 | -0.092 | 5105 |
| 3262 | 753 | coralyne | 10 µM | PC3 | 0 | -0.045 | -0.062 | 6317 |
| 3263 | 772 | noretynodrel | 13 µM | MCF7 | 0 | -0.045 | -0.082 | 7471 |
| 3264 | 745 | disulfiram | 13 µM | MCF7 | 0 | -0.045 | -0.079 | 6210 |
| 3265 | 731 | dizocilpine | 12 µM | PC3 | 0 | -0.046 | -0.069 | 5746 |
| 3266 | 700 | PNU-0230031 | 10 µM | MCF7 | 0 | -0.046 | -0.106 | 4754 |
| 3267 | 707 | lisuride | 12 µM | MCF7 | 0 | -0.046 | -0.066 | 5028 |
| 3268 | 54 | chlorpromazine | 1 µM | MCF7 | 0 | -0.046 | -0.097 | 426 |
| 3269 | 762 | diphenhydramine | 14 µM | PC3 | 0 | -0.046 | -0.097 | 7318 |
| 3270 | 616 | nomifensine | 11 µM | PC3 | 0 | -0.046 | -0.053 | 2062 |
| 3271 | 738 | parthenolide | 16 µM | MCF7 | 0 | -0.046 | -0.072 | 5530 |
| 3272 | 629 | diphenhydramine | 14 µM | HL60 | 0 | -0.046 | -0.167 | 1871 |
| 3273 | 112 | benserazide | 10 µM | MCF7 | 0 | -0.046 | -0.061 | 641 |
| 3274 | 700 | phenformin | 17 µM | MCF7 | 0 | -0.046 | -0.077 | 4747 |
| 3275 | 692 | sulfathiazole | 16 µM | PC3 | 0 | -0.046 | -0.115 | 4183 |
| 3276 | 19 | LY-294002 | 10 µM | MCF7 | 0 | -0.046 | -0.068 | 258 |
| 3277 | 680 | guanadrel | 8 µM | PC3 | 0 | -0.046 | -0.075 | 3698 |
| 3278 | 665 | loracarbef | 11 µM | HL60 | 0 | -0.046 | -0.175 | 2970 |
| 3279 | 672 | arcaine | 15 µM | MCF7 | 0 | -0.047 | -0.078 | 3349 |
| 3280 | 1003 | PF-00562151-00 | 10 µM | PC3 | 0 | -0.047 | -0.042 | 5922 |
| 3281 | 701 | finasteride | 11 µM | PC3 | 0 | -0.047 | -0.082 | 4300 |
| 3282 | 21 | prednisolone | 1 µM | MCF7 | 0 | -0.047 | -0.058 | 265 |
| 3283 | 649 | lithocholic acid | 11 µM | HL60 | 0 | -0.047 | -0.13 | 2571 |
| 3284 | 704 | pancuronium bromide | 5 µM | PC3 | 0 | -0.047 | -0.089 | 4570 |
| 3285 | 635 | orciprenaline | 8 µM | HL60 | 0 | -0.047 | -0.143 | 2485 |
| 3286 | 677 | talampicillin | 8 µM | MCF7 | 0 | -0.047 | -0.086 | 3515 |
| 3287 | 689 | sitosterol | 10 µM | PC3 | 0 | -0.047 | -0.07 | 4073 |
| 3288 | 771 | chenodeoxycholic acid | 10 µM | MCF7 | 0 | -0.047 | -0.08 | 7433 |
| 3289 | 706 | naphazoline | 16 µM | MCF7 | 0 | -0.047 | -0.1 | 4949 |
| 3290 | 746 | mephenytoin | 18 µM | MCF7 | 0 | -0.047 | -0.08 | 6275 |
| 3291 | 764 | monocrotaline | 12 µM | PC3 | 0 | -0.047 | -0.083 | 7127 |
| 3292 | 688 | trihexyphenidyl | 12 µM | PC3 | 0 | -0.047 | -0.084 | 4015 |
| 3293 | 682 | PHA-00851261E | 1 µM | PC3 | 0 | -0.047 | -0.105 | 3776 |
| 3294 | 766 | laudanosine | 11 µM | MCF7 | 0 | -0.048 | -0.076 | 7030 |
| 3295 | 744 | (-)-isoprenaline | 16 µM | MCF7 | 0 | -0.048 | -0.083 | 6833 |
| 3296 | 707 | brinzolamide | 10 µM | MCF7 | 0 | -0.048 | -0.1 | 5016 |
| 3297 | 754 | sulfacetamide | 16 µM | PC3 | 0 | -0.048 | -0.085 | 6349 |
| 3298 | 681 | PNU-0230031 | 1 µM | PC3 | 0 | -0.048 | -0.134 | 3735 |
| 3299 | 746 | Prestwick-1085 | 15 µM | MCF7 | 0 | -0.048 | -0.063 | 6250 |
| 3300 | 728 | metoprolol | 6 µM | PC3 | 0 | -0.048 | -0.097 | 4508 |
| 3301 | 637 | meclofenamic acid | 12 µM | MCF7 | 0 | -0.048 | -0.053 | 2291 |
| 3302 | 741 | gliquidone | 8 µM | MCF7 | 0 | -0.048 | -0.134 | 6004 |
| 3303 | 665 | moracizine | 9 µM | HL60 | 0 | -0.049 | -0.154 | 2959 |
| 3304 | 617 | meclofenamic acid | 12 µM | PC3 | 0 | -0.049 | -0.071 | 2128 |
| 3305 | 732 | methazolamide | 17 µM | PC3 | 0 | -0.049 | -0.056 | 5794 |
| 3306 | 677 | iopanoic acid | 7 µM | MCF7 | 0 | -0.049 | -0.125 | 3527 |
| 3307 | 756 | alfaxalone | 12 µM | MCF7 | 0 | -0.049 | -0.059 | 6514 |
| 3308 | 1095 | meteneprost | 10 µM | PC3 | 0 | -0.049 | -0.076 | 7557 |
| 3309 | 706 | cefaclor | 10 µM | MCF7 | 0 | -0.049 | -0.064 | 4967 |
| 3310 | 1055 | SB-203580 | 1 µM | MCF7 | 0 | -0.049 | -0.085 | 6899 |
| 3311 | 754 | 10-methoxyharmalan | 19 µM | PC3 | 0 | -0.049 | -0.07 | 6355 |
| 3312 | 720 | PHA-00745360 | 1 µM | MCF7 | 0 | -0.049 | -0.113 | 4384 |
| 3313 | 617 | pentoxifylline | 14 µM | PC3 | 0 | -0.049 | -0.062 | 2127 |
| 3314 | 754 | nomegestrol | 11 µM | PC3 | 0 | -0.049 | -0.135 | 6362 |
| 3315 | 714 | diclofenamide | 13 µM | PC3 | 0 | -0.049 | -0.074 | 6686 |
| 3316 | 713 | azathioprine | 14 µM | PC3 | 0 | -0.049 | -0.061 | 4667 |
| 3317 | 745 | trioxysalen | 18 µM | MCF7 | 0 | -0.049 | -0.099 | 6216 |
| 3318 | 731 | thiethylperazine | 6 µM | PC3 | 0 | -0.049 | -0.062 | 5756 |
| 3319 | 626 | tanespimycin | 1 µM | MCF7 | 0 | -0.049 | -0.084 | 1643 |
| 3320 | 726 | cephaeline | 6 µM | MCF7 | 0 | -0.05 | -0.104 | 5247 |
| 3321 | 762 | nipecotic acid | 31 µM | PC3 | 0 | -0.05 | -0.061 | 7296 |
| 3322 | 735 | nimodipine | 10 µM | MCF7 | 0 | -0.05 | -0.075 | 5421 |
| 3323 | 764 | midodrine | 14 µM | PC3 | 0 | -0.05 | -0.048 | 7156 |
| 3324 | 602 | fluphenazine | 10 µM | HL60 | 0 | -0.05 | -0.131 | 1178 |
| 3325 | 771 | pergolide | 10 µM | MCF7 | 0 | -0.05 | -0.07 | 7434 |
| 3326 | 642 | ethisterone | 13 µM | MCF7 | 0 | -0.05 | -0.077 | 2326 |
| 3327 | 685 | karakoline | 11 µM | MCF7 | 0 | -0.05 | -0.127 | 3638 |
| 3328 | 713 | cefalexin | 11 µM | PC3 | 0 | -0.05 | -0.05 | 4654 |
| 3329 | 693 | SR-95531 | 11 µM | PC3 | 0 | -0.05 | -0.088 | 4236 |
| 3330 | 741 | ribavirin | 16 µM | MCF7 | 0 | -0.05 | -0.066 | 6018 |
| 3331 | 682 | vincamine | 11 µM | PC3 | 0 | -0.05 | -0.088 | 3784 |
| 3332 | 703 | PHA-00745360 | 1 µM | PC3 | 0 | -0.05 | -0.081 | 4562 |
| 3333 | 746 | fluorometholone | 11 µM | MCF7 | 0 | -0.05 | -0.068 | 6247 |
| 3334 | 709 | methyldopate | 15 µM | PC3 | 0 | -0.05 | -0.086 | 6640 |
| 3335 | 656 | cetirizine | 9 µM | MCF7 | 0 | -0.05 | -0.073 | 2829 |
| 3336 | 737 | ornidazole | 18 µM | MCF7 | 0 | -0.05 | -0.099 | 5483 |
| 3337 | 650 | LY-294002 | 10 µM | HL60 | 0 | -0.05 | -0.065 | 2696 |
| 3338 | 632 | trimethobenzamide | 9 µM | MCF7 | 0 | -0.05 | -0.156 | 1502 |
| 3339 | 650 | sirolimus | 100 nM | HL60 | 0 | -0.05 | -0.102 | 2702 |
| 3340 | 617 | aceclofenac | 11 µM | PC3 | 0 | -0.05 | -0.096 | 2117 |
| 3341 | 712 | Prestwick-860 | 35 µM | PC3 | 0 | -0.051 | -0.074 | 4618 |
| 3342 | 710 | methoxsalen | 19 µM | PC3 | 0 | -0.051 | -0.051 | 6661 |
| 3343 | 614 | aminophenazone | 17 µM | HL60 | 0 | -0.051 | -0.086 | 1376 |
| 3344 | 615 | mafenide | 18 µM | HL60 | 0 | -0.051 | -0.141 | 1441 |
| 3345 | 38 | dexamethasone | 1 µM | ssMCF7 | 0 | -0.051 | -0.074 | 374 |
| 3346 | 688 | amiprilose | 12 µM | PC3 | 0 | -0.051 | -0.05 | 4000 |
| 3347 | 612 | tolnaftate | 13 µM | HL60 | 0 | -0.051 | -0.069 | 2001 |
| 3348 | 765 | fluphenazine | 10 µM | MCF7 | 0 | -0.051 | -0.071 | 6996 |
| 3349 | 693 | gibberellic acid | 12 µM | PC3 | 0 | -0.051 | -0.05 | 4234 |
| 3350 | 711 | flecainide | 8 µM | MCF7 | 0 | -0.051 | -0.096 | 3937 |
| 3351 | 628 | moxisylyte | 13 µM | PC3 | 0 | -0.051 | -0.11 | 1804 |
| 3352 | 1000 | estradiol | 10 nM | MCF7 | 0 | -0.051 | -0.104 | 5905 |
| 3353 | 757 | clozapine | 10 µM | MCF7 | 0 | -0.051 | -0.099 | 5589 |
| 3354 | 514 | monensin | 11 µM | MCF7 | 0 | -0.051 | -0.058 | 1105 |
| 3355 | 662 | cinchonidine | 14 µM | MCF7 | 0 | -0.051 | -0.099 | 2772 |
| 3356 | 688 | benzathine benzylpenicillin | 4 µM | PC3 | 0 | -0.051 | -0.093 | 4022 |
| 3357 | 632 | thioridazine | 10 µM | MCF7 | 0 | -0.051 | -0.068 | 1486 |
| 3358 | 1083 | suramin sodium | 10 µM | PC3 | 0 | -0.051 | -0.109 | 7501 |
| 3359 | 637 | pentetrazol | 29 µM | MCF7 | 0 | -0.051 | -0.104 | 2255 |
| 3360 | 683 | fenbendazole | 13 µM | PC3 | 0 | -0.051 | -0.048 | 3805 |
| 3361 | 506 | LY-294002 | 10 µM | MCF7 | 0 | -0.051 | -0.078 | 1019 |
| 3362 | 754 | tubocurarine chloride | 5 µM | PC3 | 0 | -0.052 | -0.085 | 6351 |
| 3363 | 704 | chloropyramine | 12 µM | PC3 | 0 | -0.052 | -0.108 | 4589 |
| 3364 | 732 | ifosfamide | 15 µM | PC3 | 0 | -0.052 | -0.115 | 5805 |
| 3365 | 650 | alvespimycin | 100 nM | HL60 | 0 | -0.052 | -0.126 | 2673 |
| 3366 | 685 | finasteride | 11 µM | MCF7 | 0 | -0.052 | -0.057 | 3641 |
| 3367 | 757 | trifluoperazine | 10 µM | MCF7 | 0 | -0.052 | -0.057 | 5584 |
| 3368 | 736 | betazole | 27 µM | MCF7 | 0 | -0.052 | -0.112 | 5445 |
| 3369 | 640 | piracetam | 28 µM | HL60 | 0 | -0.052 | -0.131 | 1710 |
| 3370 | 714 | nitrofural | 20 µM | PC3 | 0 | -0.052 | -0.123 | 6721 |
| 3371 | 704 | vitexin | 9 µM | PC3 | 0 | -0.052 | -0.06 | 4588 |
| 3372 | 646 | tretinoin | 13 µM | MCF7 | 0 | -0.052 | -0.071 | 3165 |
| 3373 | 713 | clofazimine | 8 µM | PC3 | 0 | -0.052 | -0.098 | 4682 |
| 3374 | 708 | felbinac | 19 µM | MCF7 | 0 | -0.052 | -0.078 | 5700 |
| 3375 | 720 | phenelzine | 17 µM | MCF7 | 0 | -0.052 | -0.094 | 4360 |
| 3376 | 757 | wortmannin | 10 nM | MCF7 | 0 | -0.052 | -0.07 | 5603 |
| 3377 | 737 | Prestwick-920 | 14 µM | MCF7 | 0 | -0.052 | -0.068 | 5475 |
| 3378 | 626 | tanespimycin | 1 µM | MCF7 | 0 | -0.052 | -0.083 | 1631 |
| 3379 | 698 | guanadrel | 8 µM | PC3 | 0 | -0.052 | -0.084 | 7396 |
| 3380 | 112 | colchicine | 100 nM | MCF7 | 0 | -0.052 | -0.049 | 644 |
| 3381 | 633 | moroxydine | 19 µM | MCF7 | 0 | -0.052 | -0.058 | 1527 |
| 3382 | 1085 | 16-phenyltetranorprostaglandin E2 | 10 µM | PC3 | 0 | -0.052 | -0.049 | 7509 |
| 3383 | 677 | Prestwick-1103 | 20 µM | MCF7 | 0 | -0.053 | -0.056 | 3540 |
| 3384 | 713 | serotonin | 19 µM | PC3 | 0 | -0.053 | -0.116 | 4673 |
| 3385 | 647 | adenosine phosphate | 11 µM | MCF7 | 0 | -0.053 | -0.071 | 3237 |
| 3386 | 661 | trapidil | 19 µM | HL60 | 0 | -0.053 | -0.153 | 3136 |
| 3387 | 749 | saquinavir | 5 µM | HL60 | 0 | -0.053 | -0.113 | 6127 |
| 3388 | 671 | hecogenin | 9 µM | MCF7 | 0 | -0.053 | -0.072 | 3457 |
| 3389 | 644 | sulmazole | 14 µM | HL60 | 0 | -0.053 | -0.156 | 2153 |
| 3390 | 689 | co-dergocrine mesilate | 6 µM | PC3 | 0 | -0.053 | -0.048 | 4071 |
| 3391 | 685 | etiocholanolone | 14 µM | MCF7 | 0 | -0.053 | -0.067 | 3639 |
| 3392 | 644 | hydroxyachillin | 14 µM | HL60 | 0 | -0.053 | -0.18 | 2157 |
| 3393 | 658 | oxolamine | 9 µM | HL60 | 0 | -0.053 | -0.13 | 3006 |
| 3394 | 613 | lisuride | 12 µM | HL60 | 0 | -0.053 | -0.13 | 2046 |
| 3395 | 714 | loxapine | 9 µM | PC3 | 0 | -0.054 | -0.066 | 6694 |
| 3396 | 706 | lovastatin | 10 µM | MCF7 | 0 | -0.054 | -0.085 | 4978 |
| 3397 | 703 | thiamazole | 35 µM | PC3 | 0 | -0.054 | -0.082 | 4550 |
| 3398 | 645 | karakoline | 11 µM | HL60 | 0 | -0.054 | -0.124 | 2203 |
| 3399 | 98 | thalidomide | 100 µM | MCF7 | 0 | -0.054 | -0.09 | 606 |
| 3400 | 707 | diloxanide | 12 µM | MCF7 | 0 | -0.054 | -0.065 | 5025 |
| 3401 | 505 | 5186324 | 2 µM | MCF7 | 0 | -0.054 | -0.049 | 900 |
| 3402 | 707 | bretylium tosilate | 10 µM | MCF7 | 0 | -0.054 | -0.07 | 5020 |
| 3403 | 754 | nabumetone | 18 µM | PC3 | 0 | -0.054 | -0.128 | 6327 |
| 3404 | 615 | hydrastinine | 16 µM | HL60 | 0 | -0.054 | -0.151 | 1436 |
| 3405 | 751 | finasteride | 11 µM | MCF7 | 0 | -0.054 | -0.057 | 6062 |
| 3406 | 747 | sulfamonomethoxine | 14 µM | MCF7 | 0 | -0.054 | -0.098 | 7200 |
| 3407 | 661 | bacitracin | 3 µM | HL60 | 0 | -0.054 | -0.098 | 3109 |
| 3408 | 506 | LY-294002 | 10 µM | MCF7 | 0 | -0.054 | -0.074 | 1007 |
| 3409 | 718 | flufenamic acid | 14 µM | PC3 | 0 | -0.054 | -0.057 | 5059 |
| 3410 | 615 | tranylcypromine | 24 µM | HL60 | 0 | -0.054 | -0.109 | 1417 |
| 3411 | 6 | tamoxifen | 1 µM | MCF7 | 0 | -0.054 | -0.058 | 143 |
| 3412 | 718 | Prestwick-920 | 14 µM | PC3 | 0 | -0.054 | -0.053 | 5056 |
| 3413 | 705 | diphemanil metilsulfate | 10 µM | MCF7 | 0 | -0.054 | -0.072 | 4416 |
| 3414 | 713 | timolol | 9 µM | PC3 | 0 | -0.055 | -0.095 | 4685 |
| 3415 | 655 | fluoxetine | 12 µM | MCF7 | 0 | -0.055 | -0.083 | 3314 |
| 3416 | 718 | denatonium benzoate | 9 µM | PC3 | 0 | -0.055 | -0.098 | 5061 |
| 3417 | 1002 | rotenone | 1 µM | MCF7 | 0 | -0.055 | -0.092 | 5915 |
| 3418 | 671 | securinine | 18 µM | MCF7 | 0 | -0.055 | -0.094 | 3470 |
| 3419 | 634 | biperiden | 11 µM | HL60 | 0 | -0.055 | -0.162 | 2460 |
| 3420 | 708 | Prestwick-860 | 35 µM | MCF7 | 0 | -0.055 | -0.084 | 5679 |
| 3421 | 623 | zidovudine | 15 µM | HL60 | 0 | -0.055 | -0.096 | 1595 |
| 3422 | 711 | indapamide | 11 µM | MCF7 | 0 | -0.055 | -0.053 | 3970 |
| 3423 | 743 | antazoline | 13 µM | MCF7 | 0 | -0.055 | -0.116 | 6775 |
| 3424 | 676 | sotalol | 13 µM | MCF7 | 0 | -0.055 | -0.113 | 7338 |
| 3425 | 1053 | AG-012559 | 10 µM | MCF7 | 0 | -0.055 | -0.076 | 6889 |
| 3426 | 112 | W-13 | 10 µM | MCF7 | 0 | -0.055 | -0.134 | 643 |
| 3427 | 1074 | PHA-00846566E | 10 µM | MCF7 | 0 | -0.055 | -0.089 | 7081 |
| 3428 | 735 | paracetamol | 26 µM | MCF7 | 0 | -0.055 | -0.082 | 5384 |
| 3429 | 43 | tetraethylenepentamine | 100 µM | MCF7 | 0 | -0.055 | -0.066 | 406 |
| 3430 | 612 | khellin | 15 µM | HL60 | 0 | -0.055 | -0.12 | 2004 |
| 3431 | 695 | emetine | 7 µM | MCF7 | 0 | -0.056 | -0.048 | 4827 |
| 3432 | 647 | chlormezanone | 15 µM | MCF7 | 0 | -0.056 | -0.043 | 3235 |
| 3433 | 1092 | semustine | 100 µM | MCF7 | 0 | -0.056 | -0.063 | 7540 |
| 3434 | 627 | chlorpromazine | 11 µM | MCF7 | 0 | -0.056 | -0.093 | 1700 |
| 3435 | 749 | (-)-isoprenaline | 16 µM | HL60 | 0 | -0.056 | -0.157 | 6149 |
| 3436 | 707 | megestrol | 10 µM | MCF7 | 0 | -0.056 | -0.057 | 5013 |
| 3437 | 753 | aminophylline | 10 µM | PC3 | 0 | -0.056 | -0.069 | 6295 |
| 3438 | 680 | monensin | 6 µM | PC3 | 0 | -0.056 | -0.074 | 3704 |
| 3439 | 762 | gliquidone | 8 µM | PC3 | 0 | -0.056 | -0.079 | 7301 |
| 3440 | 692 | trichlormethiazide | 11 µM | PC3 | 0 | -0.056 | -0.092 | 4198 |
| 3441 | 676 | etilefrine | 18 µM | MCF7 | 0 | -0.056 | -0.126 | 7350 |
| 3442 | 695 | amprolium | 13 µM | MCF7 | 0 | -0.057 | -0.07 | 4825 |
| 3443 | 70 | novobiocin | 100 µM | ssMCF7 | 0 | -0.057 | -0.046 | 499 |
| 3444 | 692 | diflunisal | 16 µM | PC3 | 0 | -0.057 | -0.066 | 4210 |
| 3445 | 689 | gelsemine | 12 µM | PC3 | 0 | -0.057 | -0.05 | 4097 |
| 3446 | 704 | harman | 18 µM | PC3 | 0 | -0.057 | -0.063 | 4584 |
| 3447 | 762 | heptaminol | 22 µM | PC3 | 0 | -0.057 | -0.066 | 7313 |
| 3448 | 718 | chloramphenicol | 12 µM | PC3 | 0 | -0.057 | -0.082 | 5047 |
| 3449 | 703 | fenbendazole | 13 µM | PC3 | 0 | -0.057 | -0.045 | 4542 |
| 3450 | 654 | 7-aminocephalosporanic acid | 15 µM | MCF7 | 0 | -0.057 | -0.075 | 3258 |
| 3451 | 746 | butamben | 21 µM | MCF7 | 0 | -0.057 | -0.068 | 6266 |
| 3452 | 753 | terguride | 12 µM | PC3 | 0 | -0.057 | -0.109 | 6299 |
| 3453 | 772 | esculetin | 22 µM | MCF7 | 0 | -0.057 | -0.086 | 7459 |
| 3454 | 694 | calcium pantothenate | 8 µM | MCF7 | 0 | -0.057 | -0.08 | 4775 |
| 3455 | 642 | vincamine | 11 µM | MCF7 | 0 | -0.057 | -0.073 | 2327 |
| 3456 | 650 | genistein | 10 µM | HL60 | 0 | -0.057 | -0.152 | 2695 |
| 3457 | 663 | sparteine | 17 µM | MCF7 | 0 | -0.057 | -0.055 | 2790 |
| 3458 | 683 | CP-320650-01 | 10 µM | PC3 | 0 | -0.057 | -0.13 | 3822 |
| 3459 | 670 | thiamazole | 35 µM | MCF7 | 0 | -0.057 | -0.061 | 3432 |
| 3460 | 725 | alvespimycin | 100 nM | MCF7 | 0 | -0.057 | -0.091 | 5210 |
| 3461 | 767 | rosiglitazone | 10 µM | MCF7 | 0 | -0.057 | -0.063 | 6950 |
| 3462 | 680 | Prestwick-675 | 10 µM | PC3 | 0 | -0.058 | -0.063 | 3682 |
| 3463 | 712 | bisacodyl | 11 µM | PC3 | 0 | -0.058 | -0.06 | 4616 |
| 3464 | 615 | iproniazid | 14 µM | HL60 | 0 | -0.058 | -0.111 | 1442 |
| 3465 | 1044 | STOCK1N-35874 | 14 µM | PC3 | 0 | -0.058 | -0.055 | 6583 |
| 3466 | 754 | protriptyline | 13 µM | PC3 | 0 | -0.058 | -0.077 | 6338 |
| 3467 | 710 | velnacrine | 12 µM | PC3 | 0 | -0.058 | -0.033 | 6651 |
| 3468 | 708 | niridazole | 19 µM | MCF7 | 0 | -0.058 | -0.059 | 5682 |
| 3469 | 664 | nifurtimox | 14 µM | HL60 | 0 | -0.058 | -0.105 | 2908 |
| 3470 | 1031 | 5707885 | 50 µM | PC3 | 0 | -0.058 | -0.081 | 6438 |
| 3471 | 749 | ondansetron | 12 µM | HL60 | 0 | -0.058 | -0.137 | 6153 |
| 3472 | 8 | amitriptyline | 1 µM | MCF7 | 0 | -0.058 | -0.076 | 167 |
| 3473 | 1004 | pioglitazone | 10 µM | MCF7 | 0 | -0.058 | -0.059 | 5925 |
| 3474 | 1091 | 11-deoxy-16,16-dimethylprostaglandin E2 | 10 µM | PC3 | 0 | -0.058 | -0.1 | 7538 |
| 3475 | 702 | lanatoside C | 4 µM | PC3 | 0 | -0.058 | -0.105 | 4328 |
| 3476 | 741 | metrifonate | 16 µM | MCF7 | 0 | -0.058 | -0.07 | 5989 |
| 3477 | 681 | demeclocycline | 8 µM | PC3 | 0 | -0.058 | -0.08 | 3706 |
| 3478 | 610 | ethambutol | 14 µM | PC3 | 0 | -0.058 | -0.115 | 1900 |
| 3479 | 744 | estropipate | 9 µM | MCF7 | 0 | -0.058 | -0.156 | 6808 |
| 3480 | 682 | 0317956-0000 | 10 µM | PC3 | 0 | -0.058 | -0.079 | 3774 |
| 3481 | 75 | SC-58125 | 10 µM | HL60 | 0 | -0.058 | -0.19 | 542 |
| 3482 | 735 | nicardipine | 8 µM | MCF7 | 0 | -0.059 | -0.07 | 5397 |
| 3483 | 677 | loracarbef | 11 µM | MCF7 | 0 | -0.059 | -0.071 | 3532 |
| 3484 | 765 | valproic acid | 500 µM | MCF7 | 0 | -0.059 | -0.054 | 6999 |
| 3485 | 747 | pseudopelletierine | 21 µM | MCF7 | 0 | -0.059 | -0.072 | 7184 |
| 3486 | 709 | acebutolol | 11 µM | PC3 | 0 | -0.059 | -0.04 | 6631 |
| 3487 | 749 | tropine | 28 µM | HL60 | 0 | -0.059 | -0.107 | 6147 |
| 3488 | 636 | mianserin | 13 µM | MCF7 | 0 | -0.059 | -0.095 | 2231 |
| 3489 | 706 | methoxamine | 16 µM | MCF7 | 0 | -0.059 | -0.082 | 4972 |
| 3490 | 727 | acetylsalicylic acid | 100 µM | PC3 | 0 | -0.059 | -0.076 | 4428 |
| 3491 | 672 | lasalocid | 7 µM | MCF7 | 0 | -0.059 | -0.079 | 3360 |
| 3492 | 678 | tropine | 28 µM | MCF7 | 0 | -0.059 | -0.1 | 3569 |
| 3493 | 602 | haloperidol | 10 µM | HL60 | 0 | -0.059 | -0.164 | 1185 |
| 3494 | 662 | alpha-yohimbine | 10 µM | MCF7 | 0 | -0.059 | -0.113 | 2778 |
| 3495 | 6 | chlorpropamide | 100 µM | MCF7 | 0 | -0.059 | -0.063 | 144 |
| 3496 | 637 | etofylline | 18 µM | MCF7 | 0 | -0.059 | -0.091 | 2256 |
| 3497 | 619 | acemetacin | 10 µM | HL60 | 0 | -0.059 | -0.126 | 2411 |
| 3498 | 654 | carteolol | 12 µM | MCF7 | 0 | -0.059 | -0.109 | 3276 |
| 3499 | 692 | lumicolchicine | 10 µM | PC3 | 0 | -0.059 | -0.086 | 4195 |
| 3500 | 731 | tetrahydroalstonine | 11 µM | PC3 | 0 | -0.059 | -0.05 | 5728 |
| 3501 | 735 | oxybenzone | 18 µM | MCF7 | 0 | -0.059 | -0.079 | 5410 |
| 3502 | 1064 | 6-bromoindirubin-3'-oxime | 500 nM | MCF7 | 0 | -0.059 | -0.082 | 7044 |
| 3503 | 756 | tiletamine | 15 µM | MCF7 | 0 | -0.059 | -0.043 | 6516 |
| 3504 | 634 | niridazole | 19 µM | HL60 | 0 | -0.059 | -0.103 | 2440 |
| 3505 | 743 | oxybutynin | 10 µM | MCF7 | 0 | -0.059 | -0.095 | 6770 |
| 3506 | 660 | clioquinol | 13 µM | HL60 | 0 | -0.06 | -0.086 | 3084 |
| 3507 | 626 | wortmannin | 10 nM | MCF7 | 0 | -0.06 | -0.074 | 1668 |
| 3508 | 771 | minocycline | 8 µM | MCF7 | 0 | -0.06 | -0.117 | 7436 |
| 3509 | 755 | metyrapone | 18 µM | MCF7 | 0 | -0.06 | -0.049 | 6447 |
| 3510 | 746 | azlocillin | 8 µM | MCF7 | 0 | -0.06 | -0.074 | 6262 |
| 3511 | 731 | cicloheximide | 14 µM | PC3 | 0 | -0.06 | -0.073 | 5743 |
| 3512 | 670 | glycopyrronium bromide | 10 µM | MCF7 | 0 | -0.06 | -0.073 | 3427 |
| 3513 | 735 | zoxazolamine | 24 µM | MCF7 | 0 | -0.06 | -0.042 | 5390 |
| 3514 | 754 | S-propranolol | 14 µM | PC3 | 0 | -0.06 | -0.058 | 6343 |
| 3515 | 663 | palmatine | 10 µM | MCF7 | 0 | -0.06 | -0.115 | 2795 |
| 3516 | 709 | lasalocid | 7 µM | PC3 | 0 | -0.06 | -0.077 | 6639 |
| 3517 | 665 | cyclic adenosine monophosphate | 12 µM | HL60 | 0 | -0.06 | -0.192 | 2969 |
| 3518 | 658 | carbenoxolone | 7 µM | HL60 | 0 | -0.06 | -0.179 | 3014 |
| 3519 | 676 | zuclopenthixol | 9 µM | MCF7 | 0 | -0.06 | -0.092 | 7356 |
| 3520 | 741 | moracizine | 9 µM | MCF7 | 0 | -0.06 | -0.122 | 6000 |
| 3521 | 747 | isoetarine | 12 µM | MCF7 | 0 | -0.06 | -0.087 | 7170 |
| 3522 | 720 | CP-690334-01 | 1 µM | MCF7 | 0 | -0.06 | -0.104 | 4383 |
| 3523 | 1012 | LY-294002 | 10 µM | MCF7 | 0 | -0.06 | -0.081 | 5965 |
| 3524 | 39 | tacrolimus | 1 µM | MCF7 | 0 | -0.06 | -0.08 | 378 |
| 3525 | 603 | fulvestrant | 10 nM | PC3 | 0 | -0.06 | -0.053 | 1238 |
| 3526 | 755 | etynodiol | 10 µM | MCF7 | 0 | -0.06 | -0.096 | 6479 |
| 3527 | 627 | isoxicam | 12 µM | MCF7 | 0 | -0.06 | -0.082 | 1698 |
| 3528 | 731 | physostigmine | 6 µM | PC3 | 0 | -0.06 | -0.073 | 5749 |
| 3529 | 755 | promethazine | 12 µM | MCF7 | 0 | -0.061 | -0.096 | 6477 |
| 3530 | 771 | prednisolone | 11 µM | MCF7 | 0 | -0.061 | -0.068 | 7424 |
| 3531 | 705 | ethionamide | 24 µM | MCF7 | 0 | -0.061 | -0.069 | 4418 |
| 3532 | 711 | 0317956-0000 | 1 µM | MCF7 | 0 | -0.061 | -0.117 | 3969 |
| 3533 | 630 | procainamide | 15 µM | HL60 | 0 | -0.061 | -0.149 | 1263 |
| 3534 | 633 | verapamil | 8 µM | MCF7 | 0 | -0.061 | -0.06 | 1509 |
| 3535 | 673 | diloxanide | 12 µM | MCF7 | 0 | -0.061 | -0.084 | 3399 |
| 3536 | 1003 | tanespimycin | 1 µM | PC3 | 0 | -0.061 | -0.077 | 5919 |
| 3537 | 616 | tetracycline | 8 µM | PC3 | 0 | -0.061 | -0.07 | 2080 |
| 3538 | 671 | azlocillin | 8 µM | MCF7 | 0 | -0.061 | -0.112 | 3468 |
| 3539 | 647 | desipramine | 13 µM | MCF7 | 0 | -0.061 | -0.055 | 3212 |
| 3540 | 629 | homatropine | 11 µM | HL60 | 0 | -0.061 | -0.196 | 1848 |
| 3541 | 753 | hexetidine | 12 µM | PC3 | 0 | -0.061 | -0.096 | 6319 |
| 3542 | 647 | zaprinast | 15 µM | MCF7 | 0 | -0.061 | -0.102 | 3226 |
| 3543 | 108 | monastrol | 100 µM | MCF7 | 0 | -0.061 | -0.055 | 627 |
| 3544 | 514 | MG-132 | 21 µM | MCF7 | 0 | -0.061 | -0.098 | 1140 |
| 3545 | 747 | doxycycline | 8 µM | MCF7 | 0 | -0.061 | -0.083 | 7195 |
| 3546 | 702 | dantrolene | 12 µM | PC3 | 0 | -0.061 | -0.1 | 4343 |
| 3547 | 729 | meglumine | 20 µM | MCF7 | 0 | -0.061 | -0.074 | 5285 |
| 3548 | 680 | calcium folinate | 8 µM | PC3 | 0 | -0.061 | -0.055 | 3703 |
| 3549 | 514 | phenanthridinone | 51 µM | MCF7 | 0 | -0.061 | -0.105 | 1115 |
| 3550 | 513 | valproic acid | 500 µM | MCF7 | 0 | -0.061 | -0.048 | 1078 |
| 3551 | 1068 | SB-203580 | 1 µM | MCF7 | 0 | -0.061 | -0.108 | 7061 |
| 3552 | 612 | amiloride | 13 µM | HL60 | 0 | -0.061 | -0.094 | 1970 |
| 3553 | 681 | heliotrine | 13 µM | PC3 | 0 | -0.061 | -0.069 | 3717 |
| 3554 | 766 | clorsulon | 11 µM | MCF7 | 0 | -0.061 | -0.083 | 7025 |
| 3555 | 731 | ricinine | 24 µM | PC3 | 0 | -0.062 | -0.097 | 5725 |
| 3556 | 627 | moxisylyte | 13 µM | MCF7 | 0 | -0.062 | -0.114 | 1682 |
| 3557 | 676 | zardaverine | 15 µM | MCF7 | 0 | -0.062 | -0.119 | 7347 |
| 3558 | 747 | benzydamine | 12 µM | MCF7 | 0 | -0.062 | -0.105 | 7169 |
| 3559 | 647 | clofazimine | 8 µM | MCF7 | 0 | -0.062 | -0.102 | 3239 |
| 3560 | 743 | pyrithyldione | 24 µM | MCF7 | 0 | -0.062 | -0.107 | 6801 |
| 3561 | 676 | molindone | 13 µM | MCF7 | 0 | -0.062 | -0.061 | 7337 |
| 3562 | 694 | zalcitabine | 19 µM | MCF7 | 0 | -0.062 | -0.071 | 4799 |
| 3563 | 1041 | Gly-His-Lys | 1 µM | MCF7 | 0 | -0.062 | -0.056 | 6575 |
| 3564 | 651 | butacaine | 13 µM | HL60 | 0 | -0.062 | -0.104 | 2728 |
| 3565 | 744 | piretanide | 11 µM | MCF7 | 0 | -0.062 | -0.095 | 6828 |
| 3566 | 637 | etodolac | 14 µM | MCF7 | 0 | -0.062 | -0.06 | 2254 |
| 3567 | 46 | clozapine | 10 µM | MCF7 | 0 | -0.062 | -0.062 | 416 |
| 3568 | 706 | meclocycline | 6 µM | MCF7 | 0 | -0.062 | -0.088 | 4982 |
| 3569 | 704 | dicycloverine | 12 µM | PC3 | 0 | -0.062 | -0.091 | 4581 |
| 3570 | 720 | guaifenesin | 20 µM | MCF7 | 0 | -0.062 | -0.065 | 4371 |
| 3571 | 685 | foliosidine | 13 µM | MCF7 | 0 | -0.062 | -0.08 | 3636 |
| 3572 | 743 | yohimbine | 10 µM | MCF7 | 0 | -0.062 | -0.145 | 6777 |
| 3573 | 1027 | orlistat | 10 µM | PC3 | 0 | -0.062 | -0.079 | 6420 |
| 3574 | 735 | nizatidine | 12 µM | MCF7 | 0 | -0.062 | -0.123 | 5406 |
| 3575 | 660 | tonzonium bromide | 7 µM | HL60 | 0 | -0.062 | -0.094 | 3080 |
| 3576 | 738 | pentetrazol | 29 µM | MCF7 | 0 | -0.062 | -0.055 | 5508 |
| 3577 | 1032 | 5155877 | 10 µM | PC3 | 0 | -0.062 | -0.059 | 6544 |
| 3578 | 678 | milrinone | 19 µM | MCF7 | 0 | -0.062 | -0.084 | 3552 |
| 3579 | 111 | pentamidine | 100 µM | MCF7 | 0 | -0.062 | -0.065 | 639 |
| 3580 | 766 | aceclofenac | 11 µM | MCF7 | 0 | -0.063 | -0.07 | 7029 |
| 3581 | 743 | cefoxitin | 9 µM | MCF7 | 0 | -0.063 | -0.117 | 6796 |
| 3582 | 725 | LY-294002 | 10 µM | MCF7 | 0 | -0.063 | -0.11 | 5236 |
| 3583 | 602 | LY-294002 | 10 µM | HL60 | 0 | -0.063 | -0.094 | 1180 |
| 3584 | 61 | indometacin | 100 µM | PC3 | 0 | -0.063 | -0.06 | 452 |
| 3585 | 681 | foliosidine | 13 µM | PC3 | 0 | -0.063 | -0.094 | 3739 |
| 3586 | 680 | halcinonide | 9 µM | PC3 | 0 | -0.063 | -0.073 | 3680 |
| 3587 | 633 | hydroxyzine | 9 µM | MCF7 | 0 | -0.063 | -0.088 | 1524 |
| 3588 | 655 | carbimazole | 21 µM | MCF7 | 0 | -0.063 | -0.064 | 3299 |
| 3589 | 659 | hymecromone | 23 µM | HL60 | 0 | -0.063 | -0.102 | 3045 |
| 3590 | 700 | foliosidine | 13 µM | MCF7 | 0 | -0.063 | -0.068 | 4761 |
| 3591 | 602 | monorden | 100 nM | HL60 | 0 | -0.063 | -0.126 | 1160 |
| 3592 | 718 | loracarbef | 11 µM | PC3 | 0 | -0.063 | -0.071 | 5073 |
| 3593 | 705 | iocetamic acid | 7 µM | MCF7 | 0 | -0.063 | -0.074 | 4425 |
| 3594 | 671 | gabapentin | 23 µM | MCF7 | 0 | -0.063 | -0.067 | 3472 |
| 3595 | 746 | lobeline | 11 µM | MCF7 | 0 | -0.063 | -0.075 | 6258 |
| 3596 | 730 | buspirone | 9 µM | MCF7 | 0 | -0.063 | -0.042 | 5343 |
| 3597 | 755 | meglumine | 20 µM | MCF7 | 0 | -0.063 | -0.097 | 6445 |
| 3598 | 695 | letrozole | 14 µM | MCF7 | 0 | -0.063 | -0.051 | 4824 |
| 3599 | 1000 | valinomycin | 100 nM | MCF7 | 0 | -0.063 | -0.104 | 5906 |
| 3600 | 728 | aminophenazone | 17 µM | PC3 | 0 | -0.063 | -0.046 | 4481 |
| 3601 | 767 | estradiol | 10 nM | MCF7 | 0 | -0.063 | -0.103 | 6957 |
| 3602 | 685 | ketoprofen | 16 µM | MCF7 | 0 | -0.063 | -0.087 | 3626 |
| 3603 | 1075 | pioglitazone | 10 µM | PC3 | 0 | -0.063 | -0.043 | 7088 |
| 3604 | 1044 | AG-028671 | 10 µM | PC3 | 0 | -0.063 | -0.058 | 6582 |
| 3605 | 681 | bromopride | 12 µM | PC3 | 0 | -0.063 | -0.069 | 3719 |
| 3606 | 691 | betulinic acid | 9 µM | MCF7 | 0 | -0.063 | -0.064 | 4181 |
| 3607 | 1089 | suramin sodium | 10 µM | PC3 | 0 | -0.063 | -0.086 | 7529 |
| 3608 | 733 | ursolic acid | 9 µM | PC3 | 0 | -0.064 | -0.038 | 5825 |
| 3609 | 704 | novobiocin | 6 µM | PC3 | 0 | -0.064 | -0.07 | 4569 |
| 3610 | 705 | Prestwick-642 | 14 µM | MCF7 | 0 | -0.064 | -0.07 | 4419 |
| 3611 | 754 | iopanoic acid | 7 µM | PC3 | 0 | -0.064 | -0.108 | 6348 |
| 3612 | 713 | lorglumide | 8 µM | PC3 | 0 | -0.064 | -0.072 | 4658 |
| 3613 | 690 | sulmazole | 14 µM | MCF7 | 0 | -0.064 | -0.095 | 4127 |
| 3614 | 727 | tanespimycin | 1 µM | PC3 | 0 | -0.064 | -0.064 | 4442 |
| 3615 | 633 | zimeldine | 10 µM | MCF7 | 0 | -0.064 | -0.085 | 1512 |
| 3616 | 637 | flumequine | 15 µM | MCF7 | 0 | -0.064 | -0.086 | 2276 |
| 3617 | 504 | Y-27632 | 3 µM | MCF7 | 0 | -0.064 | -0.058 | 832 |
| 3618 | 751 | lycorine | 12 µM | MCF7 | 0 | -0.064 | -0.105 | 6051 |
| 3619 | 710 | glipizide | 9 µM | PC3 | 0 | -0.064 | -0.076 | 6645 |
| 3620 | 771 | niflumic acid | 14 µM | MCF7 | 0 | -0.064 | -0.101 | 7430 |
| 3621 | 1044 | 6-bromoindirubin-3'-oxime | 500 nM | PC3 | 0 | -0.064 | -0.062 | 6585 |
| 3622 | 665 | proguanil | 14 µM | HL60 | 0 | -0.064 | -0.143 | 2944 |
| 3623 | 1083 | meteneprost | 10 µM | PC3 | 0 | -0.064 | -0.065 | 7504 |
| 3624 | 506 | 15-delta prostaglandin J2 | 10 µM | MCF7 | 0 | -0.064 | -0.075 | 1011 |
| 3625 | 756 | Prestwick-983 | 17 µM | MCF7 | 0 | -0.064 | -0.065 | 6520 |
| 3626 | 753 | metitepine | 8 µM | PC3 | 0 | -0.064 | -0.095 | 6312 |
| 3627 | 694 | trimethylcolchicinic acid | 12 µM | MCF7 | 0 | -0.064 | -0.099 | 4787 |
| 3628 | 720 | homosalate | 15 µM | MCF7 | 0 | -0.064 | -0.142 | 4355 |
| 3629 | 689 | solanine | 5 µM | PC3 | 0 | -0.064 | -0.068 | 4087 |
| 3630 | 1041 | 5155877 | 10 µM | MCF7 | 0 | -0.064 | -0.092 | 6574 |
| 3631 | 635 | trimethadione | 28 µM | HL60 | 0 | -0.064 | -0.105 | 2486 |
| 3632 | 676 | cyclopenthiazide | 11 µM | MCF7 | 0 | -0.064 | -0.105 | 7325 |
| 3633 | 695 | dydrogesterone | 13 µM | MCF7 | 0 | -0.064 | -0.086 | 4836 |
| 3634 | 631 | primaquine | 9 µM | HL60 | 0 | -0.064 | -0.154 | 1343 |
| 3635 | 729 | naftidrofuryl | 8 µM | MCF7 | 0 | -0.064 | -0.091 | 5287 |
| 3636 | 694 | gabexate | 10 µM | MCF7 | 0 | -0.065 | -0.085 | 4804 |
| 3637 | 728 | oxyphenbutazone | 12 µM | PC3 | 0 | -0.065 | -0.076 | 4506 |
| 3638 | 743 | chlortalidone | 12 µM | MCF7 | 0 | -0.065 | -0.094 | 6800 |
| 3639 | 613 | famotidine | 12 µM | HL60 | 0 | -0.065 | -0.106 | 2029 |
| 3640 | 1057 | 0297417-0002B | 10 µM | MCF7 | 0 | -0.065 | -0.061 | 6902 |
| 3641 | 700 | etiocholanolone | 14 µM | MCF7 | 0 | -0.065 | -0.058 | 4764 |
| 3642 | 707 | maprotiline | 13 µM | MCF7 | 0 | -0.065 | -0.054 | 5022 |
| 3643 | 649 | diethylstilbestrol | 15 µM | HL60 | 0 | -0.065 | -0.131 | 2567 |
| 3644 | 728 | estropipate | 9 µM | PC3 | 0 | -0.065 | -0.099 | 4472 |
| 3645 | 632 | sulfaguanidine | 19 µM | MCF7 | 0 | -0.065 | -0.098 | 1495 |
| 3646 | 737 | isometheptene | 8 µM | MCF7 | 0 | -0.065 | -0.084 | 5502 |
| 3647 | 632 | metronidazole | 23 µM | MCF7 | 0 | -0.065 | -0.06 | 1503 |
| 3648 | 634 | digoxin | 5 µM | HL60 | 0 | -0.065 | -0.113 | 2423 |
| 3649 | 764 | aconitine | 6 µM | PC3 | 0 | -0.065 | -0.102 | 7149 |
| 3650 | 640 | cyclizine | 13 µM | HL60 | 0 | -0.065 | -0.087 | 1731 |
| 3651 | 754 | pyrvinium | 3 µM | PC3 | 0 | -0.065 | -0.064 | 6339 |
| 3652 | 691 | amylocaine | 15 µM | MCF7 | 0 | -0.065 | -0.087 | 4169 |
| 3653 | 737 | mafenide | 18 µM | MCF7 | 0 | -0.065 | -0.07 | 5499 |
| 3654 | 1046 | 5194442 | 20 µM | PC3 | 0 | -0.065 | -0.071 | 6594 |
| 3655 | 688 | levobunolol | 12 µM | PC3 | 0 | -0.065 | -0.128 | 4016 |
| 3656 | 662 | stachydrine | 22 µM | MCF7 | 0 | -0.066 | -0.129 | 2743 |
| 3657 | 7a | verapamil | 10 µM | MCF7 | 0 | -0.066 | -0.03 | 161 |
| 3658 | 70 | valproic acid | 1 mM | ssMCF7 | 0 | -0.066 | -0.064 | 497 |
| 3659 | 1038 | PNU-0293363 | 10 µM | PC3 | 0 | -0.066 | -0.076 | 6563 |
| 3660 | 706 | liothyronine | 6 µM | MCF7 | 0 | -0.066 | -0.103 | 4947 |
| 3661 | 610 | pheniramine | 11 µM | PC3 | 0 | -0.066 | -0.042 | 1910 |
| 3662 | 767 | alvespimycin | 100 nM | MCF7 | 0 | -0.066 | -0.086 | 6933 |
| 3663 | 678 | azaperone | 12 µM | MCF7 | 0 | -0.066 | -0.065 | 3573 |
| 3664 | 751 | 2,6-dimethylpiperidine | 27 µM | MCF7 | 0 | -0.066 | -0.053 | 6049 |
| 3665 | 1067 | 0316684-0000 | 10 µM | PC3 | 0 | -0.066 | -0.057 | 7057 |
| 3666 | 702 | vincamine | 11 µM | PC3 | 0 | -0.066 | -0.059 | 4341 |
| 3667 | 623 | maprotiline | 13 µM | HL60 | 0 | -0.066 | -0.11 | 1621 |
| 3668 | 678 | benzylpenicillin | 11 µM | MCF7 | 0 | -0.066 | -0.115 | 3577 |
| 3669 | 663 | conessine | 11 µM | MCF7 | 0 | -0.066 | -0.073 | 2792 |
| 3670 | 673 | bemegride | 26 µM | MCF7 | 0 | -0.066 | -0.124 | 3389 |
| 3671 | 746 | tremorine | 15 µM | MCF7 | 0 | -0.066 | -0.074 | 6273 |
| 3672 | 616 | dihydrostreptomycin | 3 µM | PC3 | 0 | -0.066 | -0.093 | 2074 |
| 3673 | 610 | prednisone | 11 µM | PC3 | 0 | -0.066 | -0.097 | 1897 |
| 3674 | 758 | cephaeline | 6 µM | MCF7 | 0 | -0.066 | -0.116 | 5612 |
| 3675 | 702 | ethisterone | 13 µM | PC3 | 0 | -0.066 | -0.062 | 4340 |
| 3676 | 682 | fludrocortisone | 9 µM | PC3 | 0 | -0.066 | -0.076 | 3785 |
| 3677 | 744 | cefalotin | 10 µM | MCF7 | 0 | -0.067 | -0.102 | 6819 |
| 3678 | 746 | demecarium bromide | 6 µM | MCF7 | 0 | -0.067 | -0.099 | 6269 |
| 3679 | 613 | naloxone | 11 µM | HL60 | 0 | -0.067 | -0.1 | 2006 |
| 3680 | 728 | hydrocotarnine | 13 µM | PC3 | 0 | -0.067 | -0.061 | 4489 |
| 3681 | 640 | hydrastine hydrochloride | 10 µM | HL60 | 0 | -0.067 | -0.099 | 1740 |
| 3682 | 718 | imidurea | 10 µM | PC3 | 0 | -0.067 | -0.107 | 5062 |
| 3683 | 58 | W-13 | 10 µM | MCF7 | 0 | -0.067 | -0.049 | 440 |
| 3684 | 687 | homosalate | 15 µM | MCF7 | 0 | -0.067 | -0.108 | 3879 |
| 3685 | 633 | amoxapine | 13 µM | MCF7 | 0 | -0.067 | -0.077 | 1513 |
| 3686 | 731 | sulfametoxydiazine | 14 µM | PC3 | 0 | -0.067 | -0.121 | 5732 |
| 3687 | 687 | ciclacillin | 12 µM | MCF7 | 0 | -0.067 | -0.082 | 3882 |
| 3688 | 751 | diphenylpyraline | 13 µM | MCF7 | 0 | -0.067 | -0.083 | 6061 |
| 3689 | 646 | chlortalidone | 12 µM | MCF7 | 0 | -0.067 | -0.062 | 3198 |
| 3690 | 757 | geldanamycin | 1 µM | MCF7 | 0 | -0.067 | -0.055 | 5588 |
| 3691 | 663 | canadine | 12 µM | MCF7 | 0 | -0.067 | -0.087 | 2818 |
| 3692 | 648 | ramifenazone | 14 µM | HL60 | 0 | -0.067 | -0.151 | 2534 |
| 3693 | 743 | isopropamide iodide | 8 µM | MCF7 | 0 | -0.067 | -0.071 | 6781 |
| 3694 | 649 | calcium folinate | 8 µM | HL60 | 0 | -0.067 | -0.16 | 2579 |
| 3695 | 689 | Prestwick-691 | 14 µM | PC3 | 0 | -0.067 | -0.082 | 4092 |
| 3696 | 729 | ambroxol | 10 µM | MCF7 | 0 | -0.067 | -0.078 | 5319 |
| 3697 | 681 | flunixin | 8 µM | PC3 | 0 | -0.067 | -0.063 | 3713 |
| 3698 | 656 | terconazole | 8 µM | MCF7 | 0 | -0.067 | -0.056 | 2844 |
| 3699 | 630 | prochlorperazine | 7 µM | HL60 | 0 | -0.067 | -0.147 | 1286 |
| 3700 | 631 | SR-95639A | 10 µM | HL60 | 0 | -0.067 | -0.145 | 1336 |
| 3701 | 633 | chlortetracycline | 8 µM | MCF7 | 0 | -0.067 | -0.051 | 1541 |
| 3702 | 685 | myosmine | 27 µM | MCF7 | 0 | -0.067 | -0.069 | 3634 |
| 3703 | 665 | viomycin | 5 µM | HL60 | 0 | -0.067 | -0.185 | 2979 |
| 3704 | 733 | benzydamine | 12 µM | PC3 | 0 | -0.067 | -0.069 | 5811 |
| 3705 | 631 | cyclobenzaprine | 13 µM | HL60 | 0 | -0.068 | -0.189 | 1332 |
| 3706 | 744 | stachydrine | 22 µM | MCF7 | 0 | -0.068 | -0.06 | 6805 |
| 3707 | 687 | thiamazole | 35 µM | MCF7 | 0 | -0.068 | -0.096 | 3898 |
| 3708 | 764 | nocodazole | 13 µM | PC3 | 0 | -0.068 | -0.119 | 7145 |
| 3709 | 700 | finasteride | 11 µM | MCF7 | 0 | -0.068 | -0.134 | 4766 |
| 3710 | 688 | metronidazole | 23 µM | PC3 | 0 | -0.068 | -0.076 | 4023 |
| 3711 | 728 | benzylpenicillin | 11 µM | PC3 | 0 | -0.068 | -0.089 | 4501 |
| 3712 | 726 | clozapine | 12 µM | MCF7 | 0 | -0.068 | -0.082 | 5265 |
| 3713 | 686 | vincamine | 11 µM | MCF7 | 0 | -0.068 | -0.121 | 3865 |
| 3714 | 54 | staurosporine | 10 nM | MCF7 | 0 | -0.068 | -0.1 | 425 |
| 3715 | 39 | tamoxifen | 1 µM | MCF7 | 0 | -0.068 | -0.053 | 380 |
| 3716 | 7 | rofecoxib | 10 µM | MCF7 | 0 | -0.068 | -0.083 | 166 |
| 3717 | 743 | pimozide | 9 µM | MCF7 | 0 | -0.068 | -0.058 | 6780 |
| 3718 | 690 | fluticasone | 8 µM | MCF7 | 0 | -0.068 | -0.1 | 4129 |
| 3719 | 698 | atractyloside | 5 µM | PC3 | 0 | -0.068 | -0.065 | 7393 |
| 3720 | 626 | LY-294002 | 10 µM | MCF7 | 0 | -0.068 | -0.082 | 1664 |
| 3721 | 712 | practolol | 15 µM | PC3 | 0 | -0.068 | -0.049 | 4603 |
| 3722 | 641 | harmine | 16 µM | HL60 | 0 | -0.068 | -0.137 | 1758 |
| 3723 | 744 | ampyrone | 20 µM | MCF7 | 0 | -0.068 | -0.088 | 6845 |
| 3724 | 673 | propranolol | 14 µM | MCF7 | 0 | -0.068 | -0.072 | 3396 |
| 3725 | 738 | hyoscyamine | 14 µM | MCF7 | 0 | -0.068 | -0.088 | 5524 |
| 3726 | 712 | oleandomycin | 5 µM | PC3 | 0 | -0.068 | -0.076 | 4615 |
| 3727 | 727 | geldanamycin | 1 µM | PC3 | 0 | -0.068 | -0.078 | 4452 |
| 3728 | 650 | thioridazine | 10 µM | HL60 | 0 | -0.068 | -0.095 | 2690 |
| 3729 | 735 | arecoline | 17 µM | MCF7 | 0 | -0.068 | -0.078 | 5423 |
| 3730 | 6 | tolbutamide | 100 µM | MCF7 | 0 | -0.068 | -0.051 | 142 |
| 3731 | 713 | biperiden | 11 µM | PC3 | 0 | -0.068 | -0.124 | 4684 |
| 3732 | 665 | S-propranolol | 14 µM | HL60 | 0 | -0.069 | -0.11 | 2961 |
| 3733 | 631 | carbarsone | 15 µM | HL60 | 0 | -0.069 | -0.178 | 1313 |
| 3734 | 751 | benzamil | 11 µM | MCF7 | 0 | -0.069 | -0.073 | 6056 |
| 3735 | 636 | neomycin | 4 µM | MCF7 | 0 | -0.069 | -0.058 | 2229 |
| 3736 | 699 | glycopyrronium bromide | 10 µM | MCF7 | 0 | -0.069 | -0.073 | 4709 |
| 3737 | 634 | cefotiam | 7 µM | HL60 | 0 | -0.069 | -0.178 | 2458 |
| 3738 | 714 | promethazine | 12 µM | PC3 | 0 | -0.069 | -0.052 | 6717 |
| 3739 | 636 | tranexamic acid | 25 µM | MCF7 | 0 | -0.069 | -0.107 | 2248 |
| 3740 | 746 | dorzolamide | 11 µM | MCF7 | 0 | -0.069 | -0.095 | 6259 |
| 3741 | 671 | iopromide | 5 µM | MCF7 | 0 | -0.069 | -0.087 | 3481 |
| 3742 | 747 | streptomycin | 3 µM | MCF7 | 0 | -0.069 | -0.065 | 7194 |
| 3743 | 766 | artemisinin | 14 µM | MCF7 | 0 | -0.069 | -0.128 | 7007 |
| 3744 | 1075 | PHA-00846566E | 10 µM | PC3 | 0 | -0.069 | -0.093 | 7086 |
| 3745 | 1080 | fulvestrant | 1 µM | MCF7 | 0 | -0.069 | -0.06 | 7490 |
| 3746 | 743 | chlorphenamine | 10 µM | MCF7 | 0 | -0.069 | -0.107 | 6773 |
| 3747 | 766 | naftifine | 12 µM | MCF7 | 0 | -0.069 | -0.058 | 7032 |
| 3748 | 745 | racecadotril | 10 µM | MCF7 | 0 | -0.069 | -0.089 | 6231 |
| 3749 | 702 | 0317956-0000 | 1 µM | PC3 | 0 | -0.069 | -0.098 | 4334 |
| 3750 | 505 | 5186223 | 12 µM | MCF7 | 0 | -0.069 | -0.065 | 885 |
| 3751 | 690 | benzathine benzylpenicillin | 4 µM | MCF7 | 0 | -0.069 | -0.048 | 4140 |
| 3752 | 513 | tanespimycin | 1 µM | MCF7 | 0 | -0.069 | -0.071 | 1056 |
| 3753 | 687 | santonin | 16 µM | MCF7 | 0 | -0.069 | -0.097 | 3877 |
| 3754 | 746 | papaverine | 11 µM | MCF7 | 0 | -0.069 | -0.083 | 6245 |
| 3755 | 665 | procarbazine | 16 µM | HL60 | 0 | -0.069 | -0.144 | 2971 |
| 3756 | 644 | Prestwick-642 | 14 µM | HL60 | 0 | -0.069 | -0.098 | 2160 |
| 3757 | 687 | lithocholic acid | 11 µM | MCF7 | 0 | -0.07 | -0.101 | 3899 |
| 3758 | 29 | nitrendipine | 10 µM | MCF7 | 0 | -0.07 | -0.054 | 336 |
| 3759 | 635 | colistin | 3 µM | HL60 | 0 | -0.07 | -0.146 | 2491 |
| 3760 | 656 | omeprazole | 12 µM | MCF7 | 0 | -0.07 | -0.072 | 2828 |
| 3761 | 746 | ifosfamide | 15 µM | MCF7 | 0 | -0.07 | -0.058 | 6279 |
| 3762 | 627 | sulfadiazine | 16 µM | MCF7 | 0 | -0.07 | -0.072 | 1688 |
| 3763 | 653 | N6-methyladenosine | 14 µM | MCF7 | 0 | -0.07 | -0.072 | 2626 |
| 3764 | 505 | docosahexaenoic acid ethyl ester | 100 µM | MCF7 | 0 | -0.07 | -0.05 | 881 |
| 3765 | 702 | acacetin | 14 µM | PC3 | 0 | -0.07 | -0.058 | 4324 |
| 3766 | 632 | amiloride | 13 µM | MCF7 | 0 | -0.07 | -0.046 | 1470 |
| 3767 | 756 | octopamine | 21 µM | MCF7 | 0 | -0.07 | -0.102 | 6491 |
| 3768 | 695 | indoprofen | 14 µM | MCF7 | 0 | -0.07 | -0.053 | 4832 |
| 3769 | 690 | trihexyphenidyl | 12 µM | MCF7 | 0 | -0.07 | -0.095 | 4133 |
| 3770 | 504 | tretinoin | 1 µM | MCF7 | 0 | -0.07 | -0.054 | 849 |
| 3771 | 689 | tobramycin | 9 µM | PC3 | 0 | -0.07 | -0.065 | 4081 |
| 3772 | 758 | pirenperone | 10 µM | MCF7 | 0 | -0.07 | -0.059 | 5639 |
| 3773 | 654 | myricetin | 13 µM | MCF7 | 0 | -0.07 | -0.075 | 3270 |
| 3774 | 676 | naftopidil | 9 µM | MCF7 | 0 | -0.07 | -0.064 | 7331 |
| 3775 | 678 | kaempferol | 14 µM | MCF7 | 0 | -0.07 | -0.088 | 3579 |
| 3776 | 649 | monensin | 6 µM | HL60 | 0 | -0.07 | -0.111 | 2580 |
| 3777 | 645 | lanatoside C | 4 µM | HL60 | 0 | -0.07 | -0.103 | 2193 |
| 3778 | 751 | heliotrine | 13 µM | MCF7 | 0 | -0.07 | -0.05 | 6035 |
| 3779 | 654 | naringenin | 15 µM | MCF7 | 0 | -0.07 | -0.087 | 3278 |
| 3780 | 761 | hydroflumethiazide | 12 µM | PC3 | 0 | -0.07 | -0.059 | 7259 |
| 3781 | 700 | betonicine | 25 µM | MCF7 | 0 | -0.07 | -0.052 | 4767 |
| 3782 | 736 | iproniazid | 14 µM | MCF7 | 0 | -0.07 | -0.085 | 5458 |
| 3783 | 734 | quipazine | 9 µM | PC3 | 0 | -0.07 | -0.076 | 5887 |
| 3784 | 647 | vanoxerine | 8 µM | MCF7 | 0 | -0.071 | -0.092 | 3240 |
| 3785 | 746 | spironolactone | 10 µM | MCF7 | 0 | -0.071 | -0.052 | 6255 |
| 3786 | 755 | nilutamide | 13 µM | MCF7 | 0 | -0.071 | -0.042 | 6481 |
| 3787 | 678 | piretanide | 11 µM | MCF7 | 0 | -0.071 | -0.115 | 3567 |
| 3788 | 670 | sulfadimethoxine | 13 µM | MCF7 | 0 | -0.071 | -0.096 | 3441 |
| 3789 | 618 | flutamide | 14 µM | HL60 | 0 | -0.071 | -0.094 | 2358 |
| 3790 | 733 | pseudopelletierine | 21 µM | PC3 | 0 | -0.071 | -0.074 | 5828 |
| 3791 | 747 | fendiline | 11 µM | MCF7 | 0 | -0.071 | -0.082 | 7188 |
| 3792 | 692 | benperidol | 10 µM | PC3 | 0 | -0.071 | -0.051 | 4196 |
| 3793 | 656 | L-methionine sulfoximine | 22 µM | MCF7 | 0 | -0.071 | -0.068 | 2831 |
| 3794 | 681 | 0179445-0000 | 10 µM | PC3 | 0 | -0.071 | -0.081 | 3733 |
| 3795 | 765 | LY-294002 | 100 nM | MCF7 | 0 | -0.071 | -0.067 | 6976 |
| 3796 | 698 | PNU-0251126 | 1 µM | PC3 | 0 | -0.071 | -0.084 | 7390 |
| 3797 | 678 | repaglinide | 9 µM | MCF7 | 0 | -0.071 | -0.07 | 3558 |
| 3798 | 647 | chlorpropamide | 14 µM | MCF7 | 0 | -0.071 | -0.087 | 3210 |
| 3799 | 743 | drofenine | 11 µM | MCF7 | 0 | -0.071 | -0.137 | 6776 |
| 3800 | 645 | sulfachlorpyridazine | 14 µM | HL60 | 0 | -0.071 | -0.139 | 2191 |
| 3801 | 659 | felbinac | 19 µM | HL60 | 0 | -0.071 | -0.114 | 3061 |
| 3802 | 615 | pyrantel | 11 µM | HL60 | 0 | -0.071 | -0.08 | 1413 |
| 3803 | 636 | aminophenazone | 17 µM | MCF7 | 0 | -0.071 | -0.103 | 2222 |
| 3804 | 618 | dilazep | 6 µM | HL60 | 0 | -0.071 | -0.076 | 2333 |
| 3805 | 661 | Prestwick-920 | 14 µM | HL60 | 0 | -0.071 | -0.115 | 3118 |
| 3806 | 648 | streptozocin | 15 µM | HL60 | 0 | -0.071 | -0.144 | 2535 |
| 3807 | 755 | (-)-atenolol | 15 µM | MCF7 | 0 | -0.071 | -0.072 | 6444 |
| 3808 | 686 | phentolamine | 13 µM | MCF7 | 0 | -0.071 | -0.062 | 3860 |
| 3809 | 707 | bemegride | 26 µM | MCF7 | 0 | -0.071 | -0.093 | 5014 |
| 3810 | 736 | piribedil | 12 µM | MCF7 | 0 | -0.071 | -0.08 | 5434 |
| 3811 | 628 | carbamazepine | 17 µM | PC3 | 0 | -0.071 | -0.044 | 1805 |
| 3812 | 692 | probenecid | 14 µM | PC3 | 0 | -0.071 | -0.089 | 4185 |
| 3813 | 654 | cefixime | 9 µM | MCF7 | 0 | -0.072 | -0.077 | 3247 |
| 3814 | 735 | dobutamine | 12 µM | MCF7 | 0 | -0.072 | -0.095 | 5386 |
| 3815 | 700 | 0179445-0000 | 1 µM | MCF7 | 0 | -0.072 | -0.136 | 4758 |
| 3816 | 1027 | erastin | 20 µM | PC3 | 0 | -0.072 | -0.082 | 6417 |
| 3817 | 764 | yohimbine | 10 µM | PC3 | 0 | -0.072 | -0.073 | 7130 |
| 3818 | 1068 | SC-19220 | 10 µM | MCF7 | 0 | -0.072 | -0.111 | 7060 |
| 3819 | 111 | paclitaxel | 100 nM | MCF7 | 0 | -0.072 | -0.064 | 640 |
| 3820 | 750 | 15-delta prostaglandin J2 | 10 µM | HL60 | 0 | -0.072 | -0.121 | 6190 |
| 3821 | 31 | sodium phenylbutyrate | 100 µM | MCF7 | 0 | -0.072 | -0.069 | 341 |
| 3822 | 634 | amphotericin B | 4 µM | HL60 | 0 | -0.072 | -0.169 | 2441 |
| 3823 | 765 | haloperidol | 10 µM | MCF7 | 0 | -0.072 | -0.086 | 7003 |
| 3824 | 645 | solasodine | 10 µM | HL60 | 0 | -0.072 | -0.095 | 2170 |
| 3825 | 659 | trichostatin A | 100 nM | HL60 | 0 | -0.072 | -0.109 | 3058 |
| 3826 | 771 | diethylcarbamazine | 10 µM | MCF7 | 0 | -0.072 | -0.083 | 7425 |
| 3827 | 745 | ricinine | 24 µM | MCF7 | 0 | -0.072 | -0.106 | 6206 |
| 3828 | 96 | ciclosporin | 1 µM | MCF7 | 0 | -0.072 | -0.05 | 602 |
| 3829 | 728 | sulfanilamide | 23 µM | PC3 | 0 | -0.072 | -0.06 | 4474 |
| 3830 | 662 | DL-thiorphan | 16 µM | MCF7 | 0 | -0.072 | -0.077 | 2752 |
| 3831 | 676 | fluvoxamine | 9 µM | MCF7 | 0 | -0.072 | -0.085 | 7333 |
| 3832 | 610 | 4-hydroxyphenazone | 20 µM | PC3 | 0 | -0.072 | -0.108 | 1915 |
| 3833 | 31 | 2-deoxy-D-glucose | 10 mM | MCF7 | 0 | -0.072 | -0.08 | 344 |
| 3834 | 630 | guanfacine | 14 µM | HL60 | 0 | -0.072 | -0.13 | 1279 |
| 3835 | 702 | sulfachlorpyridazine | 14 µM | PC3 | 0 | -0.072 | -0.093 | 4326 |
| 3836 | 673 | felbinac | 19 µM | MCF7 | 0 | -0.072 | -0.068 | 3398 |
| 3837 | 689 | yohimbic acid | 11 µM | PC3 | 0 | -0.073 | -0.051 | 4082 |
| 3838 | 661 | Prestwick-972 | 23 µM | HL60 | 0 | -0.073 | -0.109 | 3132 |
| 3839 | 634 | fluoxetine | 12 µM | HL60 | 0 | -0.073 | -0.109 | 2453 |
| 3840 | 648 | podophyllotoxin | 10 µM | HL60 | 0 | -0.073 | -0.078 | 2540 |
| 3841 | 689 | selegiline | 18 µM | PC3 | 0 | -0.073 | -0.058 | 4065 |
| 3842 | 701 | betonicine | 25 µM | PC3 | 0 | -0.073 | -0.114 | 4301 |
| 3843 | 1002 | tanespimycin | 1 µM | MCF7 | 0 | -0.073 | -0.11 | 5914 |
| 3844 | 628 | allantoin | 25 µM | PC3 | 0 | -0.073 | -0.087 | 1800 |
| 3845 | 649 | sulfadimethoxine | 13 µM | HL60 | 0 | -0.073 | -0.159 | 2578 |
| 3846 | 772 | cortisone | 11 µM | MCF7 | 0 | -0.073 | -0.103 | 7458 |
| 3847 | 655 | methoxsalen | 19 µM | MCF7 | 0 | -0.073 | -0.055 | 3302 |
| 3848 | 631 | lumicolchicine | 10 µM | HL60 | 0 | -0.073 | -0.215 | 1317 |
| 3849 | 629 | adiphenine | 11 µM | HL60 | 0 | -0.073 | -0.157 | 1872 |
| 3850 | 657 | dihydroergocristine | 6 µM | MCF7 | 0 | -0.073 | -0.069 | 2895 |
| 3851 | 709 | fursultiamine | 9 µM | PC3 | 0 | -0.073 | -0.063 | 6630 |
| 3852 | 737 | levopropoxyphene | 7 µM | MCF7 | 0 | -0.073 | -0.047 | 5503 |
| 3853 | 754 | dimenhydrinate | 9 µM | PC3 | 0 | -0.073 | -0.112 | 6352 |
| 3854 | 690 | canadine | 12 µM | MCF7 | 0 | -0.073 | -0.083 | 4138 |
| 3855 | 649 | alexidine | 7 µM | HL60 | 0 | -0.073 | -0.099 | 2576 |
| 3856 | 765 | sirolimus | 100 nM | MCF7 | 0 | -0.073 | -0.087 | 7001 |
| 3857 | 663 | succinylsulfathiazole | 11 µM | MCF7 | 0 | -0.073 | -0.11 | 2821 |
| 3858 | 506 | estradiol | 10 nM | MCF7 | 0 | -0.073 | -0.106 | 1021 |
| 3859 | 688 | sulmazole | 14 µM | PC3 | 0 | -0.074 | -0.077 | 4009 |
| 3860 | 690 | sisomicin | 3 µM | MCF7 | 0 | -0.074 | -0.091 | 4132 |
| 3861 | 725 | tanespimycin | 1 µM | MCF7 | 0 | -0.074 | -0.059 | 5215 |
| 3862 | 623 | desipramine | 13 µM | HL60 | 0 | -0.074 | -0.094 | 1596 |
| 3863 | 618 | fludrocortisone | 9 µM | HL60 | 0 | -0.074 | -0.109 | 2368 |
| 3864 | 693 | seneciphylline | 12 µM | PC3 | 0 | -0.074 | -0.072 | 4238 |
| 3865 | 766 | enalapril | 8 µM | MCF7 | 0 | -0.074 | -0.083 | 7026 |
| 3866 | 502 | 5182598 | 25 µM | MCF7 | 0 | -0.074 | -0.094 | 976 |
| 3867 | 687 | nialamide | 13 µM | MCF7 | 0 | -0.074 | -0.084 | 3871 |
| 3868 | 755 | benserazide | 14 µM | MCF7 | 0 | -0.074 | -0.106 | 6482 |
| 3869 | 626 | valproic acid | 500 µM | MCF7 | 0 | -0.074 | -0.06 | 1665 |
| 3870 | 663 | co-dergocrine mesilate | 6 µM | MCF7 | 0 | -0.074 | -0.135 | 2793 |
| 3871 | 640 | iobenguane | 11 µM | HL60 | 0 | -0.074 | -0.13 | 1729 |
| 3872 | 671 | mevalolactone | 31 µM | MCF7 | 0 | -0.074 | -0.095 | 3459 |
| 3873 | 505 | 5286656 | 50 µM | MCF7 | 0 | -0.074 | -0.105 | 889 |
| 3874 | 706 | acebutolol | 11 µM | MCF7 | 0 | -0.074 | -0.061 | 4976 |
| 3875 | 733 | triamcinolone | 10 µM | PC3 | 0 | -0.074 | -0.078 | 5835 |
| 3876 | 506 | valproic acid | 500 µM | MCF7 | 0 | -0.074 | -0.065 | 1020 |
| 3877 | 603 | 15-delta prostaglandin J2 | 10 µM | PC3 | 0 | -0.074 | -0.064 | 1231 |
| 3878 | 751 | etiocholanolone | 14 µM | MCF7 | 0 | -0.074 | -0.114 | 6060 |
| 3879 | 1094 | vinblastine | 100 nM | MCF7 | 0 | -0.074 | -0.055 | 7551 |
| 3880 | 764 | drofenine | 11 µM | PC3 | 0 | -0.074 | -0.067 | 7129 |
| 3881 | 1077 | lomustine | 100 µM | PC3 | 0 | -0.074 | -0.076 | 7094 |
| 3882 | 771 | lisinopril | 9 µM | MCF7 | 0 | -0.075 | -0.069 | 7403 |
| 3883 | 651 | bergenin | 12 µM | HL60 | 0 | -0.075 | -0.091 | 2726 |
| 3884 | 725 | sirolimus | 100 nM | MCF7 | 0 | -0.075 | -0.069 | 5239 |
| 3885 | 38 | estradiol | 10 nM | ssMCF7 | 0 | -0.075 | -0.104 | 373 |
| 3886 | 733 | cinchonidine | 14 µM | PC3 | 0 | -0.075 | -0.085 | 5833 |
| 3887 | 726 | clenbuterol | 13 µM | MCF7 | 0 | -0.075 | -0.08 | 5266 |
| 3888 | 653 | coralyne | 10 µM | MCF7 | 0 | -0.075 | -0.06 | 2652 |
| 3889 | 654 | bacampicillin | 8 µM | MCF7 | 0 | -0.075 | -0.105 | 3273 |
| 3890 | 735 | 2-aminobenzenesulfonamide | 23 µM | MCF7 | 0 | -0.075 | -0.103 | 5422 |
| 3891 | 619 | dimenhydrinate | 9 µM | HL60 | 0 | -0.075 | -0.109 | 2400 |
| 3892 | 703 | CP-690334-01 | 1 µM | PC3 | 0 | -0.075 | -0.098 | 4561 |
| 3893 | 649 | merbromin | 5 µM | HL60 | 0 | -0.075 | -0.114 | 2577 |
| 3894 | 637 | tiratricol | 6 µM | MCF7 | 0 | -0.075 | -0.101 | 2259 |
| 3895 | 630 | fusidic acid | 7 µM | HL60 | 0 | -0.075 | -0.111 | 1293 |
| 3896 | 746 | hexylcaine | 13 µM | MCF7 | 0 | -0.075 | -0.077 | 6244 |
| 3897 | 754 | isradipine | 11 µM | PC3 | 0 | -0.075 | -0.139 | 6347 |
| 3898 | 695 | iodixanol | 3 µM | MCF7 | 0 | -0.075 | -0.06 | 4848 |
| 3899 | 672 | indoprofen | 14 µM | MCF7 | 0 | -0.075 | -0.137 | 3345 |
| 3900 | 757 | monorden | 100 nM | MCF7 | 0 | -0.075 | -0.101 | 5579 |
| 3901 | 771 | bumetanide | 11 µM | MCF7 | 0 | -0.075 | -0.1 | 7440 |
| 3902 | 505 | carbamazepine | 100 nM | MCF7 | 0 | -0.075 | -0.055 | 919 |
| 3903 | 660 | dexibuprofen | 19 µM | HL60 | 0 | -0.075 | -0.143 | 3094 |
| 3904 | 735 | metolazone | 11 µM | MCF7 | 0 | -0.075 | -0.114 | 5392 |
| 3905 | 730 | ceforanide | 8 µM | MCF7 | 0 | -0.075 | -0.084 | 5351 |
| 3906 | 658 | (+/-)-catechin | 14 µM | HL60 | 0 | -0.075 | -0.122 | 3012 |
| 3907 | 619 | chenodeoxycholic acid | 10 µM | HL60 | 0 | -0.075 | -0.101 | 2402 |
| 3908 | 632 | diflunisal | 16 µM | MCF7 | 0 | -0.075 | -0.073 | 1490 |
| 3909 | 633 | aciclovir | 18 µM | MCF7 | 0 | -0.076 | -0.084 | 1543 |
| 3910 | 65 | troglitazone | 10 µM | PC3 | 0 | -0.076 | -0.059 | 462 |
| 3911 | 617 | norfloxacin | 13 µM | PC3 | 0 | -0.076 | -0.042 | 2090 |
| 3912 | 631 | SR-95531 | 11 µM | HL60 | 0 | -0.076 | -0.161 | 1316 |
| 3913 | 1 | metformin | 10 µM | MCF7 | 0 | -0.076 | -0.081 | 2 |
| 3914 | 673 | xamoterol | 5 µM | MCF7 | 0 | -0.076 | -0.076 | 3401 |
| 3915 | 705 | tiabendazole | 20 µM | MCF7 | 0 | -0.076 | -0.108 | 4402 |
| 3916 | 71 | troglitazone | 10 µM | SKMEL5 | 0 | -0.076 | -0.055 | 504 |
| 3917 | 627 | 8-azaguanine | 26 µM | MCF7 | 0 | -0.076 | -0.062 | 1670 |
| 3918 | 640 | dosulepin | 12 µM | HL60 | 0 | -0.076 | -0.113 | 1713 |
| 3919 | 744 | astemizole | 9 µM | MCF7 | 0 | -0.076 | -0.063 | 6807 |
| 3920 | 619 | fenofibrate | 11 µM | HL60 | 0 | -0.076 | -0.112 | 2401 |
| 3921 | 676 | tracazolate | 12 µM | MCF7 | 0 | -0.076 | -0.054 | 7339 |
| 3922 | 602 | estradiol | 10 nM | HL60 | 0 | -0.076 | -0.132 | 1182 |
| 3923 | 628 | procaine | 15 µM | PC3 | 0 | -0.076 | -0.069 | 1796 |
| 3924 | 681 | PNU-0230031 | 10 µM | PC3 | 0 | -0.076 | -0.086 | 3732 |
| 3925 | 658 | amiprilose | 12 µM | HL60 | 0 | -0.076 | -0.069 | 3000 |
| 3926 | 678 | cefepime | 7 µM | MCF7 | 0 | -0.076 | -0.066 | 3581 |
| 3927 | 688 | etofenamate | 11 µM | PC3 | 0 | -0.076 | -0.039 | 3989 |
| 3928 | 698 | levomepromazine | 9 µM | PC3 | 0 | -0.077 | -0.042 | 7399 |
| 3929 | 733 | ipratropium bromide | 10 µM | PC3 | 0 | -0.077 | -0.048 | 5823 |
| 3930 | 772 | Prestwick-981 | 11 µM | MCF7 | 0 | -0.077 | -0.09 | 7464 |
| 3931 | 718 | prenylamine | 10 µM | PC3 | 0 | -0.077 | -0.045 | 5070 |
| 3932 | 661 | pizotifen | 9 µM | HL60 | 0 | -0.077 | -0.162 | 3134 |
| 3933 | 39 | celecoxib | 10 µM | MCF7 | 0 | -0.077 | -0.06 | 377 |
| 3934 | 1071 | PHA-00816795 | 10 µM | PC3 | 0 | -0.077 | -0.073 | 7072 |
| 3935 | 771 | perhexiline | 10 µM | MCF7 | 0 | -0.077 | -0.057 | 7441 |
| 3936 | 619 | econazole | 9 µM | HL60 | 0 | -0.077 | -0.091 | 2396 |
| 3937 | 720 | CP-320650-01 | 10 µM | MCF7 | 0 | -0.077 | -0.072 | 4379 |
| 3938 | 38 | tamoxifen | 1 µM | ssMCF7 | 0 | -0.077 | -0.058 | 375 |
| 3939 | 632 | hexamethonium bromide | 10 µM | MCF7 | 0 | -0.077 | -0.059 | 1482 |
| 3940 | 662 | aconitine | 6 µM | MCF7 | 0 | -0.077 | -0.101 | 2776 |
| 3941 | 635 | rifampicin | 5 µM | HL60 | 0 | -0.077 | -0.138 | 2487 |
| 3942 | 75 | 1,5-isoquinolinediol | 100 µM | HL60 | 0 | -0.077 | -0.114 | 543 |
| 3943 | 602 | clozapine | 10 µM | HL60 | 0 | -0.077 | -0.133 | 1170 |
| 3944 | 612 | minaprine | 11 µM | HL60 | 0 | -0.077 | -0.094 | 1968 |
| 3945 | 738 | dropropizine | 17 µM | MCF7 | 0 | -0.077 | -0.061 | 5531 |
| 3946 | 699 | kawain | 17 µM | MCF7 | 0 | -0.077 | -0.06 | 4693 |
| 3947 | 683 | paroxetine | 1 µM | PC3 | 0 | -0.077 | -0.069 | 3821 |
| 3948 | 729 | flucytosine | 31 µM | MCF7 | 0 | -0.077 | -0.084 | 5289 |
| 3949 | 610 | amprolium | 13 µM | PC3 | 0 | -0.077 | -0.089 | 1898 |
| 3950 | 629 | chlorpromazine | 11 µM | HL60 | 0 | -0.077 | -0.161 | 1864 |
| 3951 | 703 | CP-320650-01 | 10 µM | PC3 | 0 | -0.077 | -0.054 | 4557 |
| 3952 | 682 | 0317956-0000 | 1 µM | PC3 | 0 | -0.077 | -0.083 | 3777 |
| 3953 | 658 | theophylline | 20 µM | HL60 | 0 | -0.077 | -0.143 | 2986 |
| 3954 | 683 | lithocholic acid | 11 µM | PC3 | 0 | -0.077 | -0.092 | 3816 |
| 3955 | 707 | dicloxacillin | 8 µM | MCF7 | 0 | -0.077 | -0.076 | 5012 |
| 3956 | 766 | meclofenamic acid | 12 µM | MCF7 | 0 | -0.077 | -0.071 | 7038 |
| 3957 | 513 | tanespimycin | 1 µM | MCF7 | 0 | -0.078 | -0.078 | 1063 |
| 3958 | 733 | pyridoxine | 19 µM | PC3 | 0 | -0.078 | -0.065 | 5813 |
| 3959 | 1086 | 11-deoxy-16,16-dimethylprostaglandin E2 | 10 µM | MCF7 | 0 | -0.078 | -0.08 | 7514 |
| 3960 | 737 | harmalol | 15 µM | MCF7 | 0 | -0.078 | -0.13 | 5495 |
| 3961 | 749 | cefepime | 7 µM | HL60 | 0 | -0.078 | -0.099 | 6159 |
| 3962 | 771 | enalapril | 8 µM | MCF7 | 0 | -0.078 | -0.117 | 7428 |
| 3963 | 728 | napelline | 11 µM | PC3 | 0 | -0.078 | -0.13 | 4486 |
| 3964 | 1066 | colforsin | 500 nM | MCF7 | 0 | -0.078 | -0.091 | 7055 |
| 3965 | 610 | niclosamide | 12 µM | PC3 | 0 | -0.078 | -0.057 | 1916 |
| 3966 | 61 | monorden | 100 nM | PC3 | 0 | -0.078 | -0.056 | 449 |
| 3967 | 655 | metixene | 12 µM | MCF7 | 0 | -0.078 | -0.088 | 3313 |
| 3968 | 627 | mefenamic acid | 17 µM | MCF7 | 0 | -0.078 | -0.088 | 1699 |
| 3969 | 626 | haloperidol | 10 µM | MCF7 | 0 | -0.078 | -0.054 | 1669 |
| 3970 | 718 | etofylline | 18 µM | PC3 | 0 | -0.078 | -0.083 | 5048 |
| 3971 | 657 | thiamine | 12 µM | MCF7 | 0 | -0.078 | -0.087 | 2894 |
| 3972 | 656 | phenacetin | 22 µM | MCF7 | 0 | -0.078 | -0.117 | 2832 |
| 3973 | 712 | domperidone | 7 µM | PC3 | 0 | -0.078 | -0.106 | 4640 |
| 3974 | 691 | tenoxicam | 12 µM | MCF7 | 0 | -0.078 | -0.153 | 4182 |
| 3975 | 602 | alpha-estradiol | 10 nM | HL60 | 0 | -0.078 | -0.109 | 1151 |
| 3976 | 756 | Prestwick-972 | 23 µM | MCF7 | 0 | -0.079 | -0.071 | 6511 |
| 3977 | 660 | estrone | 15 µM | HL60 | 0 | -0.079 | -0.158 | 3071 |
| 3978 | 627 | atracurium besilate | 3 µM | MCF7 | 0 | -0.079 | -0.088 | 1702 |
| 3979 | 69 | fluphenazine | 10 µM | SKMEL5 | 0 | -0.079 | -0.074 | 494 |
| 3980 | 632 | levodopa | 20 µM | MCF7 | 0 | -0.079 | -0.09 | 1472 |
| 3981 | 1021 | orlistat | 10 µM | PC3 | 0 | -0.079 | -0.075 | 6388 |
| 3982 | 650 | fulvestrant | 10 nM | HL60 | 0 | -0.079 | -0.118 | 2698 |
| 3983 | 725 | LY-294002 | 10 µM | MCF7 | 0 | -0.079 | -0.116 | 5224 |
| 3984 | 631 | corticosterone | 12 µM | HL60 | 0 | -0.079 | -0.169 | 1307 |
| 3985 | 750 | estradiol | 10 nM | HL60 | 0 | -0.079 | -0.137 | 6200 |
| 3986 | 676 | oxamniquine | 14 µM | MCF7 | 0 | -0.079 | -0.098 | 7344 |
| 3987 | 718 | ornidazole | 18 µM | PC3 | 0 | -0.079 | -0.057 | 5064 |
| 3988 | 731 | chloropyrazine | 35 µM | PC3 | 0 | -0.079 | -0.066 | 5750 |
| 3989 | 745 | cytisine | 21 µM | MCF7 | 0 | -0.079 | -0.076 | 6217 |
| 3990 | 744 | amrinone | 21 µM | MCF7 | 0 | -0.079 | -0.105 | 6826 |
| 3991 | 731 | tranexamic acid | 25 µM | PC3 | 0 | -0.079 | -0.142 | 5762 |
| 3992 | 660 | flucytosine | 31 µM | HL60 | 0 | -0.079 | -0.125 | 3073 |
| 3993 | 646 | ketotifen | 9 µM | MCF7 | 0 | -0.079 | -0.07 | 3200 |
| 3994 | 676 | alprostadil | 11 µM | MCF7 | 0 | -0.079 | -0.054 | 7358 |
| 3995 | 636 | clindamycin | 9 µM | MCF7 | 0 | -0.079 | -0.106 | 2219 |
| 3996 | 627 | thiamphenicol | 11 µM | MCF7 | 0 | -0.08 | -0.095 | 1704 |
| 3997 | 690 | nadolol | 13 µM | MCF7 | 0 | -0.08 | -0.079 | 4139 |
| 3998 | 687 | tinidazole | 16 µM | MCF7 | 0 | -0.08 | -0.102 | 3896 |
| 3999 | 678 | verteporfin | 3 µM | MCF7 | 0 | -0.08 | -0.065 | 3556 |
| 4000 | 650 | haloperidol | 10 µM | HL60 | 0 | -0.08 | -0.141 | 2704 |
| 4001 | 738 | bumetanide | 11 µM | MCF7 | 0 | -0.08 | -0.163 | 5542 |
| 4002 | 734 | corbadrine | 22 µM | PC3 | 0 | -0.08 | -0.034 | 5854 |
| 4003 | 731 | etacrynic acid | 13 µM | PC3 | 0 | -0.08 | -0.068 | 5742 |
| 4004 | 720 | lithocholic acid | 11 µM | MCF7 | 0 | -0.08 | -0.068 | 4373 |
| 4005 | 686 | PHA-00851261E | 10 µM | MCF7 | 0 | -0.08 | -0.093 | 3854 |
| 4006 | 627 | naproxen | 17 µM | MCF7 | 0 | -0.08 | -0.078 | 1706 |
| 4007 | 636 | cefotaxime | 8 µM | MCF7 | 0 | -0.08 | -0.079 | 2235 |
| 4008 | 741 | lymecycline | 7 µM | MCF7 | 0 | -0.08 | -0.102 | 5994 |
| 4009 | 772 | spaglumic acid | 13 µM | MCF7 | 0 | -0.08 | -0.113 | 7465 |
| 4010 | 718 | adrenosterone | 13 µM | PC3 | 0 | -0.08 | -0.079 | 5045 |
| 4011 | 648 | leflunomide | 15 µM | HL60 | 0 | -0.08 | -0.163 | 2539 |
| 4012 | 656 | tiabendazole | 20 µM | MCF7 | 0 | -0.08 | -0.06 | 2840 |
| 4013 | 658 | novobiocin | 6 µM | HL60 | 0 | -0.08 | -0.068 | 2990 |
| 4014 | 737 | tridihexethyl | 11 µM | MCF7 | 0 | -0.08 | -0.111 | 5486 |
| 4015 | 623 | colchicine | 10 µM | HL60 | 0 | -0.08 | -0.113 | 1598 |
| 4016 | 744 | aminophenazone | 17 µM | MCF7 | 0 | -0.081 | -0.076 | 6818 |
| 4017 | 640 | raubasine | 10 µM | HL60 | 0 | -0.081 | -0.102 | 1748 |
| 4018 | 642 | fenbufen | 16 µM | MCF7 | 0 | -0.081 | -0.083 | 2308 |
| 4019 | 741 | Prestwick-1103 | 20 µM | MCF7 | 0 | -0.081 | -0.069 | 6019 |
| 4020 | 651 | raloxifene | 8 µM | HL60 | 0 | -0.081 | -0.136 | 2738 |
| 4021 | 741 | pentoxifylline | 14 µM | MCF7 | 0 | -0.081 | -0.074 | 6021 |
| 4022 | 764 | canrenoic acid | 10 µM | PC3 | 0 | -0.081 | -0.097 | 7135 |
| 4023 | 710 | dicloxacillin | 8 µM | PC3 | 0 | -0.081 | -0.092 | 6666 |
| 4024 | 728 | securinine | 18 µM | PC3 | 0 | -0.081 | -0.11 | 4493 |
| 4025 | 676 | benzathine benzylpenicillin | 4 µM | MCF7 | 0 | -0.081 | -0.085 | 7359 |
| 4026 | 655 | iohexol | 5 µM | MCF7 | 0 | -0.081 | -0.121 | 3322 |
| 4027 | 610 | doxylamine | 10 µM | PC3 | 0 | -0.081 | -0.075 | 1893 |
| 4028 | 602 | estradiol | 100 nM | HL60 | 0 | -0.081 | -0.121 | 1149 |
| 4029 | 746 | skimmianine | 15 µM | MCF7 | 0 | -0.081 | -0.093 | 6242 |
| 4030 | 1049 | fulvestrant | 1 µM | MCF7 | 0 | -0.081 | -0.075 | 6872 |
| 4031 | 617 | chlorphenesin | 16 µM | PC3 | 0 | -0.081 | -0.062 | 2115 |
| 4032 | 613 | ampicillin | 10 µM | HL60 | 0 | -0.081 | -0.156 | 2030 |
| 4033 | 750 | tretinoin | 1 µM | HL60 | 0 | -0.081 | -0.137 | 6170 |
| 4034 | 1045 | 6-bromoindirubin-3'-oxime | 500 nM | MCF7 | 0 | -0.081 | -0.078 | 6589 |
| 4035 | 715 | hemicholinium | 7 µM | PC3 | 0 | -0.082 | -0.065 | 6739 |
| 4036 | 734 | alimemazine | 5 µM | PC3 | 0 | -0.082 | -0.066 | 5881 |
| 4037 | 686 | flecainide | 8 µM | MCF7 | 0 | -0.082 | -0.074 | 3843 |
| 4038 | 13 | nordihydroguaiaretic acid | 1 µM | MCF7 | 0 | -0.082 | -0.064 | 203 |
| 4039 | 741 | pivmecillinam | 8 µM | MCF7 | 0 | -0.082 | -0.062 | 6014 |
| 4040 | 504 | 5182598 | 25 µM | MCF7 | 0 | -0.082 | -0.081 | 868 |
| 4041 | 627 | triamterene | 16 µM | MCF7 | 0 | -0.082 | -0.068 | 1697 |
| 4042 | 753 | amphotericin B | 4 µM | PC3 | 0 | -0.082 | -0.055 | 6303 |
| 4043 | 35 | LY-294002 | 10 µM | HL60 | 0 | -0.082 | -0.095 | 361 |
| 4044 | 633 | lynestrenol | 14 µM | MCF7 | 0 | -0.082 | -0.1 | 1537 |
| 4045 | 19 | dexamethasone | 1 µM | MCF7 | 0 | -0.082 | -0.048 | 255 |
| 4046 | 660 | terguride | 12 µM | HL60 | 0 | -0.082 | -0.11 | 3082 |
| 4047 | 634 | vigabatrin | 31 µM | HL60 | 0 | -0.082 | -0.171 | 2452 |
| 4048 | 1014 | pioglitazone | 10 µM | MCF7 | 0 | -0.082 | -0.113 | 5972 |
| 4049 | 670 | levomepromazine | 9 µM | MCF7 | 0 | -0.082 | -0.081 | 3440 |
| 4050 | 649 | glycopyrronium bromide | 10 µM | HL60 | 0 | -0.082 | -0.12 | 2565 |
| 4051 | 691 | co-dergocrine mesilate | 6 µM | MCF7 | 0 | -0.082 | -0.087 | 4152 |
| 4052 | 767 | tanespimycin | 1 µM | MCF7 | 0 | -0.082 | -0.072 | 6943 |
| 4053 | 653 | suloctidil | 12 µM | MCF7 | 0 | -0.082 | -0.082 | 2651 |
| 4054 | 762 | lymecycline | 7 µM | PC3 | 0 | -0.082 | -0.06 | 7291 |
| 4055 | 610 | amiloride | 13 µM | PC3 | 0 | -0.082 | -0.073 | 1890 |
| 4056 | 703 | parbendazole | 16 µM | PC3 | 0 | -0.083 | -0.075 | 4535 |
| 4057 | 664 | Prestwick-984 | 9 µM | HL60 | 0 | -0.083 | -0.172 | 2903 |
| 4058 | 720 | spiradoline | 1 µM | MCF7 | 0 | -0.083 | -0.102 | 4375 |
| 4059 | 1079 | 6-bromoindirubin-3'-oxime | 500 nM | PC3 | 0 | -0.083 | -0.055 | 7106 |
| 4060 | 1004 | fulvestrant | 1 µM | MCF7 | 0 | -0.083 | -0.038 | 5926 |
| 4061 | 754 | doxepin | 13 µM | PC3 | 0 | -0.083 | -0.071 | 6337 |
| 4062 | 706 | methyldopate | 15 µM | MCF7 | 0 | -0.083 | -0.068 | 4986 |
| 4063 | 663 | yohimbic acid | 11 µM | MCF7 | 0 | -0.083 | -0.12 | 2803 |
| 4064 | 656 | lovastatin | 10 µM | MCF7 | 0 | -0.083 | -0.057 | 2854 |
| 4065 | 629 | naproxen | 17 µM | HL60 | 0 | -0.083 | -0.163 | 1869 |
| 4066 | 720 | lycorine | 12 µM | MCF7 | 0 | -0.083 | -0.112 | 4365 |
| 4067 | 703 | chlorcyclizine | 12 µM | PC3 | 0 | -0.083 | -0.047 | 4546 |
| 4068 | 710 | diloxanide | 12 µM | PC3 | 0 | -0.083 | -0.056 | 6679 |
| 4069 | 612 | miconazole | 10 µM | HL60 | 0 | -0.083 | -0.099 | 1977 |
| 4070 | 713 | loperamide | 8 µM | PC3 | 0 | -0.083 | -0.074 | 4672 |
| 4071 | 771 | aminoglutethimide | 17 µM | MCF7 | 0 | -0.083 | -0.075 | 7421 |
| 4072 | 683 | 2,6-dimethylpiperidine | 27 µM | PC3 | 0 | -0.083 | -0.056 | 3806 |
| 4073 | 644 | harman | 18 µM | HL60 | 0 | -0.083 | -0.185 | 2150 |
| 4074 | 1082 | CP-944629 | 10 µM | MCF7 | 0 | -0.083 | -0.078 | 7497 |
| 4075 | 683 | phenelzine | 17 µM | PC3 | 0 | -0.083 | -0.097 | 3802 |
| 4076 | 641 | aconitine | 6 µM | HL60 | 0 | -0.083 | -0.099 | 1784 |
| 4077 | 628 | todralazine | 15 µM | PC3 | 0 | -0.084 | -0.039 | 1799 |
| 4078 | 649 | alpha-ergocryptine | 7 µM | HL60 | 0 | -0.084 | -0.139 | 2572 |
| 4079 | 676 | apramycin | 7 µM | MCF7 | 0 | -0.084 | -0.078 | 7334 |
| 4080 | 634 | puromycin | 7 µM | HL60 | 0 | -0.084 | -0.101 | 2448 |
| 4081 | 762 | homochlorcyclizine | 10 µM | PC3 | 0 | -0.084 | -0.05 | 7295 |
| 4082 | 730 | ethaverine | 9 µM | MCF7 | 0 | -0.084 | -0.076 | 5337 |
| 4083 | 662 | quipazine | 9 µM | MCF7 | 0 | -0.084 | -0.099 | 2782 |
| 4084 | 622 | mifepristone | 9 µM | HL60 | 0 | -0.084 | -0.119 | 1569 |
| 4085 | 641 | folic acid | 9 µM | HL60 | 0 | -0.084 | -0.092 | 1790 |
| 4086 | 704 | ciclosporin | 3 µM | PC3 | 0 | -0.084 | -0.058 | 4586 |
| 4087 | 732 | spiperone | 10 µM | PC3 | 0 | -0.084 | -0.073 | 5777 |
| 4088 | 659 | nizatidine | 12 µM | HL60 | 0 | -0.084 | -0.083 | 3047 |
| 4089 | 633 | azacyclonol | 15 µM | MCF7 | 0 | -0.084 | -0.109 | 1520 |
| 4090 | 647 | alfuzosin | 9 µM | MCF7 | 0 | -0.084 | -0.083 | 3203 |
| 4091 | 728 | atropine oxide | 12 µM | PC3 | 0 | -0.084 | -0.051 | 4476 |
| 4092 | 39 | cobalt chloride | 100 µM | MCF7 | 0 | -0.084 | -0.106 | 379 |
| 4093 | 676 | gibberellic acid | 12 µM | MCF7 | 0 | -0.084 | -0.072 | 7330 |
| 4094 | 700 | gossypol | 8 µM | MCF7 | 0 | -0.085 | -0.086 | 4762 |
| 4095 | 656 | tiaprofenic acid | 15 µM | MCF7 | 0 | -0.085 | -0.122 | 2852 |
| 4096 | 767 | chlorpromazine | 1 µM | MCF7 | 0 | -0.085 | -0.072 | 6936 |
| 4097 | 758 | clofazimine | 8 µM | MCF7 | 0 | -0.085 | -0.073 | 5642 |
| 4098 | 661 | isometheptene | 8 µM | HL60 | 0 | -0.085 | -0.124 | 3145 |
| 4099 | 661 | Prestwick-983 | 17 µM | HL60 | 0 | -0.085 | -0.123 | 3141 |
| 4100 | 628 | homatropine | 11 µM | PC3 | 0 | -0.085 | -0.075 | 1806 |
| 4101 | 120 | estradiol | 10 nM | HL60 | 0 | -0.085 | -0.082 | 782 |
| 4102 | 741 | tiletamine | 15 µM | MCF7 | 0 | -0.085 | -0.071 | 6013 |
| 4103 | 750 | geldanamycin | 1 µM | HL60 | 0 | -0.085 | -0.12 | 6187 |
| 4104 | 622 | clotrimazole | 12 µM | HL60 | 0 | -0.085 | -0.172 | 1549 |
| 4105 | 661 | adipiodone | 4 µM | HL60 | 0 | -0.085 | -0.11 | 3111 |
| 4106 | 660 | butyl hydroxybenzoate | 21 µM | HL60 | 0 | -0.085 | -0.105 | 3069 |
| 4107 | 637 | mafenide | 18 µM | MCF7 | 0 | -0.085 | -0.105 | 2287 |
| 4108 | 681 | 6-benzylaminopurine | 18 µM | PC3 | 0 | -0.086 | -0.096 | 3726 |
| 4109 | 705 | lidocaine | 15 µM | MCF7 | 0 | -0.086 | -0.087 | 4421 |
| 4110 | 628 | sulfadiazine | 16 µM | PC3 | 0 | -0.086 | -0.068 | 1810 |
| 4111 | 662 | diclofenac | 13 µM | MCF7 | 0 | -0.086 | -0.123 | 2756 |
| 4112 | 744 | pindolol | 16 µM | MCF7 | 0 | -0.086 | -0.088 | 6834 |
| 4113 | 636 | ursolic acid | 9 µM | MCF7 | 0 | -0.086 | -0.064 | 2230 |
| 4114 | 747 | clindamycin | 9 µM | MCF7 | 0 | -0.086 | -0.09 | 7172 |
| 4115 | 628 | dexpanthenol | 19 µM | PC3 | 0 | -0.086 | -0.068 | 1802 |
| 4116 | 633 | dipyridamole | 8 µM | MCF7 | 0 | -0.086 | -0.052 | 1517 |
| 4117 | 632 | amylocaine | 15 µM | MCF7 | 0 | -0.086 | -0.085 | 1491 |
| 4118 | 610 | thioridazine | 10 µM | PC3 | 0 | -0.086 | -0.058 | 1905 |
| 4119 | 762 | tiletamine | 15 µM | PC3 | 0 | -0.086 | -0.075 | 7311 |
| 4120 | 642 | mexiletine | 19 µM | MCF7 | 0 | -0.086 | -0.056 | 2324 |
| 4121 | 653 | benfluorex | 10 µM | MCF7 | 0 | -0.086 | -0.069 | 2621 |
| 4122 | 627 | carbamazepine | 17 µM | MCF7 | 0 | -0.086 | -0.069 | 1683 |
| 4123 | 632 | pheniramine | 11 µM | MCF7 | 0 | -0.087 | -0.064 | 1492 |
| 4124 | 616 | gallamine triethiodide | 4 µM | PC3 | 0 | -0.087 | -0.039 | 2059 |
| 4125 | 635 | lovastatin | 10 µM | HL60 | 0 | -0.087 | -0.106 | 2494 |
| 4126 | 1011 | valinomycin | 100 nM | PC3 | 0 | -0.087 | -0.052 | 5962 |
| 4127 | 772 | piroxicam | 12 µM | MCF7 | 0 | -0.087 | -0.073 | 7445 |
| 4128 | 771 | lincomycin | 9 µM | MCF7 | 0 | -0.087 | -0.056 | 7411 |
| 4129 | 1065 | PHA-00846566E | 10 µM | PC3 | 0 | -0.087 | -0.056 | 7046 |
| 4130 | 1085 | BCB000039 | 10 µM | PC3 | 0 | -0.087 | -0.046 | 7510 |
| 4131 | 720 | tolbutamide | 15 µM | MCF7 | 0 | -0.087 | -0.071 | 4362 |
| 4132 | 630 | ketoconazole | 8 µM | HL60 | 0 | -0.087 | -0.111 | 1285 |
| 4133 | 693 | amprolium | 13 µM | PC3 | 0 | -0.088 | -0.066 | 4241 |
| 4134 | 706 | arcaine | 15 µM | MCF7 | 0 | -0.088 | -0.106 | 4974 |
| 4135 | 691 | trimethobenzamide | 9 µM | MCF7 | 0 | -0.088 | -0.108 | 4180 |
| 4136 | 648 | propidium iodide | 6 µM | HL60 | 0 | -0.088 | -0.126 | 2541 |
| 4137 | 738 | mefenamic acid | 17 µM | MCF7 | 0 | -0.088 | -0.061 | 5534 |
| 4138 | 758 | biperiden | 11 µM | MCF7 | 0 | -0.088 | -0.08 | 5644 |
| 4139 | 685 | betonicine | 25 µM | MCF7 | 0 | -0.088 | -0.127 | 3642 |
| 4140 | 700 | karakoline | 11 µM | MCF7 | 0 | -0.088 | -0.073 | 4763 |
| 4141 | 1065 | lomustine | 100 µM | PC3 | 0 | -0.088 | -0.106 | 7050 |
| 4142 | 648 | pheneticillin | 10 µM | HL60 | 0 | -0.088 | -0.147 | 2542 |
| 4143 | 711 | 0317956-0000 | 10 µM | MCF7 | 0 | -0.088 | -0.067 | 3966 |
| 4144 | 634 | melatonin | 17 µM | HL60 | 0 | -0.088 | -0.097 | 2431 |
| 4145 | 655 | dinoprost | 8 µM | MCF7 | 0 | -0.088 | -0.102 | 3308 |
| 4146 | 695 | tocainide | 17 µM | MCF7 | 0 | -0.088 | -0.066 | 4838 |
| 4147 | 677 | naftifine | 12 µM | MCF7 | 0 | -0.088 | -0.099 | 3536 |
| 4148 | 632 | khellin | 15 µM | MCF7 | 0 | -0.089 | -0.108 | 1504 |
| 4149 | 665 | asiaticoside | 4 µM | HL60 | 0 | -0.089 | -0.124 | 2943 |
| 4150 | 728 | isocorydine | 12 µM | PC3 | 0 | -0.089 | -0.08 | 4505 |
| 4151 | 699 | monensin | 6 µM | MCF7 | 0 | -0.089 | -0.076 | 4726 |
| 4152 | 772 | risperidone | 10 µM | MCF7 | 0 | -0.089 | -0.06 | 7449 |
| 4153 | 505 | wortmannin | 10 nM | MCF7 | 0 | -0.089 | -0.058 | 911 |
| 4154 | 661 | proxyphylline | 17 µM | HL60 | 0 | -0.089 | -0.116 | 3115 |
| 4155 | 767 | haloperidol | 10 µM | MCF7 | 0 | -0.089 | -0.055 | 6960 |
| 4156 | 1092 | 16-phenyltetranorprostaglandin E2 | 10 µM | MCF7 | 0 | -0.089 | -0.095 | 7541 |
| 4157 | 720 | paroxetine | 1 µM | MCF7 | 0 | -0.089 | -0.112 | 4378 |
| 4158 | 647 | clopamide | 12 µM | MCF7 | 0 | -0.089 | -0.094 | 3220 |
| 4159 | 727 | tanespimycin | 1 µM | PC3 | 0 | -0.089 | -0.077 | 4450 |
| 4160 | 727 | fulvestrant | 10 nM | PC3 | 0 | -0.089 | -0.06 | 4462 |
| 4161 | 629 | dapsone | 16 µM | HL60 | 0 | -0.089 | -0.143 | 1868 |
| 4162 | 617 | pargyline | 20 µM | PC3 | 0 | -0.089 | -0.046 | 2102 |
| 4163 | 735 | nitrendipine | 11 µM | MCF7 | 0 | -0.089 | -0.077 | 5405 |
| 4164 | 637 | ranitidine | 11 µM | MCF7 | 0 | -0.089 | -0.072 | 2251 |
| 4165 | 711 | vincamine | 11 µM | MCF7 | 0 | -0.089 | -0.095 | 3976 |
| 4166 | 688 | nadolol | 13 µM | PC3 | 0 | -0.089 | -0.094 | 4021 |
| 4167 | 640 | parthenolide | 16 µM | HL60 | 0 | -0.09 | -0.101 | 1736 |
| 4168 | 771 | isotretinoin | 13 µM | MCF7 | 0 | -0.09 | -0.091 | 7438 |
| 4169 | 735 | verapamil | 8 µM | MCF7 | 0 | -0.09 | -0.069 | 5387 |
| 4170 | 631 | dl-alpha tocopherol | 9 µM | HL60 | 0 | -0.09 | -0.189 | 1320 |
| 4171 | 619 | clemastine | 9 µM | HL60 | 0 | -0.09 | -0.137 | 2412 |
| 4172 | 1021 | 5707885 | 50 µM | PC3 | 0 | -0.09 | -0.078 | 6390 |
| 4173 | 755 | altretamine | 19 µM | MCF7 | 0 | -0.09 | -0.064 | 6467 |
| 4174 | 772 | clemastine | 9 µM | MCF7 | 0 | -0.09 | -0.055 | 7485 |
| 4175 | 636 | canrenoic acid | 10 µM | MCF7 | 0 | -0.09 | -0.094 | 2228 |
| 4176 | 645 | etiocholanolone | 14 µM | HL60 | 0 | -0.09 | -0.147 | 2204 |
| 4177 | 755 | timolol | 9 µM | MCF7 | 0 | -0.09 | -0.083 | 6483 |
| 4178 | 651 | pridinol | 10 µM | HL60 | 0 | -0.09 | -0.107 | 2715 |
| 4179 | 738 | sulfadiazine | 16 µM | MCF7 | 0 | -0.09 | -0.065 | 5523 |
| 4180 | 707 | metixene | 12 µM | MCF7 | 0 | -0.09 | -0.132 | 5018 |
| 4181 | 631 | carisoprodol | 15 µM | HL60 | 0 | -0.09 | -0.167 | 1314 |
| 4182 | 602 | sirolimus | 100 nM | HL60 | 0 | -0.09 | -0.16 | 1183 |
| 4183 | 683 | CP-320650-01 | 1 µM | PC3 | 0 | -0.09 | -0.066 | 3825 |
| 4184 | 641 | vinburnine | 14 µM | HL60 | 0 | -0.09 | -0.116 | 1788 |
| 4185 | 733 | doxycycline | 8 µM | PC3 | 0 | -0.091 | -0.09 | 5838 |
| 4186 | 502 | prazosin | 10 µM | MCF7 | 0 | -0.091 | -0.101 | 942 |
| 4187 | 630 | droperidol | 11 µM | HL60 | 0 | -0.091 | -0.128 | 1290 |
| 4188 | 636 | diazoxide | 17 µM | MCF7 | 0 | -0.091 | -0.074 | 2214 |
| 4189 | 650 | acetylsalicylic acid | 100 µM | HL60 | 0 | -0.091 | -0.093 | 2664 |
| 4190 | 665 | ozagrel | 15 µM | HL60 | 0 | -0.091 | -0.188 | 2942 |
| 4191 | 658 | iocetamic acid | 7 µM | HL60 | 0 | -0.091 | -0.122 | 3022 |
| 4192 | 754 | pinacidil | 16 µM | PC3 | 0 | -0.091 | -0.091 | 6356 |
| 4193 | 513 | LY-294002 | 10 µM | MCF7 | 0 | -0.091 | -0.059 | 1074 |
| 4194 | 660 | nilutamide | 13 µM | HL60 | 0 | -0.091 | -0.126 | 3104 |
| 4195 | 748 | ramifenazone | 14 µM | MCF7 | 0 | -0.091 | -0.126 | 7233 |
| 4196 | 746 | saquinavir | 5 µM | MCF7 | 0 | -0.091 | -0.055 | 6246 |
| 4197 | 1029 | F0447-0125 | 10 µM | PC3 | 0 | -0.091 | -0.066 | 6429 |
| 4198 | 79 | 15-delta prostaglandin J2 | 10 µM | SKMEL5 | 0 | -0.091 | -0.112 | 564 |
| 4199 | 665 | betulin | 9 µM | HL60 | 0 | -0.091 | -0.162 | 2952 |
| 4200 | 95 | monastrol | 100 µM | MCF7 | 0 | -0.091 | -0.06 | 596 |
| 4201 | 1093 | semustine | 100 µM | PC3 | 0 | -0.092 | -0.121 | 7545 |
| 4202 | 699 | calcium folinate | 8 µM | MCF7 | 0 | -0.092 | -0.066 | 4725 |
| 4203 | 747 | nalbuphine | 10 µM | MCF7 | 0 | -0.092 | -0.109 | 7177 |
| 4204 | 647 | metitepine | 8 µM | MCF7 | 0 | -0.092 | -0.073 | 3231 |
| 4205 | 640 | prenylamine | 10 µM | HL60 | 0 | -0.092 | -0.089 | 1737 |
| 4206 | 1001 | valinomycin | 100 nM | PC3 | 0 | -0.092 | -0.102 | 5911 |
| 4207 | 626 | alvespimycin | 100 nM | MCF7 | 0 | -0.092 | -0.064 | 1638 |
| 4208 | 504 | 5230742 | 17 µM | MCF7 | 0 | -0.092 | -0.053 | 862 |
| 4209 | 660 | idazoxan | 17 µM | HL60 | 0 | -0.092 | -0.084 | 3088 |
| 4210 | 631 | chlorogenic acid | 11 µM | HL60 | 0 | -0.092 | -0.197 | 1346 |
| 4211 | 746 | cinoxacin | 15 µM | MCF7 | 0 | -0.092 | -0.085 | 6257 |
| 4212 | 683 | santonin | 16 µM | PC3 | 0 | -0.092 | -0.084 | 3795 |
| 4213 | 714 | desipramine | 13 µM | PC3 | 0 | -0.092 | -0.075 | 6693 |
| 4214 | 619 | nortriptyline | 13 µM | HL60 | 0 | -0.093 | -0.142 | 2391 |
| 4215 | 618 | clemizole | 11 µM | HL60 | 0 | -0.093 | -0.134 | 2339 |
| 4216 | 694 | diflunisal | 16 µM | MCF7 | 0 | -0.093 | -0.071 | 4794 |
| 4217 | 505 | decitabine | 100 nM | MCF7 | 0 | -0.093 | -0.053 | 920 |
| 4218 | 676 | sitosterol | 10 µM | MCF7 | 0 | -0.093 | -0.067 | 7332 |
| 4219 | 635 | nystatin | 4 µM | HL60 | 0 | -0.093 | -0.144 | 2500 |
| 4220 | 636 | midodrine | 14 µM | MCF7 | 0 | -0.093 | -0.071 | 2250 |
| 4221 | 513 | estradiol | 10 nM | MCF7 | 0 | -0.093 | -0.069 | 1079 |
| 4222 | 634 | glycocholic acid | 9 µM | HL60 | 0 | -0.093 | -0.098 | 2454 |
| 4223 | 663 | gelsemine | 12 µM | MCF7 | 0 | -0.093 | -0.051 | 2817 |
| 4224 | 661 | atropine methonitrate | 11 µM | HL60 | 0 | -0.093 | -0.106 | 3116 |
| 4225 | 729 | moroxydine | 19 µM | MCF7 | 0 | -0.093 | -0.095 | 5304 |
| 4226 | 726 | timolol | 9 µM | MCF7 | 0 | -0.093 | -0.09 | 5280 |
| 4227 | 700 | diphenylpyraline | 13 µM | MCF7 | 0 | -0.093 | -0.08 | 4765 |
| 4228 | 705 | captopril | 17 µM | MCF7 | 0 | -0.093 | -0.101 | 4410 |
| 4229 | 613 | hydroxyzine | 9 µM | HL60 | 0 | -0.093 | -0.112 | 2024 |
| 4230 | 750 | sirolimus | 100 nM | HL60 | 0 | -0.093 | -0.124 | 6167 |
| 4231 | 650 | trifluoperazine | 10 µM | HL60 | 0 | -0.093 | -0.092 | 2684 |
| 4232 | 749 | Prestwick-1085 | 15 µM | HL60 | 0 | -0.093 | -0.14 | 6131 |
| 4233 | 746 | ondansetron | 12 µM | MCF7 | 0 | -0.093 | -0.062 | 6270 |
| 4234 | 676 | 3-acetamidocoumarin | 20 µM | MCF7 | 0 | -0.093 | -0.095 | 7361 |
| 4235 | 655 | ceforanide | 8 µM | MCF7 | 0 | -0.093 | -0.105 | 3309 |
| 4236 | 765 | wortmannin | 10 nM | MCF7 | 0 | -0.094 | -0.069 | 7002 |
| 4237 | 732 | tretinoin | 13 µM | PC3 | 0 | -0.094 | -0.093 | 5767 |
| 4238 | 645 | methylbenzethonium chloride | 9 µM | HL60 | 0 | -0.094 | -0.115 | 2190 |
| 4239 | 627 | acetohexamide | 12 µM | MCF7 | 0 | -0.094 | -0.102 | 1707 |
| 4240 | 766 | dihydroergocristine | 6 µM | MCF7 | 0 | -0.094 | -0.071 | 7034 |
| 4241 | 730 | desoxycortone | 12 µM | MCF7 | 0 | -0.094 | -0.066 | 5357 |
| 4242 | 730 | zaprinast | 15 µM | MCF7 | 0 | -0.094 | -0.065 | 5349 |
| 4243 | 656 | methoxamine | 16 µM | MCF7 | 0 | -0.094 | -0.071 | 2848 |
| 4244 | 731 | raloxifene | 8 µM | PC3 | 0 | -0.094 | -0.058 | 5759 |
| 4245 | 627 | heptaminol | 22 µM | MCF7 | 0 | -0.094 | -0.069 | 1703 |
| 4246 | 677 | torasemide | 11 µM | MCF7 | 0 | -0.094 | -0.069 | 3517 |
| 4247 | 685 | diphenylpyraline | 13 µM | MCF7 | 0 | -0.094 | -0.096 | 3640 |
| 4248 | 733 | terazosin | 9 µM | PC3 | 0 | -0.095 | -0.09 | 5831 |
| 4249 | 655 | cephaeline | 6 µM | MCF7 | 0 | -0.095 | -0.1 | 3290 |
| 4250 | 1088 | suramin sodium | 10 µM | MCF7 | 0 | -0.095 | -0.093 | 7524 |
| 4251 | 1076 | lomustine | 100 µM | MCF7 | 0 | -0.095 | -0.042 | 7089 |
| 4252 | 764 | isopropamide iodide | 8 µM | PC3 | 0 | -0.095 | -0.097 | 7133 |
| 4253 | 765 | estradiol | 10 nM | MCF7 | 0 | -0.095 | -0.107 | 7000 |
| 4254 | 766 | adiphenine | 11 µM | MCF7 | 0 | -0.095 | -0.048 | 7037 |
| 4255 | 728 | piretanide | 11 µM | PC3 | 0 | -0.095 | -0.046 | 4490 |
| 4256 | 704 | lidocaine | 15 µM | PC3 | 0 | -0.095 | -0.059 | 4596 |
| 4257 | 681 | Prestwick-664 | 8 µM | PC3 | 0 | -0.095 | -0.066 | 3715 |
| 4258 | 703 | nialamide | 13 µM | PC3 | 0 | -0.096 | -0.069 | 4525 |
| 4259 | 627 | metformin | 24 µM | MCF7 | 0 | -0.096 | -0.054 | 1694 |
| 4260 | 650 | monorden | 100 nM | HL60 | 0 | -0.096 | -0.153 | 2679 |
| 4261 | 658 | iodixanol | 3 µM | HL60 | 0 | -0.096 | -0.151 | 3023 |
| 4262 | 657 | ajmaline | 12 µM | MCF7 | 0 | -0.096 | -0.107 | 2899 |
| 4263 | 688 | niclosamide | 12 µM | PC3 | 0 | -0.096 | -0.086 | 4018 |
| 4264 | 661 | halofantrine | 7 µM | HL60 | 0 | -0.096 | -0.14 | 3130 |
| 4265 | 1011 | estradiol | 10 nM | PC3 | 0 | -0.096 | -0.075 | 5960 |
| 4266 | 688 | brompheniramine | 9 µM | PC3 | 0 | -0.096 | -0.052 | 4013 |
| 4267 | 640 | sulfasalazine | 10 µM | HL60 | 0 | -0.096 | -0.094 | 1733 |
| 4268 | 1010 | estradiol | 10 nM | MCF7 | 0 | -0.097 | -0.11 | 5955 |
| 4269 | 634 | dinoprost | 8 µM | HL60 | 0 | -0.097 | -0.189 | 2446 |
| 4270 | 700 | heliotrine | 13 µM | MCF7 | 0 | -0.097 | -0.087 | 4739 |
| 4271 | 33 | valproic acid | 50 µM | MCF7 | 0 | -0.097 | -0.064 | 348 |
| 4272 | 641 | demecarium bromide | 6 µM | HL60 | 0 | -0.097 | -0.126 | 1781 |
| 4273 | 603 | chlorpromazine | 1 µM | PC3 | 0 | -0.097 | -0.075 | 1217 |
| 4274 | 659 | primidone | 18 µM | HL60 | 0 | -0.097 | -0.121 | 3065 |
| 4275 | 702 | helveticoside | 7 µM | PC3 | 0 | -0.097 | -0.094 | 4327 |
| 4276 | 700 | PNU-0230031 | 1 µM | MCF7 | 0 | -0.097 | -0.083 | 4757 |
| 4277 | 766 | thiamphenicol | 11 µM | MCF7 | 0 | -0.097 | -0.077 | 7033 |
| 4278 | 682 | acacetin | 14 µM | PC3 | 0 | -0.097 | -0.053 | 3767 |
| 4279 | 634 | hexetidine | 12 µM | HL60 | 0 | -0.097 | -0.15 | 2457 |
| 4280 | 683 | pramocaine | 12 µM | PC3 | 0 | -0.097 | -0.049 | 3811 |
| 4281 | 699 | guanadrel | 8 µM | MCF7 | 0 | -0.097 | -0.145 | 4720 |
| 4282 | 66 | monorden | 100 nM | PC3 | 0 | -0.097 | -0.071 | 484 |
| 4283 | 31 | fasudil | 10 µM | MCF7 | 0 | -0.098 | -0.104 | 343 |
| 4284 | 610 | diflunisal | 16 µM | PC3 | 0 | -0.098 | -0.054 | 1908 |
| 4285 | 701 | PNU-0230031 | 1 µM | PC3 | 0 | -0.098 | -0.085 | 4291 |
| 4286 | 1068 | 0198306-0000 | 10 µM | MCF7 | 0 | -0.098 | -0.081 | 7064 |
| 4287 | 676 | decamethonium bromide | 10 µM | MCF7 | 0 | -0.098 | -0.093 | 7353 |
| 4288 | 705 | vancomycin | 3 µM | MCF7 | 0 | -0.098 | -0.062 | 4423 |
| 4289 | 634 | pirenperone | 10 µM | HL60 | 0 | -0.098 | -0.09 | 2455 |
| 4290 | 676 | methyldopate | 15 µM | MCF7 | 0 | -0.098 | -0.119 | 7360 |
| 4291 | 731 | pheneticillin | 10 µM | PC3 | 0 | -0.098 | -0.087 | 5763 |
| 4292 | 703 | withaferin A | 1 µM | PC3 | 0 | -0.098 | -0.059 | 4554 |
| 4293 | 688 | gramine | 23 µM | PC3 | 0 | -0.098 | -0.05 | 3999 |
| 4294 | 747 | hecogenin | 9 µM | MCF7 | 0 | -0.098 | -0.114 | 7175 |
| 4295 | 771 | ifenprodil | 8 µM | MCF7 | 0 | -0.098 | -0.055 | 7404 |
| 4296 | 1068 | MG-262 | 100 nM | MCF7 | 0 | -0.098 | -0.165 | 7063 |
| 4297 | 727 | tanespimycin | 1 µM | PC3 | 0 | -0.098 | -0.049 | 4449 |
| 4298 | 665 | pivmecillinam | 8 µM | HL60 | 0 | -0.099 | -0.111 | 2973 |
| 4299 | 665 | quinpirole | 16 µM | HL60 | 0 | -0.099 | -0.168 | 2977 |
| 4300 | 640 | dihydroergocristine | 6 µM | HL60 | 0 | -0.099 | -0.104 | 1745 |
| 4301 | 771 | trazodone | 10 µM | MCF7 | 0 | -0.099 | -0.075 | 7410 |
| 4302 | 664 | alprostadil | 11 µM | HL60 | 0 | -0.099 | -0.131 | 2938 |
| 4303 | 709 | methoxamine | 16 µM | PC3 | 0 | -0.099 | -0.08 | 6627 |
| 4304 | 704 | sulpiride | 12 µM | PC3 | 0 | -0.099 | -0.082 | 4566 |
| 4305 | 705 | 3-acetamidocoumarin | 20 µM | MCF7 | 0 | -0.099 | -0.105 | 4426 |
| 4306 | 685 | gossypol | 8 µM | MCF7 | 0 | -0.1 | -0.121 | 3637 |
| 4307 | 628 | meticrane | 15 µM | PC3 | 0 | -0.1 | -0.044 | 1792 |
| 4308 | 676 | fluticasone | 8 µM | MCF7 | 0 | -0.1 | -0.074 | 7348 |
| 4309 | 731 | fluorocurarine | 12 µM | PC3 | 0 | -0.1 | -0.065 | 5741 |
| 4310 | 658 | etidronic acid | 16 µM | HL60 | 0 | -0.1 | -0.065 | 2985 |
| 4311 | 641 | noscapine | 10 µM | HL60 | 0 | -0.1 | -0.106 | 1753 |
| 4312 | 619 | homochlorcyclizine | 10 µM | HL60 | 0 | -0.1 | -0.164 | 2386 |
| 4313 | 633 | diltiazem | 9 µM | MCF7 | 0 | -0.1 | -0.082 | 1532 |
| 4314 | 747 | isoniazid | 29 µM | MCF7 | 0 | -0.1 | -0.08 | 7197 |
| 4315 | 765 | LY-294002 | 10 µM | MCF7 | 0 | -0.101 | -0.076 | 6987 |
| 4316 | 736 | molsidomine | 17 µM | MCF7 | 0 | -0.101 | -0.088 | 5426 |
| 4317 | 1045 | dinoprostone | 10 µM | MCF7 | 0 | -0.101 | -0.082 | 6590 |
| 4318 | 640 | tetracaine | 13 µM | HL60 | 0 | -0.101 | -0.121 | 1739 |
| 4319 | 661 | enilconazole | 13 µM | HL60 | 0 | -0.101 | -0.083 | 3139 |
| 4320 | 727 | nordihydroguaiaretic acid | 1 µM | PC3 | 0 | -0.101 | -0.075 | 4447 |
| 4321 | 640 | trimetazidine | 12 µM | HL60 | 0 | -0.101 | -0.11 | 1727 |
| 4322 | 630 | anisomycin | 15 µM | HL60 | 0 | -0.102 | -0.12 | 1304 |
| 4323 | 683 | citalopram | 1 µM | PC3 | 0 | -0.102 | -0.08 | 3820 |
| 4324 | 1065 | 6-bromoindirubin-3'-oxime | 500 nM | PC3 | 0 | -0.102 | -0.042 | 7048 |
| 4325 | 641 | quipazine | 9 µM | HL60 | 0 | -0.102 | -0.115 | 1789 |
| 4326 | 771 | telenzepine | 9 µM | MCF7 | 0 | -0.102 | -0.072 | 7419 |
| 4327 | 757 | genistein | 10 µM | MCF7 | 0 | -0.102 | -0.096 | 5595 |
| 4328 | 665 | lansoprazole | 11 µM | HL60 | 0 | -0.102 | -0.165 | 2967 |
| 4329 | 663 | benzocaine | 24 µM | MCF7 | 0 | -0.102 | -0.134 | 2822 |
| 4330 | 1069 | 0198306-0000 | 10 µM | PC3 | 0 | -0.102 | -0.061 | 7069 |
| 4331 | 630 | clozapine | 12 µM | HL60 | 0 | -0.102 | -0.149 | 1289 |
| 4332 | 647 | brinzolamide | 10 µM | MCF7 | 0 | -0.102 | -0.111 | 3230 |
| 4333 | 505 | 5140203 | 15 µM | MCF7 | 0 | -0.102 | -0.081 | 908 |
| 4334 | 647 | metergoline | 10 µM | MCF7 | 0 | -0.102 | -0.09 | 3221 |
| 4335 | 661 | flucloxacillin | 8 µM | HL60 | 0 | -0.102 | -0.19 | 3128 |
| 4336 | 1009 | monorden | 100 nM | PC3 | 0 | -0.102 | -0.11 | 5952 |
| 4337 | 650 | LY-294002 | 10 µM | HL60 | 0 | -0.102 | -0.103 | 2699 |
| 4338 | 714 | dexibuprofen | 19 µM | PC3 | 0 | -0.103 | -0.048 | 6712 |
| 4339 | 659 | 2-aminobenzenesulfonamide | 23 µM | HL60 | 0 | -0.103 | -0.071 | 3063 |
| 4340 | 733 | piperacetazine | 10 µM | PC3 | 0 | -0.103 | -0.057 | 5834 |
| 4341 | 705 | Prestwick-692 | 7 µM | MCF7 | 0 | -0.103 | -0.096 | 4424 |
| 4342 | 514 | tyrphostin AG-1478 | 32 µM | MCF7 | 0 | -0.103 | -0.063 | 1141 |
| 4343 | 640 | lobelanidine | 11 µM | HL60 | 0 | -0.103 | -0.145 | 1747 |
| 4344 | 633 | ampicillin | 10 µM | MCF7 | 0 | -0.103 | -0.091 | 1530 |
| 4345 | 631 | mebhydrolin | 5 µM | HL60 | 0 | -0.103 | -0.157 | 1333 |
| 4346 | 701 | 0179445-0000 | 10 µM | PC3 | 0 | -0.103 | -0.078 | 4289 |
| 4347 | 634 | nitrofural | 20 µM | HL60 | 0 | -0.103 | -0.148 | 2459 |
| 4348 | 658 | procyclidine | 12 µM | HL60 | 0 | -0.103 | -0.113 | 2991 |
| 4349 | 678 | oxyphenbutazone | 12 µM | MCF7 | 0 | -0.104 | -0.092 | 3582 |
| 4350 | 602 | acetylsalicylic acid | 100 µM | HL60 | 0 | -0.104 | -0.111 | 1145 |
| 4351 | 672 | spectinomycin | 10 µM | MCF7 | 0 | -0.104 | -0.069 | 3327 |
| 4352 | 665 | phensuximide | 21 µM | HL60 | 0 | -0.104 | -0.153 | 2960 |
| 4353 | 665 | levopropoxyphene | 7 µM | HL60 | 0 | -0.104 | -0.178 | 2980 |
| 4354 | 635 | pregnenolone | 13 µM | HL60 | 0 | -0.104 | -0.175 | 2497 |
| 4355 | 741 | harmol | 16 µM | MCF7 | 0 | -0.104 | -0.063 | 6022 |
| 4356 | 713 | cephaeline | 6 µM | PC3 | 0 | -0.105 | -0.087 | 4651 |
| 4357 | 661 | rilmenidine | 8 µM | HL60 | 0 | -0.105 | -0.157 | 3133 |
| 4358 | 630 | domperidone | 7 µM | HL60 | 0 | -0.105 | -0.11 | 1301 |
| 4359 | 648 | alclometasone | 8 µM | HL60 | 0 | -0.105 | -0.109 | 2532 |
| 4360 | 658 | trimipramine | 10 µM | HL60 | 0 | -0.105 | -0.071 | 3004 |
| 4361 | 683 | lycorine | 12 µM | PC3 | 0 | -0.106 | -0.052 | 3808 |
| 4362 | 629 | metformin | 24 µM | HL60 | 0 | -0.106 | -0.209 | 1858 |
| 4363 | 733 | mifepristone | 9 µM | PC3 | 0 | -0.106 | -0.053 | 5827 |
| 4364 | 708 | vanoxerine | 8 µM | MCF7 | 0 | -0.106 | -0.073 | 5702 |
| 4365 | 73 | tanespimycin | 1 µM | SKMEL5 | 0 | -0.106 | -0.077 | 505 |
| 4366 | 749 | sertaconazole | 8 µM | HL60 | 0 | -0.107 | -0.132 | 6128 |
| 4367 | 629 | acetohexamide | 12 µM | HL60 | 0 | -0.107 | -0.123 | 1870 |
| 4368 | 678 | mesalazine | 26 µM | MCF7 | 0 | -0.107 | -0.102 | 3584 |
| 4369 | 678 | propoxycaine | 12 µM | MCF7 | 0 | -0.107 | -0.071 | 3583 |
| 4370 | 1029 | STOCK1N-35215 | 10 µM | PC3 | 0 | -0.107 | -0.055 | 6427 |
| 4371 | 635 | tiaprofenic acid | 15 µM | HL60 | 0 | -0.107 | -0.133 | 2492 |
| 4372 | 634 | cephaeline | 6 µM | HL60 | 0 | -0.107 | -0.125 | 2429 |
| 4373 | 644 | quinisocaine | 13 µM | HL60 | 0 | -0.107 | -0.113 | 2151 |
| 4374 | 714 | primidone | 18 µM | PC3 | 0 | -0.107 | -0.083 | 6723 |
| 4375 | 749 | methanthelinium bromide | 10 µM | HL60 | 0 | -0.108 | -0.165 | 6137 |
| 4376 | 765 | genistein | 10 µM | MCF7 | 0 | -0.108 | -0.054 | 6994 |
| 4377 | 602 | rosiglitazone | 10 µM | HL60 | 0 | -0.108 | -0.132 | 1174 |
| 4378 | 626 | estradiol | 10 nM | MCF7 | 0 | -0.108 | -0.118 | 1666 |
| 4379 | 1011 | tanespimycin | 1 µM | PC3 | 0 | -0.109 | -0.108 | 5958 |
| 4380 | 698 | glycopyrronium bromide | 10 µM | PC3 | 0 | -0.109 | -0.061 | 7386 |
| 4381 | 1079 | 0198306-0000 | 10 µM | PC3 | 0 | -0.109 | -0.048 | 7102 |
| 4382 | 645 | lycorine | 12 µM | HL60 | 0 | -0.109 | -0.133 | 2195 |
| 4383 | 771 | clemastine | 9 µM | MCF7 | 0 | -0.109 | -0.086 | 7443 |
| 4384 | 765 | tanespimycin | 1 µM | MCF7 | 0 | -0.109 | -0.12 | 6986 |
| 4385 | 720 | thiamazole | 35 µM | MCF7 | 0 | -0.109 | -0.096 | 4372 |
| 4386 | 703 | tinidazole | 16 µM | PC3 | 0 | -0.109 | -0.072 | 4548 |
| 4387 | 29 | felodipine | 10 µM | MCF7 | 0 | -0.11 | -0.07 | 337 |
| 4388 | 744 | pipenzolate bromide | 9 µM | MCF7 | 0 | -0.11 | -0.07 | 6821 |
| 4389 | 736 | tubocurarine chloride | 5 µM | MCF7 | 0 | -0.11 | -0.081 | 5449 |
| 4390 | 622 | alprenolol | 14 µM | HL60 | 0 | -0.11 | -0.113 | 1571 |
| 4391 | 698 | pentolonium | 7 µM | PC3 | 0 | -0.11 | -0.067 | 7375 |
| 4392 | 657 | dirithromycin | 5 µM | MCF7 | 0 | -0.11 | -0.098 | 2863 |
| 4393 | 707 | methoxsalen | 19 µM | MCF7 | 0 | -0.11 | -0.071 | 5007 |
| 4394 | 764 | ronidazole | 20 µM | PC3 | 0 | -0.11 | -0.111 | 7131 |
| 4395 | 648 | benzethonium chloride | 9 µM | HL60 | 0 | -0.111 | -0.138 | 2508 |
| 4396 | 665 | penbutolol | 6 µM | HL60 | 0 | -0.111 | -0.163 | 2972 |
| 4397 | 651 | vidarabine | 15 µM | HL60 | 0 | -0.111 | -0.115 | 2706 |
| 4398 | 657 | clorsulon | 11 µM | MCF7 | 0 | -0.111 | -0.114 | 2884 |
| 4399 | 6 | chlorpropamide | 100 µM | MCF7 | 0 | -0.112 | -0.094 | 141 |
| 4400 | 21 | genistein | 1 µM | MCF7 | 0 | -0.112 | -0.056 | 268 |
| 4401 | 615 | tolfenamic acid | 15 µM | HL60 | 0 | -0.112 | -0.143 | 1437 |
| 4402 | 655 | epiandrosterone | 14 µM | MCF7 | 0 | -0.112 | -0.162 | 3306 |
| 4403 | 635 | tenoxicam | 12 µM | HL60 | 0 | -0.112 | -0.174 | 2501 |
| 4404 | 602 | trifluoperazine | 10 µM | HL60 | 0 | -0.113 | -0.134 | 1165 |
| 4405 | 658 | Prestwick-864 | 35 µM | HL60 | 0 | -0.113 | -0.076 | 2994 |
| 4406 | 617 | nimesulide | 13 µM | PC3 | 0 | -0.114 | -0.049 | 2112 |
| 4407 | 720 | withaferin A | 1 µM | MCF7 | 0 | -0.114 | -0.086 | 4376 |
| 4408 | 749 | piretanide | 11 µM | HL60 | 0 | -0.114 | -0.084 | 6144 |
| 4409 | 631 | betulinic acid | 9 µM | HL60 | 0 | -0.114 | -0.116 | 1345 |
| 4410 | 750 | LY-294002 | 10 µM | HL60 | 0 | -0.115 | -0.066 | 6198 |
| 4411 | 655 | dicloxacillin | 8 µM | MCF7 | 0 | -0.115 | -0.08 | 3307 |
| 4412 | 663 | emetine | 7 µM | MCF7 | 0 | -0.116 | -0.111 | 2801 |
| 4413 | 627 | dapsone | 16 µM | MCF7 | 0 | -0.116 | -0.101 | 1705 |
| 4414 | 631 | meclocycline | 6 µM | HL60 | 0 | -0.116 | -0.158 | 1341 |
| 4415 | 617 | etofylline | 18 µM | PC3 | 0 | -0.116 | -0.071 | 2093 |
| 4416 | 705 | naringenin | 15 µM | MCF7 | 0 | -0.117 | -0.126 | 4422 |
| 4417 | 627 | homatropine | 11 µM | MCF7 | 0 | -0.117 | -0.085 | 1684 |
| 4418 | 613 | azathioprine | 14 µM | HL60 | 0 | -0.117 | -0.126 | 2028 |
| 4419 | 692 | spectinomycin | 10 µM | PC3 | 0 | -0.117 | -0.083 | 4187 |
| 4420 | 746 | lidoflazine | 8 µM | MCF7 | 0 | -0.117 | -0.072 | 6278 |
| 4421 | 772 | thiamine | 12 µM | MCF7 | 0 | -0.118 | -0.095 | 7479 |
| 4422 | 686 | 0317956-0000 | 10 µM | MCF7 | 0 | -0.118 | -0.071 | 3855 |
| 4423 | 629 | diprophylline | 16 µM | HL60 | 0 | -0.118 | -0.129 | 1853 |
| 4424 | 630 | estradiol | 15 µM | HL60 | 0 | -0.118 | -0.113 | 1299 |
| 4425 | 60 | 15-delta prostaglandin J2 | 10 µM | PC3 | 0 | -0.118 | -0.106 | 446 |
| 4426 | 656 | probenecid | 14 µM | MCF7 | 0 | -0.119 | -0.069 | 2825 |
| 4427 | 747 | iopamidol | 5 µM | MCF7 | 0 | -0.119 | -0.063 | 7189 |
| 4428 | 683 | PHA-00745360 | 1 µM | PC3 | 0 | -0.119 | -0.141 | 3827 |
| 4429 | 683 | alpha-ergocryptine | 7 µM | PC3 | 0 | -0.12 | -0.106 | 3817 |
| 4430 | 765 | 15-delta prostaglandin J2 | 10 µM | MCF7 | 0 | -0.12 | -0.09 | 6990 |
| 4431 | 687 | pramocaine | 12 µM | MCF7 | 0 | -0.12 | -0.077 | 3894 |
| 4432 | 664 | cyclopenthiazide | 11 µM | HL60 | 0 | -0.12 | -0.16 | 2905 |
| 4433 | 676 | zalcitabine | 19 µM | MCF7 | 0 | -0.12 | -0.061 | 7352 |
| 4434 | 1015 | pioglitazone | 10 µM | PC3 | 0 | -0.121 | -0.064 | 5977 |
| 4435 | 1069 | SC-19220 | 10 µM | PC3 | 0 | -0.121 | -0.059 | 7065 |
| 4436 | 727 | LY-294002 | 10 µM | PC3 | 0 | -0.121 | -0.067 | 4463 |
| 4437 | 752 | alclometasone | 8 µM | MCF7 | 0 | -0.121 | -0.091 | 6094 |
| 4438 | 692 | canavanine | 14 µM | PC3 | 0 | -0.121 | -0.053 | 4197 |
| 4439 | 710 | (-)-MK-801 | 12 µM | PC3 | 0 | -0.122 | -0.065 | 6657 |
| 4440 | 658 | tolmetin | 13 µM | HL60 | 0 | -0.122 | -0.093 | 3009 |
| 4441 | 629 | isoflupredone | 10 µM | HL60 | 0 | -0.123 | -0.167 | 1873 |
| 4442 | 686 | 0317956-0000 | 1 µM | MCF7 | 0 | -0.123 | -0.116 | 3858 |
| 4443 | 656 | cefaclor | 10 µM | MCF7 | 0 | -0.123 | -0.071 | 2843 |
| 4444 | 720 | CP-320650-01 | 1 µM | MCF7 | 0 | -0.124 | -0.094 | 4382 |
| 4445 | 653 | anisomycin | 15 µM | MCF7 | 0 | -0.125 | -0.109 | 2658 |
| 4446 | 513 | LY-294002 | 10 µM | MCF7 | 0 | -0.125 | -0.036 | 1065 |
| 4447 | 766 | etodolac | 14 µM | MCF7 | 0 | -0.126 | -0.072 | 7006 |
| 4448 | 661 | ribavirin | 16 µM | HL60 | 0 | -0.126 | -0.078 | 3142 |
| 4449 | 772 | penbutolol | 6 µM | MCF7 | 0 | -0.126 | -0.087 | 7476 |
| 4450 | 713 | haloperidol | 11 µM | PC3 | 0 | -0.126 | -0.081 | 4678 |
| 4451 | 659 | eticlopride | 11 µM | HL60 | 0 | -0.127 | -0.112 | 3056 |
| 4452 | 772 | acetohexamide | 12 µM | MCF7 | 0 | -0.127 | -0.071 | 7482 |
| 4453 | 655 | biperiden | 11 µM | MCF7 | 0 | -0.127 | -0.096 | 3321 |
| 4454 | 658 | chloropyramine | 12 µM | HL60 | 0 | -0.127 | -0.101 | 3011 |
| 4455 | 631 | buflomedil | 12 µM | HL60 | 0 | -0.128 | -0.127 | 1338 |
| 4456 | 626 | LY-294002 | 10 µM | MCF7 | 0 | -0.128 | -0.055 | 1661 |
| 4457 | 731 | disulfiram | 13 µM | PC3 | 0 | -0.128 | -0.115 | 5729 |
| 4458 | 663 | Prestwick-682 | 6 µM | MCF7 | 0 | -0.128 | -0.088 | 2819 |
| 4459 | 725 | 15-delta prostaglandin J2 | 10 µM | MCF7 | 0 | -0.129 | -0.112 | 5228 |
| 4460 | 676 | cefalonium | 9 µM | MCF7 | 0 | -0.129 | -0.107 | 7341 |
| 4461 | 720 | CP-690334-01 | 10 µM | MCF7 | 0 | -0.129 | -0.1 | 4380 |
| 4462 | 1010 | tanespimycin | 1 µM | MCF7 | 0 | -0.13 | -0.128 | 5953 |
| 4463 | 632 | sulfathiazole | 16 µM | MCF7 | 0 | -0.13 | -0.06 | 1463 |
| 4464 | 683 | withaferin A | 1 µM | PC3 | 0 | -0.131 | -0.095 | 3819 |
| 4465 | 735 | clopamide | 12 µM | MCF7 | 0 | -0.131 | -0.088 | 5402 |
| 4466 | 632 | naphazoline | 16 µM | MCF7 | 0 | -0.132 | -0.069 | 1466 |
| 4467 | 648 | suxibuzone | 9 µM | HL60 | 0 | -0.132 | -0.073 | 2503 |
| 4468 | 633 | lisuride | 12 µM | MCF7 | 0 | -0.133 | -0.091 | 1545 |
| 4469 | 744 | securinine | 18 µM | MCF7 | 0 | -0.133 | -0.054 | 6831 |
| 4470 | 650 | troglitazone | 10 µM | HL60 | 0 | -0.134 | -0.091 | 2692 |
| 4471 | 693 | fosfosal | 18 µM | PC3 | 0 | -0.135 | -0.078 | 4239 |
| 4472 | 720 | PHA-00745360 | 10 µM | MCF7 | 0 | -0.136 | -0.077 | 4381 |
| 4473 | 504 | geldanamycin | 1 µM | MCF7 | 0 | -0.137 | -0.052 | 864 |
| 4474 | 506 | LY-294002 | 10 µM | MCF7 | 0 | -0.139 | -0.082 | 1016 |
| 4475 | 1079 | thapsigargin | 100 nM | PC3 | 0 | -0.142 | -0.152 | 7103 |
| 4476 | 645 | benzamil | 11 µM | HL60 | 0 | -0.142 | -0.136 | 2200 |
| 4477 | 658 | lasalocid | 7 µM | HL60 | 0 | -0.142 | -0.072 | 3021 |
| 4478 | 671 | isoetarine | 12 µM | MCF7 | 0 | -0.143 | -0.11 | 3451 |
| 4479 | 658 | benzthiazide | 9 µM | HL60 | 0 | -0.144 | -0.056 | 2989 |
| 4480 | 658 | tetroquinone | 21 µM | HL60 | 0 | -0.145 | -0.186 | 2999 |
| 4481 | 650 | tanespimycin | 1 µM | HL60 | 0 | -0.145 | -0.087 | 2685 |
| 4482 | 705 | ciclosporin | 3 µM | MCF7 | 0 | -0.147 | -0.06 | 4411 |
| 4483 | 663 | Prestwick-692 | 7 µM | MCF7 | 0 | -0.147 | -0.118 | 2820 |
| 4484 | 683 | chlorcyclizine | 12 µM | PC3 | 0 | -0.148 | -0.075 | 3810 |
| 4485 | 657 | 10-methoxyharmalan | 19 µM | MCF7 | 0 | -0.149 | -0.106 | 2893 |
| 4486 | 1066 | thapsigargin | 100 nM | MCF7 | 0 | -0.151 | -0.062 | 7053 |
| 4487 | 1023 | F0447-0125 | 10 µM | PC3 | 0 | -0.152 | -0.122 | 6401 |
| 4488 | 658 | nadolol | 13 µM | HL60 | 0 | -0.155 | -0.089 | 3020 |
| 4489 | 31 | novobiocin | 100 µM | MCF7 | 0 | -0.155 | -0.086 | 342 |
| 4490 | 660 | megestrol | 10 µM | HL60 | 0 | -0.156 | -0.123 | 3091 |
| 4491 | 641 | (+)-chelidonine | 11 µM | HL60 | 0 | -0.157 | -0.148 | 1786 |
| 4492 | 87 | butein | 10 µM | PC3 | 0 | -0.173 | -0.037 | 582 |
| 4493 | 1078 | thapsigargin | 100 nM | MCF7 | 0 | -0.18 | -0.106 | 7100 |
| 4494 | 1062 | H-89 | 500 nM | PC3 | -0.245 | -0.03 | 0.047 | 6921 |
| 4495 | 714 | cisapride | 9 µM | PC3 | -0.246 | -0.035 | 0.042 | 6706 |
| 4496 | 726 | pirenperone | 10 µM | MCF7 | -0.258 | -0.042 | 0.039 | 5274 |
| 4497 | 715 | rolitetracycline | 8 µM | PC3 | -0.271 | -0.033 | 0.052 | 6731 |
| 4498 | 1094 | BCB000040 | 10 µM | MCF7 | -0.272 | -0.044 | 0.042 | 7554 |
| 4499 | 715 | caffeic acid | 22 µM | PC3 | -0.273 | -0.036 | 0.05 | 6753 |
| 4500 | 715 | buspirone | 9 µM | PC3 | -0.273 | -0.042 | 0.044 | 6743 |
| 4501 | 682 | helveticoside | 7 µM | PC3 | -0.283 | -0.045 | 0.044 | 3770 |
| 4502 | 1095 | vinblastine | 100 nM | PC3 | -0.286 | -0.039 | 0.051 | 7556 |
| 4503 | 764 | sulfapyridine | 16 µM | PC3 | -0.291 | -0.049 | 0.042 | 7151 |
| 4504 | 710 | estrone | 15 µM | PC3 | -0.294 | -0.039 | 0.053 | 6647 |
| 4505 | 733 | ticarcillin | 9 µM | PC3 | -0.295 | -0.041 | 0.051 | 5829 |
| 4506 | 1016 | PF-01378883-00 | 10 µM | MCF7 | -0.296 | -0.036 | 0.057 | 6363 |
| 4507 | 677 | bethanechol | 20 µM | MCF7 | -0.298 | -0.034 | 0.059 | 3537 |
| 4508 | 37 | rofecoxib | 10 µM | HL60 | -0.298 | -0.039 | 0.055 | 371 |
| 4509 | 712 | procainamide | 15 µM | PC3 | -0.299 | -0.047 | 0.047 | 4602 |
| 4510 | 1017 | PF-01378883-00 | 10 µM | PC3 | -0.304 | -0.038 | 0.058 | 6368 |
| 4511 | 616 | gentamicin | 3 µM | PC3 | -0.305 | -0.046 | 0.05 | 2082 |
| 4512 | 1025 | PF-01378883-00 | 10 µM | PC3 | -0.307 | -0.044 | 0.052 | 6410 |
| 4513 | 1073 | AH-6809 | 1 µM | PC3 | -0.308 | -0.047 | 0.05 | 7075 |
| 4514 | 693 | procyclidine | 12 µM | PC3 | -0.309 | -0.046 | 0.051 | 4233 |
| 4515 | 756 | enilconazole | 13 µM | MCF7 | -0.312 | -0.057 | 0.041 | 6518 |
| 4516 | 680 | sulfamethoxazole | 16 µM | PC3 | -0.312 | -0.041 | 0.057 | 3667 |
| 4517 | 707 | benzbromarone | 9 µM | MCF7 | -0.313 | -0.048 | 0.05 | 5015 |
| 4518 | 616 | atropine oxide | 12 µM | PC3 | -0.313 | -0.053 | 0.045 | 2054 |
| 4519 | 726 | phthalylsulfathiazole | 10 µM | MCF7 | -0.316 | -0.042 | 0.057 | 5249 |
| 4520 | 718 | niflumic acid | 14 µM | PC3 | -0.317 | -0.039 | 0.06 | 5071 |
| 4521 | 731 | fipexide | 9 µM | PC3 | -0.317 | -0.051 | 0.048 | 5737 |
| 4522 | 741 | isotretinoin | 13 µM | MCF7 | -0.317 | -0.044 | 0.055 | 6017 |
| 4523 | 729 | paclitaxel | 5 µM | MCF7 | -0.32 | -0.05 | 0.05 | 5320 |
| 4524 | 710 | bezafibrate | 11 µM | PC3 | -0.32 | -0.056 | 0.044 | 6653 |
| 4525 | 616 | astemizole | 9 µM | PC3 | -0.32 | -0.057 | 0.043 | 2049 |
| 4526 | 506 | monorden | 100 nM | MCF7 | -0.321 | -0.041 | 0.06 | 999 |
| 4527 | 758 | alfuzosin | 9 µM | MCF7 | -0.321 | -0.043 | 0.058 | 5605 |
| 4528 | 1094 | CP-863187 | 10 µM | MCF7 | -0.322 | -0.059 | 0.042 | 7553 |
| 4529 | 96 | nifedipine | 10 µM | MCF7 | -0.324 | -0.033 | 0.069 | 603 |
| 4530 | 693 | carcinine | 22 µM | PC3 | -0.324 | -0.053 | 0.048 | 4225 |
| 4531 | 686 | PHA-00851261E | 1 µM | MCF7 | -0.325 | -0.053 | 0.048 | 3857 |
| 4532 | 718 | dapsone | 16 µM | PC3 | -0.325 | -0.048 | 0.054 | 5078 |
| 4533 | 754 | sulfasalazine | 10 µM | PC3 | -0.325 | -0.043 | 0.059 | 6346 |
| 4534 | 603 | monorden | 100 nM | PC3 | -0.326 | -0.046 | 0.056 | 1219 |
| 4535 | 731 | amodiaquine | 9 µM | PC3 | -0.327 | -0.037 | 0.066 | 5747 |
| 4536 | 687 | parbendazole | 16 µM | MCF7 | -0.328 | -0.04 | 0.063 | 3881 |
| 4537 | 715 | cefotiam | 7 µM | PC3 | -0.328 | -0.038 | 0.065 | 6762 |
| 4538 | 18 | celecoxib | 10 µM | MCF7 | -0.328 | -0.039 | 0.064 | 252 |
| 4539 | 715 | (-)-atenolol | 15 µM | PC3 | -0.329 | -0.058 | 0.045 | 6725 |
| 4540 | 746 | dexamethasone | 9 µM | MCF7 | -0.33 | -0.035 | 0.068 | 6271 |
| 4541 | 709 | liothyronine | 6 µM | PC3 | -0.33 | -0.065 | 0.039 | 6602 |
| 4542 | 732 | tremorine | 15 µM | PC3 | -0.331 | -0.048 | 0.055 | 5799 |
| 4543 | 677 | pivmecillinam | 8 µM | MCF7 | -0.331 | -0.049 | 0.054 | 3535 |
| 4544 | 677 | penbutolol | 6 µM | MCF7 | -0.332 | -0.05 | 0.054 | 3534 |
| 4545 | 706 | omeprazole | 12 µM | MCF7 | -0.333 | -0.048 | 0.057 | 4951 |
| 4546 | 1054 | pioglitazone | 10 µM | PC3 | -0.334 | -0.056 | 0.048 | 6893 |
| 4547 | 713 | probucol | 8 µM | PC3 | -0.334 | -0.049 | 0.056 | 4666 |
| 4548 | 709 | glimepiride | 8 µM | PC3 | -0.335 | -0.054 | 0.051 | 6628 |
| 4549 | 753 | baclofen | 19 µM | PC3 | -0.337 | -0.047 | 0.058 | 6313 |
| 4550 | 752 | fluorometholone | 11 µM | MCF7 | -0.338 | -0.046 | 0.06 | 6071 |
| 4551 | 734 | milrinone | 19 µM | PC3 | -0.339 | -0.048 | 0.058 | 5856 |
| 4552 | 45 | estradiol | 10 nM | ssMCF7 | -0.339 | -0.05 | 0.056 | 414 |
| 4553 | 614 | clindamycin | 9 µM | HL60 | -0.339 | -0.041 | 0.065 | 1373 |
| 4554 | 730 | propranolol | 14 µM | MCF7 | -0.341 | -0.045 | 0.062 | 5358 |
| 4555 | 1094 | meteneprost | 10 µM | MCF7 | -0.342 | -0.045 | 0.062 | 7552 |
| 4556 | 1080 | BCB000040 | 10 µM | MCF7 | -0.342 | -0.053 | 0.054 | 7488 |
| 4557 | 54 | iloprost | 1 µM | MCF7 | -0.343 | -0.042 | 0.065 | 427 |
| 4558 | 628 | diprophylline | 16 µM | PC3 | -0.343 | -0.057 | 0.051 | 1811 |
| 4559 | 693 | minaprine | 11 µM | PC3 | -0.346 | -0.034 | 0.075 | 4230 |
| 4560 | 694 | probenecid | 14 µM | MCF7 | -0.347 | -0.042 | 0.067 | 4771 |
| 4561 | 706 | pyrazinamide | 32 µM | MCF7 | -0.348 | -0.046 | 0.063 | 4962 |
| 4562 | 689 | decamethonium bromide | 10 µM | PC3 | -0.348 | -0.044 | 0.065 | 4094 |
| 4563 | 694 | benzocaine | 24 µM | MCF7 | -0.348 | -0.058 | 0.051 | 4808 |
| 4564 | 603 | fluphenazine | 10 µM | PC3 | -0.348 | -0.038 | 0.071 | 1237 |
| 4565 | 101 | geldanamycin | 1 µM | MCF7 | -0.348 | -0.067 | 0.042 | 611 |
| 4566 | 695 | zuclopenthixol | 9 µM | MCF7 | -0.349 | -0.053 | 0.057 | 4843 |
| 4567 | 37 | rosiglitazone | 10 µM | HL60 | -0.349 | -0.062 | 0.048 | 369 |
| 4568 | 734 | mepyramine | 10 µM | PC3 | -0.35 | -0.037 | 0.073 | 5869 |
| 4569 | 695 | carcinine | 22 µM | MCF7 | -0.351 | -0.064 | 0.046 | 4809 |
| 4570 | 648 | sulfaquinoxaline | 12 µM | HL60 | -0.351 | -0.06 | 0.05 | 2528 |
| 4571 | 37 | troglitazone | 10 µM | HL60 | -0.352 | -0.043 | 0.067 | 370 |
| 4572 | 685 | bromopride | 12 µM | MCF7 | -0.352 | -0.051 | 0.06 | 3617 |
| 4573 | 687 | CP-320650-01 | 10 µM | MCF7 | -0.352 | -0.053 | 0.057 | 3905 |
| 4574 | 751 | methylbenzethonium chloride | 9 µM | MCF7 | -0.352 | -0.058 | 0.053 | 6045 |
| 4575 | 754 | remoxipride | 10 µM | PC3 | -0.353 | -0.048 | 0.062 | 6342 |
| 4576 | 616 | pirenzepine | 9 µM | PC3 | -0.353 | -0.051 | 0.059 | 2071 |
| 4577 | 765 | fulvestrant | 10 nM | MCF7 | -0.353 | -0.054 | 0.056 | 6997 |
| 4578 | 678 | chloropyrazine | 35 µM | MCF7 | -0.353 | -0.054 | 0.057 | 3570 |
| 4579 | 703 | tolbutamide | 15 µM | PC3 | -0.354 | -0.047 | 0.063 | 4540 |
| 4580 | 29 | azathioprine | 100 µM | MCF7 | -0.355 | -0.061 | 0.05 | 338 |
| 4581 | 736 | nomegestrol | 11 µM | MCF7 | -0.355 | -0.06 | 0.051 | 5461 |
| 4582 | 617 | flufenamic acid | 14 µM | PC3 | -0.355 | -0.058 | 0.054 | 2104 |
| 4583 | 735 | pralidoxime | 23 µM | MCF7 | -0.355 | -0.047 | 0.064 | 5383 |
| 4584 | 637 | chlorphenesin | 16 µM | MCF7 | -0.356 | -0.056 | 0.055 | 2279 |
| 4585 | 741 | meticrane | 15 µM | MCF7 | -0.356 | -0.052 | 0.059 | 5984 |
| 4586 | 706 | nifurtimox | 14 µM | MCF7 | -0.356 | -0.046 | 0.065 | 4953 |
| 4587 | 747 | streptozocin | 15 µM | MCF7 | -0.356 | -0.053 | 0.059 | 7193 |
| 4588 | 714 | biotin | 16 µM | PC3 | -0.357 | -0.056 | 0.056 | 6689 |
| 4589 | 762 | imipenem | 13 µM | PC3 | -0.357 | -0.054 | 0.058 | 7294 |
| 4590 | 755 | N-acetyl-L-leucine | 23 µM | MCF7 | -0.357 | -0.052 | 0.06 | 6462 |
| 4591 | 698 | clemizole | 11 µM | PC3 | -0.359 | -0.058 | 0.055 | 7371 |
| 4592 | 698 | sulfadimethoxine | 13 µM | PC3 | -0.359 | -0.045 | 0.067 | 7400 |
| 4593 | 663 | boldine | 12 µM | MCF7 | -0.359 | -0.048 | 0.064 | 2804 |
| 4594 | 752 | leflunomide | 15 µM | MCF7 | -0.36 | -0.065 | 0.048 | 6102 |
| 4595 | 656 | salsolidin | 16 µM | MCF7 | -0.36 | -0.052 | 0.06 | 2824 |
| 4596 | 699 | pentolonium | 7 µM | MCF7 | -0.36 | -0.042 | 0.071 | 4699 |
| 4597 | 710 | etynodiol | 10 µM | PC3 | -0.361 | -0.068 | 0.045 | 6678 |
| 4598 | 766 | etamivan | 18 µM | MCF7 | -0.361 | -0.064 | 0.049 | 7021 |
| 4599 | 712 | hymecromone | 23 µM | PC3 | -0.361 | -0.053 | 0.06 | 4623 |
| 4600 | 734 | bergenin | 12 µM | PC3 | -0.361 | -0.045 | 0.069 | 5870 |
| 4601 | 699 | tiapride | 11 µM | MCF7 | -0.362 | -0.064 | 0.049 | 4686 |
| 4602 | 647 | ascorbic acid | 22 µM | MCF7 | -0.362 | -0.068 | 0.046 | 3225 |
| 4603 | 709 | apramycin | 7 µM | PC3 | -0.363 | -0.052 | 0.062 | 6614 |
| 4604 | 755 | nimodipine | 10 µM | MCF7 | -0.363 | -0.05 | 0.064 | 6480 |
| 4605 | 1067 | SB-202190 | 1 µM | PC3 | -0.363 | -0.061 | 0.053 | 7058 |
| 4606 | 710 | hydroxyzine | 9 µM | PC3 | -0.364 | -0.063 | 0.051 | 6660 |
| 4607 | 116 | mercaptopurine | 10 µM | PC3 | -0.364 | -0.064 | 0.05 | 667 |
| 4608 | 714 | mimosine | 20 µM | PC3 | -0.365 | -0.049 | 0.066 | 6703 |
| 4609 | 710 | edrophonium chloride | 20 µM | PC3 | -0.365 | -0.056 | 0.058 | 6655 |
| 4610 | 766 | pargyline | 20 µM | MCF7 | -0.365 | -0.055 | 0.06 | 7016 |
| 4611 | 513 | troglitazone | 10 µM | MCF7 | -0.366 | -0.063 | 0.052 | 1070 |
| 4612 | 657 | mometasone | 8 µM | MCF7 | -0.367 | -0.05 | 0.065 | 2896 |
| 4613 | 683 | Prestwick-1080 | 15 µM | PC3 | -0.367 | -0.062 | 0.053 | 3796 |
| 4614 | 633 | loxapine | 9 µM | MCF7 | -0.367 | -0.048 | 0.067 | 1516 |
| 4615 | 678 | thiethylperazine | 6 µM | MCF7 | -0.367 | -0.052 | 0.063 | 3576 |
| 4616 | 1036 | 6-bromoindirubin-3'-oxime | 500 nM | PC3 | -0.367 | -0.04 | 0.075 | 6559 |
| 4617 | 56 | novobiocin | 100 µM | PC3 | -0.367 | -0.064 | 0.051 | 435 |
| 4618 | 514 | doxycycline | 14 µM | MCF7 | -0.368 | -0.057 | 0.059 | 1113 |
| 4619 | 756 | Prestwick-920 | 14 µM | MCF7 | -0.368 | -0.051 | 0.064 | 6497 |
| 4620 | 726 | loperamide | 8 µM | MCF7 | -0.369 | -0.033 | 0.083 | 5267 |
| 4621 | 730 | betamethasone | 10 µM | MCF7 | -0.37 | -0.03 | 0.086 | 5328 |
| 4622 | 719 | todralazine | 15 µM | PC3 | -0.37 | -0.058 | 0.058 | 5087 |
| 4623 | 757 | tanespimycin | 1 µM | MCF7 | -0.372 | -0.063 | 0.054 | 5578 |
| 4624 | 733 | meropenem | 10 µM | PC3 | -0.372 | -0.055 | 0.061 | 5824 |
| 4625 | 745 | pheneticillin | 10 µM | MCF7 | -0.372 | -0.064 | 0.052 | 6239 |
| 4626 | 728 | zomepirac | 13 µM | PC3 | -0.372 | -0.05 | 0.067 | 4479 |
| 4627 | 712 | naringin | 7 µM | PC3 | -0.372 | -0.049 | 0.067 | 4605 |
| 4628 | 617 | levamisole | 17 µM | PC3 | -0.373 | -0.065 | 0.052 | 2094 |
| 4629 | 646 | clomipramine | 11 µM | MCF7 | -0.373 | -0.066 | 0.051 | 3182 |
| 4630 | 688 | cefotetan | 7 µM | PC3 | -0.373 | -0.045 | 0.072 | 3997 |
| 4631 | 692 | norcyclobenzaprine | 15 µM | PC3 | -0.373 | -0.047 | 0.07 | 4190 |
| 4632 | 637 | pyrantel | 11 µM | MCF7 | -0.373 | -0.057 | 0.06 | 2260 |
| 4633 | 711 | benfotiamine | 9 µM | MCF7 | -0.374 | -0.052 | 0.065 | 3931 |
| 4634 | 686 | piperacillin | 7 µM | MCF7 | -0.374 | -0.062 | 0.055 | 3845 |
| 4635 | 735 | metitepine | 8 µM | MCF7 | -0.375 | -0.049 | 0.069 | 5413 |
| 4636 | 647 | colchicine | 10 µM | MCF7 | -0.375 | -0.051 | 0.067 | 3213 |
| 4637 | 693 | cyclobenzaprine | 13 µM | PC3 | -0.376 | -0.039 | 0.078 | 4252 |
| 4638 | 730 | carbachol | 22 µM | MCF7 | -0.376 | -0.053 | 0.065 | 5342 |
| 4639 | 706 | theobromine | 22 µM | MCF7 | -0.376 | -0.049 | 0.069 | 4958 |
| 4640 | 767 | LY-294002 | 10 µM | MCF7 | -0.376 | -0.052 | 0.066 | 6945 |
| 4641 | 513 | rosiglitazone | 10 µM | MCF7 | -0.377 | -0.065 | 0.053 | 1071 |
| 4642 | 655 | cisapride | 9 µM | MCF7 | -0.377 | -0.05 | 0.068 | 3305 |
| 4643 | 1065 | AH-6809 | 1 µM | PC3 | -0.377 | -0.04 | 0.078 | 7049 |
| 4644 | 752 | naproxen | 16 µM | MCF7 | -0.378 | -0.058 | 0.061 | 6096 |
| 4645 | 710 | progesterone | 13 µM | PC3 | -0.379 | -0.053 | 0.066 | 6646 |
| 4646 | 728 | dihydroergotamine | 3 µM | PC3 | -0.379 | -0.064 | 0.055 | 4502 |
| 4647 | 703 | beta-escin | 3 µM | PC3 | -0.379 | -0.059 | 0.06 | 4544 |
| 4648 | 741 | fenspiride | 13 µM | MCF7 | -0.379 | -0.055 | 0.064 | 6001 |
| 4649 | 700 | flunixin | 8 µM | MCF7 | -0.38 | -0.057 | 0.062 | 4735 |
| 4650 | 762 | isotretinoin | 13 µM | PC3 | -0.38 | -0.057 | 0.062 | 7315 |
| 4651 | 59 | arachidonic acid | 10 µM | MCF7 | -0.38 | -0.054 | 0.065 | 443 |
| 4652 | 706 | SR-95639A | 10 µM | MCF7 | -0.381 | -0.055 | 0.064 | 4977 |
| 4653 | 693 | corynanthine | 10 µM | PC3 | -0.381 | -0.042 | 0.077 | 4227 |
| 4654 | 764 | antazoline | 13 µM | PC3 | -0.381 | -0.062 | 0.058 | 7128 |
| 4655 | 514 | minocycline | 11 µM | MCF7 | -0.381 | -0.062 | 0.057 | 1135 |
| 4656 | 713 | enoxacin | 12 µM | PC3 | -0.381 | -0.052 | 0.068 | 4655 |
| 4657 | 615 | nimesulide | 13 µM | HL60 | -0.382 | -0.049 | 0.071 | 1428 |
| 4658 | 762 | epirizole | 17 µM | PC3 | -0.382 | -0.046 | 0.074 | 7292 |
| 4659 | 680 | 0175029-0000 | 1 µM | PC3 | -0.383 | -0.064 | 0.056 | 3694 |
| 4660 | 107 | nocodazole | 1 µM | MCF7 | -0.384 | -0.049 | 0.071 | 621 |
| 4661 | 60 | diclofenac | 10 µM | PC3 | -0.384 | -0.085 | 0.035 | 445 |
| 4662 | 743 | methylprednisolone | 11 µM | MCF7 | -0.385 | -0.06 | 0.061 | 6785 |
| 4663 | 683 | CP-690334-01 | 1 µM | PC3 | -0.385 | -0.05 | 0.07 | 3826 |
| 4664 | 705 | novobiocin | 6 µM | MCF7 | -0.385 | -0.051 | 0.07 | 4392 |
| 4665 | 646 | oxybutynin | 10 µM | MCF7 | -0.386 | -0.055 | 0.066 | 3168 |
| 4666 | 1015 | PF-00539745-00 | 10 µM | PC3 | -0.386 | -0.063 | 0.058 | 5979 |
| 4667 | 678 | ramipril | 10 µM | MCF7 | -0.386 | -0.072 | 0.049 | 3572 |
| 4668 | 698 | trimethoprim | 14 µM | PC3 | -0.386 | -0.07 | 0.051 | 7377 |
| 4669 | 43 | sodium phenylbutyrate | 1 mM | MCF7 | -0.386 | -0.046 | 0.076 | 408 |
| 4670 | 751 | chlorcyclizine | 12 µM | MCF7 | -0.387 | -0.069 | 0.053 | 6053 |
| 4671 | 693 | doxylamine | 10 µM | PC3 | -0.387 | -0.059 | 0.062 | 4235 |
| 4672 | 732 | bupropion | 14 µM | PC3 | -0.387 | -0.043 | 0.078 | 5782 |
| 4673 | 720 | beta-escin | 3 µM | MCF7 | -0.387 | -0.04 | 0.081 | 4364 |
| 4674 | 753 | ampicillin | 10 µM | PC3 | -0.387 | -0.057 | 0.065 | 6307 |
| 4675 | 752 | sulfaquinoxaline | 12 µM | MCF7 | -0.388 | -0.055 | 0.066 | 6090 |
| 4676 | 758 | serotonin | 19 µM | MCF7 | -0.389 | -0.058 | 0.064 | 5633 |
| 4677 | 617 | oxolinic acid | 15 µM | PC3 | -0.389 | -0.048 | 0.074 | 2103 |
| 4678 | 686 | sulfadimidine | 13 µM | MCF7 | -0.389 | -0.059 | 0.063 | 3847 |
| 4679 | 695 | Prestwick-967 | 26 µM | MCF7 | -0.389 | -0.056 | 0.066 | 4833 |
| 4680 | 68 | haloperidol | 10 µM | MCF7 | -0.389 | -0.032 | 0.09 | 492 |
| 4681 | 649 | eucatropine | 12 µM | HL60 | -0.389 | -0.058 | 0.064 | 2556 |
| 4682 | 758 | methyldopa | 19 µM | MCF7 | -0.389 | -0.074 | 0.048 | 5637 |
| 4683 | 718 | indometacin | 11 µM | PC3 | -0.39 | -0.073 | 0.049 | 5049 |
| 4684 | 616 | ursolic acid | 9 µM | PC3 | -0.39 | -0.057 | 0.065 | 2067 |
| 4685 | 752 | altizide | 10 µM | MCF7 | -0.391 | -0.055 | 0.067 | 6089 |
| 4686 | 680 | pentolonium | 7 µM | PC3 | -0.391 | -0.046 | 0.077 | 3676 |
| 4687 | 734 | repaglinide | 9 µM | PC3 | -0.391 | -0.066 | 0.057 | 5862 |
| 4688 | 506 | sirolimus | 100 nM | MCF7 | -0.391 | -0.05 | 0.072 | 987 |
| 4689 | 725 | monorden | 100 nM | MCF7 | -0.391 | -0.065 | 0.057 | 5216 |
| 4690 | 17 | tanespimycin | 100 nM | MCF7 | -0.392 | -0.082 | 0.041 | 221 |
| 4691 | 744 | dihydroergotamine | 3 µM | MCF7 | -0.392 | -0.056 | 0.067 | 6840 |
| 4692 | 703 | PHA-00745360 | 10 µM | PC3 | -0.392 | -0.061 | 0.062 | 4559 |
| 4693 | 36 | estradiol | 100 nM | MCF7 | -0.392 | -0.069 | 0.054 | 365 |
| 4694 | 1044 | dinoprostone | 10 µM | PC3 | -0.392 | -0.066 | 0.057 | 6586 |
| 4695 | 710 | methotrexate | 9 µM | PC3 | -0.392 | -0.052 | 0.071 | 6654 |
| 4696 | 771 | bupivacaine | 12 µM | MCF7 | -0.393 | -0.062 | 0.061 | 7435 |
| 4697 | 676 | meptazinol | 15 µM | MCF7 | -0.393 | -0.075 | 0.048 | 7326 |
| 4698 | 735 | ganciclovir | 16 µM | MCF7 | -0.394 | -0.063 | 0.06 | 5389 |
| 4699 | 628 | naproxen | 17 µM | PC3 | -0.394 | -0.068 | 0.056 | 1828 |
| 4700 | 771 | indometacin | 11 µM | MCF7 | -0.394 | -0.056 | 0.067 | 7409 |
| 4701 | 695 | picrotoxinin | 14 µM | MCF7 | -0.394 | -0.055 | 0.068 | 4842 |
| 4702 | 628 | chloramphenicol | 12 µM | PC3 | -0.394 | -0.074 | 0.049 | 1795 |
| 4703 | 709 | lysergol | 16 µM | PC3 | -0.394 | -0.035 | 0.089 | 6621 |
| 4704 | 689 | carbenoxolone | 7 µM | PC3 | -0.394 | -0.062 | 0.061 | 4093 |
| 4705 | 701 | PNU-0230031 | 10 µM | PC3 | -0.395 | -0.058 | 0.065 | 4288 |
| 4706 | 514 | yohimbine | 23 µM | MCF7 | -0.395 | -0.066 | 0.058 | 1119 |
| 4707 | 670 | isocarboxazid | 17 µM | MCF7 | -0.395 | -0.05 | 0.074 | 3424 |
| 4708 | 752 | abamectin | 5 µM | MCF7 | -0.396 | -0.063 | 0.061 | 6081 |
| 4709 | 646 | sulfinpyrazone | 10 µM | MCF7 | -0.396 | -0.06 | 0.065 | 3192 |
| 4710 | 692 | mebhydrolin | 5 µM | PC3 | -0.396 | -0.063 | 0.061 | 4211 |
| 4711 | 766 | flunarizine | 8 µM | MCF7 | -0.397 | -0.062 | 0.062 | 7013 |
| 4712 | 765 | monorden | 100 nM | MCF7 | -0.397 | -0.065 | 0.06 | 6979 |
| 4713 | 693 | picrotoxinin | 14 µM | PC3 | -0.398 | -0.061 | 0.064 | 4260 |
| 4714 | 755 | bephenium hydroxynaphthoate | 9 µM | MCF7 | -0.398 | -0.062 | 0.062 | 6466 |
| 4715 | 506 | haloperidol | 10 µM | MCF7 | -0.398 | -0.065 | 0.06 | 1024 |
| 4716 | 707 | hydroxyzine | 9 µM | MCF7 | -0.398 | -0.065 | 0.06 | 5006 |
| 4717 | 610 | ticlopidine | 13 µM | PC3 | -0.399 | -0.089 | 0.036 | 1895 |
| 4718 | 767 | LY-294002 | 10 µM | MCF7 | -0.399 | -0.058 | 0.067 | 6956 |
| 4719 | 689 | betulinic acid | 9 µM | PC3 | -0.399 | -0.06 | 0.065 | 4101 |
| 4720 | 655 | amiodarone | 6 µM | MCF7 | -0.399 | -0.074 | 0.051 | 3296 |
| 4721 | 683 | orphenadrine | 13 µM | PC3 | -0.4 | -0.058 | 0.068 | 3801 |
| 4722 | 744 | hydrocotarnine | 13 µM | MCF7 | -0.4 | -0.048 | 0.078 | 6827 |
| 4723 | 56 | fasudil | 10 µM | PC3 | -0.4 | -0.067 | 0.059 | 436 |
| 4724 | 654 | amantadine | 10 µM | MCF7 | -0.4 | -0.054 | 0.072 | 3280 |
| 4725 | 765 | clozapine | 10 µM | MCF7 | -0.4 | -0.06 | 0.066 | 6988 |
| 4726 | 762 | proxyphylline | 17 µM | PC3 | -0.4 | -0.074 | 0.051 | 7290 |
| 4727 | 506 | tanespimycin | 1 µM | MCF7 | -0.4 | -0.081 | 0.045 | 998 |
| 4728 | 610 | sulfaguanidine | 19 µM | PC3 | -0.4 | -0.062 | 0.064 | 1913 |
| 4729 | 710 | amoxapine | 13 µM | PC3 | -0.4 | -0.059 | 0.066 | 6650 |
| 4730 | 678 | cyclopentolate | 12 µM | MCF7 | -0.4 | -0.073 | 0.053 | 3555 |
| 4731 | 758 | loperamide | 8 µM | MCF7 | -0.4 | -0.079 | 0.046 | 5632 |
| 4732 | 655 | pirenperone | 10 µM | MCF7 | -0.4 | -0.065 | 0.061 | 3316 |
| 4733 | 732 | papaverine | 11 µM | PC3 | -0.4 | -0.048 | 0.077 | 5769 |
| 4734 | 20 | indometacin | 20 µM | MCF7 | -0.401 | -0.039 | 0.087 | 262 |
| 4735 | 693 | (+/-)-catechin | 14 µM | PC3 | -0.401 | -0.074 | 0.052 | 4255 |
| 4736 | 633 | naloxone | 11 µM | MCF7 | -0.401 | -0.062 | 0.064 | 1506 |
| 4737 | 682 | furaltadone | 11 µM | PC3 | -0.402 | -0.06 | 0.066 | 3756 |
| 4738 | 688 | ginkgolide A | 10 µM | PC3 | -0.402 | -0.043 | 0.083 | 4002 |
| 4739 | 752 | streptozocin | 15 µM | MCF7 | -0.402 | -0.071 | 0.055 | 6098 |
| 4740 | 709 | SR-95639A | 10 µM | PC3 | -0.403 | -0.048 | 0.079 | 6632 |
| 4741 | 702 | phentolamine | 13 µM | PC3 | -0.403 | -0.038 | 0.088 | 4336 |
| 4742 | 656 | quercetin | 12 µM | MCF7 | -0.403 | -0.044 | 0.082 | 2859 |
| 4743 | 637 | chlorzoxazone | 24 µM | MCF7 | -0.403 | -0.077 | 0.049 | 2263 |
| 4744 | 730 | fluoxetine | 12 µM | MCF7 | -0.403 | -0.063 | 0.064 | 5356 |
| 4745 | 731 | mefloquine | 10 µM | PC3 | -0.403 | -0.065 | 0.062 | 5724 |
| 4746 | 726 | cefalexin | 11 µM | MCF7 | -0.404 | -0.048 | 0.079 | 5250 |
| 4747 | 616 | nocodazole | 13 µM | PC3 | -0.404 | -0.064 | 0.063 | 2076 |
| 4748 | 1084 | 16-phenyltetranorprostaglandin E2 | 10 µM | MCF7 | -0.404 | -0.043 | 0.084 | 7505 |
| 4749 | 748 | mepyramine | 10 µM | MCF7 | -0.404 | -0.064 | 0.063 | 7223 |
| 4750 | 738 | enilconazole | 13 µM | MCF7 | -0.404 | -0.071 | 0.055 | 5538 |
| 4751 | 743 | naproxen | 16 µM | MCF7 | -0.405 | -0.079 | 0.048 | 6794 |
| 4752 | 701 | myosmine | 27 µM | PC3 | -0.405 | -0.061 | 0.066 | 4293 |
| 4753 | 71 | sodium phenylbutyrate | 200 µM | SKMEL5 | -0.405 | -0.091 | 0.036 | 502 |
| 4754 | 727 | prochlorperazine | 10 µM | PC3 | -0.405 | -0.036 | 0.091 | 4439 |
| 4755 | 654 | cefadroxil | 11 µM | MCF7 | -0.406 | -0.077 | 0.05 | 3259 |
| 4756 | 756 | nomegestrol | 11 µM | MCF7 | -0.406 | -0.064 | 0.064 | 6525 |
| 4757 | 757 | nordihydroguaiaretic acid | 1 µM | MCF7 | -0.406 | -0.065 | 0.062 | 5583 |
| 4758 | 751 | gossypol | 8 µM | MCF7 | -0.406 | -0.048 | 0.079 | 6058 |
| 4759 | 756 | bacitracin | 3 µM | MCF7 | -0.406 | -0.076 | 0.051 | 6488 |
| 4760 | 767 | estradiol | 100 nM | MCF7 | -0.406 | -0.062 | 0.066 | 6928 |
| 4761 | 636 | spironolactone | 10 µM | MCF7 | -0.406 | -0.063 | 0.064 | 2226 |
| 4762 | 738 | gliclazide | 12 µM | MCF7 | -0.407 | -0.041 | 0.086 | 5514 |
| 4763 | 1095 | BCB000040 | 10 µM | PC3 | -0.408 | -0.063 | 0.065 | 7559 |
| 4764 | 74 | nordihydroguaiaretic acid | 1 µM | ssMCF7 | -0.408 | -0.045 | 0.083 | 524 |
| 4765 | 737 | norethisterone | 13 µM | MCF7 | -0.408 | -0.054 | 0.075 | 5474 |
| 4766 | 753 | dinoprost | 8 µM | PC3 | -0.409 | -0.083 | 0.045 | 6308 |
| 4767 | 659 | diclofenamide | 13 µM | HL60 | -0.409 | -0.056 | 0.072 | 3027 |
| 4768 | 715 | nilutamide | 13 µM | PC3 | -0.409 | -0.088 | 0.04 | 6763 |
| 4769 | 628 | sulfacetamide | 16 µM | PC3 | -0.409 | -0.062 | 0.066 | 1817 |
| 4770 | 655 | nitrofural | 20 µM | MCF7 | -0.409 | -0.064 | 0.064 | 3320 |
| 4771 | 705 | cyanocobalamin | 3 µM | MCF7 | -0.409 | -0.047 | 0.081 | 4395 |
| 4772 | 698 | riluzole | 15 µM | PC3 | -0.41 | -0.05 | 0.079 | 7365 |
| 4773 | 626 | troglitazone | 10 µM | MCF7 | -0.41 | -0.069 | 0.059 | 1657 |
| 4774 | 699 | cefamandole | 8 µM | MCF7 | -0.411 | -0.078 | 0.051 | 4718 |
| 4775 | 702 | mexiletine | 19 µM | PC3 | -0.411 | -0.045 | 0.084 | 4338 |
| 4776 | 734 | estriol | 14 µM | PC3 | -0.411 | -0.05 | 0.079 | 5866 |
| 4777 | 502 | rottlerin | 10 µM | MCF7 | -0.411 | -0.08 | 0.048 | 941 |
| 4778 | 1041 | PNU-0293363 | 10 µM | MCF7 | -0.411 | -0.063 | 0.066 | 6573 |
| 4779 | 632 | pyrimethamine | 16 µM | MCF7 | -0.411 | -0.078 | 0.051 | 1474 |
| 4780 | 632 | lidocaine | 15 µM | MCF7 | -0.412 | -0.074 | 0.055 | 1499 |
| 4781 | 1050 | BAS-012416453 | 38 µM | PC3 | -0.412 | -0.049 | 0.08 | 6876 |
| 4782 | 683 | diethylstilbestrol | 15 µM | PC3 | -0.412 | -0.044 | 0.085 | 3812 |
| 4783 | 761 | mecamylamine | 20 µM | PC3 | -0.412 | -0.044 | 0.085 | 7263 |
| 4784 | 712 | chlormezanone | 15 µM | PC3 | -0.412 | -0.063 | 0.066 | 4636 |
| 4785 | 645 | finasteride | 11 µM | HL60 | -0.412 | -0.046 | 0.083 | 2206 |
| 4786 | 656 | propylthiouracil | 23 µM | MCF7 | -0.413 | -0.048 | 0.081 | 2837 |
| 4787 | 753 | dequalinium chloride | 8 µM | PC3 | -0.413 | -0.043 | 0.086 | 6296 |
| 4788 | 681 | fenoprofen | 7 µM | PC3 | -0.413 | -0.053 | 0.077 | 3714 |
| 4789 | 731 | alclometasone | 8 µM | PC3 | -0.413 | -0.069 | 0.06 | 5752 |
| 4790 | 726 | serotonin | 19 µM | MCF7 | -0.413 | -0.052 | 0.077 | 5268 |
| 4791 | 695 | sulfaguanidine | 19 µM | MCF7 | -0.414 | -0.064 | 0.065 | 4839 |
| 4792 | 18 | rofecoxib | 10 µM | MCF7 | -0.414 | -0.067 | 0.063 | 251 |
| 4793 | 71 | indometacin | 100 µM | SKMEL5 | -0.414 | -0.061 | 0.069 | 503 |
| 4794 | 681 | levonorgestrel | 13 µM | PC3 | -0.414 | -0.052 | 0.078 | 3708 |
| 4795 | 671 | cicloheximide | 14 µM | MCF7 | -0.415 | -0.069 | 0.061 | 3464 |
| 4796 | 670 | lithocholic acid | 11 µM | MCF7 | -0.415 | -0.036 | 0.094 | 3433 |
| 4797 | 671 | hexylcaine | 13 µM | MCF7 | -0.415 | -0.063 | 0.068 | 3447 |
| 4798 | 673 | nizatidine | 12 µM | MCF7 | -0.415 | -0.063 | 0.067 | 3385 |
| 4799 | 683 | Prestwick-1100 | 9 µM | PC3 | -0.415 | -0.052 | 0.079 | 3798 |
| 4800 | 709 | Prestwick-857 | 12 µM | PC3 | -0.416 | -0.073 | 0.057 | 6635 |
| 4801 | 25 | N-phenylanthranilic acid | 10 µM | MCF7 | -0.416 | -0.054 | 0.076 | 317 |
| 4802 | 689 | tenoxicam | 12 µM | PC3 | -0.416 | -0.053 | 0.078 | 4102 |
| 4803 | 706 | khellin | 15 µM | MCF7 | -0.416 | -0.065 | 0.066 | 4987 |
| 4804 | 761 | chloroquine | 8 µM | PC3 | -0.416 | -0.039 | 0.092 | 7251 |
| 4805 | 754 | proxymetacaine | 12 µM | PC3 | -0.416 | -0.079 | 0.052 | 6332 |
| 4806 | 662 | atropine | 6 µM | MCF7 | -0.416 | -0.065 | 0.065 | 2761 |
| 4807 | 693 | piperine | 14 µM | PC3 | -0.416 | -0.081 | 0.05 | 4247 |
| 4808 | 687 | alpha-ergocryptine | 7 µM | MCF7 | -0.416 | -0.073 | 0.057 | 3900 |
| 4809 | 686 | methylbenzethonium chloride | 9 µM | MCF7 | -0.416 | -0.061 | 0.07 | 3850 |
| 4810 | 688 | oxybuprocaine | 12 µM | PC3 | -0.417 | -0.073 | 0.058 | 3996 |
| 4811 | 756 | chrysin | 16 µM | MCF7 | -0.417 | -0.042 | 0.089 | 6485 |
| 4812 | 761 | dihydroergocristine | 6 µM | PC3 | -0.417 | -0.058 | 0.073 | 7275 |
| 4813 | 685 | nefopam | 14 µM | MCF7 | -0.417 | -0.061 | 0.07 | 3627 |
| 4814 | 702 | methylbenzethonium chloride | 9 µM | PC3 | -0.418 | -0.07 | 0.061 | 4325 |
| 4815 | 728 | sertaconazole | 8 µM | PC3 | -0.418 | -0.039 | 0.092 | 4475 |
| 4816 | 633 | bromocriptine | 5 µM | MCF7 | -0.418 | -0.068 | 0.063 | 1507 |
| 4817 | 656 | selegiline | 18 µM | MCF7 | -0.419 | -0.059 | 0.072 | 2826 |
| 4818 | 702 | 0317956-0000 | 10 µM | PC3 | -0.419 | -0.064 | 0.067 | 4331 |
| 4819 | 714 | naftidrofuryl | 8 µM | PC3 | -0.42 | -0.051 | 0.081 | 6687 |
| 4820 | 1083 | CP-944629 | 10 µM | PC3 | -0.42 | -0.081 | 0.05 | 7502 |
| 4821 | 622 | albendazole | 15 µM | HL60 | -0.42 | -0.045 | 0.086 | 1547 |
| 4822 | 738 | thiocolchicoside | 7 µM | MCF7 | -0.42 | -0.046 | 0.086 | 5520 |
| 4823 | 1009 | AG-013608 | 10 µM | PC3 | -0.42 | -0.076 | 0.056 | 5949 |
| 4824 | 650 | tanespimycin | 1 µM | HL60 | -0.42 | -0.054 | 0.078 | 2686 |
| 4825 | 647 | dobutamine | 12 µM | MCF7 | -0.42 | -0.052 | 0.08 | 3206 |
| 4826 | 754 | ranitidine | 11 µM | PC3 | -0.42 | -0.079 | 0.053 | 6324 |
| 4827 | 1016 | erastin | 20 µM | MCF7 | -0.421 | -0.068 | 0.064 | 6364 |
| 4828 | 757 | LY-294002 | 10 µM | MCF7 | -0.421 | -0.065 | 0.067 | 5587 |
| 4829 | 699 | metaraminol | 9 µM | MCF7 | -0.421 | -0.05 | 0.081 | 4692 |
| 4830 | 707 | estrone | 15 µM | MCF7 | -0.421 | -0.069 | 0.063 | 4993 |
| 4831 | 687 | guaifenesin | 20 µM | MCF7 | -0.421 | -0.065 | 0.067 | 3897 |
| 4832 | 762 | ethosuximide | 28 µM | PC3 | -0.421 | -0.046 | 0.086 | 7308 |
| 4833 | 646 | praziquantel | 13 µM | MCF7 | -0.421 | -0.057 | 0.075 | 3189 |
| 4834 | 655 | neostigmine bromide | 13 µM | MCF7 | -0.421 | -0.059 | 0.073 | 3294 |
| 4835 | 737 | torasemide | 11 µM | MCF7 | -0.421 | -0.047 | 0.085 | 5476 |
| 4836 | 1041 | alprostadil | 10 µM | MCF7 | -0.422 | -0.06 | 0.072 | 6576 |
| 4837 | 733 | isoniazid | 29 µM | PC3 | -0.422 | -0.079 | 0.053 | 5840 |
| 4838 | 685 | hydralazine | 20 µM | MCF7 | -0.422 | -0.047 | 0.085 | 3621 |
| 4839 | 613 | loperamide | 8 µM | HL60 | -0.422 | -0.055 | 0.078 | 2033 |
| 4840 | 707 | amoxapine | 13 µM | MCF7 | -0.423 | -0.059 | 0.074 | 4996 |
| 4841 | 612 | cinchocaine | 12 µM | HL60 | -0.423 | -0.043 | 0.089 | 1969 |
| 4842 | 686 | fludrocortisone | 9 µM | MCF7 | -0.423 | -0.062 | 0.071 | 3866 |
| 4843 | 718 | ifenprodil | 8 µM | PC3 | -0.423 | -0.057 | 0.076 | 5044 |
| 4844 | 611 | oleandomycin | 5 µM | PC3 | -0.423 | -0.083 | 0.05 | 1935 |
| 4845 | 731 | phenazopyridine | 16 µM | PC3 | -0.423 | -0.058 | 0.075 | 5758 |
| 4846 | 699 | riluzole | 15 µM | MCF7 | -0.423 | -0.058 | 0.075 | 4689 |
| 4847 | 658 | theobromine | 22 µM | HL60 | -0.423 | -0.066 | 0.067 | 2995 |
| 4848 | 752 | sulfapyridine | 16 µM | MCF7 | -0.423 | -0.053 | 0.08 | 6101 |
| 4849 | 617 | alverine | 8 µM | PC3 | -0.424 | -0.047 | 0.085 | 2110 |
| 4850 | 603 | tanespimycin | 1 µM | PC3 | -0.424 | -0.04 | 0.093 | 1206 |
| 4851 | 603 | alvespimycin | 100 nM | PC3 | -0.424 | -0.048 | 0.085 | 1213 |
| 4852 | 772 | ioxaglic acid | 3 µM | MCF7 | -0.424 | -0.061 | 0.072 | 7470 |
| 4853 | 689 | moxonidine | 17 µM | PC3 | -0.424 | -0.057 | 0.076 | 4084 |
| 4854 | 737 | roxarsone | 15 µM | MCF7 | -0.424 | -0.044 | 0.089 | 5470 |
| 4855 | 693 | 7-aminocephalosporanic acid | 15 µM | PC3 | -0.424 | -0.056 | 0.077 | 4242 |
| 4856 | 686 | fluocinonide | 8 µM | MCF7 | -0.424 | -0.065 | 0.068 | 3839 |
| 4857 | 633 | azathioprine | 14 µM | MCF7 | -0.424 | -0.051 | 0.082 | 1528 |
| 4858 | 617 | tiratricol | 6 µM | PC3 | -0.425 | -0.064 | 0.069 | 2096 |
| 4859 | 633 | metanephrine | 17 µM | MCF7 | -0.425 | -0.055 | 0.078 | 1515 |
| 4860 | 62 | indometacin | 100 µM | MCF7 | -0.425 | -0.039 | 0.094 | 453 |
| 4861 | 764 | cycloserine | 39 µM | PC3 | -0.425 | -0.057 | 0.076 | 7134 |
| 4862 | 617 | hyoscyamine | 14 µM | PC3 | -0.425 | -0.087 | 0.046 | 2108 |
| 4863 | 90 | docosahexaenoic acid ethyl ester | 100 µM | PC3 | -0.425 | -0.086 | 0.048 | 664 |
| 4864 | 746 | bupropion | 14 µM | MCF7 | -0.425 | -0.081 | 0.052 | 6256 |
| 4865 | 626 | sirolimus | 100 nM | MCF7 | -0.425 | -0.064 | 0.07 | 1667 |
| 4866 | 627 | epirizole | 17 µM | MCF7 | -0.426 | -0.07 | 0.064 | 1681 |
| 4867 | 734 | bucladesine | 8 µM | PC3 | -0.426 | -0.073 | 0.061 | 5886 |
| 4868 | 764 | carbinoxamine | 10 µM | PC3 | -0.426 | -0.044 | 0.09 | 7138 |
| 4869 | 633 | paclitaxel | 5 µM | MCF7 | -0.426 | -0.055 | 0.078 | 1542 |
| 4870 | 732 | oxytetracycline | 8 µM | PC3 | -0.427 | -0.037 | 0.097 | 5772 |
| 4871 | 710 | ouabain | 5 µM | PC3 | -0.427 | -0.078 | 0.056 | 6680 |
| 4872 | 737 | flufenamic acid | 14 µM | MCF7 | -0.427 | -0.045 | 0.089 | 5478 |
| 4873 | 764 | Prestwick-1084 | 16 µM | PC3 | -0.427 | -0.059 | 0.075 | 7125 |
| 4874 | 727 | alvespimycin | 100 nM | PC3 | -0.427 | -0.077 | 0.057 | 4437 |
| 4875 | 730 | nilutamide | 13 µM | MCF7 | -0.427 | -0.058 | 0.076 | 5362 |
| 4876 | 745 | thiethylperazine | 6 µM | MCF7 | -0.428 | -0.058 | 0.076 | 6232 |
| 4877 | 642 | nalidixic acid | 15 µM | MCF7 | -0.428 | -0.055 | 0.079 | 2297 |
| 4878 | 689 | propylthiouracil | 23 µM | PC3 | -0.428 | -0.053 | 0.081 | 4076 |
| 4879 | 772 | methocarbamol | 17 µM | MCF7 | -0.428 | -0.057 | 0.078 | 7467 |
| 4880 | 700 | lomefloxacin | 10 µM | MCF7 | -0.429 | -0.044 | 0.091 | 4745 |
| 4881 | 506 | alvespimycin | 100 nM | MCF7 | -0.429 | -0.06 | 0.074 | 993 |
| 4882 | 615 | flumequine | 15 µM | HL60 | -0.429 | -0.035 | 0.1 | 1429 |
| 4883 | 42 | LY-294002 | 10 µM | ssMCF7 | -0.429 | -0.056 | 0.079 | 401 |
| 4884 | 730 | erythromycin | 5 µM | MCF7 | -0.429 | -0.067 | 0.068 | 5329 |
| 4885 | 715 | ioversol | 5 µM | PC3 | -0.43 | -0.079 | 0.056 | 6726 |
| 4886 | 771 | pimethixene | 10 µM | MCF7 | -0.43 | -0.072 | 0.063 | 7426 |
| 4887 | 731 | racecadotril | 10 µM | PC3 | -0.43 | -0.058 | 0.076 | 5755 |
| 4888 | 689 | xylazine | 18 µM | PC3 | -0.43 | -0.063 | 0.071 | 4066 |
| 4889 | 504 | blebbistatin | 17 µM | MCF7 | -0.43 | -0.064 | 0.071 | 837 |
| 4890 | 635 | vancomycin | 3 µM | HL60 | -0.43 | -0.056 | 0.079 | 2498 |
| 4891 | 633 | metolazone | 11 µM | MCF7 | -0.431 | -0.086 | 0.049 | 1514 |
| 4892 | 69 | pirinixic acid | 100 µM | SKMEL5 | -0.431 | -0.066 | 0.069 | 495 |
| 4893 | 636 | gentamicin | 3 µM | MCF7 | -0.431 | -0.065 | 0.071 | 2245 |
| 4894 | 691 | sotalol | 13 µM | MCF7 | -0.431 | -0.047 | 0.088 | 4160 |
| 4895 | 734 | noscapine | 10 µM | PC3 | -0.431 | -0.059 | 0.076 | 5851 |
| 4896 | 715 | propranolol | 14 µM | PC3 | -0.431 | -0.07 | 0.065 | 6759 |
| 4897 | 673 | bretylium tosilate | 10 µM | MCF7 | -0.431 | -0.054 | 0.082 | 3394 |
| 4898 | 678 | piperidolate | 11 µM | MCF7 | -0.431 | -0.091 | 0.044 | 3551 |
| 4899 | 714 | hesperidin | 7 µM | PC3 | -0.431 | -0.037 | 0.098 | 6714 |
| 4900 | 627 | sulindac | 11 µM | MCF7 | -0.432 | -0.072 | 0.064 | 1693 |
| 4901 | 728 | clobetasol | 9 µM | PC3 | -0.432 | -0.058 | 0.077 | 4497 |
| 4902 | 752 | etanidazole | 19 µM | MCF7 | -0.432 | -0.058 | 0.078 | 6072 |
| 4903 | 698 | salbutamol | 17 µM | PC3 | -0.432 | -0.067 | 0.069 | 7376 |
| 4904 | 703 | guaifenesin | 20 µM | PC3 | -0.432 | -0.08 | 0.056 | 4549 |
| 4905 | 765 | LY-294002 | 10 µM | MCF7 | -0.432 | -0.047 | 0.089 | 6995 |
| 4906 | 753 | pralidoxime | 23 µM | PC3 | -0.432 | -0.064 | 0.071 | 6283 |
| 4907 | 745 | sulfinpyrazone | 10 µM | MCF7 | -0.432 | -0.056 | 0.08 | 6230 |
| 4908 | 762 | thalidomide | 15 µM | PC3 | -0.432 | -0.048 | 0.087 | 7288 |
| 4909 | 1093 | BCB000038 | 10 µM | PC3 | -0.433 | -0.076 | 0.059 | 7547 |
| 4910 | 613 | danazol | 12 µM | HL60 | -0.433 | -0.072 | 0.063 | 2038 |
| 4911 | 2 | valproic acid | 1 mM | MCF7 | -0.433 | -0.053 | 0.083 | 23 |
| 4912 | 715 | pilocarpine | 15 µM | PC3 | -0.433 | -0.063 | 0.073 | 6741 |
| 4913 | 743 | picotamide | 10 µM | MCF7 | -0.433 | -0.058 | 0.078 | 6787 |
| 4914 | 715 | ceforanide | 8 µM | PC3 | -0.433 | -0.045 | 0.091 | 6751 |
| 4915 | 1089 | pioglitazone | 10 µM | PC3 | -0.433 | -0.074 | 0.062 | 7528 |
| 4916 | 1081 | semustine | 100 µM | PC3 | -0.433 | -0.062 | 0.074 | 7492 |
| 4917 | 698 | merbromin | 5 µM | PC3 | -0.434 | -0.045 | 0.091 | 7398 |
| 4918 | 711 | metamizole sodium | 12 µM | MCF7 | -0.434 | -0.048 | 0.088 | 3929 |
| 4919 | 633 | loperamide | 8 µM | MCF7 | -0.434 | -0.042 | 0.094 | 1533 |
| 4920 | 70 | tetraethylenepentamine | 100 µM | ssMCF7 | -0.435 | -0.083 | 0.053 | 498 |
| 4921 | 701 | diphenylpyraline | 13 µM | PC3 | -0.435 | -0.055 | 0.082 | 4299 |
| 4922 | 758 | meclozine | 9 µM | MCF7 | -0.435 | -0.034 | 0.103 | 5607 |
| 4923 | 504 | 5248896 | 11 µM | MCF7 | -0.436 | -0.047 | 0.089 | 838 |
| 4924 | 710 | bretylium tosilate | 10 µM | PC3 | -0.436 | -0.067 | 0.07 | 6674 |
| 4925 | 758 | sulfafurazole | 15 µM | MCF7 | -0.436 | -0.077 | 0.06 | 5622 |
| 4926 | 728 | clomipramine | 11 µM | PC3 | -0.436 | -0.093 | 0.044 | 4487 |
| 4927 | 752 | skimmianine | 15 µM | MCF7 | -0.436 | -0.053 | 0.083 | 6066 |
| 4928 | 737 | diethylcarbamazine | 10 µM | MCF7 | -0.437 | -0.033 | 0.103 | 5485 |
| 4929 | 2a | metformin | 10 µM | MCF7 | -0.437 | -0.062 | 0.075 | 61 |
| 4930 | 683 | ciclacillin | 12 µM | PC3 | -0.437 | -0.087 | 0.05 | 3800 |
| 4931 | 662 | isocorydine | 12 µM | MCF7 | -0.437 | -0.058 | 0.079 | 2780 |
| 4932 | 744 | acepromazine | 9 µM | MCF7 | -0.438 | -0.036 | 0.101 | 6832 |
| 4933 | 44 | tetraethylenepentamine | 100 µM | HL60 | -0.438 | -0.045 | 0.092 | 412 |
| 4934 | 772 | chlorphenesin | 16 µM | MCF7 | -0.438 | -0.07 | 0.067 | 7472 |
| 4935 | 75 | gefitinib | 10 µM | HL60 | -0.438 | -0.041 | 0.096 | 541 |
| 4936 | 659 | (+)-isoprenaline | 11 µM | HL60 | -0.438 | -0.065 | 0.073 | 3046 |
| 4937 | 701 | levonorgestrel | 13 µM | PC3 | -0.438 | -0.047 | 0.09 | 4269 |
| 4938 | 727 | clozapine | 10 µM | PC3 | -0.438 | -0.043 | 0.094 | 4453 |
| 4939 | 694 | benperidol | 10 µM | MCF7 | -0.438 | -0.082 | 0.055 | 4781 |
| 4940 | 752 | terazosin | 9 µM | MCF7 | -0.439 | -0.084 | 0.054 | 6092 |
| 4941 | 636 | ivermectin | 5 µM | MCF7 | -0.439 | -0.054 | 0.084 | 2213 |
| 4942 | 703 | CP-690334-01 | 10 µM | PC3 | -0.439 | -0.076 | 0.061 | 4558 |
| 4943 | 701 | ketoprofen | 16 µM | PC3 | -0.439 | -0.073 | 0.064 | 4286 |
| 4944 | 678 | cyproterone | 10 µM | MCF7 | -0.439 | -0.063 | 0.074 | 3545 |
| 4945 | 762 | Trolox C | 16 µM | PC3 | -0.439 | -0.076 | 0.062 | 7304 |
| 4946 | 617 | scopolamine N-oxide | 10 µM | PC3 | -0.439 | -0.067 | 0.07 | 2099 |
| 4947 | 694 | minoxidil | 19 µM | MCF7 | -0.439 | -0.046 | 0.091 | 4800 |
| 4948 | 765 | thioridazine | 10 µM | MCF7 | -0.439 | -0.088 | 0.05 | 6989 |
| 4949 | 751 | sulfamerazine | 15 µM | MCF7 | -0.439 | -0.059 | 0.079 | 6036 |
| 4950 | 633 | famotidine | 12 µM | MCF7 | -0.44 | -0.074 | 0.064 | 1529 |
| 4951 | 670 | sulfadimidine | 13 µM | MCF7 | -0.44 | -0.067 | 0.071 | 3422 |
| 4952 | 656 | benperidol | 10 µM | MCF7 | -0.44 | -0.053 | 0.085 | 2836 |
| 4953 | 514 | phentolamine | 12 µM | MCF7 | -0.44 | -0.05 | 0.088 | 1138 |
| 4954 | 28 | diclofenac | 10 µM | MCF7 | -0.441 | -0.066 | 0.072 | 333 |
| 4955 | 688 | Prestwick-864 | 35 µM | PC3 | -0.441 | -0.094 | 0.044 | 3994 |
| 4956 | 753 | paracetamol | 26 µM | PC3 | -0.441 | -0.076 | 0.062 | 6284 |
| 4957 | 616 | clindamycin | 9 µM | PC3 | -0.441 | -0.046 | 0.093 | 2057 |
| 4958 | 746 | DL-thiorphan | 16 µM | MCF7 | -0.441 | -0.057 | 0.081 | 6249 |
| 4959 | 680 | Prestwick-665 | 12 µM | PC3 | -0.442 | -0.076 | 0.062 | 3681 |
| 4960 | 744 | dacarbazine | 22 µM | MCF7 | -0.442 | -0.055 | 0.083 | 6816 |
| 4961 | 764 | cefoxitin | 9 µM | PC3 | -0.442 | -0.074 | 0.065 | 7148 |
| 4962 | 690 | oxamniquine | 14 µM | MCF7 | -0.442 | -0.046 | 0.092 | 4124 |
| 4963 | 642 | dilazep | 6 µM | MCF7 | -0.443 | -0.072 | 0.067 | 2294 |
| 4964 | 744 | isocorydine | 12 µM | MCF7 | -0.443 | -0.076 | 0.063 | 6843 |
| 4965 | 513 | fluphenazine | 10 µM | MCF7 | -0.443 | -0.058 | 0.081 | 1075 |
| 4966 | 617 | xylometazoline | 14 µM | PC3 | -0.444 | -0.073 | 0.066 | 2107 |
| 4967 | 758 | aciclovir | 18 µM | MCF7 | -0.444 | -0.055 | 0.084 | 5643 |
| 4968 | 677 | roxarsone | 15 µM | MCF7 | -0.444 | -0.053 | 0.086 | 3511 |
| 4969 | 720 | alpha-ergocryptine | 7 µM | MCF7 | -0.444 | -0.041 | 0.098 | 4374 |
| 4970 | 1052 | SB-202190 | 1 µM | PC3 | -0.444 | -0.089 | 0.05 | 6882 |
| 4971 | 707 | fluspirilene | 8 µM | MCF7 | -0.445 | -0.044 | 0.096 | 5008 |
| 4972 | 756 | gliquidone | 8 µM | MCF7 | -0.445 | -0.085 | 0.055 | 6505 |
| 4973 | 764 | naproxen | 16 µM | PC3 | -0.445 | -0.051 | 0.088 | 7146 |
| 4974 | 708 | epiandrosterone | 14 µM | MCF7 | -0.445 | -0.061 | 0.078 | 5687 |
| 4975 | 653 | tacrine | 16 µM | MCF7 | -0.445 | -0.052 | 0.088 | 2633 |
| 4976 | 623 | ambroxol | 10 µM | HL60 | -0.445 | -0.043 | 0.097 | 1623 |
| 4977 | 654 | piperine | 14 µM | MCF7 | -0.445 | -0.065 | 0.074 | 3263 |
| 4978 | 732 | methacholine chloride | 20 µM | PC3 | -0.445 | -0.064 | 0.076 | 5773 |
| 4979 | 655 | digoxin | 5 µM | MCF7 | -0.445 | -0.075 | 0.064 | 3283 |
| 4980 | 663 | gramine | 23 µM | MCF7 | -0.445 | -0.054 | 0.086 | 2799 |
| 4981 | 705 | prednisone | 11 µM | MCF7 | -0.445 | -0.079 | 0.061 | 4400 |
| 4982 | 732 | ebselen | 15 µM | PC3 | -0.445 | -0.06 | 0.08 | 5778 |
| 4983 | 758 | lorglumide | 8 µM | MCF7 | -0.446 | -0.066 | 0.074 | 5619 |
| 4984 | 637 | iproniazid | 14 µM | MCF7 | -0.446 | -0.084 | 0.056 | 2288 |
| 4985 | 5 | alpha-estradiol | 10 nM | MCF7 | -0.446 | -0.06 | 0.08 | 122 |
| 4986 | 761 | talampicillin | 8 µM | PC3 | -0.446 | -0.077 | 0.063 | 7254 |
| 4987 | 711 | sulfadimidine | 13 µM | MCF7 | -0.446 | -0.068 | 0.072 | 3940 |
| 4988 | 656 | tenoxicam | 12 µM | MCF7 | -0.446 | -0.055 | 0.085 | 2860 |
| 4989 | 709 | naphazoline | 16 µM | PC3 | -0.446 | -0.044 | 0.096 | 6604 |
| 4990 | 616 | canrenoic acid | 10 µM | PC3 | -0.446 | -0.053 | 0.087 | 2065 |
| 4991 | 732 | azlocillin | 8 µM | PC3 | -0.447 | -0.064 | 0.076 | 5788 |
| 4992 | 40 | tretinoin | 1 µM | MCF7 | -0.447 | -0.07 | 0.07 | 384 |
| 4993 | 636 | nalbuphine | 10 µM | MCF7 | -0.447 | -0.081 | 0.059 | 2225 |
| 4994 | 748 | bambuterol | 10 µM | MCF7 | -0.447 | -0.072 | 0.069 | 7239 |
| 4995 | 502 | tanespimycin | 1 µM | MCF7 | -0.447 | -0.049 | 0.091 | 947 |
| 4996 | 758 | amiodarone | 6 µM | MCF7 | -0.447 | -0.046 | 0.094 | 5618 |
| 4997 | 765 | sirolimus | 100 nM | MCF7 | -0.447 | -0.068 | 0.073 | 6981 |
| 4998 | 748 | milrinone | 19 µM | MCF7 | -0.448 | -0.073 | 0.067 | 7210 |
| 4999 | 74 | tanespimycin | 1 µM | ssMCF7 | -0.448 | -0.072 | 0.069 | 521 |
| 5000 | 633 | erythromycin | 5 µM | MCF7 | -0.448 | -0.065 | 0.076 | 1510 |
| 5001 | 761 | naftifine | 12 µM | PC3 | -0.449 | -0.063 | 0.077 | 7273 |
| 5002 | 710 | etoposide | 7 µM | PC3 | -0.449 | -0.083 | 0.058 | 6681 |
| 5003 | 720 | tinidazole | 16 µM | MCF7 | -0.449 | -0.053 | 0.088 | 4370 |
| 5004 | 754 | trifluoperazine | 8 µM | PC3 | -0.449 | -0.058 | 0.083 | 6341 |
| 5005 | 695 | succinylsulfathiazole | 11 µM | MCF7 | -0.449 | -0.058 | 0.082 | 4847 |
| 5006 | 734 | harmine | 16 µM | PC3 | -0.449 | -0.079 | 0.062 | 5855 |
| 5007 | 755 | flucytosine | 31 µM | MCF7 | -0.449 | -0.064 | 0.077 | 6450 |
| 5008 | 637 | gemfibrozil | 16 µM | MCF7 | -0.449 | -0.074 | 0.066 | 2277 |
| 5009 | 683 | CP-690334-01 | 10 µM | PC3 | -0.449 | -0.072 | 0.068 | 3823 |
| 5010 | 727 | tretinoin | 1 µM | PC3 | -0.449 | -0.045 | 0.096 | 4435 |
| 5011 | 767 | sirolimus | 100 nM | MCF7 | -0.449 | -0.081 | 0.059 | 6958 |
| 5012 | 686 | solasodine | 10 µM | MCF7 | -0.449 | -0.053 | 0.088 | 3830 |
| 5013 | 735 | dextromethorphan | 11 µM | MCF7 | -0.45 | -0.055 | 0.086 | 5401 |
| 5014 | 732 | demecarium bromide | 6 µM | PC3 | -0.45 | -0.075 | 0.066 | 5795 |
| 5015 | 741 | epirizole | 17 µM | MCF7 | -0.45 | -0.072 | 0.069 | 5995 |
| 5016 | 747 | kaempferol | 14 µM | MCF7 | -0.45 | -0.077 | 0.064 | 7196 |
| 5017 | 701 | gossypol | 8 µM | PC3 | -0.45 | -0.047 | 0.094 | 4296 |
| 5018 | 752 | ramifenazone | 14 µM | MCF7 | -0.45 | -0.058 | 0.083 | 6097 |
| 5019 | 613 | baclofen | 19 µM | HL60 | -0.45 | -0.049 | 0.092 | 2036 |
| 5020 | 765 | valproic acid | 1 mM | MCF7 | -0.451 | -0.058 | 0.083 | 6969 |
| 5021 | 701 | bromopride | 12 µM | PC3 | -0.451 | -0.032 | 0.109 | 4278 |
| 5022 | 719 | nisoxetine | 13 µM | PC3 | -0.451 | -0.058 | 0.084 | 5091 |
| 5023 | 758 | cefalexin | 11 µM | MCF7 | -0.451 | -0.074 | 0.067 | 5615 |
| 5024 | 614 | gallamine triethiodide | 4 µM | HL60 | -0.451 | -0.05 | 0.091 | 1375 |
| 5025 | 758 | dipyridamole | 8 µM | MCF7 | -0.452 | -0.048 | 0.094 | 5617 |
| 5026 | 504 | prazosin | 10 µM | MCF7 | -0.452 | -0.096 | 0.046 | 826 |
| 5027 | 513 | wortmannin | 10 nM | MCF7 | -0.452 | -0.076 | 0.066 | 1081 |
| 5028 | 733 | tetrandrine | 6 µM | PC3 | -0.452 | -0.082 | 0.06 | 5821 |
| 5029 | 672 | fosfosal | 18 µM | MCF7 | -0.453 | -0.081 | 0.061 | 3336 |
| 5030 | 1074 | CAY-10397 | 10 µM | MCF7 | -0.453 | -0.072 | 0.07 | 7082 |
| 5031 | 738 | deptropine | 8 µM | MCF7 | -0.453 | -0.067 | 0.075 | 5543 |
| 5032 | 695 | theophylline | 20 µM | MCF7 | -0.453 | -0.053 | 0.089 | 4812 |
| 5033 | 73 | wortmannin | 10 nM | SKMEL5 | -0.453 | -0.082 | 0.06 | 506 |
| 5034 | 671 | sulfametoxydiazine | 14 µM | MCF7 | -0.454 | -0.068 | 0.074 | 3453 |
| 5035 | 746 | cefuroxime | 9 µM | MCF7 | -0.454 | -0.063 | 0.08 | 6261 |
| 5036 | 704 | cefixime | 9 µM | PC3 | -0.454 | -0.063 | 0.079 | 4567 |
| 5037 | 744 | zomepirac | 13 µM | MCF7 | -0.454 | -0.082 | 0.06 | 6815 |
| 5038 | 513 | sirolimus | 100 nM | MCF7 | -0.454 | -0.065 | 0.077 | 1059 |
| 5039 | 642 | ketoprofen | 16 µM | MCF7 | -0.454 | -0.067 | 0.075 | 2316 |
| 5040 | 712 | tonzonium bromide | 7 µM | PC3 | -0.454 | -0.052 | 0.09 | 4617 |
| 5041 | 687 | citalopram | 1 µM | MCF7 | -0.454 | -0.075 | 0.067 | 3903 |
| 5042 | 734 | cefmetazole | 8 µM | PC3 | -0.454 | -0.063 | 0.08 | 5868 |
| 5043 | 700 | benzamil | 11 µM | MCF7 | -0.455 | -0.075 | 0.068 | 4760 |
| 5044 | 610 | cinchocaine | 12 µM | PC3 | -0.455 | -0.079 | 0.063 | 1889 |
| 5045 | 82 | copper sulfate | 100 µM | MCF7 | -0.455 | -0.054 | 0.088 | 575 |
| 5046 | 681 | lomefloxacin | 10 µM | PC3 | -0.455 | -0.08 | 0.062 | 3723 |
| 5047 | 626 | clozapine | 10 µM | MCF7 | -0.455 | -0.086 | 0.057 | 1654 |
| 5048 | 706 | Prestwick-857 | 12 µM | MCF7 | -0.455 | -0.065 | 0.078 | 4980 |
| 5049 | 755 | estrone | 15 µM | MCF7 | -0.455 | -0.074 | 0.069 | 6448 |
| 5050 | 766 | viomycin | 5 µM | MCF7 | -0.455 | -0.084 | 0.058 | 7036 |
| 5051 | 727 | wortmannin | 10 nM | PC3 | -0.456 | -0.083 | 0.06 | 4467 |
| 5052 | 90 | resveratrol | 50 µM | PC3 | -0.456 | -0.074 | 0.069 | 662 |
| 5053 | 731 | tetracycline | 8 µM | PC3 | -0.456 | -0.075 | 0.068 | 5757 |
| 5054 | 685 | 0179445-0000 | 1 µM | MCF7 | -0.456 | -0.071 | 0.072 | 3633 |
| 5055 | 711 | solasodine | 10 µM | MCF7 | -0.456 | -0.049 | 0.094 | 3924 |
| 5056 | 603 | estradiol | 100 nM | PC3 | -0.456 | -0.058 | 0.085 | 1208 |
| 5057 | 715 | cyproheptadine | 12 µM | PC3 | -0.456 | -0.07 | 0.074 | 6740 |
| 5058 | 772 | triflupromazine | 10 µM | MCF7 | -0.456 | -0.071 | 0.072 | 7466 |
| 5059 | 738 | telenzepine | 9 µM | MCF7 | -0.457 | -0.067 | 0.076 | 5521 |
| 5060 | 741 | nifenazone | 13 µM | MCF7 | -0.457 | -0.077 | 0.066 | 6016 |
| 5061 | 686 | mexiletine | 19 µM | MCF7 | -0.457 | -0.058 | 0.086 | 3862 |
| 5062 | 502 | Y-27632 | 3 µM | MCF7 | -0.458 | -0.077 | 0.066 | 948 |
| 5063 | 513 | nordihydroguaiaretic acid | 1 µM | MCF7 | -0.458 | -0.089 | 0.054 | 1061 |
| 5064 | 693 | indoprofen | 14 µM | PC3 | -0.458 | -0.081 | 0.063 | 4249 |
| 5065 | 651 | clidinium bromide | 9 µM | HL60 | -0.458 | -0.067 | 0.076 | 2734 |
| 5066 | 710 | lisuride | 12 µM | PC3 | -0.459 | -0.078 | 0.065 | 6682 |
| 5067 | 735 | clebopride | 8 µM | MCF7 | -0.459 | -0.097 | 0.047 | 5412 |
| 5068 | 702 | eldeline | 8 µM | PC3 | -0.459 | -0.06 | 0.084 | 4306 |
| 5069 | 648 | trioxysalen | 18 µM | HL60 | -0.459 | -0.054 | 0.09 | 2516 |
| 5070 | 727 | fluphenazine | 10 µM | PC3 | -0.459 | -0.06 | 0.084 | 4461 |
| 5071 | 677 | asiaticoside | 4 µM | MCF7 | -0.459 | -0.048 | 0.096 | 3504 |
| 5072 | 752 | ricinine | 24 µM | MCF7 | -0.46 | -0.052 | 0.092 | 6067 |
| 5073 | 699 | dilazep | 6 µM | MCF7 | -0.46 | -0.063 | 0.081 | 4688 |
| 5074 | 747 | tetrandrine | 6 µM | MCF7 | -0.46 | -0.09 | 0.054 | 7178 |
| 5075 | 670 | flunixin | 8 µM | MCF7 | -0.46 | -0.051 | 0.094 | 3411 |
| 5076 | 743 | cycloserine | 39 µM | MCF7 | -0.46 | -0.05 | 0.094 | 6782 |
| 5077 | 655 | cefotiam | 7 µM | MCF7 | -0.46 | -0.086 | 0.058 | 3319 |
| 5078 | 734 | convolamine | 12 µM | PC3 | -0.461 | -0.051 | 0.094 | 5876 |
| 5079 | 637 | ornidazole | 18 µM | MCF7 | -0.461 | -0.062 | 0.082 | 2272 |
| 5080 | 728 | amrinone | 21 µM | PC3 | -0.461 | -0.072 | 0.073 | 4488 |
| 5081 | 676 | pronetalol | 15 µM | MCF7 | -0.462 | -0.085 | 0.06 | 7322 |
| 5082 | 686 | proglumide | 12 µM | MCF7 | -0.462 | -0.066 | 0.079 | 3861 |
| 5083 | 603 | geldanamycin | 1 µM | PC3 | -0.462 | -0.088 | 0.057 | 1228 |
| 5084 | 692 | colistin | 3 µM | PC3 | -0.462 | -0.054 | 0.091 | 4212 |
| 5085 | 673 | capsaicin | 13 µM | MCF7 | -0.462 | -0.051 | 0.094 | 3372 |
| 5086 | 702 | citiolone | 25 µM | PC3 | -0.462 | -0.067 | 0.078 | 4311 |
| 5087 | 20 | ciclosporin | 1 µM | MCF7 | -0.463 | -0.053 | 0.092 | 261 |
| 5088 | 644 | diflorasone | 8 µM | HL60 | -0.463 | -0.054 | 0.091 | 2142 |
| 5089 | 762 | meticrane | 15 µM | PC3 | -0.463 | -0.059 | 0.086 | 7282 |
| 5090 | 748 | mesalazine | 26 µM | MCF7 | -0.463 | -0.055 | 0.091 | 7241 |
| 5091 | 689 | tiaprofenic acid | 15 µM | PC3 | -0.463 | -0.069 | 0.077 | 4091 |
| 5092 | 103 | monastrol | 20 µM | MCF7 | -0.463 | -0.082 | 0.063 | 614 |
| 5093 | 747 | natamycin | 6 µM | MCF7 | -0.464 | -0.08 | 0.065 | 7167 |
| 5094 | 678 | Prestwick-1085 | 15 µM | MCF7 | -0.464 | -0.043 | 0.103 | 3554 |
| 5095 | 101 | LM-1685 | 10 µM | MCF7 | -0.464 | -0.091 | 0.055 | 612 |
| 5096 | 656 | R-atenolol | 15 µM | MCF7 | -0.465 | -0.084 | 0.061 | 2855 |
| 5097 | 765 | tanespimycin | 1 µM | MCF7 | -0.465 | -0.097 | 0.049 | 6978 |
| 5098 | 732 | methanthelinium bromide | 10 µM | PC3 | -0.465 | -0.058 | 0.088 | 5780 |
| 5099 | 1054 | NS-398 | 10 µM | PC3 | -0.465 | -0.047 | 0.099 | 6892 |
| 5100 | 727 | sirolimus | 100 nM | PC3 | -0.465 | -0.067 | 0.079 | 4466 |
| 5101 | 672 | iodixanol | 3 µM | MCF7 | -0.465 | -0.072 | 0.074 | 3362 |
| 5102 | 748 | noscapine | 10 µM | MCF7 | -0.466 | -0.073 | 0.073 | 7204 |
| 5103 | 698 | isocarboxazid | 17 µM | PC3 | -0.466 | -0.064 | 0.082 | 7383 |
| 5104 | 698 | ofloxacin | 11 µM | PC3 | -0.466 | -0.058 | 0.088 | 7372 |
| 5105 | 741 | lincomycin | 9 µM | MCF7 | -0.466 | -0.07 | 0.076 | 5992 |
| 5106 | 513 | haloperidol | 10 µM | MCF7 | -0.466 | -0.084 | 0.062 | 1041 |
| 5107 | 653 | ouabain | 5 µM | MCF7 | -0.466 | -0.058 | 0.088 | 2656 |
| 5108 | 698 | metaraminol | 9 µM | PC3 | -0.466 | -0.065 | 0.081 | 7368 |
| 5109 | 1092 | CP-944629 | 10 µM | MCF7 | -0.467 | -0.055 | 0.092 | 7544 |
| 5110 | 732 | cinoxacin | 15 µM | PC3 | -0.467 | -0.04 | 0.107 | 5783 |
| 5111 | 26b | arachidonyltrifluoromethane | 10 µM | MCF7 | -0.468 | -0.056 | 0.091 | 327 |
| 5112 | 506 | valproic acid | 50 µM | MCF7 | -0.468 | -0.076 | 0.07 | 1002 |
| 5113 | 755 | aminohippuric acid | 21 µM | MCF7 | -0.468 | -0.051 | 0.096 | 6453 |
| 5114 | 695 | cyclopenthiazide | 11 µM | MCF7 | -0.469 | -0.074 | 0.073 | 4813 |
| 5115 | 689 | trimethadione | 28 µM | PC3 | -0.469 | -0.058 | 0.089 | 4086 |
| 5116 | 689 | diflorasone | 8 µM | PC3 | -0.469 | -0.05 | 0.097 | 4077 |
| 5117 | 741 | ketorolac | 11 µM | MCF7 | -0.469 | -0.068 | 0.079 | 5988 |
| 5118 | 655 | niridazole | 19 µM | MCF7 | -0.469 | -0.067 | 0.08 | 3301 |
| 5119 | 701 | lomefloxacin | 10 µM | PC3 | -0.469 | -0.07 | 0.077 | 4281 |
| 5120 | 689 | carteolol | 12 µM | PC3 | -0.469 | -0.052 | 0.095 | 4096 |
| 5121 | 693 | dydrogesterone | 13 µM | PC3 | -0.469 | -0.083 | 0.064 | 4254 |
| 5122 | 615 | tiratricol | 6 µM | HL60 | -0.47 | -0.052 | 0.095 | 1412 |
| 5123 | 692 | atovaquone | 11 µM | PC3 | -0.47 | -0.087 | 0.06 | 4201 |
| 5124 | 623 | amikacin | 7 µM | HL60 | -0.47 | -0.044 | 0.103 | 1618 |
| 5125 | 502 | wortmannin | 1 µM | MCF7 | -0.47 | -0.051 | 0.096 | 977 |
| 5126 | 655 | felodipine | 10 µM | MCF7 | -0.47 | -0.063 | 0.084 | 3295 |
| 5127 | 734 | neomycin | 4 µM | PC3 | -0.47 | -0.079 | 0.069 | 5867 |
| 5128 | 751 | flunisolide | 9 µM | MCF7 | -0.47 | -0.074 | 0.074 | 6023 |
| 5129 | 737 | chlorpromazine | 11 µM | MCF7 | -0.471 | -0.056 | 0.092 | 5493 |
| 5130 | 687 | withaferin A | 1 µM | MCF7 | -0.471 | -0.091 | 0.057 | 3902 |
| 5131 | 713 | dipyridamole | 8 µM | PC3 | -0.471 | -0.065 | 0.083 | 4656 |
| 5132 | 756 | denatonium benzoate | 9 µM | MCF7 | -0.471 | -0.079 | 0.069 | 6502 |
| 5133 | 698 | griseofulvin | 11 µM | PC3 | -0.471 | -0.075 | 0.073 | 7363 |
| 5134 | 761 | dyclonine | 12 µM | PC3 | -0.472 | -0.046 | 0.102 | 7261 |
| 5135 | 627 | hydroflumethiazide | 12 µM | MCF7 | -0.472 | -0.087 | 0.061 | 1687 |
| 5136 | 762 | norfloxacin | 13 µM | PC3 | -0.472 | -0.069 | 0.079 | 7283 |
| 5137 | 632 | troleandomycin | 5 µM | MCF7 | -0.472 | -0.088 | 0.06 | 1465 |
| 5138 | 13 | raloxifene | 100 nM | MCF7 | -0.472 | -0.07 | 0.078 | 202 |
| 5139 | 611 | baclofen | 19 µM | PC3 | -0.472 | -0.097 | 0.051 | 1952 |
| 5140 | 748 | diclofenac | 13 µM | MCF7 | -0.472 | -0.056 | 0.092 | 7215 |
| 5141 | 710 | suloctidil | 12 µM | PC3 | -0.472 | -0.067 | 0.081 | 6675 |
| 5142 | 680 | ofloxacin | 11 µM | PC3 | -0.472 | -0.055 | 0.093 | 3673 |
| 5143 | 690 | pheniramine | 11 µM | MCF7 | -0.472 | -0.074 | 0.074 | 4130 |
| 5144 | 617 | methocarbamol | 17 µM | PC3 | -0.473 | -0.094 | 0.054 | 2111 |
| 5145 | 686 | lanatoside C | 4 µM | MCF7 | -0.473 | -0.041 | 0.107 | 3852 |
| 5146 | 746 | mianserin | 13 µM | MCF7 | -0.473 | -0.084 | 0.064 | 6260 |
| 5147 | 703 | phenelzine | 17 µM | PC3 | -0.473 | -0.075 | 0.073 | 4538 |
| 5148 | 699 | nitrofurantoin | 17 µM | MCF7 | -0.473 | -0.077 | 0.072 | 4697 |
| 5149 | 719 | prednicarbate | 8 µM | PC3 | -0.473 | -0.054 | 0.094 | 5119 |
| 5150 | 772 | acetazolamide | 18 µM | MCF7 | -0.474 | -0.058 | 0.091 | 7461 |
| 5151 | 767 | monorden | 100 nM | MCF7 | -0.474 | -0.085 | 0.064 | 6938 |
| 5152 | 727 | haloperidol | 10 µM | PC3 | -0.474 | -0.078 | 0.071 | 4427 |
| 5153 | 745 | cefepime | 7 µM | MCF7 | -0.474 | -0.097 | 0.051 | 6237 |
| 5154 | 655 | pilocarpine | 15 µM | MCF7 | -0.474 | -0.07 | 0.079 | 3300 |
| 5155 | 623 | methapyrilene | 13 µM | HL60 | -0.474 | -0.041 | 0.107 | 1588 |
| 5156 | 615 | pargyline | 20 µM | HL60 | -0.474 | -0.045 | 0.104 | 1418 |
| 5157 | 748 | gentamicin | 3 µM | MCF7 | -0.474 | -0.058 | 0.091 | 7237 |
| 5158 | 732 | dorzolamide | 11 µM | PC3 | -0.475 | -0.049 | 0.1 | 5785 |
| 5159 | 745 | raloxifene | 8 µM | MCF7 | -0.475 | -0.056 | 0.093 | 6235 |
| 5160 | 700 | ketoprofen | 16 µM | MCF7 | -0.475 | -0.099 | 0.05 | 4751 |
| 5161 | 707 | edrophonium chloride | 20 µM | MCF7 | -0.475 | -0.08 | 0.069 | 5001 |
| 5162 | 692 | conessine | 11 µM | PC3 | -0.475 | -0.059 | 0.09 | 4191 |
| 5163 | 636 | dihydrostreptomycin | 3 µM | MCF7 | -0.475 | -0.053 | 0.096 | 2237 |
| 5164 | 656 | colistin | 3 µM | MCF7 | -0.475 | -0.07 | 0.079 | 2851 |
| 5165 | 756 | proxyphylline | 17 µM | MCF7 | -0.476 | -0.068 | 0.082 | 6494 |
| 5166 | 689 | metrizamide | 5 µM | PC3 | -0.476 | -0.08 | 0.069 | 4075 |
| 5167 | 714 | glycocholic acid | 9 µM | PC3 | -0.476 | -0.093 | 0.056 | 6716 |
| 5168 | 63 | deferoxamine | 100 µM | PC3 | -0.476 | -0.067 | 0.082 | 460 |
| 5169 | 748 | benzethonium chloride | 9 µM | MCF7 | -0.476 | -0.059 | 0.091 | 7207 |
| 5170 | 765 | rosiglitazone | 10 µM | MCF7 | -0.476 | -0.074 | 0.076 | 6992 |
| 5171 | 689 | cefsulodin | 7 µM | PC3 | -0.476 | -0.061 | 0.089 | 4067 |
| 5172 | 738 | todralazine | 15 µM | MCF7 | -0.477 | -0.065 | 0.084 | 5512 |
| 5173 | 685 | lomefloxacin | 10 µM | MCF7 | -0.477 | -0.085 | 0.065 | 3620 |
| 5174 | 603 | LY-294002 | 10 µM | PC3 | -0.477 | -0.053 | 0.097 | 1239 |
| 5175 | 36 | imatinib | 10 µM | MCF7 | -0.478 | -0.089 | 0.06 | 366 |
| 5176 | 707 | ouabain | 5 µM | MCF7 | -0.478 | -0.072 | 0.078 | 5026 |
| 5177 | 655 | glycocholic acid | 9 µM | MCF7 | -0.478 | -0.057 | 0.093 | 3315 |
| 5178 | 622 | etacrynic acid | 13 µM | HL60 | -0.478 | -0.048 | 0.101 | 1565 |
| 5179 | 694 | atovaquone | 11 µM | MCF7 | -0.478 | -0.061 | 0.089 | 4786 |
| 5180 | 626 | LY-294002 | 10 µM | MCF7 | -0.478 | -0.073 | 0.077 | 1652 |
| 5181 | 623 | betamethasone | 10 µM | HL60 | -0.478 | -0.052 | 0.098 | 1590 |
| 5182 | 712 | niridazole | 19 µM | PC3 | -0.479 | -0.063 | 0.088 | 4621 |
| 5183 | 622 | cinnarizine | 11 µM | HL60 | -0.479 | -0.05 | 0.1 | 1558 |
| 5184 | 506 | geldanamycin | 1 µM | MCF7 | -0.479 | -0.074 | 0.077 | 1008 |
| 5185 | 709 | Prestwick-682 | 6 µM | PC3 | -0.479 | -0.073 | 0.078 | 6638 |
| 5186 | 755 | prazosin | 10 µM | MCF7 | -0.479 | -0.085 | 0.066 | 6475 |
| 5187 | 745 | cyclopentolate | 12 µM | MCF7 | -0.479 | -0.077 | 0.073 | 6214 |
| 5188 | 735 | hexetidine | 12 µM | MCF7 | -0.48 | -0.047 | 0.103 | 5420 |
| 5189 | 614 | ampyrone | 20 µM | HL60 | -0.48 | -0.081 | 0.07 | 1402 |
| 5190 | 1010 | valinomycin | 100 nM | MCF7 | -0.48 | -0.06 | 0.09 | 5957 |
| 5191 | 637 | ethosuximide | 28 µM | MCF7 | -0.48 | -0.067 | 0.084 | 2280 |
| 5192 | 736 | tolfenamic acid | 15 µM | MCF7 | -0.48 | -0.044 | 0.107 | 5454 |
| 5193 | 736 | S-propranolol | 14 µM | MCF7 | -0.48 | -0.084 | 0.066 | 5444 |
| 5194 | 758 | thioperamide | 10 µM | MCF7 | -0.48 | -0.068 | 0.083 | 5635 |
| 5195 | 678 | (-)-isoprenaline | 16 µM | MCF7 | -0.481 | -0.065 | 0.086 | 3571 |
| 5196 | 764 | pimozide | 9 µM | PC3 | -0.481 | -0.066 | 0.085 | 7132 |
| 5197 | 758 | butyl hydroxybenzoate | 21 µM | MCF7 | -0.481 | -0.079 | 0.072 | 5608 |
| 5198 | 730 | (-)-atenolol | 15 µM | MCF7 | -0.482 | -0.085 | 0.066 | 5325 |
| 5199 | 706 | glimepiride | 8 µM | MCF7 | -0.482 | -0.072 | 0.079 | 4973 |
| 5200 | 603 | genistein | 10 µM | PC3 | -0.482 | -0.075 | 0.076 | 1235 |
| 5201 | 695 | procyclidine | 12 µM | MCF7 | -0.482 | -0.047 | 0.104 | 4817 |
| 5202 | 698 | nitrofurantoin | 17 µM | PC3 | -0.482 | -0.093 | 0.058 | 7373 |
| 5203 | 702 | flunisolide | 9 µM | PC3 | -0.482 | -0.047 | 0.104 | 4303 |
| 5204 | 753 | nitrendipine | 11 µM | PC3 | -0.482 | -0.081 | 0.07 | 6304 |
| 5205 | 703 | spiradoline | 1 µM | PC3 | -0.482 | -0.064 | 0.087 | 4553 |
| 5206 | 738 | rimexolone | 11 µM | MCF7 | -0.484 | -0.054 | 0.098 | 5517 |
| 5207 | 750 | tanespimycin | 1 µM | HL60 | -0.484 | -0.049 | 0.103 | 6184 |
| 5208 | 682 | promazine | 12 µM | PC3 | -0.485 | -0.077 | 0.075 | 3752 |
| 5209 | 737 | piracetam | 28 µM | MCF7 | -0.485 | -0.074 | 0.078 | 5462 |
| 5210 | 623 | kanamycin | 7 µM | HL60 | -0.485 | -0.063 | 0.089 | 1609 |
| 5211 | 715 | bisoprolol | 9 µM | PC3 | -0.486 | -0.062 | 0.091 | 6748 |
| 5212 | 744 | oxyphenbutazone | 12 µM | MCF7 | -0.486 | -0.077 | 0.076 | 6844 |
| 5213 | 645 | helveticoside | 7 µM | HL60 | -0.487 | -0.065 | 0.088 | 2192 |
| 5214 | 735 | prazosin | 10 µM | MCF7 | -0.487 | -0.096 | 0.057 | 5416 |
| 5215 | 677 | ioxaglic acid | 3 µM | MCF7 | -0.487 | -0.074 | 0.079 | 3528 |
| 5216 | 611 | hydroxyzine | 9 µM | PC3 | -0.487 | -0.075 | 0.078 | 1941 |
| 5217 | 727 | rosiglitazone | 10 µM | PC3 | -0.487 | -0.084 | 0.069 | 4457 |
| 5218 | 727 | trifluoperazine | 10 µM | PC3 | -0.487 | -0.06 | 0.093 | 4448 |
| 5219 | 1006 | LY-294002 | 10 µM | MCF7 | -0.487 | -0.075 | 0.077 | 5937 |
| 5220 | 654 | betulinic acid | 9 µM | MCF7 | -0.487 | -0.088 | 0.065 | 3281 |
| 5221 | 728 | riboflavin | 11 µM | PC3 | -0.488 | -0.076 | 0.077 | 4485 |
| 5222 | 637 | aztreonam | 9 µM | MCF7 | -0.488 | -0.07 | 0.083 | 2282 |
| 5223 | 627 | amitriptyline | 13 µM | MCF7 | -0.488 | -0.099 | 0.054 | 1701 |
| 5224 | 632 | isoxsuprine | 12 µM | MCF7 | -0.488 | -0.089 | 0.064 | 1485 |
| 5225 | 771 | bufexamac | 18 µM | MCF7 | -0.488 | -0.084 | 0.069 | 7413 |
| 5226 | 713 | cotinine | 23 µM | PC3 | -0.488 | -0.036 | 0.117 | 4650 |
| 5227 | 641 | calycanthine | 12 µM | HL60 | -0.488 | -0.05 | 0.103 | 1771 |
| 5228 | 772 | etomidate | 16 µM | MCF7 | -0.488 | -0.079 | 0.074 | 7460 |
| 5229 | 719 | aztreonam | 9 µM | PC3 | -0.488 | -0.052 | 0.101 | 5110 |
| 5230 | 33 | valproic acid | 500 µM | MCF7 | -0.489 | -0.052 | 0.101 | 347 |
| 5231 | 676 | letrozole | 14 µM | MCF7 | -0.489 | -0.09 | 0.063 | 7336 |
| 5232 | 767 | tretinoin | 1 µM | MCF7 | -0.489 | -0.048 | 0.106 | 6931 |
| 5233 | 703 | levcycloserine | 39 µM | PC3 | -0.489 | -0.062 | 0.092 | 4524 |
| 5234 | 710 | prochlorperazine | 7 µM | PC3 | -0.489 | -0.049 | 0.105 | 6664 |
| 5235 | 719 | thiocolchicoside | 7 µM | PC3 | -0.489 | -0.063 | 0.091 | 5095 |
| 5236 | 704 | etidronic acid | 16 µM | PC3 | -0.489 | -0.081 | 0.073 | 4564 |
| 5237 | 694 | isoxsuprine | 12 µM | MCF7 | -0.489 | -0.054 | 0.099 | 4789 |
| 5238 | 617 | antimycin A | 7 µM | PC3 | -0.49 | -0.07 | 0.083 | 2098 |
| 5239 | 736 | procaine | 15 µM | MCF7 | -0.49 | -0.068 | 0.086 | 5430 |
| 5240 | 651 | cefoxitin | 9 µM | HL60 | -0.49 | -0.068 | 0.085 | 2735 |
| 5241 | 1019 | valdecoxib | 10 µM | PC3 | -0.49 | -0.083 | 0.071 | 6378 |
| 5242 | 734 | nomifensine | 11 µM | PC3 | -0.49 | -0.04 | 0.114 | 5863 |
| 5243 | 757 | LY-294002 | 10 µM | MCF7 | -0.491 | -0.073 | 0.081 | 5599 |
| 5244 | 718 | pivampicillin | 9 µM | PC3 | -0.491 | -0.08 | 0.074 | 5046 |
| 5245 | 726 | amiodarone | 6 µM | MCF7 | -0.491 | -0.086 | 0.068 | 5253 |
| 5246 | 704 | etamsylate | 15 µM | PC3 | -0.491 | -0.074 | 0.08 | 4576 |
| 5247 | 758 | guanfacine | 14 µM | MCF7 | -0.491 | -0.035 | 0.119 | 5621 |
| 5248 | 672 | Prestwick-857 | 12 µM | MCF7 | -0.492 | -0.089 | 0.065 | 3355 |
| 5249 | 623 | ketanserin | 7 µM | HL60 | -0.492 | -0.059 | 0.095 | 1593 |
| 5250 | 733 | strophanthidin | 10 µM | PC3 | -0.493 | -0.067 | 0.087 | 5826 |
| 5251 | 767 | haloperidol | 10 µM | MCF7 | -0.494 | -0.07 | 0.085 | 6923 |
| 5252 | 732 | cefuroxime | 9 µM | PC3 | -0.494 | -0.057 | 0.098 | 5787 |
| 5253 | 1028 | F0447-0125 | 10 µM | MCF7 | -0.494 | -0.083 | 0.072 | 6424 |
| 5254 | 698 | mephenesin | 22 µM | PC3 | -0.494 | -0.073 | 0.081 | 7374 |
| 5255 | 729 | diltiazem | 9 µM | MCF7 | -0.494 | -0.062 | 0.093 | 5309 |
| 5256 | 628 | 8-azaguanine | 26 µM | PC3 | -0.494 | -0.079 | 0.076 | 1791 |
| 5257 | 767 | geldanamycin | 1 µM | MCF7 | -0.495 | -0.077 | 0.079 | 6946 |
| 5258 | 720 | citalopram | 1 µM | MCF7 | -0.495 | -0.091 | 0.064 | 4377 |
| 5259 | 700 | myosmine | 27 µM | MCF7 | -0.495 | -0.07 | 0.086 | 4759 |
| 5260 | 748 | alimemazine | 5 µM | MCF7 | -0.495 | -0.09 | 0.065 | 7235 |
| 5261 | 712 | eticlopride | 11 µM | PC3 | -0.495 | -0.086 | 0.069 | 4634 |
| 5262 | 645 | gossypol | 8 µM | HL60 | -0.495 | -0.069 | 0.086 | 2202 |
| 5263 | 758 | clozapine | 12 µM | MCF7 | -0.496 | -0.056 | 0.1 | 5630 |
| 5264 | 617 | nifenazone | 13 µM | PC3 | -0.496 | -0.08 | 0.076 | 2122 |
| 5265 | 680 | mebendazole | 14 µM | PC3 | -0.496 | -0.057 | 0.098 | 3671 |
| 5266 | 772 | triflusal | 16 µM | MCF7 | -0.496 | -0.092 | 0.063 | 7451 |
| 5267 | 767 | 15-delta prostaglandin J2 | 10 µM | MCF7 | -0.496 | -0.079 | 0.077 | 6948 |
[truncated: 63,801 more chars]
